# Differential Selective Pressures Experienced by the Aurora Kinase Gene Family

**DOI:** 10.3390/ijms19010072

**Published:** 2017-12-28

**Authors:** Joni M. Seeling, Alexis A. Farmer, Adam Mansfield, Hyuk Cho, Madhusudan Choudhary

**Affiliations:** 1 Department of Biology, Lone Star College, Woodlands, TX 77375, USA; 2 Department of Biological Sciences, Sam Houston State University, Huntsville, TX 77341, USA; aafarmer@shsu.edu; 3 Department of Computer Science, Sam Houston State University, Huntsville, TX 77341, USA; ajm090@shsu.edu (A.M.); hyukcho@shsu.edu (H.C.)

**Keywords:** aurora kinase, molecular phylogeny, synonymous, nonsynonymous

## Abstract

Aurora kinases (AKs) are serine/threonine kinases that are essential for cell division. Humans have three AK genes: *AKA*, *AKB*, and *AKC*. AKA is required for centrosome assembly, centrosome separation, and bipolar spindle assembly, and its mutation leads to abnormal spindle morphology. AKB is required for the spindle checkpoint and proper cytokinesis, and mutations cause chromosome misalignment and cytokinesis failure. AKC is expressed in germ cells, and has a role in meiosis analogous to that of AKB in mitosis. Mutation of any of the three isoforms can lead to cancer. AK proteins possess divergent N- and C-termini and a conserved central catalytic domain. We examined the evolution of the AK gene family using an identity matrix and by building a phylogenetic tree. The data suggest that *AKA* is the vertebrate ancestral gene, and that *AKB* and *AKC* resulted from gene duplication in placental mammals. In a nonsynonymous/synonymous rate substitution analysis, we found that *AKB* experienced the strongest, and *AKC* the weakest, purifying selection. Both the N- and C-termini and regions within the kinase domain experienced differential selection among the AK isoforms. These differentially selected sequences may be important for species specificity and isoform specificity, and are therefore potential therapeutic targets.

## 1. Introduction

Aurora kinases (AKs) are serine/threonine kinases that are essential for the orderly progression of mitotic and/or meiotic events in eukaryotic cells. Fungi possess a single aurora gene, whereas invertebrates and nonmammalian vertebrates have two, and mammals have three. The aurora kinase genes are relatively well conserved. Overall, the identity between human AKA and *Saccharomyces cerevisiae* Ipl1p is 41%, whereas their kinase domains share 49% identity [1]. Human and rodent ortholog pairs share 78–84% identity [2].

AKs consist of a central protein kinase domain bordered by short N- and C-terminal domains [3]. Protein kinase domains range in size from 250–300 amino acids and form a two-lobed structure (N- and C-terminal lobes). They contain twelve conserved subdomains that are separated by less highly conserved regions that serve as sites for insertions [4]. Subdomains I–IV are in the N-terminal lobe, subdomain V bridges the two lobes and forms a hinge between them, and subdomains VIA–XI are in the C-terminal lobe. Subdomain I contains a glycine-rich loop, and subdomain II contains an invariant lysine, both of which bind ATP [4,5]. Subdomain IV is important for the structure of the N-terminal lobe, subdomains VIB and VII chelate Mg^2+^, subdomain VIII faces the catalytic cleft, and subdomain IX is important for the structure of the active conformation [4,5]. Subdomains VIII, X, and XI are involved in substrate binding. AKs also possess an activation loop in the C-terminal lobe containing a threonine residue whose phosphorylation activates its kinase activity [6].

AK N- and C-termini are not as highly conserved as the kinase domain, and contain degrons, motifs that promote proteasomal-mediated degradation. Degron motifs are also present within the kinase domain [7,8,9]. AK expression levels vary within the cell cycle, and degrons induce the degradation of AKs at the end of mitosis/meiosis. Three types of degrons are present in AKs: D-boxes, A-boxes, and KEN-boxes [7]. D-boxes are present in each human AK; they bind to anaphase promoting complex/cyclosome (APC/C), resulting in proteasomal-mediated degradation of the target protein. The presence of multiple degrons is believed to enhance interaction with APC/C and therefore promote target protein degradation [7]. The N-termini of AKA and AKB, but not AKC, contain KEN and A-box degrons that may enhance AK degradation.

The expression patterns of AKs vary with the mitotic stage [10,11,12]. AKA is known as the “polar” kinase. During prophase, it is expressed in the centrosome, promoting centrosome separation and maturation. During metaphase, AKA localizes to polar microtubules and promotes spindle assembly, while in anaphase it maintains its localization to polar microtubules but also localizes to the spindle midzone. In cytokinesis, AKA is localized to the midbody. AKB is a member of the chromosome passenger complex (CPC) and is referred to as the “equatorial” kinase. AKB localizes to the centromere during prophase and metaphase, where it contributes to the spindle assembly checkpoint. It moves to the spindle midzone and the cell cortex during anaphase to promote cleavage furrow ingression. AKB then localizes to the midbody at cytokinesis. AKC is expressed at significant levels only in germ cells [9]. Data suggest that AKC plays a role in the CPC in meiosis analogous to that of AKB in mitosis.

Mutation or amplification of the three AK genes is associated with tumorigenesis. *AKA* is in a chromosomal region frequently amplified in cancer, and its mutation increases the risk of several cancers, such as esophageal, ovarian, lung, and breast cancers [12]. AKA promotes the inhibition and degradation of the tumor suppressor p53, and its overexpression can cause aneuploidy [12,13]. *AKB* is overexpressed in several cancers, including leukemia, leading to polyploidy and genomic instability [14]. *AKC* overexpression induces cell proliferation, and it is overexpressed in cancers of the reproductive tract [9].

Here, we examine the evolution of the AK gene family by employing an array of gene and protein analysis methods to provide a better understanding of the factors underlying the distinct functions of the family members. Sequences that were differentially selected in the three isoforms were identified, suggesting that they may be important for species specificity and isoform specificity, and therefore also may be targets for isoform-specific therapeutic agents.

## 2. Results and Discussion

### 2.1. Hierarchical Clustering

**Figure 1:**
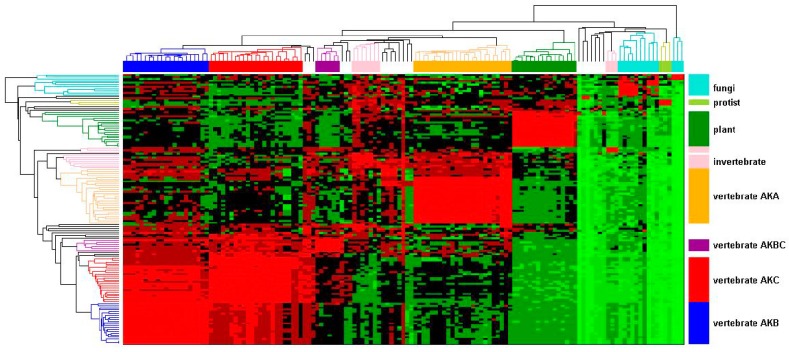
Evolutionary relationships in the AK gene family. An AK gene family hierarchical cluster was constructed based on percent identity. Each AK protein sequence was chosen in turn as query sequence in Blastp searches. The resultant pair-wise percent identities were plotted. The identity is indicated by color, ranging from the highest to lowest identity, progressively colored light red, red, maroon, black, dark green, medium green, and light green. The AK isoform designation refers to the vertebrate isoforms; other designations refers to the relevant species groups: invertebrates, protist, fungi, or plant.

A hierarchical clustering was undertaken to gain insight into the relationship among the AK genes from animal, fungal, protist, and plant species. Genes were chosen to ensure a broad representation of species rather than complete AK gene content from each species. Therefore, not all AK genes from any given species are present in the dataset. This analysis was based on sequence identity obtained through Blastp similarity searches [15]. The identity matrix was populated with percent identity values of AK proteins, where rows and columns correspond to the queries of 137 AK proteins. The identity matrix was then visualized using hierarchical clustering. The dendrograms and heat map delineate four separate AK protein clusters (Figure 1). The largest cluster consists of distinct vertebrate AKB, AKC, and AKBC subclusters. Among these subclusters, AKB and AKC shared the most identity with one another. The AKC cluster shared more identity than AKB with AKBC, suggesting that AKB experienced divergent selection following the duplication of AKBC. Conversely, there was higher identity within the AKB cluster than within the AKC cluster, suggesting that, after the initial diversifying selection, AKB experienced more stringent selection. This pattern of selection is seen among many duplicated genes [16]. An adjacent cluster consisted of AKA; the identity within this cluster was intermediate of that within the AKB or AKC clusters. The AKA cluster also had moderate identity to invertebrate AKs. The other two clusters consisted of plants and fungi. Although this data visualization clearly delineates the interrelationships amongst the broader AK gene family, it provides only an overview of the evolution of the AK gene family.

### 2.2. Phylogenetic Analysis

**Figure 2:**
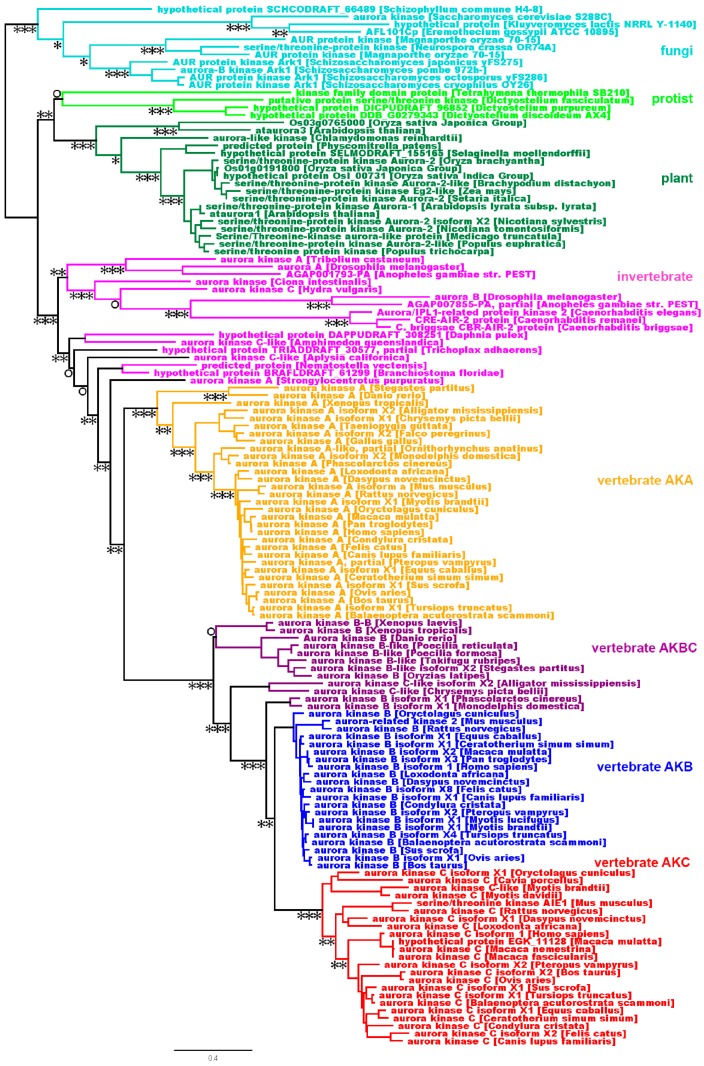
The evolution of the AK gene family. An AK phylogenetic tree was built using FatTree 2. Horizontal lines are proportional to the substitution rate. The bar represents 0.3 changes per amino acid. Local support values are marked with ***, **, * and ○ for 0.9–1.0, 0.7–0.89, 0.5–0.69 and <0.5, respectively.

To get a better understanding of the evolutionary relationship of the AK homologs, we employed a phylogenetic analysis of the same animal, fungal, protist, and plant AK genes (see sequence alignment, Figure S1). To ensure that the resultant phylogenetic tree was not biased due to low-quality alignments, we constructed trees using Gblock-filtered alignments and found that the topology of the trees made with filtered alignments was comparable to our tree [17]. A single copy of the AK gene was present prior to the formation of plant, protist, fungal, and animal clades (Figure 2). Protists and fungi maintained a single AK gene, whereas plants, which have undergone several genome-wide duplications, possess multiple genes (Figure 2 and [18,19]. Invertebrates, which include species ranging from simple animals such as sponges to invertebrate chordates, contain single or multiple AK genes, depending on the species. The invertebrate AK genes have branched into multiple clades, which are distinct from the vertebrate gene clades. Because of this, it must be kept in mind that the invertebrate *AKA* and *AKB* genes are separate groups from the vertebrate *AKA*, *AKB*, *AKC*, and *AKBC* genes.

Vertebrate AK genes separated into two clades, *AKA* and *AKBC*; the *AKBC* clade underwent further branching into *AKB* and *AKC* clades (Figure 2). The *AKA* gene, which is common to all vertebrates, is ancestral to the *AKB* and *AKC* genes. Depending on the timing of the *AKBC* duplication with regard to the speciation event, vertebrates have either two or three AK genes. An earlier analysis of the kinase domain of the AK gene family reported that cold-blooded vertebrates (frogs and fish) have two AK genes (A and B, C), whereas mammals have three (A, B, and C), with the suggestion that the *AKB* and *AKC* genes resulted from the duplication of the *AKBC* gene [20]. To determine the timing of the *AKBC* gene duplication, we examined the AK gene complement in diverse vertebrates. We first examined reptiles, including birds, to see if the *AKBC* duplication occurred prior to their separation from mammals. Two genes were identified in *Alligator mississippiensis* and *Chrysemys picta bellii* (painted turtle), *AKA* and *AKBC*, while a single AK gene, *AKA*, was identified in the birds *Falco peregrinus* and *Gallus gallus*. Sequences were available from these four genomes at 100X, 15X, 44.5X, and 106.7X whole genome coverage, respectively, suggesting that the full complement of their AK genes was present in sequence databases [21,22,23,24]. Therefore, the duplication of *AKBC* occurred after reptiles and mammals separated. Note that the bird genome has undergone much gene loss, explaining the reduction in the number of AK genes from two to one between other reptiles and birds [25]. Comparison studies between bird and reptile AK genes may lead to important functional information, as a single bird gene carries out the functions of two reptilian genes.

To more precisely determine the timing of the BC gene duplication, we examined the AK gene complement present in three mammalian groups: monotremes, marsupials, and placentals. One gene, *AKA*, was identified in the monotreme *Ornithorhynchus anatinus* (duck-billed platypus). The *Ornithorhynchus anatinus* genome has only been sequenced at 6.0X coverage, so our isolation of a single AK gene may have been due to insufficient sequencing of the platypus genome, or the absence of an *AKBC* ortholog [26]. In the marsupials *Monodelphis domestica* (opossum) and *Phascolarctos cinereus* (koala bear), two AK genes were identified, *AKA* and *AKBC*. As the koala genome has been sequenced at 57.3X coverage (and the opossum genome to 6.8X coverage), it is likely that these two genes represent the entire complement of AK genes in marsupials [27,28]. Every placental mammal that we examined possessed three AK genes. Therefore, the duplication of *AKBC* likely occurred after the branching of monotremes and marsupials from placental mammals.

*AKA* and *AKBC* genes were present more than 320 million years ago, before mammals branched from reptiles [29]. Subsequently, *AKB* and *AKC* likely arose in placental mammals by a duplication of the *AKBC* gene after placental mammals diverged from marsupials approximately 170 million years ago [30]. *AKC* experienced a higher substitution rate then *AKB* (Figure 1 and Figure 2), suggesting that *AKC* is either older than *AKB*, e.g., *AKB* arose from a secondary gene duplication followed by a gene loss, or that *AKC* has experienced less purifying selection than *AKB*. Our current data correlate well with the lineage deduced from a tree made using the AK kinase domain and lacking N- and C-termini, but adds more depth and specificity to the tree [20]. In addition, we have pinpointed the timing of the AKBC duplication to a point after monotremes and marsupials branched from placental mammals. The phylogenetic tree also shows that the exclusion of the N- and C-termini did not significantly affect the topology of the tree.

### 2.3. Selective Pressure Across the Placental Mammal Aurora Kinase Sequence

To better understand how AKA, AKB, and AKC evolved their distinct cellular functions, we analyzed the rate of nonsynonymous (*dN*) and synonymous (*dS*) substitutions in the AK gene family in placental mammals. We selected fourteen placental mammalian species for which *AKA*, *AKB*, and *AKC* genes, each containing a minimum of 95% of the gene sequence, were available. Sequences possessing high nonsynonymous, relative to synonymous, substitution rates denote regions of diversifying selection and are likely to account for the differential roles of the AK family members in the cell. *dN*/*dS* (ω) values of 1.0 signify neutrality, while values greater than 1 correspond to diversifying, and greater than 3 strong diversifying, selection. Conversely, ω values less than 1 signify purifying, and less than 0.3 strong purifying, selection.

#### 2.3.1. Whole Gene Analyses

**Figure 3:**
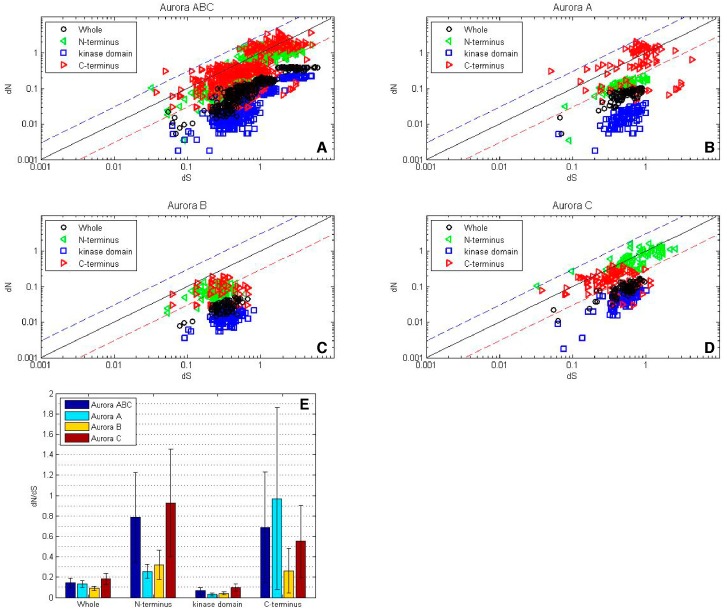
While the AK kinase domains experienced strong purifying selection, the N- and C-termini experienced either purifying or diversifying selection. A log-log plot depicting *dN*/*dS* values for the AK gene family. For each grouping, the values for the N-terminus are depicted by green triangles, the core are represented by blue squares, the C-terminus are depicted by red triangles, and the values for the entire gene are represented by black circles. The blue line corresponds to *dN*/*dS* = 1 and reflects neutrality. The dashed blue line corresponds to *dN*/*dS* = 3 and the dashed red line correspond to *dN*/*dS* = 0.3, this analysis was carried out with (**A**) the entire AK gene family, or with the individual isoform (**B**) *AKA*, (**C**) *AKB* and (**D**) *AKC*, (**E**) *dN*/*dS* values were calculated for the whole sequence as well as for the N- and C-termini and the kinase domain using all AK sequences (blue), as well as *AKA* (turquoise), *AKB* (gold) and *AKC* (maroon) sequence.

The ω values of AK genes were first examined as pair-wise comparisons in log-log plots (Figure 3A–D), and summarized as average ω values in a bar diagram (Figure 3E). Overall, the AK gene family experienced strong purifying selection, with ω values less than 1, and the vast majority under 0.3 (Figure 3A). Within the isoforms, *AKB* experienced the strongest purifying selection, followed by *AKA* and then *AKC* (Figure 3B–E). This correlates with the results of the hierarchical clustering and phylogenetic tree, in which AKB displayed the highest level of identity and the lowest number of nonsynonymous substitutions, followed by AKA and then AKC (Figure 1 and Figure 2).

#### 2.3.2. Domain Analyses

When the separate domains of the entire AK gene family were analyzed, it was found that the kinase domain experienced stronger purifying selection than the whole gene (Figure 3A). Among the individual isoforms, the kinase domain displayed strong purifying selection, with average ω values of less than 0.05 for *AKA* and *AKB*, and approximately 0.1 for *AKC*, as would be expected to maintain its catalytic activity.

Although the majority of N- and C-termini experienced purifying selection, select termini experienced strong diversifying selection (Figure 3A). *AKA* and *AKB* N-termini encoding regions experienced purifying selection, however, and approximately a third of *AKC* N-termini pair-wise comparisons showed diversifying selection (Figure 3B–D). The average ω values of the N-termini encoding regions ranged from approximately 0.25 for *AKA* and *AKB* to approximately 0.9 for *AKC*, although individual values for *AKC* were as high as 3.1 (Figure 3E).

With regard to the C-termini encoding regions, *AKB* experienced purifying to neutral selection, whereas the majority of *AKA* pair-wise comparisons yielded diversifying selection; most *AKC* pair-wise comparisons revealed purifying selection, with a small percentage showing diversifying selection. The average ω values of the C-termini encoding regions ranged from 0.25 for *AKB* to 0.55 for *AKC* and 0.97 for *AKA*, with *AKA* values as high as 5.8 (Figure 3). The diversifying selection observed with *AKA* C-termini and *AKC* N-termini suggests that these termini may interact with protein binding partners that are not highly conserved.

### 2.4. Sliding Window Analyses of Selective Pressurse on Placental Mammal Aurora Kinase

**Figure 4:**
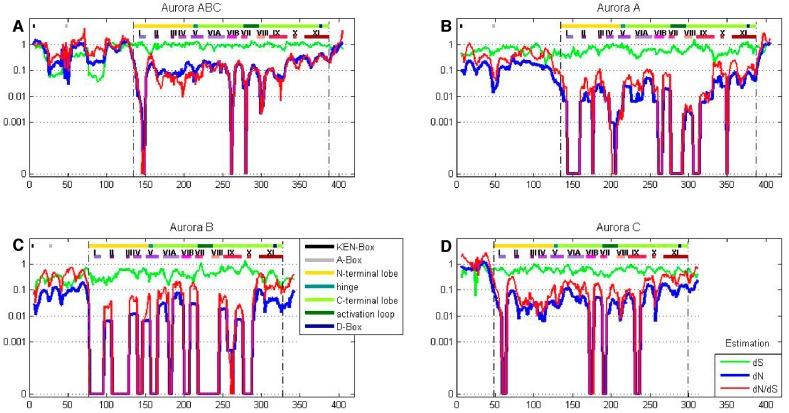
A sliding window analysis of the AK gene family exhibits the twelve kinase subdomains. A ten amino acid sliding window was employed to determine *dN* (blue), *dS* (green) and *dN*/*dS* (red) value for (**A**) the entire AK gene, (**B**) *AKA*, (**C**) *AKB*, and (**D**) *AKC*. Values were plotted at the center of each window. Dashed lines demarcate the kinase domain. The bars highlight motifs, including the N-terminal lobe; the hinge; the C-terminal lobe; the activation loop; the KEN-, A-, and D-boxes; and the twelve kinase subdomains (I–XI).

**Figure 5:**
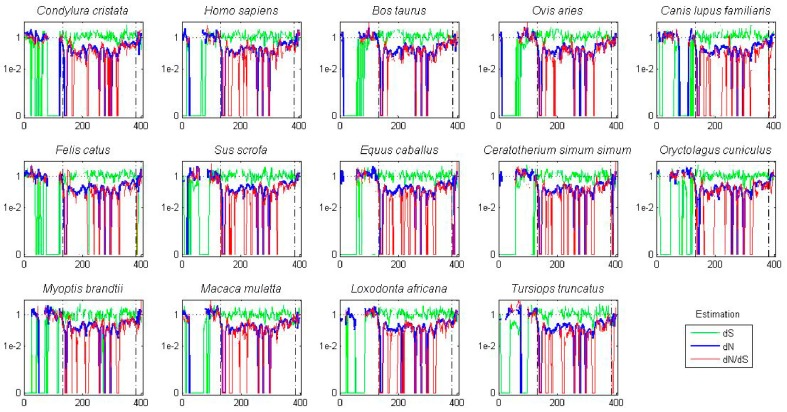
AK isoforms possess species-specific codon bias. A ten amino acid sliding window was employed to determine *dN* (blue), *dS* (green) and *dN*/*dS* (red) values for the tree AK paralogs (*AKA*, *AKB*, and *AKC*) in each of fourteen placental mammal species. Value were plotted at the center of each window. Dashed lines demarcate the kinase domain.

A sliding window analysis of *dN*, *dS*, and *dN*/*dS* values was undertaken to look more closely at the selective pressures experienced by different regions of the AK gene family. To examine both family-wide and isoform-specific selection, the entire AK gene family was analyzed together, and each isoform was examined separately (see sequence alignments, Figures S2–S5). We conducted these analyses using either a ten or thirty amino acid window size. In general, the kinase domain experienced strong purifying selection, while the N- and C-termini were less selectively constrained (Figure 4 and Figure S6). This was not unexpected, based on the functional constraints of kinase domains. In the analysis including the entire AK gene family, *dS* values were maintained near 1, except in the N-termini, in which two broad depressions with *dS* values of approximately 0.06 were observed (Figure 4A). These depressions did not correlate with two known motifs present in the N-termini, the KEN- and A-boxes. The low *dS* rate suggests that codon bias may be present, reflecting selection against synonymous substitutions. Codon bias may alter gene expression through several mechanisms, e.g., effects on cis regulatory elements, mRNA stability, and/or rates of translation [31]. This pair of N-terminal *dS* depressions was not present in the isoform-specific plots, although the *AKC* plot did possess a distinct narrow depression in *dS* (Figure 4B–D). This suggests that the reductions in *dS* were due to reduced synonymous substitutions specific for individual animal species. Therefore, an analysis of *dN*/*dS* values for AK genes within each of the fourteen species was carried out. These analyses showed that each of the fourteen species displayed reduced synonymous substitutions in their N-termini (Figure 5). This suggests that AK genes display species-specific codon bias in their N-termini. Whether this codon bias regulates AK expression will be examined in future studies.

#### 2.4.1. Aurora Kinase Subdomains—AK Family-Wide

The sliding window analysis of the entire AK gene family with a thirty amino acid window revealed two broad *dN*/*dS* depressions centered over the N- and C-terminal lobes, separated by a peak near the hinge region, suggesting that the lobes experienced strong purifying selection; this pattern was also observed in the isoform-specific analyses (Figure S6).

**Figure 6:**
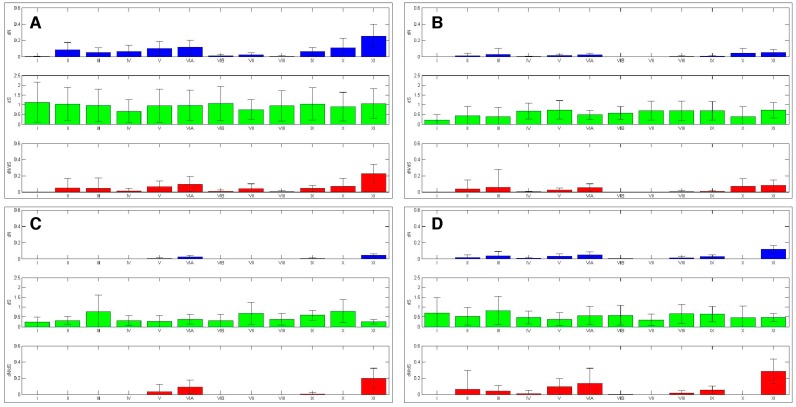
AK kinase subdomains experienced differential selection. The selection experienced by the AK subdomains was calculated. The bars represent average value, while the error bars represent plus or minus one standard deviation for *dN* (top), *dS* (middle), and *dN*/*dS* (bottom), this analysis was carried out with (**A**) the entire AK gene family, (**B**) *AKA*, (**C**) *AKB* and (**D**) *AKC*.

The twelve kinase subdomains are short sequences within the kinase domain that are more conserved than the flanking sequences [4]. These subdomains were revealed as twelve distinct depressions in the ten amino acid sliding window AK family-wide analyses, as can be seen when comparing the depressions with the protein bars at the top of each panel, as well as in the ω values calculated from the subdomains (Figure 4 and Figure 6). Examination of *dN*, *dS*, and *dN*/*dS* values revealed that subdomains I, VIB, VII, and VIII experienced the strongest purifying selection. In fact, subdomain I was completely conserved within the AK family (Figure 4A and Figure 6A). Strong purifying selection of these same subdomains was also evident in the species-specific analyses (Figure 5). These domains were especially prominent when examining *dN* values. Our results complement a previous report that found that subdomains VIB, VIII, and IX were the most conserved subdomains in the kinase superfamily [4]. However, our analyses revealed that subdomains I and VII also experienced strong purifying selection in the AK gene family (Figure 4A and Figure 6A). In addition, although subdomain IX was one of the three most conserved subdomains in the kinase superfamily, the purifying selection it experienced in the AK gene family was approximately an order of magnitude weaker than the subdomains under the strongest purifying selection. Each of these subdomains plays a critical role in kinase function. Subdomain I, which was invariant, encodes for a glycine-rich loop that binds ATP; subdomain VIB is required for catalytic activity, subdomain VII chelates Mg^2+^, and subdomain VIII faces the catalytic cleft [4,5]. The strong purifying selection of subdomains I and VII in AKs suggests that they may play a more prominent role in the catalytic cleft of AKs than in other kinases. Therefore, similar but distinct selective forces may have acted upon AKs versus the kinase superfamily as a whole, i.e., subdomains I and VII may play primary, whereas subdomain IX may play secondary, role(s) in AKs.

#### 2.4.2. Aurora Kinase Subdomains—AK Isoforms

We next examined the *dN*/*dS* plots of each isoform to determine if any of them experienced distinct selective pressures that may have led to their unique cellular functions. The *dN*/*dS* plots revealed that each AK isoform experienced unique patterns of purifying selection (Figure 4B–D and Figure 6B–D). The *dN*/*dS* values for *AKA* and *AKB* were reduced as compared to those of *AKABC*, as would be expected if *AKA* and *AKB* experienced distinct constraints on their cellular functions. However, the *dN*/*dS* values for *AKC* closely mirrored those of *AKABC* in magnitude. A generalized reduction in purifying selection of *AKC* may have been necessary for sequence variations in *AKC* that conferred meiosis-specific functions upon it.

With *AKA*, the subdomains that experienced the strongest purifying selection coincided with those identified in the AK family-wide analysis; subdomains I, VIB, and VII had no nonsynonymous mutations, while subdomain VIII’s ω value was 0.003. AKB experienced the strongest purifying selection of all of the isoforms in its kinase subdomains, with eight of the twelve subdomains displaying no nonsynonymous substitutions (I–IV, VIB–VIII, and X). This correlates with the hierarchical analysis which also suggests that after an initial period of diversification, *AKB* became the most constrained isoform.

Subdomain X experienced strong purifying selection in *AKB* and *AKC*, as no nonsynonymous substitutions were present within each isoform (Figure 4 and Figure 6). However, there was nominal differential selection between *AKB* and *AKC*, as reflected by an ω value of 0.05 in their pair-wise analysis (Figure S7 and see alignment, Figure S8). In comparison, subdomain X was one of the least strongly selected subdomains within *AKA*, and was distinct from the selection experienced by *AKB* and *AKC*, as the ω values were 0.257 or 0.164 between *AKA* and *AKB* or *AKC*, respectively (Figure S7 and see alignment, Figures S9 and S10). Subdomain X binds substrate proteins, and the low number of nonsynonymous substitutions between *AKB* and *AKC* suggests that *AKB* and *AKC* bind the same or related substrates, whereas *AKA* binds distinct substrate(s). Subdomain XI displayed weak purifying selection in and between *AKB* and *AKC*, possessing ω values more than two-fold higher than any other subdomain, whereas subdomain XI’s ω value was similar to other weakly selected subdomains in *AKA* (Figures 4, 6 and S7). The role of subdomain XI in binding substrates suggests that its weak purifying selection may promote the binding of distinct substrates to AK isoforms.

**Figure 7:**
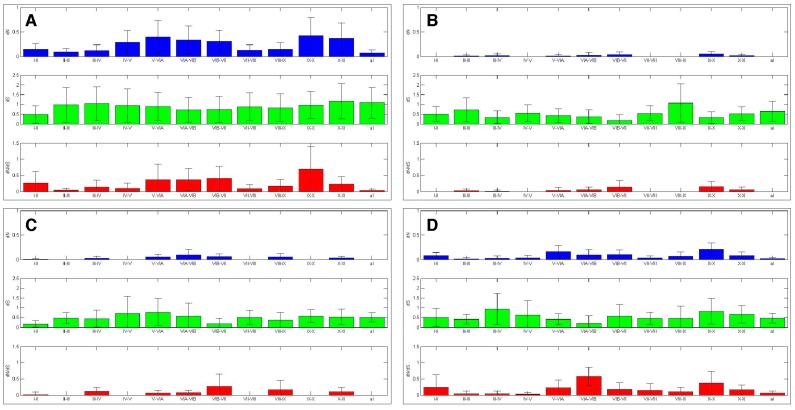
AK kinase interdomains experienced differential selection. The selection experienced by the AK interdomains and activation loop was calculated. The bars represent average value, while the error bars represent plus or minus one standard deviation for *dN* (top), *dS* (middle), and *dN*/*dS* (bottom), this analysis was carried out with (**A**) the entire AK gene family, (**B**) *AKA*, (**C**) *AKB* and (**D**) *AKC*.

The AK activation loop begins in subdomain VII and ends in subdomain VIII; therefore, the strong purifying selection of these subdomains, as well as the intervening sequence, is likely due to the presence of the activation loop. *AKA* and *AKB* both experienced strong purifying selection of the activation loop, exhibiting no nonsynonymous substitutions, whereas *AKC*’s purifying selection of the activation loop was not quite as strong, perhaps due to its divergence in acquiring its role in meiosis (Figure 7). In pair-wise comparisons between the different isoforms, average ω values ranged from 0.037 to 0.080; therefore, the activation domain experienced strong, but not identical, selection in each isoform (Figure S7).

### 2.5. Sliding Window Analyses of Selective Pressures Outside of Aurora Kinase Subdomains

The ten amino acid sliding window analysis of the AK isoforms also uncovered purifying selection in the sequences that flank the subdomains, the “interdomains” (Figure 4). When ω values were calculated, it was found that the interdomains experienced varying levels of purifying selection (Figure 7). This finding correlates with the characterization of the interdomains as sites of insertions or other sequence variations that define kinase families [4]. Conversely, several interdomains experienced weak diversifying selection, suggesting that these domains may be important in defining AK isoform specificity.

The *dN*/*dS* analysis of *AKC* interdomains was relatively similar to that of the AK family-wide analysis (Figure 7). The *dN*/*dS* analyses of *AKA* and *AKB* revealed that several interdomains experienced strong purifying selection. Interdomains IV–V and VII–VIII lacked nonsynonymous substitutions in both *AKA* and *AKB*, whereas interdomains I–II and VIII–IX lacked nonsynonymous substitutions only in *AKA*, and interdomains II–III and IX–X lacked nonsynonymous substitutions only in *AKB*. Each of these interdomains was distinct between *AKA* and *AKB* (Figure S7). In fact, interdomain IX–X displayed ω values of up to 2.34 in the *AKA*/*AKB* analysis (Figure S7). Interdomains V–VIA and VIB–VII also experienced diversifying selection between *AKA* and *AKB*, with ω values of up to 2.62 and 2.19, respectively. Interdomains V–VIA and IX–X experienced diversifying selection between *AKA* and *AKC*, with ω values of up to 2.71 and 3.26, respectively (Figure S7). This suggests that the AK interdomains V–VIA, VIB–VII, and IX–X may have a role in defining AK isoform specificity. Further experimentation will be necessary to determine the contribution that these interdomains make to AK isoform specificity.

## 3. Materials and Methods

### 3.1. Identification of Human Aurora Kinase Gene Homologs

The three human AK genes were used to identify AK homologs. The amino acid sequences of each human AK (AKA, AKB, and AKC) were used as queries to search the NCBI non-redundant protein sequences (nr) database in a Blastp search [15]. Using the default values for the algorithm parameters, a total of approximately 10,000 target sequences resulted from each human Aurora kinase query; thus, a total of 30,000 sequences were collected, including duplicates between searches. The resulting sequences were filtered by the following criteria: percent identity ≥ 40, percent query coverage ≥ 50, bit-score ≥ 250, and *E*-value ≤ 10^−3^, giving 2683 sequences for AKA, 2701 for AKB, and 2677 for AKC. Multiple data entries were then converged to a single entry, and only a single strain of each species was kept. Any fusion vector sequences were also removed. The filtered lists were further refined to select the highest-scoring target sequences from each query, which included fully-sequenced model organisms. The resulting sequences from each query were then combined and duplicate sequences were removed, resulting in a total of 137 non-redundant sequences spanning the major eukaryotic taxonomic groups.

### 3.2. Hierarchical Clustering

We employed Hierarchical Agglomerative Clustering (HAC) to visualize the relationships among the AK homologs as described previously [32]. First, all the pair-wise amino acid identities were computed using Blastp for every possible pair of the 137 homologous genes chosen above, resulting in a protein percent identity matrix. HAC clustered sequences first along the columns of the percent identity matrix based on Euclidian distance between rows, producing the row-clustered data. HAC then clustered sequences along the rows, resulting in column-clustered data. Therefore, two dendrograms were generated, one for the row-clustered data and one for the column-clustered data. A heat map was then generated with reordered rows and columns of the identity matrix based on the row and column clusters. Each row of this matrix was transformed with z-score transformation to have a mean of 0 and a standard deviation of 1 for better visualization. To be more specific, each amino acid sequence, the identity between pairs of amino acid sequences, and Unweighted Pair Group Method with Arithmetic Mean (UPGMA) was used as a data point, a pair-wise similarity, and an intergroup similarity measure, respectively, for HAC [33]. The heat map and accompanying dendograms for the 137 homologs were generated using the clustergram function of the Bioinformatics Toolbox of MATLAB 7.11 (R2010b) (MathWorks, Natick, MA, USA) [34].

### 3.3. Phylogenetic Analysis

A phylogenetic analysis using the maximum-likelihood method was utilized to construct a phylogenetic tree using the Geneious 9.0 platform, a suit of molecular biology tools [35]. First, amino acid sequences were aligned using MUltiple Sequence Comparison by Log-Expectation (MUSCLE) with default option values [36,37]. Then, the resulting alignment was input to FastTree version 2.1.5, an open-source approximately maximum-likelihood algorithm for generating phylogenies over large alignments, with default option values. FastTree is faster than, and as accurate as, other maximum-likelihood methods such as RAxML and PhyML [38,39,40]. The tree was rooted with fungi. Trees with similar topology were obtained when rooting with either plants or protists (data not shown). Trees with similar topology were also constructed using Gblocks-trimmed alignments; this ensured that the unfiltered alignments were of high quality (available online: http://phylogeny.lirmm.fr/phylo_cgi/one_task.cgi?task_type=gblocks).

### 3.4. dN/dS Calculation

Using the results of hierarchical clustering and phylogenetic analyses, AK homologs were assigned into one of three groups: *AKA*, *AKB*, or *AKC*. These groups were further studied to determine the selective constraints operating on the genes using the ratio of the nonsynonymous substitution rate (*dN*) over the synonymous substitution rate (*dS*) for two aligned sequences [41]. The amino acid sequences in each group were first aligned with MUSCLE to prevent the introduction of frame-shifts due to the incorrect placement of gaps during alignments [42]. Our sequences were aligned using a progressive multiple sequence alignment method, *multialign* function, implemented in the Bioinformatics Toolbox in MATLAB 7.11 (R2010B) with default values (MathWorks, Natick, MA, USA) [32]. Secondly, the aligned sequences were used as a guide to insert the appropriate gaps into the original nucleotide sequences. Then, the *dN*/*dS* ratio for every possible pair of the multiple-aligned, gap-inserted nucleotide sequences was estimated using the *dnds* function of the Bioinformatics Toolbox in MATLAB 7.11 (R2010B) with default option values (MathWorks, Natick, MA, USA) [34]. Then, using a sliding window of ten or thirty codons, the *dN*/*dS* ratio value for each window over the entire aligned length was calculated as was done previously [43]. The resulting averaged *dN*, *dS*, and *dN*/*dS* values were plotted at the center of each window.

Furthermore, to estimate the selective pressure experienced in AK domains, the multiple-aligned sequences were split using the multialign function according to the structure of human AK. These domains included the N-terminus, the kinase domain, the C-terminus, the activation loop, the twelve kinase subdomains, and the eleven kinase interdomains.

## 4. Conclusions

Our data suggest that *AKA* is the ancestral gene in vertebrates, and that the duplication of *AKBC* in placental mammals led to the presence of three AK genes. The N-termini encoding domains of *AKC* and the C-termini encoding domains of *AKA* experienced diversifying selection, perhaps due to their interaction with protein binding partners that experienced diversifying selection. *AKC* experienced a generalized reduction in purifying selection, which may have facilitated the acquisition of meiosis-specific functions. AK genes displayed species-specific reductions in synonymous substitutions in their N-termini, indicating that codon bias may play a role in the regulation of AK expression. The data suggest that selective pressure experienced by kinase subdomains and interdomains were important in determining specificity within the AK family, as well as between kinase families. Each AK gene is deregulated in one or more types of human cancer, and further studies identifying residues that experienced purifying or diversifying selective pressure may aid in the combined bioinformatic and cheminformatic design of pan or isoform-specific aurora kinase inhibitors, respectively.

## Supplementary Materials

Supplementary materials can be found at www.mdpi.com/1422-0067/19/1/72/s1.

## Abbreviations

APC/C	anaphase promoting complex/cyclosome
AK	aurora kinase
CPC	chromosome passenger complex
HAC	Hierarchical Agglomerative Clustering
MUSCLE	MUltiple Sequence Comparison by Log-Expectation
nr	non-redundant
UPGMA	Unweighted Pair Group Method with Arithmetic Mean
