# Supplementary material for: Differential Selective Pressures Experienced by the Aurora Kinase Gene Family
Source: Int J Mol Sci. 2017 Dec 28;19(1):72. doi: 10.3390/ijms19010072 (PMC5796022; doi:10.3390/ijms19010072)
Supplement: Supplementary file 1 [file ijms-19-00072-s001.zip › FigureS8AKBCalignwLegend.pdf]

Figure S8. Alignment of placental mammal AKB and AKC sequences. The alignment of the placental mammal AKB and AKC sequences used for the dN/dS analyses.

```
MATLAB multiple sequence alignment


lcl|NM_004217.3 MAQKENSYPWPYGRQTAPSGLSTLPQRVLRKEPVTPSALVLMSRSNVQPTAAPGQ-KVME  59
lcl|XM_01511855 MAQKENAYPWPYGRQTAPSGLSTLPQRVLRKEPVTPSALVLMSRSNVQPTAAPGQ-KVVE  59
lcl|NM_183084.2 MAQKENAYPWPYGRQTAQPGLNTLPQRVLRKEPVTPSALVLMSRSNAQPTAAPGQ-KVVE  59
lcl|XM_00401268 MAQKENAYPWPYGRQTAQPGLNTLPQRVLRKEAVIPSALVLMSRSNAQPTAAPGQ-KVVE  59
lcl|XM_844813.4 MTQKENAYPWPYGRQTTQPGLNTLPQRVLRKDPATPSALVLMSRSNGQPTAAPGQ-KVVE  59
lcl|XM_00399619 MAQKENAYPWPYGRQTTQPGLNTLPQRVLRKEPATPSALVLMSRSNTQPTAAPGQ-KVVE  59
lcl|NM_213919.2 MAQKENTYPWPYGRQTAQSGLNILPQRVLRKEAVTPSALVLMSRSNTQPTAAPGQ-KVVE  59
lcl|XM_00150481 MTQKENAYPWPYGRQTAQSGLNTLPQRVLRKEPATPSALVLMSRSNAQPTVALGQ-KVME  59
lcl|XM_00443309 MAQKENAYPWPYGRQTAQSGLNTLPQRVLRKEPVTPSALVLMSRSNAQPTAALGQ-KVVE  59
lcl|XM_00827074 MAQKENAYPWPYSRPTPQSGLNTLPQRILRKEAATPSALVLLNRSNAPPTAVLGQ-KGME  59
lcl|XM_00587878 MTQKENAYPWPYGRQTAQCGLNTLPQRVLQKDPTTPSSLVLMSRSNAQPTAAPGQ-----  55
lcl|XM_00468461 MAQKENAYPWPYGRQTAQSGLNTLPQRVLRKDPTTPSALVLMNRSNTQPTAAPGQ-KVVE  59
lcl|XM_00341691 MAQKENAYPWPYGRQTAQSGLNTLPQRVFRKDLATPSALVLMSRSNAQPTAAPGEKKMTE  60
lcl|XM_01994424 MAQKENAYPWPYGRQTAQSGLNTLPQRVLRKEPVTPSALVLMSRSNAQPTAAPLQ-KVVE  59
lcl|NM_00101587 MSSPRAVVQLGKA----------------------------QPAGEELATANQTA-----  27
lcl|XM_00109836 MSSPKAAVQLGRA----------------------------QPAGEAVTAANQTA-----  27
lcl|XM_00521979 MSHRGTARKAGG----------------------------------MQHAAAAGQ-----  21
lcl|XM_00401544 MSHRGTARKAGG----------------------------------MQPAAAAGQ-----  21
lcl|XM_00561607 MLSGSAQPASE------------------------------GEKTQEQSKDAAGH-----  25
lcl|XM_00694080 MQSGSVQPAAEE-----------------------------EEQTRDQGTAAAGL-----  26
lcl|XM_02109730 MKSGSVQPAVA----------------------------------------AAGQ-----  15
lcl|XM_00150199 MSLPEAVMKSG---------------------------------SVRPAVPTAGQ-----  22
lcl|XM_00443911 MSLPRAVMKLG---------------------------------DVQSAGETAGQ-----  22
lcl|XM_00824896 MQS---------------------------------------------GSSQAAG-----  10
lcl|XM_01453723 MNPGFLLSIIAE--------------------------------------KKEPW-----  17
lcl|XM_01273470 MKS-----------------------------------------GDAQAAGAAGG-----  14
lcl|XM_00340664 MFTNVALSFARR-------------------------------EWKELAPTERAL-----  24
lcl|XM_01992887 MKSGSAQPAAV-----------------------------------------AGQ-----  14
                *

lcl|NM_004217.3 NSSGTPDILTRHFTIDDFEIGRPLGKGKFGNVYLAREKKSHFIVALKVLFKSQIEKEGVE 119
lcl|XM_01511855 NSSGTPNILMRHFTIDDFEIGRPLGKGKFGNVYLAREKKSHFIVALKVLFKSQIEKEGVE 119
lcl|NM_183084.2 NSSGTPNIPKRSFTIDDFEIGRPLGKGKFGNVYLAREKKSHFIVALKVLFKSQIEKEGVE 119
lcl|XM_00401268 NSSGTLNIPKRSFTIDDFEIGRPLGKGKFGNVYLAREKKSHFIVALKVLFKSQIEKEGVE 119
lcl|XM_844813.4 NSSGIPNFSMRSFTIDDFEIGRPLGKGKFGNVYLAREKKSHFIVALKVLFKSQIEKEGVE 119
lcl|XM_00399619 NSSGTPNFSMRSFTIDDFEIGRPLGKGKFGNVYLAREKKSHFIVALKVLFKSQIEKEGVE 119
lcl|NM_213919.2 NSSGTPNFSTRSFTIDDFEIGRPLGKGKFGNVYLAREKKSHFIVALKVLFKSQIEKEGVE 119
lcl|XM_00150481 NSSGTPNLLMRSFTIDDFEIGRPLGKGKFGNVYLAREKKSHFIVALKVLFKSQIEKEGVE 119
lcl|XM_00443309 NSSGTPNLLMRSFTIDDFEIGRPLGKGKFGNVYLAREKKSHFIVALKVLFKSQIEKEGVE 119
lcl|XM_00827074 NSSGAPNF-TRPFTIDDFEIGRPLGKGKFGNVYLAREKKSHFIVALKVLFKSQIEKEGVE 118
lcl|XM_00587878 NSNGTPN-LMRSFTIDDFEIGRPLGKGKFGNVYLAREKKSRFIVALKVLFKSQIEKEGVE 114
lcl|XM_00468461 NSSGTPNLSMRSFTIDDFEIGRPLGKGKFGNVYLAREKKSHFIVALKVLFKSQIEKEGVE 119
lcl|XM_00341691 NSSGTPSFLMRPFTIDDFEIGRPLGKGKFGNVYLAREKKSHFIVALKVLFKSQIEKEGVE 120
lcl|XM_01994424 NNSRTPNFSMRSFTIDDFEIGRPLGKGKFGNVYLAREKKSHFIVALKVLFKSQIEKEGVE 119
lcl|NM_00101587 --QQPSSPAMRRLTVDDFEIGRPLGKGKFGNVYLARLKESHFIVALKVLFKSQIEKEGLE  85
lcl|XM_00109836 --QQPSSPAMRRLKIDDFEIGRPLGKGKFGNVYLARLKESHFIVALKVLFKSQIEKEGLE  85
lcl|XM_00521979 --TVPGAPTARRFTVDDFEIGRPLGKGKFGNVYLARLKKNHFIVALKVLFKSQIEKEGLE  79
lcl|XM_00401544 --SVPGAPAARRFTIDDFEIGRPLGKGKFGNVYLARLKKNHFIVALKVLFKSQIEKEGLE  79
lcl|XM_00561607 --VELSVPPGRRLTIDDFEIGRPLGKGKFGNVYLARLKESHFIVALKVLFKSQIEKEGLE  83
lcl|XM_00694080 --VELGMPPVRRLTIEDFEIGRPLGKGKFGNVYLARLKESHFLVALKVIFKSQIEKEGLE  84
lcl|XM_02109730 --TVSNIPTGRRLTIDDFEIGRPLGKGKFGNVYLARLKESHFIVALKVLFKSQIEKEGLE  73
lcl|XM_00150199 --SQPGFPNVRHLTIDDFEIGRPLGKGKFGNVYLARLKESHFIVALKVLFKSQIEKEGLE  80
lcl|XM_00443911 --TQPSFPTVRHFTIDDFEIGRPLGKGKFGNVYLARLKESHFIVALKVLFKSQIEKEGLE  80
lcl|XM_00824896 --PAQPSVPLRRLTIEDFEIGRPLGKGKFGNVYLARLKDSHFIVALKVLFKSQIEKEGME  68
lcl|XM_01453723 --PSSPSPGSRSLTIDDFDIRCPLGKGKFGNVYLARLRDSHFIVALKVLFKSQLEKEGME  75
lcl|XM_01273470 --PALPVATVRRLTIDDFEIGRPLGKGKFGNVYLARLRESRFLVALKVLFKSQIEKEGLE  72
lcl|XM_00340664 --YGDVLLEMRRLRIDDFEIGRPLGKGKFGNVYLARLQENHFLVALKVLFKSQIEKEGLE  82
lcl|XM_01992887 --TMPSIPTVRRLTIDDFEIGRPLGKGKFGNVYLARLKESHFIVALKVLFKSQIEKEGLE  72
                          * : ::**:*  ************** :..:*:*****:****:****:*

lcl|NM_004217.3 HQLRREIEIQAHLHHPNILRLYNYFYDRRRIYLILEYAPRGELYKELQKSCTFDEQRTAT 179
lcl|XM_01511855 HQLRREIEIQAHLHHPNILRLYNYFYDRRRIYLILEYAPRGELYKELQKSRTFDEQRTAT 179
lcl|NM_183084.2 HQLRREIEIQAHLQHPNILRLYNYFYDRRRIYLILEYAPRGELYKELQKSRTFDEQRTAT 179
lcl|XM_00401268 HQLRREIEIQAHLQHPNILRLYNYFYDRRRIYLILEYAPRGELYKELQKSRTFDEQRTAT 179
lcl|XM_844813.4 HQLRREIEIQAHLQHPNILRLYNYFYDRRRIYLILEYAPRGELYKELQKSHTFDEQRTAT 179
lcl|XM_00399619 HQLRREIEIQAHLQHPNILRLYNYFYDRRRIYLILEYAPRGELYKELQKSRTFDEQRTAT 179
lcl|NM_213919.2 HQLRREIEIQAHLQHPNILRLYNYFYDRRRIYLILEYAPRGELYKELQKCRTFDEQRTAT 179
lcl|XM_00150481 HQLRREIEIQAHLQHPNILRLYNYFYDRRRIYLILEYAPRGELYKELQKSRTFDEQRTAT 179
lcl|XM_00443309 HQLRREIEIQAHLQHPNILRLYNYFYDRRRIYLILEYAPRGELYKELQKSRTFDEQRTAT 179
```

```
lcl|XM_00827074 HQLRREIEIQAHLQHPNILRLYNYFYDRRRIYLILEYAPRGELYKELQKSRTFDEQRTAT 178
lcl|XM_00587878 HQLRREIEIQAHLQHPNILRLYNYFYDRRRIYLILEYAPRGELYKELQKSRTFDQQRTAT 174
lcl|XM_00468461 HQLRREIEIQAHLQHPNILRLYNYFYDRRRIYLILEYAPRGELYKELQKSRTFDEQRTAT 179
lcl|XM_00341691 HQLRREIEIQAHLQHPNILRLYNYFYDRRRVYLILEYAPRGELYKELQKSRTFDEQRTAT 180
lcl|XM_01994424 HQLRREIEIQAHLQHPNILRLYNYFYDRRRIYLILEYAPRGELYKELQKSHTFDEQRTAT 179
lcl|NM_00101587 HQLRREIEIQAHLQHPNILRLYNYFHDARRVYLILEYAPRGELYKELQKSEKLDEQRTAT 145
lcl|XM_00109836 HQLRREIEIQAHLQHPNILRLYNYFHDARRVYLILEFAPRGELYKELQKSEKLDEQRAAT 145
lcl|XM_00521979 HQLRREVEIQAHLQHPNILRLYNYFHDARRVYLILEYAPKGELYKALQRSHTFDEQRTAT 139
lcl|XM_00401544 HQLRREVEIQAHLQHPNILRLYNYFHDARRVYLILEYAPKGELYKALQRSHTFDEQRTAT 139
lcl|XM_00561607 HQLRREIEIQAHLQHPNILRLYNYFHDARRVYLILEYAPRGELYKELQKSHTLDEQHTAT 143
lcl|XM_00694080 HQLRREIEIQAHLQHPNILRLYNYFHDARRVYLILEYAPRGELYKELQKSNTLDEQHTAT 144
lcl|XM_02109730 HQLRREIEIQAHLQHPNILRLYNYFHDARRVYLILEYAPRGELYKELQKSHTLDEQRTAT 133
lcl|XM_00150199 HQLRREIEIQAHLQHPNILRLYNYFHDARRVYLILEYAPRGELYKELQKSHTLDEQRTAT 140
lcl|XM_00443911 HQLRREIEIQAHLQHPNILRLYNYFHDARRVYLILEYAPRGELYKELQKSHTLDEQRTAT 140
lcl|XM_00824896 HQLRREIEIQAHLQHPNILRLYNYFHDARRIYLILEYAPRGELYKELQKCHTLDEQRTAT 128
lcl|XM_01453723 HQLRREIEIQSHLQHPNILRLHNYFHDERXVYLILEYAPRGELYKELQKSHTIDEQSTAT 135
lcl|XM_01273470 HQLRREVEIQAHLRHPNILRLYNYFHDARRVYLILEYAPRGELYKELQNCHTLDEQRTAT 132
lcl|XM_00340664 HQLRREIEIQAHLQHPNILRLYNYFHDSRRVYLILEYAPRGELYKELQKSHRLDEQRTAT 142
lcl|XM_01992887 HQLRREIEIQAHLQHPNILRLYNYFHDARRMYLILEYAPRGELYKELQKSHTLDEQRTAT 132
                ******:***:**:*******:***:* * :*****:**:***** **..  :*:* :**

lcl|NM_004217.3 IMEELADALMYCHGKKVIHRDIKPENLLLGLKGELKIADFGWSVHAPSLRRKTMCGTLDY 239
lcl|XM_01511855 IMEELADALMYCHGKKVIHRDIKPENLLLGLKGELKIADFGWSVHAPSLRRKTMCGTLDY 239
lcl|NM_183084.2 IMEELADALTYCHAKKVIHRDIKPENLLLGLRGELKIADFGWSVHAPSLRRKTMCGTLDY 239
lcl|XM_00401268 IMEELADALTYCHAKKVIHRDIKPENLLLGLRGELKIADFGWSVHAPSLRRKTMCGTLDY 239
lcl|XM_844813.4 IMEELADALMYCHGKKVIHRDIKPENLLLGLQGELKIADFGWSVHAPSLRRKTMCGTLDY 239
lcl|XM_00399619 IMEELADALLYCHGKKVIHRDIKPENLLLGLQGELKIADFGWSVHAPSLRRKTMCGTLDY 239
lcl|NM_213919.2 IMEELADALIYCHGKKVIHRDIKPENLLLGLQGELKIADFGWSVHAPSLRRKTMCGTLDY 239
lcl|XM_00150481 IMEELADALMYCHGKKVIHRDIKPENLLLGLQGELKIADFGWSVHAPSLRRKTMCGTLDY 239
lcl|XM_00443309 IMEELADALIYCHGKKVIHRDIKPENLLLGLQGELKIADFGWSVHAPSLRRKTMCGTLDY 239
lcl|XM_00827074 IMEELADALRYCHEKKVIHRDIKPENLLLGLQGELKIADFGWSVHAPSLRRKTMCGTLDY 238
lcl|XM_00587878 IMEELADALMYCHKKKVIHRDIKPENLLLGLQGELKIADFGWSVHAPSLRRKTMCGTLDY 234
lcl|XM_00468461 IMEELADALIYCHGKKVIHRDIKPENLLLGLQGELKIADFGWSVHAPSLRRKTMCGTLDY 239
lcl|XM_00341691 IMEELADALMYCHGKKVIHRDIKPENLLLGLRGELKIADFGWSVHAPSLRRKTMCGTLDY 240
lcl|XM_01994424 IMEELADALIYCHGKKVIHRDIKPENLLLGLQGELKIADFGWSVHAPSLRRKTMCGTLDY 239
lcl|NM_00101587 IIEELADALTYCHDKKVIHRDIKPENLLLGFRGEVKIADFGWSVHTPSLRRKTMCGTLDY 205
lcl|XM_00109836 IIEELADALTYCHEKKVIHRDIKPENLLLGFRGEVKIADFGWSVHTPSLRRKTMCGTLDY 205
lcl|XM_00521979 IIEELADALIYCHERKVIHRDIKPENLLLGLMGEVKIADFGWSVHTPSLRRRTTCGTLDY 199
lcl|XM_00401544 IIEELADALIYCHEKKVIHRDIKPENLLLGLMGEVKIADFGWSVHTPSLRRRTTCGTLDY 199
lcl|XM_00561607 IMEELADALTYCHEKKVIHRDIKPENLLLGFKGEVKIADFGWSVHTPSLRRKTMCGTLDY 203
lcl|XM_00694080 IMEELADALTYCHEKKVIHRDIKPENLLLGFRGEVKIADFGWSVHTPSLRRKTMCGTLDY 204
lcl|XM_02109730 IMEELADALTYCHEKKVIHRDIKPENLLLGFRGEVKIADFGWSVHTPSLRRKTMCGTLDY 193
lcl|XM_00150199 IMEELADALTYCHEKKVIHRDIKPENLLLGFRGEVKIADFGWSVHTPSLRRKTMCGTLDY 200
lcl|XM_00443911 IMEELADALTYCHEKKVIHRDIKPENLLLGFRGEVKIADFGWSVHTPSLRRKTMCGTLDY 200
lcl|XM_00824896 IMEELADALTYCHGKKVIHRDIKPENLLLGFRGEVKIADFGWSVHTLSLRRKTMCGTLDY 188
lcl|XM_01453723 IMEELAHALAYCHENKVIHRDIKPENLLLGLRGEVKIADFGWSVHTPSLRRKTMCGTLDY 195
lcl|XM_01273470 IIEELADALTYCHEKKVIHRDIKPENLLLGYRGEVKIADFGWSVHTPSLRRKTMCGTLDY 192
lcl|XM_00340664 IMEELADALIYCHEKKVIHRDIKPENLLLGLRGEVKIADFGWSVHTLSLRRKTMCGTLDY 202
lcl|XM_01992887 VMEELADALTYCHEKKVIHRDIKPENLLLGFRGEVKIADFGWSVHTPSLRRRTMCGTLDY 192
                ::****.** *** .***************  **:**********: ****:* ******

lcl|NM_004217.3 LPPEMIEGRMHNEKVDLWCIGVLCYELLVGNPPFESASHNETYRRIVKVDLKFPASVPMG 299
lcl|XM_01511855 LPPEMIEGRTHNEKVDLWCIGVLCYELLVGNPPFESASHNETYRRIVKVDLKFPASVPTG 299
lcl|NM_183084.2 LPPEMIEGRTHNEKVDLWCIGVLCYELLVGNPPFESASHNETYRRIVKVDLKFPPSVPLG 299
lcl|XM_00401268 LPPEMIEGRTHNEKVDLWCIGVLCYELLVGNPPFESASHNETYRRIVKVDLKFPPSVPMG 299
lcl|XM_844813.4 LPPEMIEGRMHNEKVDLWCIGVLCYELLVGNPPFESASHNETYRRIVKVDLKFPPSVPTG 299
lcl|XM_00399619 LPPEMIEGRTHNEKVDLWCIGVLCYELLVGNPPFESASHNETYRRIVKVDLKFPPSVPTG 299
lcl|NM_213919.2 LPPEMIEGRTHNEKVDLWCIGVLCYELLVGNPPFESASHNETYRRIVKVDLKFPPSVPAG 299
lcl|XM_00150481 LPPEMIEGRTHNEKVDLWCIGVLCYELLVGNPPFESASHNETYRRIVKVDLKFPPSVPAG 299
lcl|XM_00443309 LPPEMIEGRTHNEKVDLWCIGVLCYELLVGNPPFESASHNETYRRIVKVDLKFPASVPAG 299
lcl|XM_00827074 LPPEMIEGRTHNEKVDLWCIGVLCYELLVGNPPFESASHNETYRRIVKVDLKFPASVPAG 298
lcl|XM_00587878 LPPEMIEGRTHNEKVDLWCIGVLCYELLVGNPPFESASHNETYRRIVKVDLKFPPSVPVG 294
lcl|XM_00468461 LPPEMIEGRTHNEKVDLWCIGVLCYELLVGSPPFESASHNETYRRIVKVDLKFPLSVPSG 299
lcl|XM_00341691 LPPEMIEGRMHNEKVDLWCIGVLCYELLVGNPPFESASHNETYRRIVKVDLKFPPSVPTG 300
lcl|XM_01994424 LPPEMIEGRTHNEKVDLWCIGVLCYELLVGNPPFESASHNETYRRIVKVDLKIPPSMPAG 299
lcl|NM_00101587 LPPEMIEGRTYDEKVDLWCIGVLCYELLVGYPPFESASHSETYRRILKVDVRFPLSMPLG 265
lcl|XM_00109836 LPPEMIEGRTYDEKVDLWCIGVLCYELLVGYPPFESTSHSETYRRILKVDVRFPLSMPLG 265
lcl|XM_00521979 LPPEMIEGRTYDEKVDLWCIGVLCYELLVGNPPFESASTSETYRRILKVDLRFPPSMSSG 259
lcl|XM_00401544 LPPEMIEGRTYDEKVDLWCIGVLCYELLVGNPPFESASTSETYRRILKVDLRFPPSMSSG 259
lcl|XM_00561607 LPPEMIERRTYNEKVDLWCIGVLCYELLVGYPPFESPSHNETYRRILKVDLRFPPSLPLG 263
lcl|XM_00694080 LPPEMIERRTYNETVDLWCIGVLCYELLVGNPPFESHSHNETYRRILKVDIRFPPSIPLG 264
lcl|XM_02109730 LPPEMIEGRTYNEKVDLWCIGVLCYELLVGNPPFESASHNETYRRILKVDVRFPPSLPLG 253
```

```
lcl|XM_00150199 LPPEMIEGRTYNEKVDLWCIGVLCYELLVGNPPFESNSHNETYRRILKVDVRFPPSIPSG 260
lcl|XM_00443911 LPPEMIEGRTYNEKVDLWCIGVLCYELLVGNPPFESNSHNETYRRILKVDVRFPPSIPLG 260
lcl|XM_00824896 LPPEMIEGRTYDEKVDLWCIGVLCYELLVGNPPFESVSHTETYRRILKVDVKFPVTMSLG 248
lcl|XM_01453723 LPPEMVEQRTYSEMVDLWCIGVLCYELLVGNPPFKSSSYSETYRRILKVDVKFPPSIPLG 255
lcl|XM_01273470 LPPEMIEGRTYNEKVDLWCIGVLCYELLVGKPPFESSTHNETYRRILKVDVRFPPSVPLG 252
lcl|XM_00340664 LPPEMIEGRTYDERVDLWCIGVLCYELLVGNPPFESPSHTETYRRILKVDVRFPSSMPSG 262
lcl|XM_01992887 LPPEMIEGRTYNEKVDLWCIGVLCYELLVGNPPFESPSHNETYRRILQVDVRFPPSMPLG 252
                *****:* * :.* **************** ***:* : .******::**:::* ::. *

lcl|NM_004217.3 AQDLISKLLRHNPSERLPLAQVSAHPWVRANSRRVLPPSALQSVA* 345
lcl|XM_01511855 AQDLISKLLRHNPSERLPLAQVSAHPWVRANSRRVLPPSALQSVA* 345
lcl|NM_183084.2 AQDLIYKLLKHNPSERLPLAQVSAHPWVRTHSRRVLPPSAPQSVP* 345
lcl|XM_00401268 AQDLISKLLKHNPSERLPLAQVSAHPWVRAHSRRVLPPSAPQSVP* 345
lcl|XM_844813.4 AQDLISKLLKHNPSERLPLSQVSAHPWVRAHSRRMLPPSAVQAIP* 345
lcl|XM_00399619 AQDLISKLLKHNPSERLPLAQVSAHPWVRAHSRRVLPPSALQSVP* 345
lcl|NM_213919.2 AQDLISKLLKHNPSDRLPLAQVSAHPWVRAHSRRVLPPSAPQSVP* 345
lcl|XM_00150481 AQDLISKLLRHNPSERLPLAQVSAHPWVRAHSRRVLPPSALQSVP* 345
lcl|XM_00443309 AQDLISKLLKHNPSERLPLAQVSAHPWVRAHSRRVLPPSAPQSVP* 345
lcl|XM_00827074 AQDLISKLLRHNPSDRLTLAQVSAHPWVRAHSRRVLPPSAMQSVA* 344
lcl|XM_00587878 AQDLISKLLKHNPSDRLPLAQVSAHPWVRAHSRRVLPPSALQSVH* 340
lcl|XM_00468461 AQDLITKLLKHNPSERLPLTQVSAHPWVRAHSRRVLPPSALQSVP* 345
lcl|XM_00341691 AQDLISKLLKHNPSERLPLAQVSAHPWVRAHSRRVLPPSALQSVP* 346
lcl|XM_01994424 AQDLISKLLKHNPSERLPLAQVAAHPWVRAHSRRVLPPSALQSVP* 345
lcl|NM_00101587 ARDLISRLLRYQPLERLPLAQILKHPWVQAHSRRVLPPCAQMAS*- 310
lcl|XM_00109836 ARDLISKLLRYQPLERLPLAQILKHPWVQVHSRRVLPPCAQMAS*- 310
lcl|XM_00521979 ARDLISKLLRFQPLERLPLVRVLEHPWIRAHSQRVLPPSVPMAF*- 304
lcl|XM_00401544 ARDLISKLLRFQPLERLPLVRVLEHPWIRAHSQRVLPPSVQRAF*- 304
lcl|XM_00561607 AQDLVSKLLRYQPLERLPLAQIMEHPWVRAHSRRVLPPSVQMAS*- 308
lcl|XM_00694080 ARDLISKLLRYQPLERLPLSQILEHPWVRAHSRRVVPPSVQMDS*- 309
lcl|XM_02109730 AQDLISRLLRYQPSDRLPLDQILEHPWVRAHSRRVLPPSTQMVS*- 298
lcl|XM_00150199 AKDLISKLLRYQPLERLPLDQILEHPWVRAHSRRVLPPSAQMAS*- 305
lcl|XM_00443911 AKDLISKLLRYQPLERLPLDQIMEHPWVRAHSRRVLPPSAQMAS*- 305
lcl|XM_00824896 ARDLISRLLRYQPSERLPLAQILRHPWVQAHSRRVLPPCAHPAS*- 293
lcl|XM_01453723 AQDLISKLLRYQPSERLLLAQVLQHPWVQAHSRRVGPPPAPSAS*- 300
lcl|XM_01273470 AQDLISSLLRYQPSERLPLAQILQHPWVRMHSRRVLPPSAQVAS*- 297
lcl|XM_00340664 AQDLISKLLRYQPFERLALAQVLEHPWVQAHSQRVLPPSAQMAS*- 307
lcl|XM_01992887 AQDLISKLLRYQPSERLPLVQILEHPWVRAHSRRVLPPSAQMVS*- 297
                *:**:  **:.:* :** * ::  ***:: :*:*: ** .
```
