# Supplementary material for: Differential Selective Pressures Experienced by the Aurora Kinase Gene Family
Source: Int J Mol Sci. 2017 Dec 28;19(1):72. doi: 10.3390/ijms19010072 (PMC5796022; doi:10.3390/ijms19010072)
Supplement: Supplementary file 1 [file ijms-19-00072-s001.zip › FigureS9AKABalignwLegend.pdf]

Figure S9. Alignment of placental mammal AKA and AKB sequences. The alignment of the placental mammal AKA and AKB sequences used for the dN/dS analyses.

```
MATLAB multiple sequence alignment


lcl|NM_198436.2 MDRSKENCISGPVKATAPVG-GPKRVLVTQQFPCQNPLPVNSGQAQRVLCPSNSSQRIPL  59
lcl|XM_01514873 MDRSKENCISGPVKPAAPVG-GPKRVLVTQQFPCQNPLPANSGQAQRVLCPSNSSQRVPL  59
lcl|NM_00103802 MDRCKENCISGP-KTAVPLSDGPKRVPVAQQFPSQNPVSVNSGQAQRVLCPTNSSQRVPS  59
lcl|NM_00114250 MDRCKENCISGP-KTAVPLSDGPKRVPVAQQFPSQNPVSVNSGQAQRVLCPTNSSQRVPS  59
lcl|XM_00563519 MDKSKENCIAGPVKTAIALGDGPKRVLVTQQVPSQNPLSANSGQAQRVLCPSNSSQRVPP  60
lcl|XM_00398335 MDKSKENCVAGPVKTTIPLGDGPKRVPVTQQFPSQNLLSANSGQAQRVLCPSNSSQRIPS  60
lcl|XM_00567303 MDKCKENCISGL-KTTVPPGDGPKRVPVTQHFPAQHLPSANSGQAQRVLCPSNSSQRLPS  59
lcl|XM_00148927 MDRSKENCISGPVKTTVPLGDGPKRVPVTQQLP-QNLLSANSGQAQRVLCPSNSSHRVPS  59
lcl|XM_00443024 MDRSKENCVAGPVKTTIPLGDGPKRVLVTQQFPSQHPLSANSGQAQRVLCPSNSSHRAPT  60
lcl|XM_00827414 MERSKENCISGPIKAAALLGDGPKRVPVTQRFPSQNPVSACSGQAERVLCPSNSSQRVPS  60
lcl|XM_01452869 MDKSKENGISGLVKTTIPSGDGPKRVLVTQQFPSQYPLPANSGQAQRVLCPSNSSQQVPS  60
lcl|XM_00468690 MDKSKENCISGPVKTMVPLRDGAKRVPVHQHAPSQSALPASSGQAQRVLCPSN-SQRLPS  59
lcl|XM_00341990 MDKSKENCISGPIKTTLPLGDGPKRVPVTQPFPSQNPLSASGGQAQRVLCPSNSSQSIPS  60
lcl|XM_00432103 MDKCKENSISGP-KTTVPLGDGPKRVPVTQQFPSQNPVSANSGQAQRVLCPSNSSQRVPS  59
lcl|NM_004217.3 MAQKENS--------------------------YPWPYGRQTAPSGLSTLPQRVLRKEPV  34
lcl|XM_01511855 MAQKENA--------------------------YPWPYGRQTAPSGLSTLPQRVLRKEPV  34
lcl|NM_183084.2 MAQKENA--------------------------YPWPYGRQTAQPGLNTLPQRVLRKEPV  34
lcl|XM_00401268 MAQKENA--------------------------YPWPYGRQTAQPGLNTLPQRVLRKEAV  34
lcl|XM_844813.4 MTQKENA--------------------------YPWPYGRQTTQPGLNTLPQRVLRKDPA  34
lcl|XM_00399619 MAQKENA--------------------------YPWPYGRQTTQPGLNTLPQRVLRKEPA  34
lcl|NM_213919.2 MAQKENT--------------------------YPWPYGRQTAQSGLNILPQRVLRKEAV  34
lcl|XM_00150481 MTQKENA--------------------------YPWPYGRQTAQSGLNTLPQRVLRKEPA  34
lcl|XM_00443309 MAQKENA--------------------------YPWPYGRQTAQSGLNTLPQRVLRKEPV  34
lcl|XM_00827074 MAQKENA--------------------------YPWPYSRPTPQSGLNTLPQRILRKEAA  34
lcl|XM_00587878 MTQKENA--------------------------YPWPYGRQTAQCGLNTLPQRVLQKDPT  34
lcl|XM_00468461 MAQKENA--------------------------YPWPYGRQTAQSGLNTLPQRVLRKDPT  34
lcl|XM_00341691 MAQKENA--------------------------YPWPYGRQTAQSGLNTLPQRVFRKDLA  34
lcl|XM_01994424 MAQKENA--------------------------YPWPYGRQTAQSGLNTLPQRVLRKEPV  34
                * : ::                                            * .  :

lcl|NM_198436.2 QAQKLVSSHK--PVQNQKQKQLQATSVPHPVSRPLNNTQKSKQPLPSAPENNPEEELASK 117
lcl|XM_01514873 QAQKLVSSHK--LVQNQKQKQLQATSVPHPVSRPPSHTPKSKQPLPSAPENNPEEELASK 117
lcl|NM_00103802 QAQKLVSIQK--PVQTLKQKPPQAASAPRPVTRPPSNTQKSKQPQPPAPGNNPEKEVASK 117
lcl|NM_00114250 QAQKLVSIQK--PVQTLKQKPPQAASAPRPVARPPSNTQKSKQPQPPAPGNNPEKEVASK 117
lcl|XM_00563519 QTQKLVSSHK--PAQNLKQKQLQATGVPRPASRSLNNTQKSEQPSSSAPGNNSEKELATK 118
lcl|XM_00398335 QTQKLVSSHK--PVQNLKQKQLQATSVPRSVSRPLNNTQKSEQPLPSATGNNSEKELTTK 118
lcl|XM_00567303 HTQKLVSSHK--PVQNLKQKQSQATSGPRPVSRPLSNTQQSEQPQPAAPGNNPEKEAASK 117
lcl|XM_00148927 QAQKLVSSHK--PVQNLKQKQSQATSVPHPVPRPLNNTQRSEQLQPSAPGNNSEKELASK 117
lcl|XM_00443024 QAQKLVSSHR--PVQNLKQKQSQATSVPPPVSRPLNSTQKSEQPQPSAPGNNSEKERTSK 118
lcl|XM_00827414 QAQKLLPAEK----PVQKLKQAQAASVPRPVSKPPNNTPKSEQPPPSAPGNNPEKELTSN 116
lcl|XM_01452869 KAQKLVSSHK--PVLNLKQKQLQATSGPCPVSRPLNNTQKSEQPQPPAPGKNSEKEVASK 118
lcl|XM_00468690 QAQKLVSSHKAVPNPNLKQKQLQANNAPRAAPRPLAGTPKSEPP-PSAPGNNSEKEQASK 118
lcl|XM_00341990 QAQKLVSSRK--PVQSQKQKPLQITTVPRPVSRPPNNTPKGEQAQPPASGNNSGKELASK 118
lcl|XM_00432103 QVQKLVSSQK--SVQPLKQKPTQAASAPRSVSRPLSNTQKSEQPQLPAPGNNPEKEAASK 117
lcl|NM_004217.3 TPSALVLMSR-----------------------------SNVQPTAAPGQ---KVMENS  61
lcl|XM_01511855 TPSALVLMSR-----------------------------SNVQPTAAPGQ---KVVENS  61
lcl|NM_183084.2 TPSALVLMSR-----------------------------SNAQPTAAPGQ---KVVENS  61
lcl|XM_00401268 IPSALVLMSR-----------------------------SNAQPTAAPGQ---KVVENS  61
lcl|XM_844813.4 TPSALVLMSR-----------------------------SNGQPTAAPGQ---KVVENS  61
lcl|XM_00399619 TPSALVLMSR-----------------------------SNTQPTAAPGQ---KVVENS  61
lcl|NM_213919.2 TPSALVLMSR-----------------------------SNTQPTAAPGQ---KVVENS  61
lcl|XM_00150481 TPSALVLMSR-----------------------------SNAQPTVALGQ---KVMENS  61
lcl|XM_00443309 TPSALVLMSR-----------------------------SNAQPTAALGQ---KVVENS  61
lcl|XM_00827074 TPSALVLLNR-----------------------------SNAPPTAVLGQ---KGMENS  61
lcl|XM_00587878 TPSSLVLMSR-----------------------------SNAQPTAAPGQ-------NS  57
lcl|XM_00468461 TPSALVLMNR-----------------------------SNTQPTAAPGQ---KVVENS  61
lcl|XM_00341691 TPSALVLMSR-----------------------------SNAQPTAAPGE--KKMTENS  62
lcl|XM_01994424 TPSALVLMSR-----------------------------SNAQPTAAPLQ---KVVENN  61
                  . *:   :                              .:     .  :       ..

lcl|NM_198436.2 QKNEESKKRQWALEDFEIGRPLGKGKFGNVYLAREKQSKFILALKVLFKAQLEKAGVEHQ 177
lcl|XM_01514873 QKNEESKKRQWALEDFEIGRPLGKGKFGNVYLAREKQSKFILALKVLFKAQLEKAGVEHQ 177
lcl|NM_00103802 QKNEESKKRQWALEDFEIGRPLGKGKFGNVYLAREKQSKFILALKVLFKAQLEKAGVEHQ 177
lcl|NM_00114250 QKNEESKKRQWALEDFEIGRPLGKGKFGNVYLAREKQSKFILALKVLFKAQLEKAGVEHQ 177
lcl|XM_00563519 QKNEESKKRQWALEDFEIGRPLGKGKFGNVYLAREKQSKFILAIKVLFKAQLEKAGVEHQ 178
lcl|XM_00398335 QKNEESKKRQWALEDFEIGRPLGKGKFGNVYLAREKQSKFILALKVLFKAQLEKAGVEHQ 178
lcl|XM_00567303 QKNEESKKRQWALEDFEIGRPLGKGKFGNVYLAREKQSKFILALKVLFKTQLEKAGVEHQ 177
```

```
lcl|XM_00148927 EKNEESKKRQWALEDFEIGRPLGKGKFGNVYLAREKQSKFILALKVLFKAQLEKAGVEHQ 177
lcl|XM_00443024 EKNEDSKRRQWALEDFEIGRPLGKGKFGNVYLAREKQSKFILALKVLFKAQLEKAGVEHQ 178
lcl|XM_00827414 QKNEESKKRQWTLEDFDIGRPLGKGKFGNVYLAREKQSKFILALKVLFKAQLEKAGVEHQ 176
lcl|XM_01452869 QKNEESKK-QWTLEDFEIGRPLGKGKFGNVYLAREKQSKFILALKVLFKAQLEKAGVEHQ 177
lcl|XM_00468690 QKTEESKKRQWALEDFEIGRPLGKGKFGNVYLAREKQSKFILALKVLFKAQLEKAGVEHQ 178
lcl|XM_00341990 QKKEESKRRQWALEDFEIGRPLGKGKFGNVYLAREKQSKFILALKVLFKAQLEKAGVEHQ 178
lcl|XM_00432103 QKNEESKRRQWALEDFEIGRPLGKGKFGNVYLAREKQSKFILALKVLFKAQLEKAGVEHQ 177
lcl|NM_004217.3 SGTPDILTRHFTIDDFEIGRPLGKGKFGNVYLAREKKSHFIVALKVLFKSQIEKEGVEHQ 121
lcl|XM_01511855 SGTPNILMRHFTIDDFEIGRPLGKGKFGNVYLAREKKSHFIVALKVLFKSQIEKEGVEHQ 121
lcl|NM_183084.2 SGTPNIPKRSFTIDDFEIGRPLGKGKFGNVYLAREKKSHFIVALKVLFKSQIEKEGVEHQ 121
lcl|XM_00401268 SGTLNIPKRSFTIDDFEIGRPLGKGKFGNVYLAREKKSHFIVALKVLFKSQIEKEGVEHQ 121
lcl|XM_844813.4 SGIPNFSMRSFTIDDFEIGRPLGKGKFGNVYLAREKKSHFIVALKVLFKSQIEKEGVEHQ 121
lcl|XM_00399619 SGTPNFSMRSFTIDDFEIGRPLGKGKFGNVYLAREKKSHFIVALKVLFKSQIEKEGVEHQ 121
lcl|NM_213919.2 SGTPNFSTRSFTIDDFEIGRPLGKGKFGNVYLAREKKSHFIVALKVLFKSQIEKEGVEHQ 121
lcl|XM_00150481 SGTPNLLMRSFTIDDFEIGRPLGKGKFGNVYLAREKKSHFIVALKVLFKSQIEKEGVEHQ 121
lcl|XM_00443309 SGTPNLLMRSFTIDDFEIGRPLGKGKFGNVYLAREKKSHFIVALKVLFKSQIEKEGVEHQ 121
lcl|XM_00827074 SGAPNF-TRPFTIDDFEIGRPLGKGKFGNVYLAREKKSHFIVALKVLFKSQIEKEGVEHQ 120
lcl|XM_00587878 NGTPN-LMRSFTIDDFEIGRPLGKGKFGNVYLAREKKSRFIVALKVLFKSQIEKEGVEHQ 116
lcl|XM_00468461 SGTPNLSMRSFTIDDFEIGRPLGKGKFGNVYLAREKKSHFIVALKVLFKSQIEKEGVEHQ 121
lcl|XM_00341691 SGTPSFLMRPFTIDDFEIGRPLGKGKFGNVYLAREKKSHFIVALKVLFKSQIEKEGVEHQ 122
lcl|XM_01994424 SRTPNFSMRSFTIDDFEIGRPLGKGKFGNVYLAREKKSHFIVALKVLFKSQIEKEGVEHQ 121
                .   .     ::::**:*******************:*:**:*:*****:*:** *****

lcl|NM_198436.2 LRREVEIQSHLRHPNILRLYGYFHDATRVYLILEYAPLGTVYRELQKLSKFDEQRTAT-Y 236
lcl|XM_01514873 LRREVEIQSHLRHPNILRLYGYFHDATRVYLILEYAPLGTVYRELQKLSKFDEQRTAT-Y 236
lcl|NM_00103802 LRREVEIQSHLRHPNILRLYGYFHDATRVYLILEYAPLGAVYRELQKLSKFDEQRTAT-Y 236
lcl|NM_00114250 LRREIKIQSHLKHPNILRLYGYFHDATRVYLILEYAPLGAVYRELQKLSKFDEQRTAT-Y 236
lcl|XM_00563519 LRREVEIQSHLRHPNILRLYGYFHDATRVYLILEYAPLGAVYRELQKLSKFDEQRTAT-Y 237
lcl|XM_00398335 LRREVEIQSHLRHPNILRLYGYFHDATRVYLILEYAPLGAVYRELQKLSKFDEQRTAT-Y 237
lcl|XM_00567303 LRREVEIQSHLRHPNILRLYGYFHDATRVYLILEYAPLGAVYRELQKLSKFDEQRTAT-Y 236
lcl|XM_00148927 LRREVEIQSHLRHPNILRLYGYFHDATRVYLILEYAPLGAVYRELQKLSKFDEQRTAT-Y 236
lcl|XM_00443024 LRREVEIQSHLRHPNILRLYGYFHDATRVYLILEYAPLGAVYRELQKLSKFDEQRTAT-Y 237
lcl|XM_00827414 LRREVEIQSHLRHPNILRLYGYFHDATRVYLILEYAPLGTVYRELQKLSKFDEQRTAT-Y 235
lcl|XM_01452869 IRREVEIQSHLRHPNILRLYGYFHDATRVYLILEYAPLGTVYRELQKLSRFDEQRTAT-Y 236
lcl|XM_00468690 LRREVEIQSHLRHPNILRLYGYFHDATRVYLILEYAPLGAVYRELQKLSKFDEQRTAT-Y 237
lcl|XM_00341990 VRREVEIQSHLRHPNILRLYGYFHDATRVYLILEYAPLGTVYRELQKXSKFDEQRTAP*Y 238
lcl|XM_00432103 LRREVEIQSHLRHPNILRLYGYFHDATRVYLILEYAPLGAVYRELQKLSKFDEQRTAT-Y 236
lcl|NM_004217.3 LRREIEIQAHLHHPNILRLYNYFYDRRRIYLILEYAPRGELYKELQKSCTFDEQRTAT-I 180
lcl|XM_01511855 LRREIEIQAHLHHPNILRLYNYFYDRRRIYLILEYAPRGELYKELQKSRTFDEQRTAT-I 180
lcl|NM_183084.2 LRREIEIQAHLQHPNILRLYNYFYDRRRIYLILEYAPRGELYKELQKSRTFDEQRTAT-I 180
lcl|XM_00401268 LRREIEIQAHLQHPNILRLYNYFYDRRRIYLILEYAPRGELYKELQKSRTFDEQRTAT-I 180
lcl|XM_844813.4 LRREIEIQAHLQHPNILRLYNYFYDRRRIYLILEYAPRGELYKELQKSHTFDEQRTAT-I 180
lcl|XM_00399619 LRREIEIQAHLQHPNILRLYNYFYDRRRIYLILEYAPRGELYKELQKSRTFDEQRTAT-I 180
lcl|NM_213919.2 LRREIEIQAHLQHPNILRLYNYFYDRRRIYLILEYAPRGELYKELQKCRTFDEQRTAT-I 180
lcl|XM_00150481 LRREIEIQAHLQHPNILRLYNYFYDRRRIYLILEYAPRGELYKELQKSRTFDEQRTAT-I 180
lcl|XM_00443309 LRREIEIQAHLQHPNILRLYNYFYDRRRIYLILEYAPRGELYKELQKSRTFDEQRTAT-I 180
lcl|XM_00827074 LRREIEIQAHLQHPNILRLYNYFYDRRRIYLILEYAPRGELYKELQKSRTFDEQRTAT-I 179
lcl|XM_00587878 LRREIEIQAHLQHPNILRLYNYFYDRRRIYLILEYAPRGELYKELQKSRTFDQQRTAT-I 175
lcl|XM_00468461 LRREIEIQAHLQHPNILRLYNYFYDRRRIYLILEYAPRGELYKELQKSRTFDEQRTAT-I 180
lcl|XM_00341691 LRREIEIQAHLQHPNILRLYNYFYDRRRVYLILEYAPRGELYKELQKSRTFDEQRTAT-I 181
lcl|XM_01994424 LRREIEIQAHLQHPNILRLYNYFYDRRRIYLILEYAPRGELYKELQKSHTFDEQRTAT-I 180
                :***::**:**:********.**:*  *:******** * :*:****   **:****.

lcl|NM_198436.2 ITELANALSYCHSKRVIHRDIKPENLLLGSAGELKIADFGWSVHAPSSRRTTLCGTLDYL 296
lcl|XM_01514873 ITELANALSYCHSKRVIHRDIKPENLLLGSAGELKIADFGWSVHAPSSRRTTLCGTLDYL 296
lcl|NM_00103802 ITELANALSYCHSKRVIHRDIKPENLLLGSAGELKIADFGWSVHAPSSRRTTLCGTLDYL 296
lcl|NM_00114250 ITELANALSYCHSKRVIHRDIKPENLLLGSAGELKIADFGWSVHAPSSRRTTLCGTLDYL 296
lcl|XM_00563519 ITELADALSYCHSKRVIHRDIKPENLLLGSAGELKIADFGWSVHAPSSRRTTLCGTLDYL 297
lcl|XM_00398335 ITELADALSYCHSKRVIHRDIKPENLLLGSAGELKIADFGWSVHAPSSRRTTLCGTLDYL 297
lcl|XM_00567303 ITELANALSYCHSKRVIHRDIKPENLLLGSAGELKIADFGWSVHAPSSRRTTLCGTLDYL 296
lcl|XM_00148927 ITELANALAYCHSKRVIHRDIKPENLLLGSAGELKIADFGWSVHAPSSRRTTLCGTLDYL 296
lcl|XM_00443024 ITELANALAYCHSKRVIHRDIKPENLLLGSAGELKIADFGWSVHAPSSRRTTLCGTLDYL 297
lcl|XM_00827414 ITELANALLYCHSKRVIHRDIKPENLLLGSAGELKIADFGWSVHAPSSRRTTLCGTLDYL 295
lcl|XM_01452869 ITELTNALSYCHSKRVIHRDIKPENLLLGSTGELKIADFGWSVHAPSSRRTTLCGTLDYL 296
lcl|XM_00468690 ITELADALSYCHSKRVIHRDIKPENLLLGSAGELKIADFGWSVHAPSSRRTTLCGTLDYL 297
lcl|XM_00341990 ITELADALSYCHSKKVIHRDIKPENLLLGSTGELKIADFGWSVHAPSSRRTTLCGTLDYL 298
lcl|XM_00432103 ITELANALSYCHSKRVIHRDIKPENLLLGSSGELKIADFGWSVHAPSSRRTTLCGTLDYL 296
lcl|NM_004217.3 MEELADALMYCHGKKVIHRDIKPENLLLGLKGELKIADFGWSVHAPSLRRKTMCGTLDYL 240
lcl|XM_01511855 MEELADALMYCHGKKVIHRDIKPENLLLGLKGELKIADFGWSVHAPSLRRKTMCGTLDYL 240
lcl|NM_183084.2 MEELADALTYCHAKKVIHRDIKPENLLLGLRGELKIADFGWSVHAPSLRRKTMCGTLDYL 240
```

```
lcl|XM_00401268 MEELADALTYCHAKKVIHRDIKPENLLLGLRGELKIADFGWSVHAPSLRRKTMCGTLDYL 240
lcl|XM_844813.4 MEELADALMYCHGKKVIHRDIKPENLLLGLQGELKIADFGWSVHAPSLRRKTMCGTLDYL 240
lcl|XM_00399619 MEELADALLYCHGKKVIHRDIKPENLLLGLQGELKIADFGWSVHAPSLRRKTMCGTLDYL 240
lcl|NM_213919.2 MEELADALIYCHGKKVIHRDIKPENLLLGLQGELKIADFGWSVHAPSLRRKTMCGTLDYL 240
lcl|XM_00150481 MEELADALMYCHGKKVIHRDIKPENLLLGLQGELKIADFGWSVHAPSLRRKTMCGTLDYL 240
lcl|XM_00443309 MEELADALIYCHGKKVIHRDIKPENLLLGLQGELKIADFGWSVHAPSLRRKTMCGTLDYL 240
lcl|XM_00827074 MEELADALRYCHEKKVIHRDIKPENLLLGLQGELKIADFGWSVHAPSLRRKTMCGTLDYL 239
lcl|XM_00587878 MEELADALMYCHKKKVIHRDIKPENLLLGLQGELKIADFGWSVHAPSLRRKTMCGTLDYL 235
lcl|XM_00468461 MEELADALIYCHGKKVIHRDIKPENLLLGLQGELKIADFGWSVHAPSLRRKTMCGTLDYL 240
lcl|XM_00341691 MEELADALMYCHGKKVIHRDIKPENLLLGLRGELKIADFGWSVHAPSLRRKTMCGTLDYL 241
lcl|XM_01994424 MEELADALIYCHGKKVIHRDIKPENLLLGLQGELKIADFGWSVHAPSLRRKTMCGTLDYL 240
                : **::** *** *:**************  **************** **.*:*******

lcl|NM_198436.2 PPEMIEGRMHDEKVDLWSLGVLCYEFLVGKPPFEANTYQETYKRISRVEFTFPDFVTEGA 356
lcl|XM_01514873 PPEMIEGRMHDEKVDLWSLGVLCYEFLVGKPPFEANTYQETYKRISRVEFTFPDFVTEGA 356
lcl|NM_00103802 PPEMIEGRMHDEKVDLWSLGVLCYEFLVGKPPFEADTYQETYRRISRVEFTFPDCVPEGA 356
lcl|NM_00114250 PPEMIEGRMHDEKVDLWSLGVLCYEFLVGKPPFEADTYQETYRRISRVEFTFPDCVPEGA 356
lcl|XM_00563519 PPEMIEGRMHDEKVDLWSLGVLCYEFLVGKPPFEASTYQETYKRISRVEFTFPDFVPEGA 357
lcl|XM_00398335 PPEMIEGRMHDEKVDLWSLGVLCYEFLVGKPPFEASTYQETYKRISRVEFTFPDFVPEGA 357
lcl|XM_00567303 PPEMIEGRMHDEKVDLWSLGVLCYEFLVGKPPFEANTYQETYKRISRVEFTFPDFVPEGA 356
lcl|XM_00148927 PPEMIEGRMHDEKVDLWSLGVLCYEFLVGKPPFEASTYQETYKRISRVEFTFPDFVPEGA 356
lcl|XM_00443024 PPEMIEGRMHDEKVDLWSLGVLCYEFLVGKPPFEASTYQETYKRISRVEFTFPDFVPDGA 357
lcl|XM_00827414 PPEMIEGRMHDEKVDLWSLGVLCYEFLVGKPPFEANTYQETYRRISRVEFTFPDFVTEGA 355
lcl|XM_01452869 PPEMIEGRMHDEKVDLWSLGVLCYEFLVGKPPFEASTYQETYKRISRVEFTFPDFVREGA 356
lcl|XM_00468690 PPEMIEGRMHDEKVDLWSLGVLCYEFLVGKPPFEASTYQETYKRISRVEFTFPDFVPEGA 357
lcl|XM_00341990 PPEMIEGRMHDEKVDLWSLGVLCYEFLVGKPPFEASTYQETYNRISRVEFTFPDLVTVGA 358
lcl|XM_00432103 PPEMIEGRMHDEKVDLWSLGVLCYEFLVGKPPFEANTYQETYKRISRVEFTFPDFVPEGA 356
lcl|NM_004217.3 PPEMIEGRMHNEKVDLWCIGVLCYELLVGNPPFESASHNETYRRIVKVDLKFPASVPMGA 300
lcl|XM_01511855 PPEMIEGRTHNEKVDLWCIGVLCYELLVGNPPFESASHNETYRRIVKVDLKFPASVPTGA 300
lcl|NM_183084.2 PPEMIEGRTHNEKVDLWCIGVLCYELLVGNPPFESASHNETYRRIVKVDLKFPPSVPLGA 300
lcl|XM_00401268 PPEMIEGRTHNEKVDLWCIGVLCYELLVGNPPFESASHNETYRRIVKVDLKFPPSVPMGA 300
lcl|XM_844813.4 PPEMIEGRMHNEKVDLWCIGVLCYELLVGNPPFESASHNETYRRIVKVDLKFPPSVPTGA 300
lcl|XM_00399619 PPEMIEGRTHNEKVDLWCIGVLCYELLVGNPPFESASHNETYRRIVKVDLKFPPSVPTGA 300
lcl|NM_213919.2 PPEMIEGRTHNEKVDLWCIGVLCYELLVGNPPFESASHNETYRRIVKVDLKFPPSVPAGA 300
lcl|XM_00150481 PPEMIEGRTHNEKVDLWCIGVLCYELLVGNPPFESASHNETYRRIVKVDLKFPPSVPAGA 300
lcl|XM_00443309 PPEMIEGRTHNEKVDLWCIGVLCYELLVGNPPFESASHNETYRRIVKVDLKFPASVPAGA 300
lcl|XM_00827074 PPEMIEGRTHNEKVDLWCIGVLCYELLVGNPPFESASHNETYRRIVKVDLKFPASVPAGA 299
lcl|XM_00587878 PPEMIEGRTHNEKVDLWCIGVLCYELLVGNPPFESASHNETYRRIVKVDLKFPPSVPVGA 295
lcl|XM_00468461 PPEMIEGRTHNEKVDLWCIGVLCYELLVGSPPFESASHNETYRRIVKVDLKFPLSVPSGA 300
lcl|XM_00341691 PPEMIEGRMHNEKVDLWCIGVLCYELLVGNPPFESASHNETYRRIVKVDLKFPPSVPTGA 301
lcl|XM_01994424 PPEMIEGRTHNEKVDLWCIGVLCYELLVGNPPFESASHNETYRRIVKVDLKIPPSMPAGA 300
                ******** *:******.:******:***.****: :::***.** :*::.:*  :  **

lcl|NM_198436.2 RDLISRLLKHNPSQRPMLREVLEHPWITANSSKPSNCQNKESASKQS*- 404
lcl|XM_01514873 RDLISRLLKHNPSQRPMLREVLEHPWITANSSKPSNCQNRVN*------ 399
lcl|NM_00103802 RDLISRLLKHNPSQRPTLKEVLEHPWIIANSKPSSCQKKESTSKQS*-- 403
lcl|NM_00114250 RDLISRLLKHNPSQRPTLKEVLEHPWITANSKPSSCQKKESTSKQS*-- 403
lcl|XM_00563519 RDLISRLLKHNPSQRPTLKDVLEHPWIMANSSKPSSSQKNKDSTSKQS* 406
lcl|XM_00398335 RDLISRLLKHNPSQRPTLKEVLEHPWVTANSSKPSGSQKSKESTGKQS* 406
lcl|XM_00567303 RDLISRLLKHNPSHRPTLKEVLEHPWITANSKPASSHKKESTSKQP*-- 403
lcl|XM_00148927 RDLISRLLKHNPSQRPTLKEVLEHPWITANSSKPSNSQKSKESTSKQP* 405
lcl|XM_00443024 RDLISRLLKHNPSQRPTLKEVLEHPWITANASKPSNSQKSKESTSKQS* 406
lcl|XM_00827414 KDFISRLLKHNPSHRPTLAEVLEHPWVKAKSSKPSSCPDKEPASKQS*- 403
lcl|XM_01452869 RDLISRLLMHNPNQRLTLKEVLEHPWITANSSKPSSSQKSKESTSKQS* 405
lcl|XM_00468690 RDFISRLLKHNPSQRPTLKEVLEHPWITANSSKPPSSQKNKESTSKQS* 406
lcl|XM_00341990 RDLVSRLLKHNPSQRLTLKEVLEHPWITANSKKPPSSQNKEPTSKQS*- 406
lcl|XM_00432103 RDLISRLLKHNPSQRPTLKEVLEHPWITANSKPSSSQKKESASKQS*-- 403
lcl|NM_004217.3 QDLISKLLRHNPSERLPLAQVSAHPWVRANSRRVLPPSALQSVA*---- 345
lcl|XM_01511855 QDLISKLLRHNPSERLPLAQVSAHPWVRANSRRVLPPSALQSVA*---- 345
lcl|NM_183084.2 QDLIYKLLKHNPSERLPLAQVSAHPWVRTHSRRVLPPSAPQSVP*---- 345
lcl|XM_00401268 QDLISKLLKHNPSERLPLAQVSAHPWVRAHSRRVLPPSAPQSVP*---- 345
lcl|XM_844813.4 QDLISKLLKHNPSERLPLSQVSAHPWVRAHSRRMLPPSAVQAIP*---- 345
lcl|XM_00399619 QDLISKLLKHNPSERLPLAQVSAHPWVRAHSRRVLPPSALQSVP*---- 345
lcl|NM_213919.2 QDLISKLLKHNPSDRLPLAQVSAHPWVRAHSRRVLPPSAPQSVP*---- 345
lcl|XM_00150481 QDLISKLLRHNPSERLPLAQVSAHPWVRAHSRRVLPPSALQSVP*---- 345
lcl|XM_00443309 QDLISKLLKHNPSERLPLAQVSAHPWVRAHSRRVLPPSAPQSVP*---- 345
lcl|XM_00827074 QDLISKLLRHNPSDRLTLAQVSAHPWVRAHSRRVLPPSAMQSVA*---- 344
lcl|XM_00587878 QDLISKLLKHNPSDRLPLAQVSAHPWVRAHSRRVLPPSALQSVH*---- 340
lcl|XM_00468461 QDLITKLLKHNPSERLPLTQVSAHPWVRAHSRRVLPPSALQSVP*---- 345
lcl|XM_00341691 QDLISKLLKHNPSERLPLAQVSAHPWVRAHSRRVLPPSALQSVP*---- 346
```

```
lcl|XM_01994424 QDLISKLLKHNPSERLPLAQVAAHPWVRAHSRRVLPPSALQSVP*---- 345
                :*:: :** ***..*  * :*  ***: :::
```
