# Supplementary material for: Differential Selective Pressures Experienced by the Aurora Kinase Gene Family
Source: Int J Mol Sci. 2017 Dec 28;19(1):72. doi: 10.3390/ijms19010072 (PMC5796022; doi:10.3390/ijms19010072)
Supplement: Supplementary file 1 [file ijms-19-00072-s001.zip › FigureS10AKACalignwLegend.pdf]

Figure S10. Alignment of placental mammal AKA and AKC sequences. The alignment of the placental mammal AKA and AKC sequences used for the dN/dS analyses.

```
MATLAB multiple sequence alignment


lcl|NM_198436.2 MDRSKENCISGPVKATAPVG-GPKRVLVTQQFPCQNPLPVNSGQAQRVLCPSNSSQRIPL  59
lcl|XM_01514873 MDRSKENCISGPVKPAAPVG-GPKRVLVTQQFPCQNPLPANSGQAQRVLCPSNSSQRVPL  59
lcl|NM_00103802 MDRCKENCISGP-KTAVPLSDGPKRVPVAQQFPSQNPVSVNSGQAQRVLCPTNSSQRVPS  59
lcl|NM_00114250 MDRCKENCISGP-KTAVPLSDGPKRVPVAQQFPSQNPVSVNSGQAQRVLCPTNSSQRVPS  59
lcl|XM_00563519 MDKSKENCIAGPVKTAIALGDGPKRVLVTQQVPSQNPLSANSGQAQRVLCPSNSSQRVPP  60
lcl|XM_00398335 MDKSKENCVAGPVKTTIPLGDGPKRVPVTQQFPSQNLLSANSGQAQRVLCPSNSSQRIPS  60
lcl|XM_00567303 MDKCKENCISGL-KTTVPPGDGPKRVPVTQHFPAQHLPSANSGQAQRVLCPSNSSQRLPS  59
lcl|XM_00148927 MDRSKENCISGPVKTTVPLGDGPKRVPVTQQLP-QNLLSANSGQAQRVLCPSNSSHRVPS  59
lcl|XM_00443024 MDRSKENCVAGPVKTTIPLGDGPKRVLVTQQFPSQHPLSANSGQAQRVLCPSNSSHRAPT  60
lcl|XM_00827414 MERSKENCISGPIKAAALLGDGPKRVPVTQRFPSQNPVSACSGQAERVLCPSNSSQRVPS  60
lcl|XM_01452869 MDKSKENGISGLVKTTIPSGDGPKRVLVTQQFPSQYPLPANSGQAQRVLCPSNSSQQVPS  60
lcl|XM_00468690 MDKSKENCISGPVKTMVPLRDGAKRVPVHQHAPSQSALPASSGQAQRVLCPSN-SQRLPS  59
lcl|XM_00341990 MDKSKENCISGPIKTTLPLGDGPKRVPVTQPFPSQNPLSASGGQAQRVLCPSNSSQSIPS  60
lcl|XM_00432103 MDKCKENSISGP-KTTVPLGDGPKRVPVTQQFPSQNPVSANSGQAQRVLCPSNSSQRVPS  59
lcl|NM_00101587 MSS-------------------------------------------------------PR   5
lcl|XM_00109836 MSS-------------------------------------------------------PK   5
lcl|XM_00521979 MSH---------------------------------------------------------   3
lcl|XM_00401544 MSH---------------------------------------------------------   3
lcl|XM_00561607 MLS--------------------------------------------------------G   4
lcl|XM_00694080 MQS--------------------------------------------------------G   4
lcl|XM_02109730 ------------------------------------------------------------   0
lcl|XM_00150199 M-----------------------------------------------------------   1
lcl|XM_00443911 M-----------------------------------------------------------   1
lcl|XM_00824896 MQS---------------------------------------------------------   3
lcl|XM_01453723 MNP---------------------------------------------------------   3
lcl|XM_01273470 MKS---------------------------------------------------------   3
lcl|XM_00340664 MFT---------------------------------------------------------   3
lcl|XM_01992887 ------------------------------------------------------------   0


lcl|NM_198436.2 QAQKLVSSHK--PVQNQKQKQLQATSVPHPVSRPLNNTQKSKQPLPSAPENNPEEELASK 117
lcl|XM_01514873 QAQKLVSSHK--LVQNQKQKQLQATSVPHPVSRPPSHTPKSKQPLPSAPENNPEEELASK 117
lcl|NM_00103802 QAQKLVSIQK--PVQTLKQKPPQAASAPRPVTRPPSNTQKSKQPQPPAPGNNPEKEVASK 117
lcl|NM_00114250 QAQKLVSIQK--PVQTLKQKPPQAASAPRPVARPPSNTQKSKQPQPPAPGNNPEKEVASK 117
lcl|XM_00563519 QTQKLVSSHK--PAQNLKQKQLQATGVPRPASRSLNNTQKSEQPSSSAPGNNSEKELATK 118
lcl|XM_00398335 QTQKLVSSHK--PVQNLKQKQLQATSVPRSVSRPLNNTQKSEQPLPSATGNNSEKELTTK 118
lcl|XM_00567303 HTQKLVSSHK--PVQNLKQKQSQATSGPRPVSRPLSNTQQSEQPQPAAPGNNPEKEAASK 117
lcl|XM_00148927 QAQKLVSSHK--PVQNLKQKQSQATSVPHPVPRPLNNTQRSEQLQPSAPGNNSEKELASK 117
lcl|XM_00443024 QAQKLVSSHR--PVQNLKQKQSQATSVPPPVSRPLNSTQKSEQPQPSAPGNNSEKERTSK 118
lcl|XM_00827414 QAQKLLPAEK----PVQKLKQAQAASVPRPVSKPPNNTPKSEQPPPSAPGNNPEKELTSN 116
lcl|XM_01452869 KAQKLVSSHK--PVLNLKQKQLQATSGPCPVSRPLNNTQKSEQPQPPAPGKNSEKEVASK 118
lcl|XM_00468690 QAQKLVSSHKAVPNPNLKQKQLQANNAPRAAPRPLAGTPKSEPP-PSAPGNNSEKEQASK 118
lcl|XM_00341990 QAQKLVSSRK--PVQSQKQKPLQITTVPRPVSRPPNNTPKGEQAQPPASGNNSGKELASK 118
lcl|XM_00432103 QVQKLVSSQK--SVQPLKQKPTQAASAPRSVSRPLSNTQKSEQPQLPAPGNNPEKEAASK 117
lcl|NM_00101587 AVVQLGKAQP--------------------------------------AGEELATANQTA  27
lcl|XM_00109836 AAVQLGRAQP--------------------------------------AGEAVTAANQTA  27
lcl|XM_00521979 ----RGTARK--------------------------------------AGGMQHAAAAGQ  21
lcl|XM_00401544 ----RGTARK--------------------------------------AGGMQPAAAAGQ  21
lcl|XM_00561607 SAQPASEGE---------------------------------------KTQEQSKDAAGH  25
lcl|XM_00694080 SVQPAAEEEE--------------------------------------QTRDQGTAAAGL  26
lcl|XM_02109730 -------MKS--------------------------------------GSVQPAVAAAGQ  15
lcl|XM_00150199 -SLPEAVMKS--------------------------------------GSVRPAVPTAGQ  22
lcl|XM_00443911 -SLPRAVMKL--------------------------------------GDVQSAGETAGQ  22
lcl|XM_00824896 -----------------------------------------------------GSSQAAG  10
lcl|XM_01453723 -GFLLSI---------------------------------------------IAEKKEPW  17
lcl|XM_01273470 -------------------------------------------------GDAQAAGAAGG  14
```

```
lcl|XM_00340664 -NVALSFARR--------------------------------------EWKELAPTERAL  24
lcl|XM_01992887 -------MKS--------------------------------------GSAQPA-AVAGQ  14


lcl|NM_198436.2 QKNEESKKRQWALEDFEIGRPLGKGKFGNVYLAREKQSKFILALKVLFKAQLEKAGVEHQ 177
lcl|XM_01514873 QKNEESKKRQWALEDFEIGRPLGKGKFGNVYLAREKQSKFILALKVLFKAQLEKAGVEHQ 177
lcl|NM_00103802 QKNEESKKRQWALEDFEIGRPLGKGKFGNVYLAREKQSKFILALKVLFKAQLEKAGVEHQ 177
lcl|NM_00114250 QKNEESKKRQWALEDFEIGRPLGKGKFGNVYLAREKQSKFILALKVLFKAQLEKAGVEHQ 177
lcl|XM_00563519 QKNEESKKRQWALEDFEIGRPLGKGKFGNVYLAREKQSKFILAIKVLFKAQLEKAGVEHQ 178
lcl|XM_00398335 QKNEESKKRQWALEDFEIGRPLGKGKFGNVYLAREKQSKFILALKVLFKAQLEKAGVEHQ 178
lcl|XM_00567303 QKNEESKKRQWALEDFEIGRPLGKGKFGNVYLAREKQSKFILALKVLFKTQLEKAGVEHQ 177
lcl|XM_00148927 EKNEESKKRQWALEDFEIGRPLGKGKFGNVYLAREKQSKFILALKVLFKAQLEKAGVEHQ 177
lcl|XM_00443024 EKNEDSKRRQWALEDFEIGRPLGKGKFGNVYLAREKQSKFILALKVLFKAQLEKAGVEHQ 178
lcl|XM_00827414 QKNEESKKRQWTLEDFDIGRPLGKGKFGNVYLAREKQSKFILALKVLFKAQLEKAGVEHQ 176
lcl|XM_01452869 QKNEESKK-QWTLEDFEIGRPLGKGKFGNVYLAREKQSKFILALKVLFKAQLEKAGVEHQ 177
lcl|XM_00468690 QKTEESKKRQWALEDFEIGRPLGKGKFGNVYLAREKQSKFILALKVLFKAQLEKAGVEHQ 178
lcl|XM_00341990 QKKEESKRRQWALEDFEIGRPLGKGKFGNVYLAREKQSKFILALKVLFKAQLEKAGVEHQ 178
lcl|XM_00432103 QKNEESKRRQWALEDFEIGRPLGKGKFGNVYLAREKQSKFILALKVLFKAQLEKAGVEHQ 177
lcl|NM_00101587 QQPSSPAMRRLTVDDFEIGRPLGKGKFGNVYLARLKESHFIVALKVLFKSQIEKEGLEHQ  87
lcl|XM_00109836 QQPSSPAMRRLKIDDFEIGRPLGKGKFGNVYLARLKESHFIVALKVLFKSQIEKEGLEHQ  87
lcl|XM_00521979 TVPGAPTARRFTVDDFEIGRPLGKGKFGNVYLARLKKNHFIVALKVLFKSQIEKEGLEHQ  81
lcl|XM_00401544 SVPGAPAARRFTIDDFEIGRPLGKGKFGNVYLARLKKNHFIVALKVLFKSQIEKEGLEHQ  81
lcl|XM_00561607 VELSVPPGRRLTIDDFEIGRPLGKGKFGNVYLARLKESHFIVALKVLFKSQIEKEGLEHQ  85
lcl|XM_00694080 VELGMPPVRRLTIEDFEIGRPLGKGKFGNVYLARLKESHFLVALKVIFKSQIEKEGLEHQ  86
lcl|XM_02109730 TVSNIPTGRRLTIDDFEIGRPLGKGKFGNVYLARLKESHFIVALKVLFKSQIEKEGLEHQ  75
lcl|XM_00150199 SQPGFPNVRHLTIDDFEIGRPLGKGKFGNVYLARLKESHFIVALKVLFKSQIEKEGLEHQ  82
lcl|XM_00443911 TQPSFPTVRHFTIDDFEIGRPLGKGKFGNVYLARLKESHFIVALKVLFKSQIEKEGLEHQ  82
lcl|XM_00824896 PAQPSVPLRRLTIEDFEIGRPLGKGKFGNVYLARLKDSHFIVALKVLFKSQIEKEGMEHQ  70
lcl|XM_01453723 PSSPSPGSRSLTIDDFDIRCPLGKGKFGNVYLARLRDSHFIVALKVLFKSQLEKEGMEHQ  77
lcl|XM_01273470 PALPVATVRRLTIDDFEIGRPLGKGKFGNVYLARLRESRFLVALKVLFKSQIEKEGLEHQ  74
lcl|XM_00340664 YGDVLLEMRRLRIDDFEIGRPLGKGKFGNVYLARLQENHFLVALKVLFKSQIEKEGLEHQ  84
lcl|XM_01992887 TMPSIPTVRRLTIDDFEIGRPLGKGKFGNVYLARLKESHFIVALKVLFKSQIEKEGLEHQ  74
                            ::**:*  ************** :..:*::*:**:**:*:** *:***

lcl|NM_198436.2 LRREVEIQSHLRHPNILRLYGYFHDATRVYLILEYAPLGTVYRELQKLSKFDEQRTAT-Y 236
lcl|XM_01514873 LRREVEIQSHLRHPNILRLYGYFHDATRVYLILEYAPLGTVYRELQKLSKFDEQRTAT-Y 236
lcl|NM_00103802 LRREVEIQSHLRHPNILRLYGYFHDATRVYLILEYAPLGAVYRELQKLSKFDEQRTAT-Y 236
lcl|NM_00114250 LRREIKIQSHLKHPNILRLYGYFHDATRVYLILEYAPLGAVYRELQKLSKFDEQRTAT-Y 236
lcl|XM_00563519 LRREVEIQSHLRHPNILRLYGYFHDATRVYLILEYAPLGAVYRELQKLSKFDEQRTAT-Y 237
lcl|XM_00398335 LRREVEIQSHLRHPNILRLYGYFHDATRVYLILEYAPLGAVYRELQKLSKFDEQRTAT-Y 237
lcl|XM_00567303 LRREVEIQSHLRHPNILRLYGYFHDATRVYLILEYAPLGAVYRELQKLSKFDEQRTAT-Y 236
lcl|XM_00148927 LRREVEIQSHLRHPNILRLYGYFHDATRVYLILEYAPLGAVYRELQKLSKFDEQRTAT-Y 236
lcl|XM_00443024 LRREVEIQSHLRHPNILRLYGYFHDATRVYLILEYAPLGAVYRELQKLSKFDEQRTAT-Y 237
lcl|XM_00827414 LRREVEIQSHLRHPNILRLYGYFHDATRVYLILEYAPLGTVYRELQKLSKFDEQRTAT-Y 235
lcl|XM_01452869 IRREVEIQSHLRHPNILRLYGYFHDATRVYLILEYAPLGTVYRELQKLSRFDEQRTAT-Y 236
lcl|XM_00468690 LRREVEIQSHLRHPNILRLYGYFHDATRVYLILEYAPLGAVYRELQKLSKFDEQRTAT-Y 237
lcl|XM_00341990 VRREVEIQSHLRHPNILRLYGYFHDATRVYLILEYAPLGTVYRELQKXSKFDEQRTAP*Y 238
lcl|XM_00432103 LRREVEIQSHLRHPNILRLYGYFHDATRVYLILEYAPLGAVYRELQKLSKFDEQRTAT-Y 236
lcl|NM_00101587 LRREIEIQAHLQHPNILRLYNYFHDARRVYLILEYAPRGELYKELQKSEKLDEQRTAT-I 146
lcl|XM_00109836 LRREIEIQAHLQHPNILRLYNYFHDARRVYLILEFAPRGELYKELQKSEKLDEQRAAT-I 146
lcl|XM_00521979 LRREVEIQAHLQHPNILRLYNYFHDARRVYLILEYAPKGELYKALQRSHTFDEQRTAT-I 140
lcl|XM_00401544 LRREVEIQAHLQHPNILRLYNYFHDARRVYLILEYAPKGELYKALQRSHTFDEQRTAT-I 140
lcl|XM_00561607 LRREIEIQAHLQHPNILRLYNYFHDARRVYLILEYAPRGELYKELQKSHTLDEQHTAT-I 144
lcl|XM_00694080 LRREIEIQAHLQHPNILRLYNYFHDARRVYLILEYAPRGELYKELQKSNTLDEQHTAT-I 145
lcl|XM_02109730 LRREIEIQAHLQHPNILRLYNYFHDARRVYLILEYAPRGELYKELQKSHTLDEQRTAT-I 134
lcl|XM_00150199 LRREIEIQAHLQHPNILRLYNYFHDARRVYLILEYAPRGELYKELQKSHTLDEQRTAT-I 141
lcl|XM_00443911 LRREIEIQAHLQHPNILRLYNYFHDARRVYLILEYAPRGELYKELQKSHTLDEQRTAT-I 141
lcl|XM_00824896 LRREIEIQAHLQHPNILRLYNYFHDARRIYLILEYAPRGELYKELQKCHTLDEQRTAT-I 129
lcl|XM_01453723 LRREIEIQSHLQHPNILRLHNYFHDERXVYLILEYAPRGELYKELQKSHTIDEQSTAT-I 136
```

```
lcl|XM_01273470 LRREVEIQAHLRHPNILRLYNYFHDARRVYLILEYAPRGELYKELQNCHTLDEQRTAT-I 133
lcl|XM_00340664 LRREIEIQAHLQHPNILRLYNYFHDSRRVYLILEYAPRGELYKELQKSHRLDEQRTAT-I 143
lcl|XM_01992887 LRREIEIQAHLQHPNILRLYNYFHDARRMYLILEYAPRGELYKELQKSHTLDEQRTAT-V 133
                :***::**:**:*******:.****   :*****:** * :*: **.   :*** :*.

lcl|NM_198436.2 ITELANALSYCHSKRVIHRDIKPENLLLGSAGELKIADFGWSVHAPSSRRTTLCGTLDYL 296
lcl|XM_01514873 ITELANALSYCHSKRVIHRDIKPENLLLGSAGELKIADFGWSVHAPSSRRTTLCGTLDYL 296
lcl|NM_00103802 ITELANALSYCHSKRVIHRDIKPENLLLGSAGELKIADFGWSVHAPSSRRTTLCGTLDYL 296
lcl|NM_00114250 ITELANALSYCHSKRVIHRDIKPENLLLGSAGELKIADFGWSVHAPSSRRTTLCGTLDYL 296
lcl|XM_00563519 ITELADALSYCHSKRVIHRDIKPENLLLGSAGELKIADFGWSVHAPSSRRTTLCGTLDYL 297
lcl|XM_00398335 ITELADALSYCHSKRVIHRDIKPENLLLGSAGELKIADFGWSVHAPSSRRTTLCGTLDYL 297
lcl|XM_00567303 ITELANALSYCHSKRVIHRDIKPENLLLGSAGELKIADFGWSVHAPSSRRTTLCGTLDYL 296
lcl|XM_00148927 ITELANALAYCHSKRVIHRDIKPENLLLGSAGELKIADFGWSVHAPSSRRTTLCGTLDYL 296
lcl|XM_00443024 ITELANALAYCHSKRVIHRDIKPENLLLGSAGELKIADFGWSVHAPSSRRTTLCGTLDYL 297
lcl|XM_00827414 ITELANALLYCHSKRVIHRDIKPENLLLGSAGELKIADFGWSVHAPSSRRTTLCGTLDYL 295
lcl|XM_01452869 ITELTNALSYCHSKRVIHRDIKPENLLLGSTGELKIADFGWSVHAPSSRRTTLCGTLDYL 296
lcl|XM_00468690 ITELADALSYCHSKRVIHRDIKPENLLLGSAGELKIADFGWSVHAPSSRRTTLCGTLDYL 297
lcl|XM_00341990 ITELADALSYCHSKKVIHRDIKPENLLLGSTGELKIADFGWSVHAPSSRRTTLCGTLDYL 298
lcl|XM_00432103 ITELANALSYCHSKRVIHRDIKPENLLLGSSGELKIADFGWSVHAPSSRRTTLCGTLDYL 296
lcl|NM_00101587 IEELADALTYCHDKKVIHRDIKPENLLLGFRGEVKIADFGWSVHTPSLRRKTMCGTLDYL 206
lcl|XM_00109836 IEELADALTYCHEKKVIHRDIKPENLLLGFRGEVKIADFGWSVHTPSLRRKTMCGTLDYL 206
lcl|XM_00521979 IEELADALIYCHERKVIHRDIKPENLLLGLMGEVKIADFGWSVHTPSLRRRTTCGTLDYL 200
lcl|XM_00401544 IEELADALIYCHEKKVIHRDIKPENLLLGLMGEVKIADFGWSVHTPSLRRRTTCGTLDYL 200
lcl|XM_00561607 MEELADALTYCHEKKVIHRDIKPENLLLGFKGEVKIADFGWSVHTPSLRRKTMCGTLDYL 204
lcl|XM_00694080 MEELADALTYCHEKKVIHRDIKPENLLLGFRGEVKIADFGWSVHTPSLRRKTMCGTLDYL 205
lcl|XM_02109730 MEELADALTYCHEKKVIHRDIKPENLLLGFRGEVKIADFGWSVHTPSLRRKTMCGTLDYL 194
lcl|XM_00150199 MEELADALTYCHEKKVIHRDIKPENLLLGFRGEVKIADFGWSVHTPSLRRKTMCGTLDYL 201
lcl|XM_00443911 MEELADALTYCHEKKVIHRDIKPENLLLGFRGEVKIADFGWSVHTPSLRRKTMCGTLDYL 201
lcl|XM_00824896 MEELADALTYCHGKKVIHRDIKPENLLLGFRGEVKIADFGWSVHTLSLRRKTMCGTLDYL 189
lcl|XM_01453723 MEELAHALAYCHENKVIHRDIKPENLLLGLRGEVKIADFGWSVHTPSLRRKTMCGTLDYL 196
lcl|XM_01273470 IEELADALTYCHEKKVIHRDIKPENLLLGYRGEVKIADFGWSVHTPSLRRKTMCGTLDYL 193
lcl|XM_00340664 MEELADALIYCHEKKVIHRDIKPENLLLGLRGEVKIADFGWSVHTLSLRRKTMCGTLDYL 203
lcl|XM_01992887 MEELADALTYCHEKKVIHRDIKPENLLLGFRGEVKIADFGWSVHTPSLRRRTMCGTLDYL 193
                : **:.** *** .:**************  **:**********: * ** * *******

lcl|NM_198436.2 PPEMIEGRMHDEKVDLWSLGVLCYEFLVGKPPFEANTYQETYKRISRVEFTFPDFVTEGA 356
lcl|XM_01514873 PPEMIEGRMHDEKVDLWSLGVLCYEFLVGKPPFEANTYQETYKRISRVEFTFPDFVTEGA 356
lcl|NM_00103802 PPEMIEGRMHDEKVDLWSLGVLCYEFLVGKPPFEADTYQETYRRISRVEFTFPDCVPEGA 356
lcl|NM_00114250 PPEMIEGRMHDEKVDLWSLGVLCYEFLVGKPPFEADTYQETYRRISRVEFTFPDCVPEGA 356
lcl|XM_00563519 PPEMIEGRMHDEKVDLWSLGVLCYEFLVGKPPFEASTYQETYKRISRVEFTFPDFVPEGA 357
lcl|XM_00398335 PPEMIEGRMHDEKVDLWSLGVLCYEFLVGKPPFEASTYQETYKRISRVEFTFPDFVPEGA 357
lcl|XM_00567303 PPEMIEGRMHDEKVDLWSLGVLCYEFLVGKPPFEANTYQETYKRISRVEFTFPDFVPEGA 356
lcl|XM_00148927 PPEMIEGRMHDEKVDLWSLGVLCYEFLVGKPPFEASTYQETYKRISRVEFTFPDFVPEGA 356
lcl|XM_00443024 PPEMIEGRMHDEKVDLWSLGVLCYEFLVGKPPFEASTYQETYKRISRVEFTFPDFVPDGA 357
lcl|XM_00827414 PPEMIEGRMHDEKVDLWSLGVLCYEFLVGKPPFEANTYQETYRRISRVEFTFPDFVTEGA 355
lcl|XM_01452869 PPEMIEGRMHDEKVDLWSLGVLCYEFLVGKPPFEASTYQETYKRISRVEFTFPDFVREGA 356
lcl|XM_00468690 PPEMIEGRMHDEKVDLWSLGVLCYEFLVGKPPFEASTYQETYKRISRVEFTFPDFVPEGA 357
lcl|XM_00341990 PPEMIEGRMHDEKVDLWSLGVLCYEFLVGKPPFEASTYQETYNRISRVEFTFPDLVTVGA 358
lcl|XM_00432103 PPEMIEGRMHDEKVDLWSLGVLCYEFLVGKPPFEANTYQETYKRISRVEFTFPDFVPEGA 356
lcl|NM_00101587 PPEMIEGRTYDEKVDLWCIGVLCYELLVGYPPFESASHSETYRRILKVDVRFPLSMPLGA 266
lcl|XM_00109836 PPEMIEGRTYDEKVDLWCIGVLCYELLVGYPPFESTSHSETYRRILKVDVRFPLSMPLGA 266
lcl|XM_00521979 PPEMIEGRTYDEKVDLWCIGVLCYELLVGNPPFESASTSETYRRILKVDLRFPPSMSSGA 260
lcl|XM_00401544 PPEMIEGRTYDEKVDLWCIGVLCYELLVGNPPFESASTSETYRRILKVDLRFPPSMSSGA 260
lcl|XM_00561607 PPEMIERRTYNEKVDLWCIGVLCYELLVGYPPFESPSHNETYRRILKVDLRFPPSLPLGA 264
lcl|XM_00694080 PPEMIERRTYNETVDLWCIGVLCYELLVGNPPFESHSHNETYRRILKVDIRFPPSIPLGA 265
lcl|XM_02109730 PPEMIEGRTYNEKVDLWCIGVLCYELLVGNPPFESASHNETYRRILKVDVRFPPSLPLGA 254
lcl|XM_00150199 PPEMIEGRTYNEKVDLWCIGVLCYELLVGNPPFESNSHNETYRRILKVDVRFPPSIPSGA 261
lcl|XM_00443911 PPEMIEGRTYNEKVDLWCIGVLCYELLVGNPPFESNSHNETYRRILKVDVRFPPSIPLGA 261
lcl|XM_00824896 PPEMIEGRTYDEKVDLWCIGVLCYELLVGNPPFESVSHTETYRRILKVDVKFPVTMSLGA 249
```

```
lcl|XM_01453723 PPEMVEQRTYSEMVDLWCIGVLCYELLVGNPPFKSSSYSETYRRILKVDVKFPPSIPLGA 256
lcl|XM_01273470 PPEMIEGRTYNEKVDLWCIGVLCYELLVGKPPFESSTHNETYRRILKVDVRFPPSVPLGA 253
lcl|XM_00340664 PPEMIEGRTYDERVDLWCIGVLCYELLVGNPPFESPSHTETYRRILKVDVRFPSSMPSGA 263
lcl|XM_01992887 PPEMIEGRTYNEKVDLWCIGVLCYELLVGNPPFESPSHNETYRRILQVDVRFPPSMPLGA 253
                ****:* * :.* ****.:******:*** ***:: :  ***.** :*:. **  :  **

lcl|NM_198436.2 RDLISRLLKHNPSQRPMLREVLEHPWITANSSKPSNCQNKESASKQS*- 404
lcl|XM_01514873 RDLISRLLKHNPSQRPMLREVLEHPWITANSSKPSNCQNRVN*------ 399
lcl|NM_00103802 RDLISRLLKHNPSQRPTLKEVLEHPWIIANSKPSSCQKKESTSKQS*-- 403
lcl|NM_00114250 RDLISRLLKHNPSQRPTLKEVLEHPWITANSKPSSCQKKESTSKQS*-- 403
lcl|XM_00563519 RDLISRLLKHNPSQRPTLKDVLEHPWIMANSSKPSSSQKNKDSTSKQS* 406
lcl|XM_00398335 RDLISRLLKHNPSQRPTLKEVLEHPWVTANSSKPSGSQKSKESTGKQS* 406
lcl|XM_00567303 RDLISRLLKHNPSHRPTLKEVLEHPWITANSKPASSHKKESTSKQP*-- 403
lcl|XM_00148927 RDLISRLLKHNPSQRPTLKEVLEHPWITANSSKPSNSQKSKESTSKQP* 405
lcl|XM_00443024 RDLISRLLKHNPSQRPTLKEVLEHPWITANASKPSNSQKSKESTSKQS* 406
lcl|XM_00827414 KDFISRLLKHNPSHRPTLAEVLEHPWVKAKSSKPSSCPDKEPASKQS*- 403
lcl|XM_01452869 RDLISRLLMHNPNQRLTLKEVLEHPWITANSSKPSSSQKSKESTSKQS* 405
lcl|XM_00468690 RDFISRLLKHNPSQRPTLKEVLEHPWITANSSKPPSSQKNKESTSKQS* 406
lcl|XM_00341990 RDLVSRLLKHNPSQRLTLKEVLEHPWITANSKKPPSSQNKEPTSKQS*- 406
lcl|XM_00432103 RDLISRLLKHNPSQRPTLKEVLEHPWITANSKPSSSQKKESASKQS*-- 403
lcl|NM_00101587 RDLISRLLRYQPLERLPLAQILKHPWVQAHSRRVLPPCAQMAS*----- 310
lcl|XM_00109836 RDLISKLLRYQPLERLPLAQILKHPWVQVHSRRVLPPCAQMAS*----- 310
lcl|XM_00521979 RDLISKLLRFQPLERLPLVRVLEHPWIRAHSQRVLPPSVPMAF*----- 304
lcl|XM_00401544 RDLISKLLRFQPLERLPLVRVLEHPWIRAHSQRVLPPSVQRAF*----- 304
lcl|XM_00561607 QDLVSKLLRYQPLERLPLAQIMEHPWVRAHSRRVLPPSVQMAS*----- 308
lcl|XM_00694080 RDLISKLLRYQPLERLPLSQILEHPWVRAHSRRVVPPSVQMDS*----- 309
lcl|XM_02109730 QDLISRLLRYQPSDRLPLDQILEHPWVRAHSRRVLPPSTQMVS*----- 298
lcl|XM_00150199 KDLISKLLRYQPLERLPLDQILEHPWVRAHSRRVLPPSAQMAS*----- 305
lcl|XM_00443911 KDLISKLLRYQPLERLPLDQIMEHPWVRAHSRRVLPPSAQMAS*----- 305
lcl|XM_00824896 RDLISRLLRYQPSERLPLAQILRHPWVQAHSRRVLPPCAHPAS*----- 293
lcl|XM_01453723 QDLISKLLRYQPSERLLLAQVLQHPWVQAHSRRVGPPPAPSAS*----- 300
lcl|XM_01273470 QDLISSLLRYQPSERLPLAQILQHPWVRMHSRRVLPPSAQVAS*----- 297
lcl|XM_00340664 QDLISKLLRYQPFERLALAQVLEHPWVQAHSQRVLPPSAQMAS*----- 307
lcl|XM_01992887 QDLISKLLRYQPSERLPLVQILEHPWVRAHSRRVLPPSAQMVS*----- 297
                :*::* ** .:* .*  *  ::.***:  ::
```
