# Supplementary material for: Differential Selective Pressures Experienced by the Aurora Kinase Gene Family
Source: Int J Mol Sci. 2017 Dec 28;19(1):72. doi: 10.3390/ijms19010072 (PMC5796022; doi:10.3390/ijms19010072)
Supplement: Supplementary file 1 [file ijms-19-00072-s001.zip › FigureS1wlegend.pdf]

Figure S1. Alignment of AK sequences spanning major taxonomic groups. The alignment of 137 animal, fungal, protist, and plant AK sequences used to construct the phylogenetic tree.

```
CLUSTAL W(1.81) multiple sequence alignment


XP_020952967.1/1-297                    ------------------------------------------------------------
XP_012380002.1/1-290                    ------------------------------------------------------------
XP_006102660.1/1-339                    ------------------------------------------------------------
XP_005673091.1/1-402                    ------------------------------------------------------------
XP_004484680.1/1-405                    ------------------------------------------------------------
XP_004462482.1/1-349                    ------------------------------------------------------------
Q6NW76.1/1-320                          ------------------------------------------------------------
NP_999084.2/1-344                       ------------------------------------------------------------
gi|1190438012|ref|XP_020822491.1|/1-365 ------------------------------------------------------------
gi|1190373986|ref|XP_020837071.1|/1-405 ------------------------------------------------------------
gi|1154067554|ref|NP_001153906.2|/1-332 ------------------------------------------------------------
gi|1131237831|ref|XP_019799799.1|/1-344 ------------------------------------------------------------
gi|1131178530|ref|XP_019784431.1|/1-296 ------------------------------------------------------------
gi|1126479796|ref|XP_006940862.2|/1-308 ------------------------------------------------------------
gi|1113784803|ref|XP_019353949.1|/1-376 ------------------------------------------------------------
gi|1022987698|ref|XP_016286291.1|/1-365 ------------------------------------------------------------
gi|966994491|ref|XP_014974040.1|/1-344  ------------------------------------------------------------
gi|966965902|ref|XP_015004225.1|/1-398  ------------------------------------------------------------
gi|955535802|ref|XP_004439167.2|/1-335  ------------------------------------------------------------
gi|951053762|ref|XP_014465099.1|/1-416  ------------------------------------------------------------
gi|946796851|ref|XP_014384184.1|/1-404  ------------------------------------------------------------
gi|946756071|ref|XP_014392719.1|/1-299  ------------------------------------------------------------
gi|922383691|ref|XP_013462066.1|/1-300  ------------------------------------------------------------
gi|909794683|ref|XP_013153467.1|/1-419  ------------------------------------------------------------
gi|891599507|ref|XP_013025412.1|/1-351  ------------------------------------------------------------
gi|891565640|ref|XP_013019059.1|/1-351  ------------------------------------------------------------
gi|884853217|ref|XP_013010870.1|/1-315  ------------------------------------------------------------
gi|830244266|ref|XP_012590158.1|/1-296  ------------------------------------------------------------
gi|828211890|ref|XP_012558808.1|/1-339  ------------------------------------------------------------
gi|820033943|ref|NP_001295465.1|/1-282  ------------------------------------------------------------
gi|795377289|ref|XP_011750175.1|/1-471  MRTPWVYALFMLSGPLPSPPRRTLCLLPSRASPLGLAFRAQPRPARSSSAPTRGPWAESP
gi|779989227|ref|XP_011666707.1|/1-321  ------------------------------------------------------------
gi|761905244|ref|XP_011402440.1|/1-385  ------------------------------------------------------------
gi|759175630|ref|XP_011377486.1|/1-303  ------------------------------------------------------------
gi|759149200|ref|XP_011368486.1|/1-311  ------------------------------------------------------------
gi|759116391|ref|XP_011357226.1|/1-339  ------------------------------------------------------------
gi|758991346|ref|XP_957232.2|/1-420     ------------------------------------------------------------
gi|743922878|ref|XP_011005517.1|/1-293  ------------------------------------------------------------
gi|698438703|ref|XP_009757361.1|/1-296  ------------------------------------------------------------
gi|697188794|ref|XP_009603442.1|/1-294  ------------------------------------------------------------
gi|658871623|ref|XP_008418750.1|/1-330  ------------------------------------------------------------
gi|657559162|ref|XP_008283516.1|/1-424  ------------------------------------------------------------
gi|657529148|ref|XP_008293493.1|/1-319  ------------------------------------------------------------
gi|655887082|ref|XP_008247187.1|/1-292  ------------------------------------------------------------
gi|655881324|ref|XP_008272369.1|/1-402  ------------------------------------------------------------
gi|655868725|ref|XP_008268971.1|/1-343  ------------------------------------------------------------
gi|641774253|ref|XP_008170712.1|/1-431  ------------------------------------------------------------
gi|620969612|ref|XP_007654691.1|/1-303  ------------------------------------------------------------
gi|617451788|ref|XP_007568855.1|/1-331  ------------------------------------------------------------
gi|594674988|ref|XP_007185333.1|/1-402  ------------------------------------------------------------
gi|594654199|ref|XP_007175509.1|/1-282  ------------------------------------------------------------
gi|594623572|ref|XP_007166415.1|/1-344  ------------------------------------------------------------
gi|584038001|ref|XP_006753367.1|/1-368  ------------------------------------------------------------
gi|573912300|ref|XP_006643864.1|/1-292  ------------------------------------------------------------
gi|554569173|ref|XP_005878848.1|/1-339  ------------------------------------------------------------
gi|545541126|ref|XP_005635254.1|/1-405  ------------------------------------------------------------
gi|545487136|ref|XP_005616134.1|/1-307  ------------------------------------------------------------
gi|544514002|ref|XP_005590555.1|/1-471  MRTPWVYALFMLSGPLPSPPRRTLCLLPSRASPLGLAFRAQPRPARSSSAPTRGPWAESP
gi|530653727|ref|XP_005313062.1|/1-365  ------------------------------------------------------------
gi|528993703|ref|XP_005219854.1|/1-303  ------------------------------------------------------------
gi|524890835|ref|XP_005101959.1|/1-332  ------------------------------------------------------------
gi|514776534|ref|XP_004968502.1|/1-292  ------------------------------------------------------------
gi|507960369|ref|XP_004686961.1|/1-405  ------------------------------------------------------------
gi|507953708|ref|XP_004684672.1|/1-344  ------------------------------------------------------------
gi|478517838|ref|XP_004433150.1|/1-344  ------------------------------------------------------------
gi|478512086|ref|XP_004430306.1|/1-405  ------------------------------------------------------------
gi|470628341|ref|XP_004321082.1|/1-402  ------------------------------------------------------------
```

```
gi|470261727|ref|XP_004360485.1|/1-457  --------------------------MNKTNATSGGMSTFKATSNAMKENHHPATTTTTK
gi|449486478|ref|XP_002195961.2|/1-319  ------------------------------------------------------------
gi|426243291|ref|XP_004015492.1|/1-303  ------------------------------------------------------------
gi|426237569|ref|XP_004012730.1|/1-344  ------------------------------------------------------------
gi|410979749|ref|XP_003996244.1|/1-344  ------------------------------------------------------------
gi|410953494|ref|XP_003983405.1|/1-405  ------------------------------------------------------------
gi|410914403|ref|XP_003970677.1|/1-332  ------------------------------------------------------------
gi|389641901|ref|XP_003718583.1|/1-397  ------------------------------------------------------------
gi|389635857|ref|XP_003715581.1|/1-400  ------------------------------------------------------------
gi|357126095|ref|XP_003564724.1|/1-291  ------------------------------------------------------------
gi|355703962|gb|EHH30453.1|/1-309       ------------------------------------------------------------
gi|350538969|ref|NP_001233545.1|/1-403  ------------------------------------------------------------
gi|347966592|ref|XP_321274.5|/1-404     ------------------------------------------------------------
gi|345800233|ref|XP_849906.2|/1-344     ------------------------------------------------------------
gi|344296517|ref|XP_003419953.1|/1-405  ------------------------------------------------------------
gi|344290468|ref|XP_003416960.1|/1-345  ------------------------------------------------------------
gi|344269722|ref|XP_003406697.1|/1-306  ------------------------------------------------------------
gi|338709975|ref|XP_001502047.3|/1-304  ------------------------------------------------------------
gi|334312551|ref|XP_001378079.2|/1-402  ------------------------------------------------------------
gi|332848444|ref|XP_511856.3|/1-344     ------------------------------------------------------------
gi|330793523|ref|XP_003284833.1|/1-359  ------------------------------------------------------------
gi|321461440|gb|EFX72472.1|/1-304       ------------------------------------------------------------
gi|308497987|ref|XP_003111180.1|/1-306  ------------------------------------------------------------
gi|302801442|ref|XP_002982477.1|/1-288  ------------------------------------------------------------
gi|302688103|ref|XP_003033731.1|/1-328  ------------------------------------------------------------
gi|297802712|ref|XP_002869240.1|/1-290  ------------------------------------------------------------
gi|281427796|ref|NP_001164000.1|/1-322  ------------------------------------------------------------
gi|268565413|ref|XP_002639437.1|/1-302  ------------------------------------------------------------
gi|260784959|ref|XP_002587531.1|/1-282  ------------------------------------------------------------
gi|226499040|ref|NP_001148791.1|/1-296  ------------------------------------------------------------
gi|224088986|ref|XP_002308590.1|/1-293  ------------------------------------------------------------
gi|215983064|ref|NP_001135979.1|/1-405  ------------------------------------------------------------
gi|213404608|ref|XP_002173076.1|/1-346  ------------------------------------------------------------
gi|198430178|ref|XP_002119314.1|/1-305  ------------------------------------------------------------
gi|196012778|ref|XP_002116251.1|/1-270  ------------------------------------------------------------
gi|168044897|ref|XP_001774916.1|/1-297  ------------------------------------------------------------
gi|159468203|ref|XP_001692272.1|/1-292  ------------------------------------------------------------
gi|158297402|ref|XP_317640.3|/1-307     ------------------------------------------------------------
gi|156368106|ref|XP_001627537.1|/1-314  ------------------------------------------------------------
gi|149734120|ref|XP_001489320.1|/1-404  ------------------------------------------------------------
gi|149724949|ref|XP_001504864.1|/1-344  ------------------------------------------------------------
gi|148225464|ref|NP_001085657.1|/1-368  ------------------------------------------------------------
gi|146172296|ref|XP_001018388.2|/1-360  ------------------------------------------------------------
gi|144226857|ref|NP_001016859.2|/1-360  ------------------------------------------------------------
gi|125524746|gb|EAY72860.1|/1-292       ------------------------------------------------------------
gi|115749607|ref|NP_898907.2|/1-344     ------------------------------------------------------------
gi|115455545|ref|NP_001051373.1|/1-309  ------------------------------------------------------------
gi|115435032|ref|NP_001042274.1|/1-292  ------------------------------------------------------------
gi|84000031|ref|NP_001033117.1|/1-402   ------------------------------------------------------------
gi|83776600|ref|NP_004208.2|/1-344      ------------------------------------------------------------
gi|76253851|ref|NP_695208.2|/1-397      ------------------------------------------------------------
gi|68013217|ref|NP_001018849.1|/1-355   ------------------------------------------------------------
gi|66815573|ref|XP_641803.1|/1-384      ------------------------------------------------------------
gi|62865639|ref|NP_001015878.1|/1-309   ------------------------------------------------------------
gi|62857967|ref|NP_001016570.1|/1-415   ------------------------------------------------------------
gi|57525577|ref|NP_001003640.1|/1-405   ------------------------------------------------------------
gi|50759065|ref|XP_425725.1|/1-409      ------------------------------------------------------------
gi|50303387|ref|XP_451635.1|/1-361      ------------------------------------------------------------
gi|46358064|ref|NP_035627.1|/1-417      ------------------------------------------------------------
gi|45198420|ref|NP_985449.1|/1-367      ------------------------------------------------------------
gi|38327570|ref|NP_940838.1|/1-403      ------------------------------------------------------------
gi|17505246|ref|NP_491714.1|/1-305      ------------------------------------------------------------
gi|17137514|ref|NP_477336.1|/1-329      ------------------------------------------------------------
gi|17136516|ref|NP_476749.1|/1-411      ------------------------------------------------------------
gi|16758580|ref|NP_446201.1|/1-343      ------------------------------------------------------------
gi|15233958|ref|NP_195009.1|/1-294      ------------------------------------------------------------
gi|15225495|ref|NP_182073.1|/1-288      ------------------------------------------------------------
gi|6708213|gb|AAF25838.1|/1-282         ------------------------------------------------------------
gi|6325047|ref|NP_015115.1|/1-367       ------------------------------------------------------------
gi|2979623|gb|AAC12683.1|/1-345         ------------------------------------------------------------
```

```
XP_020952967.1/1-297                     ------------------------------------------------------------
XP_012380002.1/1-290                     ------------------------------------------------------------
XP_006102660.1/1-339                     ------------------------------------------------------------
XP_005673091.1/1-402                     ------------------------------------------------------------
XP_004484680.1/1-405                     ------------------------------------------------------------
XP_004462482.1/1-349                     ------------------------------------------------------------
Q6NW76.1/1-320                           ------------------------------------------------------------
NP_999084.2/1-344                        ------------------------------------------------------------
gi|1190438012|ref|XP_020822491.1|/1-365 ------------------------------------------------------------
gi|1190373986|ref|XP_020837071.1|/1-405 ------------------------------------------------------------
gi|1154067554|ref|NP_001153906.2|/1-332 ------------------------------------------------------------
gi|1131237831|ref|XP_019799799.1|/1-344 ------------------------------------------------------------
gi|1131178530|ref|XP_019784431.1|/1-296 ------------------------------------------------------------
gi|1126479796|ref|XP_006940862.2|/1-308 ------------------------------------------------------------
gi|1113784803|ref|XP_019353949.1|/1-376 ------------------------------------------------------------
gi|1022987698|ref|XP_016286291.1|/1-365 ------------------------------------------------------------
gi|966994491|ref|XP_014974040.1|/1-344  ------------------------------------------------------------
gi|966965902|ref|XP_015004225.1|/1-398  ------------------------------------------------------------
gi|955535802|ref|XP_004439167.2|/1-335  ------------------------------------------------------------
gi|951053762|ref|XP_014465099.1|/1-416  ------------------------------------------------------------
gi|946796851|ref|XP_014384184.1|/1-404  ------------------------------------------------------------
gi|946756071|ref|XP_014392719.1|/1-299  ------------------------------------------------------------
gi|922383691|ref|XP_013462066.1|/1-300  ------------------------------------------------------------
gi|909794683|ref|XP_013153467.1|/1-419  ------------------------------------------------------------
gi|891599507|ref|XP_013025412.1|/1-351  ---------------------------------------------------MSDSKLAGS
gi|891565640|ref|XP_013019059.1|/1-351  ---------------------------------------------------MTDSKLAGS
gi|884853217|ref|XP_013010870.1|/1-315  ------------------------------------------------------------
gi|830244266|ref|XP_012590158.1|/1-296  ------------------------------------------------------------
gi|828211890|ref|XP_012558808.1|/1-339  ------------------------------------------------------------
gi|820033943|ref|NP_001295465.1|/1-282  ------------------------------------------------------------
gi|795377289|ref|XP_011750175.1|/1-471  ISVFSIGRTSDGVRNFSQWESARKYLSERAAPGIKEGRAATALHEAADTGVCVCLGPREA
gi|779989227|ref|XP_011666707.1|/1-321  ------------------------------------------------------------
gi|761905244|ref|XP_011402440.1|/1-385  ------------------------------------------------------------
gi|759175630|ref|XP_011377486.1|/1-303  ------------------------------------------------------------
gi|759149200|ref|XP_011368486.1|/1-311  ------------------------------------------------------------
gi|759116391|ref|XP_011357226.1|/1-339  ------------------------------------------------------------
gi|758991346|ref|XP_957232.2|/1-420     -------------------------------------------------MAG--QPMESR
gi|743922878|ref|XP_011005517.1|/1-293  ------------------------------------------------------------
gi|698438703|ref|XP_009757361.1|/1-296  ------------------------------------------------------------
gi|697188794|ref|XP_009603442.1|/1-294  ------------------------------------------------------------
gi|658871623|ref|XP_008418750.1|/1-330  ------------------------------------------------------------
gi|657559162|ref|XP_008283516.1|/1-424  ------------------------------------------------------------
gi|657529148|ref|XP_008293493.1|/1-319  ------------------------------------------------------------
gi|655887082|ref|XP_008247187.1|/1-292  ------------------------------------------------------------
gi|655881324|ref|XP_008272369.1|/1-402  ------------------------------------------------------------
gi|655868725|ref|XP_008268971.1|/1-343  ------------------------------------------------------------
gi|641774253|ref|XP_008170712.1|/1-431  ----------------------------------------------------------MS
gi|620969612|ref|XP_007654691.1|/1-303  ------------------------------------------------------------
gi|617451788|ref|XP_007568855.1|/1-331  ------------------------------------------------------------
gi|594674988|ref|XP_007185333.1|/1-402  ------------------------------------------------------------
gi|594654199|ref|XP_007175509.1|/1-282  ------------------------------------------------------------
gi|594623572|ref|XP_007166415.1|/1-344  ------------------------------------------------------------
gi|584038001|ref|XP_006753367.1|/1-368  -------------------------------------MWGQRHQLRGPSGTYVRDDEVLG
gi|573912300|ref|XP_006643864.1|/1-292  ------------------------------------------------------------
gi|554569173|ref|XP_005878848.1|/1-339  ------------------------------------------------------------
gi|545541126|ref|XP_005635254.1|/1-405  ------------------------------------------------------------
gi|545487136|ref|XP_005616134.1|/1-307  ------------------------------------------------------------
gi|544514002|ref|XP_005590555.1|/1-471  ISVFSIGRTSDGVRNFSQWESARKYLSERAAPGIKEGRAATALHEAADTGACVCLGPREA
gi|530653727|ref|XP_005313062.1|/1-365  ------------------------------------------------------------
gi|528993703|ref|XP_005219854.1|/1-303  ------------------------------------------------------------
gi|524890835|ref|XP_005101959.1|/1-332  ------------------------------------------------------------
gi|514776534|ref|XP_004968502.1|/1-292  ------------------------------------------------------------
gi|507960369|ref|XP_004686961.1|/1-405  ------------------------------------------------------------
gi|507953708|ref|XP_004684672.1|/1-344  ------------------------------------------------------------
gi|478517838|ref|XP_004433150.1|/1-344  ------------------------------------------------------------
gi|478512086|ref|XP_004430306.1|/1-405  ------------------------------------------------------------
gi|470628341|ref|XP_004321082.1|/1-402  ------------------------------------------------------------
```

```
gi|470261727|ref|XP_004360485.1|/1-457  IVPPSKMAPQMTSSSTVPSSAPKVIGSSFQKPTTTTTTTLPSQEKILTSNTSVLNAAHHQ
gi|449486478|ref|XP_002195961.2|/1-319  ------------------------------------------------------------
gi|426243291|ref|XP_004015492.1|/1-303  ------------------------------------------------------------
gi|426237569|ref|XP_004012730.1|/1-344  ------------------------------------------------------------
gi|410979749|ref|XP_003996244.1|/1-344  ------------------------------------------------------------
gi|410953494|ref|XP_003983405.1|/1-405  ------------------------------------------------------------
gi|410914403|ref|XP_003970677.1|/1-332  ------------------------------------------------------------
gi|389641901|ref|XP_003718583.1|/1-397  -------------------------------------------------MATKAMQLEEC
gi|389635857|ref|XP_003715581.1|/1-400  ------------------------------------------------MRKSLRSARRTA
gi|357126095|ref|XP_003564724.1|/1-291  ------------------------------------------------------------
gi|355703962|gb|EHH30453.1|/1-309       ------------------------------------------------------------
gi|350538969|ref|NP_001233545.1|/1-403  ------------------------------------------------------------
gi|347966592|ref|XP_321274.5|/1-404     ------------------------------------------------------------
gi|345800233|ref|XP_849906.2|/1-344     ------------------------------------------------------------
gi|344296517|ref|XP_003419953.1|/1-405  ------------------------------------------------------------
gi|344290468|ref|XP_003416960.1|/1-345  ------------------------------------------------------------
gi|344269722|ref|XP_003406697.1|/1-306  ------------------------------------------------------------
gi|338709975|ref|XP_001502047.3|/1-304  ------------------------------------------------------------
gi|334312551|ref|XP_001378079.2|/1-402  ------------------------------------------------------------
gi|332848444|ref|XP_511856.3|/1-344     ------------------------------------------------------------
gi|330793523|ref|XP_003284833.1|/1-359  -----------MSSFTNKENVSNN--TSFSNQNKQPQRVLQQ-------NNNVTN-----
gi|321461440|gb|EFX72472.1|/1-304       ------------------------------------------------------------
gi|308497987|ref|XP_003111180.1|/1-306  ------------------------------------------------------------
gi|302801442|ref|XP_002982477.1|/1-288  ------------------------------------------------------------
gi|302688103|ref|XP_003033731.1|/1-328  ------------------------------------------------------------
gi|297802712|ref|XP_002869240.1|/1-290  ------------------------------------------------------------
gi|281427796|ref|NP_001164000.1|/1-322  ------------------------------------------------------------
gi|268565413|ref|XP_002639437.1|/1-302  ------------------------------------------------------------
gi|260784959|ref|XP_002587531.1|/1-282  ------------------------------------------------------------
gi|226499040|ref|NP_001148791.1|/1-296  ------------------------------------------------------------
gi|224088986|ref|XP_002308590.1|/1-293  ------------------------------------------------------------
gi|215983064|ref|NP_001135979.1|/1-405  ------------------------------------------------------------
gi|213404608|ref|XP_002173076.1|/1-346  ---------------------------------------------------MSESKLVDS
gi|198430178|ref|XP_002119314.1|/1-305  ------------------------------------------------------------
gi|196012778|ref|XP_002116251.1|/1-270  ------------------------------------------------------------
gi|168044897|ref|XP_001774916.1|/1-297  ------------------------------------------------------------
gi|159468203|ref|XP_001692272.1|/1-292  ------------------------------------------------------------
gi|158297402|ref|XP_317640.3|/1-307     ------------------------------------------------------------
gi|156368106|ref|XP_001627537.1|/1-314  ------------------------------------------------------------
gi|149734120|ref|XP_001489320.1|/1-404  ------------------------------------------------------------
gi|149724949|ref|XP_001504864.1|/1-344  ------------------------------------------------------------
gi|148225464|ref|NP_001085657.1|/1-368  ------------------------------------------------------------
gi|146172296|ref|XP_001018388.2|/1-360  ------------------------------------------------------------
gi|144226857|ref|NP_001016859.2|/1-360  ------------------------------------------------------------
gi|125524746|gb|EAY72860.1|/1-292       ------------------------------------------------------------
gi|115749607|ref|NP_898907.2|/1-344     ------------------------------------------------------------
gi|115455545|ref|NP_001051373.1|/1-309  ------------------------------------------------------------
gi|115435032|ref|NP_001042274.1|/1-292  ------------------------------------------------------------
gi|84000031|ref|NP_001033117.1|/1-402   ------------------------------------------------------------
gi|83776600|ref|NP_004208.2|/1-344      ------------------------------------------------------------
gi|76253851|ref|NP_695208.2|/1-397      ------------------------------------------------------------
gi|68013217|ref|NP_001018849.1|/1-355   ---------------------------------------------------MSDSKLADS
gi|66815573|ref|XP_641803.1|/1-384      -----------MSYPNNKENSNNIGGGSFSVPSKQPQRVLQQQ------NTNINNHQ---
gi|62865639|ref|NP_001015878.1|/1-309   ------------------------------------------------------------
gi|62857967|ref|NP_001016570.1|/1-415   ------------------------------------------------------------
gi|57525577|ref|NP_001003640.1|/1-405   ------------------------------------------------------------
gi|50759065|ref|XP_425725.1|/1-409      ------------------------------------------------------------
gi|50303387|ref|XP_451635.1|/1-361      ------------------------------------------------------------
gi|46358064|ref|NP_035627.1|/1-417      -----------------------------------------------------MAVEGEP
gi|45198420|ref|NP_985449.1|/1-367      ------------------------------------------------------------
gi|38327570|ref|NP_940838.1|/1-403      ------------------------------------------------------------
gi|17505246|ref|NP_491714.1|/1-305      ------------------------------------------------------------
gi|17137514|ref|NP_477336.1|/1-329      ------------------------------------------------------------
gi|17136516|ref|NP_476749.1|/1-411      ------------------------------------------------------------
gi|16758580|ref|NP_446201.1|/1-343      ------------------------------------------------------------
gi|15233958|ref|NP_195009.1|/1-294      ------------------------------------------------------------
gi|15225495|ref|NP_182073.1|/1-288      ------------------------------------------------------------
gi|6708213|gb|AAF25838.1|/1-282         ------------------------------------------------------------
gi|6325047|ref|NP_015115.1|/1-367       ------------------------------------------------------------
gi|2979623|gb|AAC12683.1|/1-345         ------------------------------------------------------------
```

```
XP_020952967.1/1-297                    -------------------------------------------------MKSGSV-----
XP_012380002.1/1-290                    ------------------------------------------------------------
XP_006102660.1/1-339                    ---------------------------------------MTQKENAYPWPYGRQT-----
XP_005673091.1/1-402                    ---------------MDKC-KENCISGL----KTTVPPGDGPKRVPVTQHFPAQH-----
XP_004484680.1/1-405                    ---------------MDKC-KENYISGPV---KTTVLSGDGPKRVLMTPQLPSQN-----
XP_004462482.1/1-349                    ---------------------------------------MAQKENAHPWPYGRQM-----
Q6NW76.1/1-320                          ---------------------------------------MQNKENREP----RVQQ----
NP_999084.2/1-344                       ---------------------------------------MAQKENTYPWPYGRQT-----
gi|1190438012|ref|XP_020822491.1|/1-365 -------------------------MAAKNSLHPLFLCKMSEKENAHLGSYSTSL-----
gi|1190373986|ref|XP_020837071.1|/1-405 ---------------MDKS-KENSVSGPSN--KTMTPLGEGPKRVPVTQQISSQN-----
gi|1154067554|ref|NP_001153906.2|/1-332 ---------------------------------------MQNKENHEPKGPHRPFA----
gi|1131237831|ref|XP_019799799.1|/1-344 ---------------------------------------MAQKENAYPWPYGRQT-----
gi|1131178530|ref|XP_019784431.1|/1-296 -------------------------------------------------MKSGSA-----
gi|1126479796|ref|XP_006940862.2|/1-308 --------------------------------------MQSGSVQPAAEEEEQTR-----
gi|1113784803|ref|XP_019353949.1|/1-376 ------MKRREPQARARTRRPSQPMGARGGADLKREAAAAAVGRGTRRMAHKENV-----
gi|1022987698|ref|XP_016286291.1|/1-365 -------------------------MEAKNSLHPFFLYKMSEKENVHLGSYSTSL-----
gi|966994491|ref|XP_014974040.1|/1-344  ---------------------------------------MAQKENAYPWPYGRQT-----
gi|966965902|ref|XP_015004225.1|/1-398  ---------------MDRS-KENCISGPV---KPAAPVG-GPKRVLVTQQFPCQN-----
gi|955535802|ref|XP_004439167.2|/1-335  -----------MLPPPTSFQDHPDPGIQFHGAQEGVVSPPSLMSLPRAVMKLGDV-----
gi|951053762|ref|XP_014465099.1|/1-416  ---------------MDRTRKENRPGLPTHGVKIPAHVGDGPKRVPVS-QLP-QS-----
gi|946796851|ref|XP_014384184.1|/1-404  ---------------MDKS-KENGISGLV---KTTIPSGDGPKRVLVTQQFPSQY-----
gi|946756071|ref|XP_014392719.1|/1-299  -----------------------------------------------MNPGFLLS-----
gi|922383691|ref|XP_013462066.1|/1-300  --------------------------------------------MAIATETQPQPH----
gi|909794683|ref|XP_013153467.1|/1-419  ---------------MDKSMKENHSGYPSRAAKISNPIEDGPKRVPVSQHSV-QS-----
gi|891599507|ref|XP_013025412.1|/1-351  VNQLSIS-----------------------SGSTLPSSANPGRQQLLKLAVSNQRQVN--
gi|891565640|ref|XP_013019059.1|/1-351  VNQLSMS-----------------------SGSALPNSANPGRQQLLKLAVSNQRQVN--
gi|884853217|ref|XP_013010870.1|/1-315  ------------------------------MDRSSPSPTSASASSVSSSSSSSLC-----
gi|830244266|ref|XP_012590158.1|/1-296  -------------------------------------------------MKSGDA-----
gi|828211890|ref|XP_012558808.1|/1-339  ------------------------------------------------------------
gi|820033943|ref|NP_001295465.1|/1-282  ------------------------------------------------------------
gi|795377289|ref|XP_011750175.1|/1-471  AGRAGLEAHQPESDPQPLSGRCEREQPSRGLRKASAPSPLLPMSSPKAAVQLGRA-----
gi|779989227|ref|XP_011666707.1|/1-321  ----------------------------------------------MHVHLSLQV-----
gi|761905244|ref|XP_011402440.1|/1-385  ------------------------MAGVSTNIRTLTTQVKGMTLGSASNKENATS-----
gi|759175630|ref|XP_011377486.1|/1-303  ------------------------------------------MSHSQAMVKSDSA-----
gi|759149200|ref|XP_011368486.1|/1-311  ------------------------------------------------KDSYSKT-----
gi|759116391|ref|XP_011357226.1|/1-339  ---------------------------------------MTQKENAYPWPYCRQT-----
gi|758991346|ref|XP_957232.2|/1-420     FGRMSIHDENNDPSDGARLQQKARELKSKAGPSQVQHTSAPGRPNLFKVALQSQSGNTVR
gi|743922878|ref|XP_011005517.1|/1-293  --------------------------------------------MAIAAEAQPQ------
gi|698438703|ref|XP_009757361.1|/1-296  --------------------------------------------MGIATENQPQ------
gi|697188794|ref|XP_009603442.1|/1-294  --------------------------------------------MAIAAETQPQ------
gi|658871623|ref|XP_008418750.1|/1-330  ---------------------------------------MQNKENYEPKG-LQRQ-----
gi|657559162|ref|XP_008283516.1|/1-424  --------------MDS---VKIPKDTKPQRPE-VKSHGNGPKRIPVSQQLIQKSVVTPT
gi|657529148|ref|XP_008293493.1|/1-319  ---------------------------------------MQ-------------FA----
gi|655887082|ref|XP_008247187.1|/1-292  ------------------------------------------------------M-----
gi|655881324|ref|XP_008272369.1|/1-402  ---------------MERS-KENCISGPI---KAAALLGDGPKRVPVTQRFPSQN-----
gi|655868725|ref|XP_008268971.1|/1-343  ---------------------------------------MAQKENAYPWPYSRPT-----
gi|641774253|ref|XP_008170712.1|/1-431  LHEISLQLNVLYLGNMEKNGKENHNGLPGHRVKTLPTIGDGPKRLPVHQQHP-QN-----
gi|620969612|ref|XP_007654691.1|/1-303  ------------------------------------------------------------
gi|617451788|ref|XP_007568855.1|/1-331  ---------------------------------------MQNKENYEPKG-LQRQF----
gi|594674988|ref|XP_007185333.1|/1-402  ---------------MDKC-KENSISGP----KTTVPLGDGPKRVPVTQQFPSQN-----
gi|594654199|ref|XP_007175509.1|/1-282  ------------------------------------------------------------
gi|594623572|ref|XP_007166415.1|/1-344  ---------------------------------------MAQKENAYPWPYGRQT-----
gi|584038001|ref|XP_006753367.1|/1-368  VAETGQDLQTGRSGARAVLEFSNCTSSRLFSSQHVPPPPKAPPLPVSALPSHWPS-----
gi|573912300|ref|XP_006643864.1|/1-292  --------------------------------------------MAIATESCCHE-----
gi|554569173|ref|XP_005878848.1|/1-339  ---------------------------------------MTQKENAYPWPYGRQT-----
gi|545541126|ref|XP_005635254.1|/1-405  ---------------MDKS-KENCIAGPV---KTAIALGDGPKRVLVTQQVPSQN-----
gi|545487136|ref|XP_005616134.1|/1-307  --------------------------------------MLSGSAQPASEGE-KTQ-----
gi|544514002|ref|XP_005590555.1|/1-471  AGRAGLEAHQPESDPQPLSGRCEREQPSRGLRKASAPSPLLPMSSPKAAVQLGRA-----
gi|530653727|ref|XP_005313062.1|/1-365  ------MAYKEN----VTPGTGTSLAKYG----------VLSGPGPQRVPRKDPY-----
gi|528993703|ref|XP_005219854.1|/1-303  ------------------------------------------MSHRGTARKAGGM-----
gi|524890835|ref|XP_005101959.1|/1-332  --------------------------------------------MDPASRVLKPN-----
gi|514776534|ref|XP_004968502.1|/1-292  --------------------------------------------MAIATESRGSE-----
gi|507960369|ref|XP_004686961.1|/1-405  ---------------MDKS-KENCISGPV---KTMVPLRDGAKRVPVHQHAPSQS-----
gi|507953708|ref|XP_004684672.1|/1-344  ---------------------------------------MAQKENAYPWPYGRQT-----
gi|478517838|ref|XP_004433150.1|/1-344  ---------------------------------------MAQKENAYPWPYGRQT-----
gi|478512086|ref|XP_004430306.1|/1-405  ---------------MDRS-KENCVAGPV---KTTIPLGDGPKRVLVTQQFPSQH-----
gi|470628341|ref|XP_004321082.1|/1-402  ---------------MDKC-KENSISGP----KTTVPLGDGPKRVPVTQQFPSQN-----
```

```
gi|470261727|ref|XP_004360485.1|/1-457  QHQPSNMKQPVPPTSFKQQQPVPQMLSKSQMAPITNQQHPTTTTTAAPTATISSAL----
gi|449486478|ref|XP_002195961.2|/1-319  -------------------MKENHAACP--------------------------------
gi|426243291|ref|XP_004015492.1|/1-303  ------------------------------------------MSHRGTARKAGGM-----
gi|426237569|ref|XP_004012730.1|/1-344  ---------------------------------------MAQKENAYPWPYGRQT-----
gi|410979749|ref|XP_003996244.1|/1-344  ---------------------------------------MAQKENAYPWPYGRQT-----
gi|410953494|ref|XP_003983405.1|/1-405  ---------------MDKS-KENCVAGPV---KTTIPLGDGPKRVPVTQQFPSQN-----
gi|410914403|ref|XP_003970677.1|/1-332  ---------------------------------------MQNKENYDPKSHTRTFP----
gi|389641901|ref|XP_003718583.1|/1-397  FEHLNVNDEND---HGDRLVQKS---KISMSTAQISHGSS--RPNLFKVALQSQSANTVT
gi|389635857|ref|XP_003715581.1|/1-400  INPVAVRDLLIR--CSSRPRSDS---RKLPRDAAVGDGNDDPRGNTQKEPARRTS--SPR
gi|357126095|ref|XP_003564724.1|/1-291  --------------------------------------------MAISTDSR-SE-----
gi|355703962|gb|EHH30453.1|/1-309       ------------------------------------------MSSPKAAVQLGRA-----
gi|350538969|ref|NP_001233545.1|/1-403  ---------------MDRS-KENCISGPV---KATAPVG-GPKRVLVTQQFPCQN-----
gi|347966592|ref|XP_321274.5|/1-404     ----------------MACNKENRKLLNGPSSVAA-STGIPSHSGTLLVKKGAGVG--GP
gi|345800233|ref|XP_849906.2|/1-344     ---------------------------------------MTQKENAYPWPYGRQT-----
gi|344296517|ref|XP_003419953.1|/1-405  ---------------MDKS-KENCISGPI---KTTLPLGDGPKRVPVTQPFPSQN-----
gi|344290468|ref|XP_003416960.1|/1-345  ---------------------------------------MAQKENAYPWPYGRQT-----
gi|344269722|ref|XP_003406697.1|/1-306  ----------------------------------------MFTNVALSFARREWK-----
gi|338709975|ref|XP_001502047.3|/1-304  ------------------------------------------MSLPEAVMKSGSV-----
gi|334312551|ref|XP_001378079.2|/1-402  ---------------MDIS-KENSVSGPSN--KTAILLGEGPKRVPITQQISSQS-----
gi|332848444|ref|XP_511856.3|/1-344     ---------------------------------------MAQKENAYPWPYGRQT-----
gi|330793523|ref|XP_003284833.1|/1-359  -------------TTATTKSTITK-----------------TTSASSTPSTSTPST----
gi|321461440|gb|EFX72472.1|/1-304       ------------------------------------------METKQPLKENSTN-----
gi|308497987|ref|XP_003111180.1|/1-306  -------------------------------------------MENKPEVIHDHD-----
gi|302801442|ref|XP_002982477.1|/1-288  ------------------------------------------------------M-----
gi|302688103|ref|XP_003033731.1|/1-328  ---------------------------------------MGGWRRRTRSAGSRCTATP--
gi|297802712|ref|XP_002869240.1|/1-290  --------------------------------------------MAIPTETQHQ------
gi|281427796|ref|NP_001164000.1|/1-322  -----------------------------------------------MSQRK------AP
gi|268565413|ref|XP_002639437.1|/1-302  -------------------------------------------MENKPQILQT-------
gi|260784959|ref|XP_002587531.1|/1-282  ------------------------------------------------------------
gi|226499040|ref|NP_001148791.1|/1-296  --------------------------------------------MAVATESRGSE-----
gi|224088986|ref|XP_002308590.1|/1-293  --------------------------------------------MAIASDTQPQ------
gi|215983064|ref|NP_001135979.1|/1-405  ------------MGIMDRC-KENCISGP----KTAVPLSDGPKRVPVAQQFPSQN-----
gi|213404608|ref|XP_002173076.1|/1-346  LNRLSVN-----------------------NGS----TVASGRQQLLRLAVSNQMHAN--
gi|198430178|ref|XP_002119314.1|/1-305  ------------------------------------------------------------
gi|196012778|ref|XP_002116251.1|/1-270  ------------------------------------------------------------
gi|168044897|ref|XP_001774916.1|/1-297  --------------------------------------------MGSDADVKYVR-----
gi|159468203|ref|XP_001692272.1|/1-292  ----------------------------------------------MLAQRQDAG-----
gi|158297402|ref|XP_317640.3|/1-307     ----------------------------------CANKSTPTMDDDVFATDGDVN-----
gi|156368106|ref|XP_001627537.1|/1-314  ------------------------------------------------------------
gi|149734120|ref|XP_001489320.1|/1-404  ---------------MDRS-KENCISGPV---KTTVPLGDGPKRVPVTQQLP-QN-----
gi|149724949|ref|XP_001504864.1|/1-344  ---------------------------------------MTQKENAYPWPYGRQT-----
gi|148225464|ref|NP_001085657.1|/1-368  ---------------------------------------MSYKENLIPSSCSSSSSSSS-
gi|146172296|ref|XP_001018388.2|/1-360  -------------MKTFDFNYNQSALQNQQKLKNGTSSQILKNIKDQENKNSSKDQENFK
gi|144226857|ref|NP_001016859.2|/1-360  ---------------------------------------MSYKENLNPSSYTS-------
gi|125524746|gb|EAY72860.1|/1-292       --------------------------------------------MAIATESRCEE-----
gi|115749607|ref|NP_898907.2|/1-344     ---------------------------------------MAQKENAYPWPYGRQT-----
gi|115455545|ref|NP_001051373.1|/1-309  ------------------------------------------------------------
gi|115435032|ref|NP_001042274.1|/1-292  --------------------------------------------MAIATESRCEE-----
gi|84000031|ref|NP_001033117.1|/1-402   ---------------MDRC-KENCISGP----KTAVPLSDGPKRVPVAQQFPSQN-----
gi|83776600|ref|NP_004208.2|/1-344      ---------------------------------------MAQKENSYPWPYGRQT-----
gi|76253851|ref|NP_695208.2|/1-397      ---------------MDRC-KENCVSRPV---KSTVPFG--PKRVLVTEQIPSQH-----
gi|68013217|ref|NP_001018849.1|/1-355   LNCLSVS-----------------------TPS---TTANPGRQQLLRLAVSNQRQVNNV
gi|66815573|ref|XP_641803.1|/1-384      -------------TTSTVKSTITKPTTTGATTSTNTSTIPPTTTASSSSSSSSSSS----
gi|62865639|ref|NP_001015878.1|/1-309   ------------------------------------------MSSPRAVVQLGKA-----
gi|62857967|ref|NP_001016570.1|/1-415   ---------------MERAVKENLPSNV----KVFHPTMEGPKRIPVTQPPSTQV-----
gi|57525577|ref|NP_001003640.1|/1-405   --------------MDSGARSKSSRDLKIHRPENEKVSAAGPKRVPVTQS-VQKPVSNP-
gi|50759065|ref|XP_425725.1|/1-409      ---------------MDRKTKENCPGNPSRATKVANPVVDGPKRVPVPQNSA-QS-----
gi|50303387|ref|XP_451635.1|/1-361      --------------------------MRPSLSRDASDTGRRHSLLQQSKLLSIRLSN---
gi|46358064|ref|NP_035627.1|/1-417      GCCKRIGKAVWRRGDMDRC-KENCVSRPV---KTTVPFG--PKRVLVTEQIPSQN-----
gi|45198420|ref|NP_985449.1|/1-367      --------------------------MDADAILKKDQ--RRSSLKQRNLLLSMRLNQTTA
gi|38327570|ref|NP_940838.1|/1-403      ---------------MDRS-KENCISGPV---KATAPVG-GPKRVLVTQQFPCQN-----
gi|17505246|ref|NP_491714.1|/1-305      -------------------------------------------MENKPPVINLP------
gi|17137514|ref|NP_477336.1|/1-329      ---------------------------------MTLSRAKHANRNHLPHLLAKVP-----
gi|17136516|ref|NP_476749.1|/1-411      --------------MSHPSDHVLRPKENAPHRMPEKSAAVHNMQKNLLLGKKPNSENMAP
gi|16758580|ref|NP_446201.1|/1-343      ---------------------------------------MAQKENVYPWPYGSKT-----
gi|15233958|ref|NP_195009.1|/1-294      --------------------------------------------MAIPTETQHQ------
gi|15225495|ref|NP_182073.1|/1-288      ------------------------------------------------------------
gi|6708213|gb|AAF25838.1|/1-282         ------------------------------------------------------------
gi|6325047|ref|NP_015115.1|/1-367       --------------------------MQRNSLVNIKLNANSPSKKTTTRPNTSRINK---
gi|2979623|gb|AAC12683.1|/1-345         ---------------------------------------MAQKENAYPWPYGSKT-----
```

```
XP_020952967.1/1-297 ---QPAV-----AAAGQTVS-----NIPT-------------------------------
XP_012380002.1/1-290 ----MAN-----TTRCLT------------------------------------------
XP_006102660.1/1-339 ---AQCGLNTLPQRVLQKDP-----TTPSSLVLMSRS-----------------------
XP_005673091.1/1-402 ---LPSANSGQAQRVLCPSNSSQRLPSHT--QKLVSSHKPVQN----LKQKQSQATSGPR
XP_004484680.1/1-405 ---PISASSGQAQRVLCSSNTSHRIPTQA--QKLVSSRKAVQI----QKQKQPQTTPVSH
XP_004462482.1/1-349 ---TQSGVNTLPQRVFRKEP-----ATPSALVLMSRSKAVLEPS----------------
Q6NW76.1/1-320 ----TPSAGVGPLRVEMNP----DTHAVSGPGRVPVK-----------------------
NP_999084.2/1-344 ---AQSGLNILPQRVLRKEA-----VTPSALVLMSRS-----------------------
gi|1190438012|ref|XP_020822491.1|/1-365 ---YHSSLNALPLRILRKEL-----STPSSTLLMNRSRAFLEPS----------------
gi|1190373986|ref|XP_020837071.1|/1-405 ---RLPASSGQAQRVLCSSNT-LLVSSQA--QKNVSSHKAIQT----QQPQKLQPAPVIH
gi|1154067554|ref|NP_001153906.2|/1-332 ----TPSMVTGPQRVLAKPRAELNKSTVTGPGRECVG-----------------------
gi|1131237831|ref|XP_019799799.1|/1-344 ---AQSGLNTLPQRVLRKEP-----VTPSALVLMSRS-----------------------
gi|1131178530|ref|XP_019784431.1|/1-296 ---QPA------AVAGQTMP-----SIPT-------------------------------
gi|1126479796|ref|XP_006940862.2|/1-308 ---DQGT-----AAAGLVEL-----GMPP-------------------------------
gi|1113784803|ref|XP_019353949.1|/1-376 ---NPGALGS---LAKYGAP-----GPQRVPRKETPAEALL--------------------
gi|1022987698|ref|XP_016286291.1|/1-365 ---PQSSLNTLPLNIFRKEI-----ATPSATLLMHRSRGLLELS----------------
gi|966994491|ref|XP_014974040.1|/1-344 ---APSGLSTLPQRVLRKEP-----VTPSALVLMSRS-----------------------
gi|966965902|ref|XP_015004225.1|/1-398 ---PLPANSGQAQRVLCPSNSSQRVPLQA--QKLVSSHKLVQN----QKQKQLQATSVPH
gi|955535802|ref|XP_004439167.2|/1-335 ---QSAG-----ETAGQTQP-----SFPT-------------------------------
gi|951053762|ref|XP_014465099.1|/1-416 ---RVPGGAAQAQRVLCPSNLAQRVPVPSQAPKTAASNQKLSHNQ--RMQQPQPTCVVQT
gi|946796851|ref|XP_014384184.1|/1-404 ---PLPANSGQAQRVLCPSNSSQQVPSKA--QKLVSSHKPVLN----LKQKQLQATSGPC
gi|946756071|ref|XP_014392719.1|/1-299 ---IIAE-----KKEPWPSS-----PSPG-------------------------------
gi|922383691|ref|XP_013462066.1|/1-300 -------QQHHTASSEVSGSAKD-------------------------------------
gi|909794683|ref|XP_013153467.1|/1-419 ---QLLNSGVQAQRVLCPSNFAQRVPVQS--QKPALSNQKLPNSQ--MTQQPRQKL-VQA
gi|891599507|ref|XP_013025412.1|/1-351 -YPVTKKDSRVLEGKRYSSSK---------------------------------------
gi|891565640|ref|XP_013019059.1|/1-351 -YPVTKKDSRGLENKRYSSSK---------------------------------------
gi|884853217|ref|XP_013010870.1|/1-315 ---PPSPLLQNCPQAEHQAP-----GFPA-------------------------------
gi|830244266|ref|XP_012590158.1|/1-296 ---QAAG------AAGGPAL-----PVAT-------------------------------
gi|828211890|ref|XP_012558808.1|/1-339 ---------------------------------------------------MNALKDNNF
gi|820033943|ref|NP_001295465.1|/1-282 -----------------MEP-----STST-------------------------------
gi|795377289|ref|XP_011750175.1|/1-471 ---QPAGEAVTAANQTAQQP-----STPA-------------------------------
gi|779989227|ref|XP_011666707.1|/1-321 ---ASSLNRG--------------------------------------------------
gi|761905244|ref|XP_011402440.1|/1-385 ---SYQLGSNYRLGQKTAPNLEPKVLCHP-LQESKTHQIPVSR------TGQTSATGQTA
gi|759175630|ref|XP_011377486.1|/1-303 ---QPT------AAEGQTLP-----GVPT-------------------------------
gi|759149200|ref|XP_011368486.1|/1-311 ---DFLLYVG--------------------------------------------------
gi|759116391|ref|XP_011357226.1|/1-339 ---AYSGLNTLPQRVLQKEP-----TTPSSLVLMNRS-----------------------
gi|758991346|ref|XP_957232.2|/1-420 TVPVATKSSTIVSSAAHKSTTYAGIRRVDDDDHDELS-----------------------
gi|743922878|ref|XP_011005517.1|/1-293 ----------QKARTDVSGAEK--------------------------------------
gi|698438703|ref|XP_009757361.1|/1-296 --------QQHKASPEASSEAD--------------------------------------
gi|697188794|ref|XP_009603442.1|/1-294 ---------QHKGSSEGAVVDN--------------------------------------
gi|658871623|ref|XP_008418750.1|/1-330 ----SPLSVTGPQRVLVKPKADAEKSYVAGPARERVG-----------------------
gi|657559162|ref|XP_008283516.1|/1-424 QYQRVLGVSNGPQRIQRPVSHQKPVSHVSVAAKSTHTADQNANPATQNVNPAPQPKSGSQ
gi|657529148|ref|XP_008293493.1|/1-319 ----TPSMVAGPQRVLVKPRVETIRNAVTGPGRECVS-----------------------
gi|655887082|ref|XP_008247187.1|/1-292 ---QSGS-----SQAAGPAQ-----PSVP-------------------------------
gi|655881324|ref|XP_008272369.1|/1-402 ---PVSACSGQAERVLCPSNSSQRVPSQA--QKLLPAEKPVQK------LKQAQAASVPR
gi|655868725|ref|XP_008268971.1|/1-343 ---PQSGLNTLPQRILRKEA-----ATPSALVLLNRS-----------------------
gi|641774253|ref|XP_008170712.1|/1-431 ---RVPGGGVQAQRILCPSNLAQRVPVQSRAQKSALSNQKPSQNQ--LMQQPHPTSLVQP
gi|620969612|ref|XP_007654691.1|/1-303 ------------------------------------------------------------
gi|617451788|ref|XP_007568855.1|/1-331 ----TPVSVTGPQRVLVKPKADAEKSYVAGPGRECVS-----------------------
gi|594674988|ref|XP_007185333.1|/1-402 ---PVSANSGQAQRVLCPSNSSQRLPSQV--QKLVSSQKPVQP----LKQKPAQAASAPR
gi|594654199|ref|XP_007175509.1|/1-282 -----------------MMP-----SIPT-------------------------------
gi|594623572|ref|XP_007166415.1|/1-344 ---AQSGLNTLPQRVLRKEP-----VTPSALVLMSRS-----------------------
gi|584038001|ref|XP_006753367.1|/1-368 ---VLAA-----DEAP-PLP-----SAPP-------------------------------
gi|573912300|ref|XP_006643864.1|/1-292 ----------EKAPPHSQEE----------------------------------------
gi|554569173|ref|XP_005878848.1|/1-339 ---AQCGLNTLPQRVLQKDP-----TTPSSLVLMSRS-----------------------
gi|545541126|ref|XP_005635254.1|/1-405 ---PLSANSGQAQRVLCPSNSSQRVPPQT--QKLVSSHKPAQN----LKQKQLQATGVPR
gi|545487136|ref|XP_005616134.1|/1-307 ---EQSK-----DAAGHVEL-----SVPP-------------------------------
gi|544514002|ref|XP_005590555.1|/1-471 ---QPAGEAVTAANQTAQQP-----SSPA-------------------------------
gi|530653727|ref|XP_005313062.1|/1-365 ---MPSGTPSESLLAQRAMA-----STNKIILKSS-------------------------
gi|528993703|ref|XP_005219854.1|/1-303 ---QHAA------AAGQTVP-----GAPT-------------------------------
gi|524890835|ref|XP_005101959.1|/1-332 ---LTSGTTAAHVDSLAAAKLPLRVPAEK-------------------------------
gi|514776534|ref|XP_004968502.1|/1-292 ----------DKASAHANEE----------------------------------------
gi|507960369|ref|XP_004686961.1|/1-405 ---ALPASSGQAQRVLCPSNS-QRLPSQA--QKLVSSHKAVPNPN--LKQKQLQANNAPR
gi|507953708|ref|XP_004684672.1|/1-344 ---AQSGLNTLPQRVLRKDP-----TTPSALVLMNRS-----------------------
gi|478517838|ref|XP_004433150.1|/1-344 ---AQSGLNTLPQRVLRKEP-----VTPSALVLMSRS-----------------------
gi|478512086|ref|XP_004430306.1|/1-405 ---PLSANSGQAQRVLCPSNSSHRAPTQA--QKLVSSHRPVQN----LKQKQSQATSVPP
gi|470628341|ref|XP_004321082.1|/1-402 ---PVSANSGQAQRVLCPSNSSQRVPSQV--QKLVSSQKSVQP----LKQKPTQAASAPR
```

gi|470261727|ref|XP_004360485.1|/1-457 -------SSSASVSTQQQQSTRQQMMQQKQLLQEQKQQQYQED-----------------
gi|449486478|ref|XP_002195961.2|/1-319 --RPRR------
gi|426243291|ref|XP_004015492.1|/1-303 ---QPAA------AAGQSVP-----GAPA-------------------------------
gi|426237569|ref|XP_004012730.1|/1-344 ---AQPGLNTLPQRVLRKEA-----VIPSALVLMSRS-----------------------
gi|410979749|ref|XP_003996244.1|/1-344 ---TQPGLNTLPQRVLRKEP-----ATPSALVLMSRS-----------------------
gi|410953494|ref|XP_003983405.1|/1-405 ---LLSANSGQAQRVLCPSNSSQRIPSQT--QKLVSSHKPVQN----LKQKQLQATSVPR
gi|410914403|ref|XP_003970677.1|/1-332 ----APMTLGGPQRVQVKPRAEMDRSAITGPGRECVP-----------------------
gi|389641901|ref|XP_003718583.1|/1-397 SVTLPSQAAQRKMAPPPKSREPPSPSKHKKD-----------------------------
gi|389635857|ref|XP_003715581.1|/1-400 QASLESQIREFLHAKKPREQG-AGKEPLEQ------------------------------
gi|357126095|ref|XP_003564724.1|/1-291 ----------EKAAQHSQEE--
gi|355703962|gb|EHH30453.1|/1-309 ---QPAGEAVTAANQTAQQP-----SSPA-------------------------------
gi|350538969|ref|NP_001233545.1|/1-403 ---PLPVNSGQAQRVLCPSNSSQRVPLQA--QKLVSSHKPVQN----QKQKQLQATSVPH
gi|347966592|ref|XP_321274.5|/1-404 AANTAAGRSNNAAKMVGKVAEGGGQHMGG--FKAPQLPAPVVRPAASSSKPVPEKQIT-P
gi|345800233|ref|XP_849906.2|/1-344 ---TQPGLNTLPQRVLRKDP-----ATPSALVLMSRS-----------------------
gi|344296517|ref|XP_003419953.1|/1-405 ---PLSASGGQAQRVLCPSNSSQSIPSQA--QKLVSSRKPVQS----QKQKPLQITTVPR
gi|344290468|ref|XP_003416960.1|/1-345 ---AQSGLNTLPQRVFRKDL-----ATPSALVLMSRS-----------------------
gi|344269722|ref|XP_003406697.1|/1-306 ---ELAP-----TERALYGD-----VLLE-------------------------------
gi|338709975|ref|XP_001502047.3|/1-304 ---RPAV-----PTAGQSQP-----GFPN-------------------------------
gi|334312551|ref|XP_001378079.2|/1-402 ---RLPGSSGQAQRVLCSSNP-LLVSSQA--QKNISSHKTSHA----HQSQKLQQAPVIH
gi|332848444|ref|XP_511856.3|/1-344 ---APSGLSTLPQRVLRKEP-----VTPSALVLMSRC-----------------------
gi|330793523|ref|XP_003284833.1|/1-359 -------STPTSTSTSSSNSG---------------------P-----------------
gi|321461440|gb|EFX72472.1|/1-304 ------------QPTVKLEN--
gi|308497987|ref|XP_003111180.1|/1-306 ----------EKKSKNNPMK--
gi|302801442|ref|XP_002982477.1|/1-288 -----------VEAPEAVPE--
gi|302688103|ref|XP_003033731.1|/1-328 --RSSSPSTRAWQSACPTCCA---------------------------------------
gi|297802712|ref|XP_002869240.1|/1-290 ----------EKEASDA-----A-------------------------------------
gi|281427796|ref|NP_001164000.1|/1-322 VCQNKENN--------------------------------VPRAAAKKGPPAQKG-----
gi|268565413|ref|XP_002639437.1|/1-302 ------------KSKNTPNK--
gi|260784959|ref|XP_002587531.1|/1-282 --
gi|226499040|ref|NP_001148791.1|/1-296 ----------DRASAHANQE--
gi|224088986|ref|XP_002308590.1|/1-293 ----------EKASTEVSGEEK--------------------------------------
gi|215983064|ref|NP_001135979.1|/1-405 ---PVSVNSGQAQRVLCPTNSSQRVPSQA--QKLVSIQKPVQT----LKQKPPQAASAPR
gi|213404608|ref|XP_002173076.1|/1-346 -----NNSSTSADALRERQER---------------------------------------
gi|198430178|ref|XP_002119314.1|/1-305 --
gi|196012778|ref|XP_002116251.1|/1-270 --
gi|168044897|ref|XP_001774916.1|/1-297 -----------QESQGAGGDSIA--------------------------------------
gi|159468203|ref|XP_001692272.1|/1-292 ----------QAAGTSGGPK--
gi|158297402|ref|XP_317640.3|/1-307 -AHSEVVLQ--TIKMMQHPAYEN-------------------------------------
gi|156368106|ref|XP_001627537.1|/1-314 --
gi|149734120|ref|XP_001489320.1|/1-404 ---LLSANSGQAQRVLCPSNSSHRVPSQA--QKLVSSHKPVQN----LKQKQSQATSVPH
gi|149724949|ref|XP_001504864.1|/1-344 ---AQSGLNTLPQRVLRKEP-----ATPSALVLMSRS-----------------------
gi|148225464|ref|NP_001085657.1|/1-368 -KFATPSSATAAQRVLRKQPYVSIFTTPSDNLLAQRAQLAPRVT----------------
gi|146172296|ref|XP_001018388.2|/1-360 NSQLLSVKSIKKIEQIEQQSDRVQILKVP-------------------------------
gi|144226857|ref|NP_001016859.2|/1-360 -KFATPSSATAAQRVLRKEPYVSTFTTPSDNLLAQRAQLA-RIT----------------
gi|125524746|gb|EAY72860.1|/1-292 ----------EKASPHSQEV--
gi|115749607|ref|NP_898907.2|/1-344 ---AQPGLNTLPQRVLRKEP-----VTPSALVLMSRS-----------------------
gi|115455545|ref|NP_001051373.1|/1-309 ---------------MEKP---
gi|115435032|ref|NP_001042274.1|/1-292 ----------EKASPHSQEV--
gi|84000031|ref|NP_001033117.1|/1-402 ---PVSVNSGQAQRVLCPTNSSQRVPSQA--QKLVSIQKPVQT----LKQKPPQAASAPR
gi|83776600|ref|NP_004208.2|/1-344 ---APSGLSTLPQRVLRKEP-----VTPSALVLMSRS-----------------------
gi|76253851|ref|NP_695208.2|/1-397 ---PGSASSGQAQRVLCPSNS-QRVPPQA--QKPVAGQKPVLK--------QLPAASGPR
gi|68013217|ref|NP_001018849.1|/1-355 SLANGKENKRTSNSKFNSSLR---------------------------------------
gi|66815573|ref|XP_641803.1|/1-384 -------SSSSSSSSSSSSSSQS------------------VP-----------------
gi|62865639|ref|NP_001015878.1|/1-309 ---QPAGEELATANQTAQQP-----SSPA-------------------------------
gi|62857967|ref|NP_001016570.1|/1-415 ---RPPGTAVSAQRILGPSNVPQRVVSQAQ-KPVMSAQKPVMSTQKPMTQGPLRPVTHGH
gi|57525577|ref|NP_001003640.1|/1-405 -HTRVLGAAQGPQRVQRPVG------------KTSCPGDQNTRPEQH--KPAAHSKP---
gi|50759065|ref|XP_425725.1|/1-409 ---RPLNSGVQPQRVLCPSNFAQRVPVQS--QKSSLSNQKLSNNQ--TTQQPRPKLPVQP
gi|50303387|ref|XP_451635.1|/1-361 ---AGKRPSLSRGHAHEKDSI-TSSHHRLSTGPVNNN-----------------------
gi|46358064|ref|NP_035627.1|/1-417 ---LGSASSGQAQRVLCPSNS-QRVPSQA--QKLGAGQKPAPK--------QLPAASVPR
gi|45198420|ref|NP_985449.1|/1-367 TNGAPPQARVQPGKGYRNPGK-VLSPIRNQEMSPGKR-----------------------
gi|38327570|ref|NP_940838.1|/1-403 ---PLPVNSGQAQRVLCPSNSSQRIPLQA--QKLVSSHKPVQN----QKQKQLQATSVPH
gi|17505246|ref|NP_491714.1|/1-305 ----------EKETVNTPQK--
gi|17137514|ref|NP_477336.1|/1-329 -EEHQEPIKNMCLKMMSHDAYGQ-------------------------------------
gi|17136516|ref|NP_476749.1|/1-411 ASKPLPGSSGALIRKPGLGGSNSIASSEGNNFQKPMVPS-VKKTTSEFAAPAPVAPIKKP
gi|16758580|ref|NP_446201.1|/1-343 ---SQSGLNTLPQRVLRKEP-----AVTPAQALMNRS-----------------------
gi|15233958|ref|NP_195009.1|/1-294 ----------EKEASDAS-AAAA-------------------------------------
gi|15225495|ref|NP_182073.1|/1-288 ---------------MSKKSTESDAGNT--------------------------------
gi|6708213|gb|AAF25838.1|/1-282 -----------------MEP-----STST-------------------------------
gi|6325047|ref|NP_015115.1|/1-367 ----PWRISHSPQQRNPNSKIPSPVREKLNRLPVNNK-----------------------
gi|2979623|gb|AAC12683.1|/1-345 ---SQSGLNTLSQRVLRKEP-----ATTSALALVNRS-----------------------

```
XP_020952967.1/1-297                      -----------------------------------------------GRRLTIDDFEIGR
XP_012380002.1/1-290                      ----------------------------------------------------IDDFEIGR
XP_006102660.1/1-339                      -------NAQPTAAPGQ-----NSNGTPNL-----------------MRSFTIDDFEIGR
XP_005673091.1/1-402                      PVSRPL-SNTQQSEQPQPAAPGNNPEKEAASKQK--------NEESKKRQWALEDFEIGR
XP_004484680.1/1-405                      PVSKPL-NNTQKSEQSQPPASGSNSGKELESKQK--------KEESKKRQWALEDFEIGR
XP_004462482.1/1-349                      -------NAQPTA--GQK-TVENSSGTPSFL----------------MRSFTIEDFEIGR
Q6NW76.1/1-320                            -----------------------------SNS----------------KVLSIDDFDIGR
NP_999084.2/1-344                         -------NTQPTAAPGQK-VVENSSGTPNFS----------------TRSFTIDDFEIGR
gi|1190438012|ref|XP_020822491.1|/1-365 -------STQPAAVPGRI-GVDSSNMKLNLP----------------VHQLKIDDFEIGR
gi|1190373986|ref|XP_020837071.1|/1-405 PASRPP-KSTLNTEQSQTPVSASTPGKGETPRQK--------NEETKKRQWALDDFEIGR
gi|1154067554|ref|NP_001153906.2|/1-332 -----------------------------TSSVSP------------PKKISIDDFDIGR
gi|1131237831|ref|XP_019799799.1|/1-344 -------NAQPTAAPLQK-VVENNSRTPNFS----------------MRSFTIDDFEIGR
gi|1131178530|ref|XP_019784431.1|/1-296 -----------------------------------------------VRRLTIDDFEIGR
gi|1126479796|ref|XP_006940862.2|/1-308 -----------------------------------------------VRRLTIEDFEIGR
gi|1113784803|ref|XP_019353949.1|/1-376 -------AQRATAVPGRVPVEA--GAQVAPQPP--------------LKTLSIEDFEIGR
gi|1022987698|ref|XP_016286291.1|/1-365 -------GAQPAALPGRI-GLDNSNVKSNQT----------------VRHLKIEDFEIGR
gi|966994491|ref|XP_014974040.1|/1-344  -------NVQPTAAPGQK-VVENSSGTPNIL----------------MRHFTIDDFEIGR
gi|966965902|ref|XP_015004225.1|/1-398  PVSRPP-SHTPKSKQPLPSAPENNPEEELASKQK--------NEESKKRQWALEDFEIGR
gi|955535802|ref|XP_004439167.2|/1-335  -----------------------------------------------VRHFTIDDFEIGR
gi|951053762|ref|XP_014465099.1|/1-416  SSRPQA-PN-NNEKPHHVPVSANQPEKEGTSKPK--------NQETKKRQWSLDDFEIGR
gi|946796851|ref|XP_014384184.1|/1-404  PVSRPL-NNTQKSEQPQPPAPGKNSEKEVASKQK--------NEESKK-QWTLEDFEIGR
gi|946756071|ref|XP_014392719.1|/1-299  -----------------------------------------------SRSLTIDDFDIRC
gi|922383691|ref|XP_013462066.1|/1-300  -----------------------------------------------QRRWILNDFDIGK
gi|909794683|ref|XP_013153467.1|/1-419  TARSQV-PTKNNEKPQQVPVPAKNPEAESTSKQKNEETAKKKNEETKKRQWSLDDFEIGR
gi|891599507|ref|XP_013025412.1|/1-351  -------------QKDEPIAGVPSPMGPQ------------------WRDFHIGMFEIGK
gi|891565640|ref|XP_013019059.1|/1-351  -------------QQDEPIAGVPSPMGPQ------------------WRDFHIGMFEIGK
gi|884853217|ref|XP_013010870.1|/1-315  -----------------------------------------------RRPFTIEDFDIGC
gi|830244266|ref|XP_012590158.1|/1-296  -----------------------------------------------VRRLTIDDFEIGR
gi|828211890|ref|XP_012558808.1|/1-339  QNSSKGTVKTMLQKSDKPSSLEKENQDQKEPKNQN----KENPPPPIDKCWSLSDFDIGK
gi|820033943|ref|NP_001295465.1|/1-282  -----------------------------------------------KKHFTINDFEIGR
gi|795377289|ref|XP_011750175.1|/1-471  -----------------------------------------------MRRLKIDDFEIGR
gi|779989227|ref|XP_011666707.1|/1-321  ------------TTAQARTQPVKSAENGHGHDDK---------QMEPKKSWTLKDFDIGR
gi|761905244|ref|XP_011402440.1|/1-385  HGTREHLTVPPSAPPPVSSSLGEAPQGGAGTATDQ-------SQQPVSKKWGLSDFDIGR
gi|759175630|ref|XP_011377486.1|/1-303  -----------------------------------------------TRQLTIDDFEIGR
gi|759149200|ref|XP_011368486.1|/1-311  ----------------------NNSEKELASKQK--------NEESKKRQWALEDFEIGR
gi|759116391|ref|XP_011357226.1|/1-339  -------NAQTTAAPGQ-----NSSGTPNL-----------------MRSFTIDDFEIGR
gi|758991346|ref|XP_957232.2|/1-420    -------QPMASASVSSTGTSTSESSSSAGYEQSI-----------SPKQFHLGMFEIGR
gi|743922878|ref|XP_011005517.1|/1-293  ------------------------------------------------KRWTLNDFDIGK
gi|698438703|ref|XP_009757361.1|/1-296  -----------------------------------------------KKRWMLNDFDIGK
gi|697188794|ref|XP_009603442.1|/1-294  ------------------------------------------------KRWTVNDFDIGK
gi|658871623|ref|XP_008418750.1|/1-330  -----------------------------SSSNPP------------PKKFTIDDFDIGR
gi|657559162|ref|XP_008283516.1|/1-424  QNQPKTHVPTVKPELTTPASESAKLEKPQNKPAKN----DAANASSSKKPWSLENFDIGR
gi|657529148|ref|XP_008293493.1|/1-319  -----------------------------AASSSV------------SKKISIDDFDIGR
gi|655887082|ref|XP_008247187.1|/1-292  -----------------------------------------------LRRLTIEDFEIGR
gi|655881324|ref|XP_008272369.1|/1-402  PVSKPP-NNTPKSEQPPPSAPGNNPEKELTSNQK--------NEESKKRQWTLEDFDIGR
gi|655868725|ref|XP_008268971.1|/1-343  -------NAPPTAVLGQK-GMENSSGAPNF-----------------TRPFTIDDFEIGR
gi|641774253|ref|XP_008170712.1|/1-431  ISRPQA-PSKNNEEPPQTSLSAKNLEAEGTSVQK--------NEETKKRQWCLDDFEIGR
gi|620969612|ref|XP_007654691.1|/1-303  ----------------NIPEKREVPSQKEAPCQK--------NEESKKRQWSLDDFDIGR
gi|617451788|ref|XP_007568855.1|/1-331  -----------------------------SSSNSP------------PKKFTIDDFDIGR
gi|594674988|ref|XP_007185333.1|/1-402  AVSRPL-SNTQKSEQPQLPAPGNNPEKEAASKQK--------NEESKRRQWALEDFEIGR
gi|594654199|ref|XP_007175509.1|/1-282  -----------------------------------------------MRRLTIDDFEIGR
gi|594623572|ref|XP_007166415.1|/1-344  -------NAQPTAAPLQK-VVENNSGTPNFS----------------MRSFTIDDFEIGR
gi|584038001|ref|XP_006753367.1|/1-368  -----------------------------------------------VRSLTIDDFDIGR
gi|573912300|ref|XP_006643864.1|/1-292  ------------------------------------------------KRWVLSDFDIGK
gi|554569173|ref|XP_005878848.1|/1-339  -------NAQPTAAPGQ-----NSNGTPNL-----------------MRSFTIDDFEIGR
gi|545541126|ref|XP_005635254.1|/1-405  PASRSL-NNTQKSEQPSSSAPGNNSEKELATKQK--------NEESKKRQWALEDFEIGR
gi|545487136|ref|XP_005616134.1|/1-307  -----------------------------------------------GRRLTIDDFEIGR
gi|544514002|ref|XP_005590555.1|/1-471  -----------------------------------------------MRRLKIDDFEIGR
gi|530653727|ref|XP_005313062.1|/1-365  -------SIQKPAVPGRVPAELSSGAQAAQPTP--------------QRIFTINDFEIGR
gi|528993703|ref|XP_005219854.1|/1-303  -----------------------------------------------ARRFTVDDFEIGR
gi|524890835|ref|XP_005101959.1|/1-332  --------------------ENNSSGGDIASSSD---------SSNGPVKWGLSNFDIGR
gi|514776534|ref|XP_004968502.1|/1-292  ------------------------------------------------KRWVLSDFEVGK
gi|507960369|ref|XP_004686961.1|/1-405  AAPRPL-AGTPKSEPP-PSAPGNNSEKEQASKQK--------TEESKKRQWALEDFEIGR
gi|507953708|ref|XP_004684672.1|/1-344  -------NTQPTAAPGQK-VVENSSGTPNLS----------------MRSFTIDDFEIGR
gi|478517838|ref|XP_004433150.1|/1-344  -------NAQPTAALGQK-VVENSSGTPNLL----------------MRSFTIDDFEIGR
gi|478512086|ref|XP_004430306.1|/1-405  PVSRPL-NSTQKSEQPQPSAPGNNSEKERTSKEK--------NEDSKRRQWALEDFEIGR
gi|470628341|ref|XP_004321082.1|/1-402  SVSRPL-SNTQKSEQPQLPAPGNNPEKEAASKQK--------NEESKRRQWALEDFEIGR
```

```
gi|470261727|ref|XP_004360485.1|/1-457  -----------------------------------------------KKKWTIDDFDIGK
gi|449486478|ref|XP_002195961.2|/1-319  --------------------QAQNPEAESTSKQKTEET-KKKSEETKKRQWSLDDFEIGR
gi|426243291|ref|XP_004015492.1|/1-303  -----------------------------------------------ARRFTIDDFEIGR
gi|426237569|ref|XP_004012730.1|/1-344  -------NAQPTAAPGQK-VVENSSGTLNIP----------------KRSFTIDDFEIGR
gi|410979749|ref|XP_003996244.1|/1-344  -------NTQPTAAPGQK-VVENSSGTPNFS----------------MRSFTIDDFEIGR
gi|410953494|ref|XP_003983405.1|/1-405  SVSRPL-NNTQKSEQPLPSATGNNSEKELTTKQK--------NEESKKRQWALEDFEIGR
gi|410914403|ref|XP_003970677.1|/1-332  -----------------------------SSSTSS------------TRKITIDDFDIGR
gi|389641901|ref|XP_003718583.1|/1-397  ---------SVSSSVDELANEEVEASAPR-----------------VPKEFHLGMFEIGR
gi|389635857|ref|XP_003715581.1|/1-400  ---------LQAAQAAAAAAALVAQPSAQ------------------PVAMHLGMFEIGK
gi|357126095|ref|XP_003564724.1|/1-291  ------------------------------------------------KRWVLSDFEVGK
gi|355703962|gb|EHH30453.1|/1-309       -----------------------------------------------MRRLKIDDFEIGR
gi|350538969|ref|NP_001233545.1|/1-403  PVSRPL-NNTQKSKQPLPSAPENNPEEELASKQK--------NEESKKRQWALEDFEIGR
gi|347966592|ref|XP_321274.5|/1-404     KTESEPMDTTPADDRPD--AAEQKQSGTDGNQQAQ-------QAKPAKKVWTLSNFDIGR
gi|345800233|ref|XP_849906.2|/1-344     -------NGQPTAAPGQK-VVENSSGIPNFS----------------MRSFTIDDFEIGR
gi|344296517|ref|XP_003419953.1|/1-405  PVSRPP-NNTPKGEQAQPPASGNNSGKELASKQK--------KEESKRRQWALEDFEIGR
gi|344290468|ref|XP_003416960.1|/1-345  -------NAQPTAAPGEKKMTENSSGTPSFL----------------MRPFTIDDFEIGR
gi|344269722|ref|XP_003406697.1|/1-306  -----------------------------------------------MRRLRIDDFEIGR
gi|338709975|ref|XP_001502047.3|/1-304  -----------------------------------------------VRHLTIDDFEIGR
gi|334312551|ref|XP_001378079.2|/1-402  PTSRPP-SSTQNTEQSQPPVSASIPGKGETPRQK--------NEETKKRQWALDDFEIGR
gi|332848444|ref|XP_511856.3|/1-344     -------NVQPTAAPGQK-VMENSSGTPNIL----------------TRHFTIDDFEIGR
gi|330793523|ref|XP_003284833.1|/1-359  -----------------------------------------------KKKWCIDDFDIGK
gi|321461440|gb|EFX72472.1|/1-304       ------------------------------------------------SEWSLDNFEIGR
gi|308497987|ref|XP_003111180.1|/1-306  -----------------------------------------------GGKLSIKDFEIGR
gi|302801442|ref|XP_002982477.1|/1-288  -----------------------------------------------ERRWKLADFDIGK
gi|302688103|ref|XP_003033731.1|/1-328  ---------------PARILTPVSRRYPT-------------------REWTLHDFDIGR
gi|297802712|ref|XP_002869240.1|/1-290  -----------------------------------------------QKRWTLSDFDIGK
gi|281427796|ref|NP_001164000.1|/1-322  -ALSENK--------------AQKGAASDTAQESS-------KGRR----WTLADFDIGK
gi|268565413|ref|XP_002639437.1|/1-302  -----------------------------------------------GGKLSINDFEIGR
gi|260784959|ref|XP_002587531.1|/1-282  ---------------------------------------------MKS--WKLEDFDIGR
gi|226499040|ref|NP_001148791.1|/1-296  ------------------------------------------------KRWVLSDFEVGK
gi|224088986|ref|XP_002308590.1|/1-293  ------------------------------------------------KRWTLNDFDIGK
gi|215983064|ref|NP_001135979.1|/1-405  PVARPP-SNTQKSKQPQPPAPGNNPEKEVASKQK--------NEESKKRQWALEDFEIGR
gi|213404608|ref|XP_002173076.1|/1-346  -------------HYSLSSDGNNNSSKP-------------------LRNFHLGMFEIGK
gi|198430178|ref|XP_002119314.1|/1-305  --------------MSSANNENKAPEKASTSGRAT----ASSAKDNASDTWSLKNFDIGK
gi|196012778|ref|XP_002116251.1|/1-270  -----------------------------------------------RKRWTLDDFDIGR
gi|168044897|ref|XP_001774916.1|/1-297  -----------------------------------------------EKRWSLTDFDIGK
gi|159468203|ref|XP_001692272.1|/1-292  ------------------------------------------------RRWTIDDFDIGK
gi|158297402|ref|XP_317640.3|/1-307     -----------------------------------------------PYEWSTDDFEVGR
gi|156368106|ref|XP_001627537.1|/1-314  -MAKIPSNTHQTTVSSFNPSITKHMKGSKGGPFK-------LLENMKQGKWNLSDFDIGK
gi|149734120|ref|XP_001489320.1|/1-404  PVPRPL-NNTQRSEQLQPSAPGNNSEKELASKEK--------NEESKKRQWALEDFEIGR
gi|149724949|ref|XP_001504864.1|/1-344  -------NAQPTVALGQK-VMENSSGTPNLL----------------MRSFTIDDFEIGR
gi|148225464|ref|NP_001085657.1|/1-368  -------PAASSSVPGRVALGTDVASHNTALAEAP------------KRKFTIDDFDIGR
gi|146172296|ref|XP_001018388.2|/1-360  -----------------------------------------------KIQWQLSDFELGK
gi|144226857|ref|NP_001016859.2|/1-360  -------PSASSSVPGRVAVSMDASSQNTALAELP------------KRKFTIDDFDIGR
gi|125524746|gb|EAY72860.1|/1-292       ------------------------------------------------KRWVLSDFDIGK
gi|115749607|ref|NP_898907.2|/1-344     -------NAQPTAAPGQK-VVENSSGTPNIP----------------KRSFTIDDFEIGR
gi|115455545|ref|NP_001051373.1|/1-309  -------------------------------------------------EWSMDDFEIGK
gi|115435032|ref|NP_001042274.1|/1-292  ------------------------------------------------KRWVLSDFDIGK
gi|84000031|ref|NP_001033117.1|/1-402   PVTRPP-SNTQKSKQPQPPAPGNNPEKEVASKQK--------NEESKKRQWALEDFEIGR
gi|83776600|ref|NP_004208.2|/1-344      -------NVQPTAAPGQK-VMENSSGTPDIL----------------TRHFTIDDFEIGR
gi|76253851|ref|NP_695208.2|/1-397      PASR-L-SNPQKSEQPQPAASGNNSEKEQTSIQK--------TEDSKKRQWTLEDFDIGR
gi|68013217|ref|NP_001018849.1|/1-355   -------------KIEEPIAGVPSSAGPQ------------------WREFHIGMFEIGK
gi|66815573|ref|XP_641803.1|/1-384      -----------------------------------------------KKKWCIDDFDIGK
gi|62865639|ref|NP_001015878.1|/1-309   -----------------------------------------------MRRLTVDDFEIGR
gi|62857967|ref|NP_001016570.1|/1-415   QAGKPQGPNENRTPQQTSHSSTPNVEKKGSTDQG--KTLAVPKEEGKKKQWCLEDFEIGR
gi|57525577|ref|NP_001003640.1|/1-405   QPKPPLSAETNKTAEPSKQDKPHQTPSSTTSTSSN----TSGSSKSSKKAWTLENFDIGR
gi|50759065|ref|XP_425725.1|/1-409      TAGPQV-PSKNSEKPQQAPVPAKSSEADSVSKQKNEESTKKKKEETKKRQWSLDDFEIGR
gi|50303387|ref|XP_451635.1|/1-361      -------PRRSLMVKK----PNTAHGT-RVPILND--------HNDKFENMTLQDFEIGK
gi|46358064|ref|NP_035627.1|/1-417      PVSR-L-NNPQKNEQ--PAASGNDSEKEQASLQK--------TEDTKKRQWTLEDFDIGR
gi|45198420|ref|NP_985449.1|/1-367      -------PTLEISELKNVSPINQAHKTGKARAGGV--------PLSKLQNLKLADFEIGK
gi|38327570|ref|NP_940838.1|/1-403      PVSRPL-NNTQKSKQPLPSAPENNPEEELASKQK--------NEESKKRQWALEDFEIGR
gi|17505246|ref|NP_491714.1|/1-305      -----------------------------------------------GGKFTINDFEIGR
gi|17137514|ref|NP_477336.1|/1-329      -----------------------------------------------PYDWSPRDFEMGA
gi|17136516|ref|NP_476749.1|/1-411      ESLSKQKPTAASSESSKELGAASSSAEKEKTKTET-------QPQKPKKTWELNNFDIGR
gi|16758580|ref|NP_446201.1|/1-343      -------NSQSTAVPGQKLTENK--GATALQGSQS------------RQPFTIDNFEIGR
gi|15233958|ref|NP_195009.1|/1-294      -----------------------------------------------QKRWTLSDFDIGK
gi|15225495|ref|NP_182073.1|/1-288      -----------------------------------------------EKQWSLADFEIGR
gi|6708213|gb|AAF25838.1|/1-282         -----------------------------------------------RKHFTINDFEIGR
gi|6325047|ref|NP_015115.1|/1-367       -------KFLDMESSKIPSPIRKATSS-KMIHENK--------KLPKFKSLSLDDFELGK
gi|2979623|gb|AAC12683.1|/1-345         -------NSQSTAAPGQKLAENKSQGSTASQGSQN------------KQPFTIDNFEIGR
```

```
                                                                                     *::

XP_020952967.1/1-297                   PLGKGKFGNVYLARLKES-HFIVALKVLFKSQIEKEG-LEHQLRREIEIQAHLQ------
XP_012380002.1/1-290                   PLGKGKFGSVYLARLKEN-HFIVALKILFKSQIEKEG-LEHQLRREIEIQAHLQEEPDHL
XP_006102660.1/1-339                   PLGKGKFGNVYLAREKKS-RFIVALKVLFKSQIEKEG-VEHQLRREIEIQAHLQ------
XP_005673091.1/1-402                   PLGKGKFGNVYLAREKQS-KFILALKVLFKTQLEKAG-VEHQLRREVEIQSHLR------
XP_004484680.1/1-405                   PLGKGKFGNVYLAREKQS-KFILALKVLFKAQLEKAG-VEHQVRREVEIQSHLR------
XP_004462482.1/1-349                   PLGKGKFGNVYLAREKKS-HFIVALKVLFKSQIEKEG-VEHQLRREIEIQAHLQ------
Q6NW76.1/1-320                         PLGKGKFGNVYLARERKL-KVVIALKVLFKSQMVKEG-VEHQLRREIEIQSHLR------
NP_999084.2/1-344                      PLGKGKFGNVYLAREKKS-HFIVALKVLFKSQIEKEG-VEHQLRREIEIQAHLQ------
gi|1190438012|ref|XP_020822491.1|/1-365 PLGKGKFGNVYLAREKNS-QFIVALKVLFKSQMEKEG-VEHQLRREIEIQSHLR------
gi|1190373986|ref|XP_020837071.1|/1-405 PLGKGKFGNVYLAREKQS-KFILALKVLFKAQLEKAG-VEHQLRREVEIQSHLR------
gi|1154067554|ref|NP_001153906.2|/1-332 PLGKGKFGNVYLARVKKL-QAIVALKVLFKSQMEKEG-VEHQLRREIEIQAHLK------
gi|1131237831|ref|XP_019799799.1|/1-344 PLGKGKFGNVYLAREKKS-HFIVALKVLFKSQIEKEG-VEHQLRREIEIQAHLQ------
gi|1131178530|ref|XP_019784431.1|/1-296 PLGKGKFGNVYLARLKES-HFIVALKVLFKSQIEKEG-LEHQLRREIEIQAHLQ------
gi|1126479796|ref|XP_006940862.2|/1-308 PLGKGKFGNVYLARLKES-HFLVALKVIFKSQIEKEG-LEHQLRREIEIQAHLQ------
gi|1113784803|ref|XP_019353949.1|/1-376 PLGKGKFGNVYLAREKGS-HFLVALKVLFKSQMEKEG-VEHQLRREIEIQAHLR------
gi|1022987698|ref|XP_016286291.1|/1-365 PLGKGKFGNVYLAREKNS-QFIVALKVLFKSQMEKEG-VEHQLRREIEIQSHLR------
gi|966994491|ref|XP_014974040.1|/1-344  PLGKGKFGNVYLAREKKS-HFIVALKVLFKSQIEKEG-VEHQLRREIEIQAHLH------
gi|966965902|ref|XP_015004225.1|/1-398  PLGKGKFGNVYLAREKQS-KFILALKVLFKAQLEKAG-VEHQLRREVEIQSHLR------
gi|955535802|ref|XP_004439167.2|/1-335  PLGKGKFGNVYLARLKES-HFIVALKVLFKSQIEKEG-LEHQLRREIEIQAHLQ------
gi|951053762|ref|XP_014465099.1|/1-416  PLGKGKFGNVYLAREKQS-KFILALKVLFKSQLEEAG-VEHQLRREVEIQSHLR------
gi|946796851|ref|XP_014384184.1|/1-404  PLGKGKFGNVYLAREKQS-KFILALKVLFKAQLEKAG-VEHQIRREVEIQSHLR------
gi|946756071|ref|XP_014392719.1|/1-299  PLGKGKFGNVYLARLRDS-HFIVALKVLFKSQLEKEG-MEHQLRREIEIQSHLQ------
gi|922383691|ref|XP_013462066.1|/1-300  PLGRGKFGHVYLAREKTS-NHIVALKVLFKSQLQQSQ-VEHQLRREVEIQSHLR------
gi|909794683|ref|XP_013153467.1|/1-419  PLGKGKFGNVYLAREKQS-KFILALKVLFKTQLEEAG-VEHQLRREVEIQSHLR------
gi|891599507|ref|XP_013025412.1|/1-351  PLGKGKFGRVYLAKEKKT-GFVVALKTLHKSELIHSR-IEKQVRREIEIQSNLR------
gi|891565640|ref|XP_013019059.1|/1-351  PLGKGKFGRVYLAKEKKT-GFIVALKTLHKSELIHSR-IEKQVRREIEIQSNLR------
gi|884853217|ref|XP_013010870.1|/1-315  PLGKGKFGSVYLARLKQS-HFIVALKVLFKSQVEKEG-MEHQLRREVEIQAHLH------
gi|830244266|ref|XP_012590158.1|/1-296  PLGKGKFGNVYLARLRES-RFLVALKVLFKSQIEKEG-LEHQLRREVEIQAHLR------
gi|828211890|ref|XP_012558808.1|/1-339  PLGKGKFGSVYLAREKQS-HFIVALKVLFKSQLMKAA-VEHQLRREIEIQSHLK------
gi|820033943|ref|NP_001295465.1|/1-282  PLGRGKFGRVYLARLKEN-HFIVALKVLFKSEIGKEG-LEHQLRREVEIQSHLQ------
gi|795377289|ref|XP_011750175.1|/1-471  PLGKGKFGNVYLARLKES-HFIVALKVLFKSQIEKEG-LEHQLRREIEIQAHLQ------
gi|779989227|ref|XP_011666707.1|/1-321  PLGKGKFGSVYLAREKQT-KYIVALKVLFKSQLQKAQ-VEHQLRREIEIQSHLR------
gi|761905244|ref|XP_011402440.1|/1-385  PLGRGKFGNVYLAREKKT-KFVVALKVLFKSQLQKAS-VEHQLRREIEIQAHLR------
gi|759175630|ref|XP_011377486.1|/1-303  PLGKGKFGNVYLARLKES-HFIVALKVLFKSQIEKEG-MEHQLRREIEIQSHLQ------
gi|759149200|ref|XP_011368486.1|/1-311  PLGKGKFGNVYLAREKQS-KFILALKVLFKAQLEKAG-VEHQLRREVEIQSHLR------
gi|759116391|ref|XP_011357226.1|/1-339  PLGKGKFGNVYLAREKKS-HFIVALKVLFKSQIEKEG-VEHQLRREIEIQAHLQ------
gi|758991346|ref|XP_957232.2|/1-420     PLGKGKFGRVYLARERSS-GFICALKVLYKSELQHGTGVEKQVRREIEIQSNLR------
gi|743922878|ref|XP_011005517.1|/1-293  PLGRGKFGHVYLAREKRS-NHIVALKVLFKSQLQQSQ-VEHQLRREVEIQSHLR------
gi|698438703|ref|XP_009757361.1|/1-296  PLGRGKFGHVYLAREKRS-NHVVALKVLFKSQLKQSQ-VEHQLRREVEIQSHLR------
gi|697188794|ref|XP_009603442.1|/1-294  PLGRGKFGHVYLAREKRS-NHIVALKVLFKSQLKQSQ-VEHQLRREVEIQSHLR------
gi|658871623|ref|XP_008418750.1|/1-330  PLGKGKFGNVYLAKEKKH-QFIVALKVLFKSQMEKEG-VEHQLRREIEIQSHLK------
gi|657559162|ref|XP_008283516.1|/1-424  PLGKGKFGNVYLARERQS-KFILALKVLFKKQLEKAG-VEHQLRREVEIQSHLR------
gi|657529148|ref|XP_008293493.1|/1-319  PLGKGKFGNVYLARVKEL-QAIVALKVLFKSQMEKEG-VEHQLRREIEIQAHLK------
gi|655887082|ref|XP_008247187.1|/1-292  PLGKGKFGNVYLARLKDS-HFIVALKVLFKSQIEKEG-MEHQLRREIEIQAHLQ------
gi|655881324|ref|XP_008272369.1|/1-402  PLGKGKFGNVYLAREKQS-KFILALKVLFKAQLEKAG-VEHQLRREVEIQSHLR------
gi|655868725|ref|XP_008268971.1|/1-343  PLGKGKFGNVYLAREKKS-HFIVALKVLFKSQIEKEG-VEHQLRREIEIQAHLQ------
gi|641774253|ref|XP_008170712.1|/1-431  PLGKGKFGNVYLAREKQS-KFILALKVLFKTQLEKAG-VEHQLRREVEIQSHLR------
gi|620969612|ref|XP_007654691.1|/1-303  PLGKGKFGNVYLAREKQS-KFILALKVLFKGQLEKAG-VEHQLRREVEIQSHLR------
gi|617451788|ref|XP_007568855.1|/1-331  PLGKGKFGNVYLAKEKKH-QFIVALKVLFKSQMEKEG-VEHQLRREIEIQSHLK------
gi|594674988|ref|XP_007185333.1|/1-402  PLGKGKFGNVYLAREKQS-KFILALKVLFKAQLEKAG-VEHQLRREVEIQSHLR------
gi|594654199|ref|XP_007175509.1|/1-282  PLGKGKFGNVYLARLKES-HFIVALKVLFKSQIEKEG-LEHQLRREIEIQAHLQ------
gi|594623572|ref|XP_007166415.1|/1-344  PLGKGKFGNVYLAREKKS-RFIVALKVLFKSQIEKEG-VEHQLRREIEIQAHLQ------
gi|584038001|ref|XP_006753367.1|/1-368  PLGKGKFGNVYLARLRDS-HFIVALKVLFKSQLEKEG-MEHQLRREIEIQAHLQ------
gi|573912300|ref|XP_006643864.1|/1-292  PLGRGKFGHVYLAREKRS-NHIVALKVLFKSQLKQSQ-VEHQLRREVEIQSHLR------
gi|554569173|ref|XP_005878848.1|/1-339  PLGKGKFGNVYLAREKKS-RFIVALKVLFKSQIEKEG-VEHQLRREIEIQAHLQ------
gi|545541126|ref|XP_005635254.1|/1-405  PLGKGKFGNVYLAREKQS-KFILAIKVLFKAQLEKAG-VEHQLRREVEIQSHLR------
gi|545487136|ref|XP_005616134.1|/1-307  PLGKGKFGNVYLARLKES-HFIVALKVLFKSQIEKEG-LEHQLRREIEIQAHLQ------
gi|544514002|ref|XP_005590555.1|/1-471  PLGKGKFGNVYLARLKES-HFIVALKVLFKSQIEKEG-LEHQLRREIEIQAHLQ------
gi|530653727|ref|XP_005313062.1|/1-365  PLGKGKFGNVYLARERAS-RFIVALKVLFKSQMEKEG-VEHQLRREIEIQSHLQ------
gi|528993703|ref|XP_005219854.1|/1-303  PLGKGKFGNVYLARLKKN-HFIVALKVLFKSQIEKEG-LEHQLRREVEIQAHLQ------
gi|524890835|ref|XP_005101959.1|/1-332  PLGKGKFGNVYLAREKRS-KYIVALKLMFKSQLVKAG-VEHQLRREIEIQTHLR------
gi|514776534|ref|XP_004968502.1|/1-292  PLGRGKFGHVYLAREKRS-NQIVALKVLFKSQLKQSQ-VEHQLRREVEIQSHLR------
gi|507960369|ref|XP_004686961.1|/1-405  PLGKGKFGNVYLAREKQS-KFILALKVLFKAQLEKAG-VEHQLRREVEIQSHLR------
gi|507953708|ref|XP_004684672.1|/1-344  PLGKGKFGNVYLAREKKS-HFIVALKVLFKSQIEKEG-VEHQLRREIEIQAHLQ------
gi|478517838|ref|XP_004433150.1|/1-344  PLGKGKFGNVYLAREKKS-HFIVALKVLFKSQIEKEG-VEHQLRREIEIQAHLQ------
gi|478512086|ref|XP_004430306.1|/1-405  PLGKGKFGNVYLAREKQS-KFILALKVLFKAQLEKAG-VEHQLRREVEIQSHLR------
gi|470628341|ref|XP_004321082.1|/1-402  PLGKGKFGNVYLAREKQS-KFILALKVLFKAQLEKAG-VEHQLRREVEIQSHLR------
```

```
gi|470261727|ref|XP_004360485.1|/1-457  PLGRGRFGYVYLAREKKS-KFVVALKVLFKSQLQAAN-IEHQLRREIEIQSHLR------
gi|449486478|ref|XP_002195961.2|/1-319  PLGKGKFGNVYLAREKQS-KFILALKVLFKTQLEEAG-VEHQLRREVEIQSHLR------
gi|426243291|ref|XP_004015492.1|/1-303  PLGKGKFGNVYLARLKKN-HFIVALKVLFKSQIEKEG-LEHQLRREVEIQAHLQ------
gi|426237569|ref|XP_004012730.1|/1-344  PLGKGKFGNVYLAREKKS-HFIVALKVLFKSQIEKEG-VEHQLRREIEIQAHLQ------
gi|410979749|ref|XP_003996244.1|/1-344  PLGKGKFGNVYLAREKKS-HFIVALKVLFKSQIEKEG-VEHQLRREIEIQAHLQ------
gi|410953494|ref|XP_003983405.1|/1-405  PLGKGKFGNVYLAREKQS-KFILALKVLFKAQLEKAG-VEHQLRREVEIQSHLR------
gi|410914403|ref|XP_003970677.1|/1-332  PLGKGKFGNVYLARVKKL-EAIVALKVLFKSQMEKEG-VEHQLRREIEIQSHLK------
gi|389641901|ref|XP_003718583.1|/1-397  PLGKGKFGRVYLARERTT-GFICALKVLYKKELESGS-VEKQVRREIEIQTNLC------
gi|389635857|ref|XP_003715581.1|/1-400  PLGRGKFGRVYLAREREH-GFVCALKVLHKRELQDAH-VERQVQREIEIQSNLR------
gi|357126095|ref|XP_003564724.1|/1-291  ALGRGKFGHVYLAREKRS-NQIVALKVLFKSQLKQSQ-VEHQLRREVEIQSHLR------
gi|355703962|gb|EHH30453.1|/1-309       PLGKGKFGNVYLARLKES-HFIVALKVLFKSQIEKEG-LEHQLRREIEIQAHLQ------
gi|350538969|ref|NP_001233545.1|/1-403  PLGKGKFGNVYLAREKQS-KFILALKVLFKAQLEKAG-VEHQLRREVEIQSHLR------
gi|347966592|ref|XP_321274.5|/1-404     PLGRGKFGNVYLAREKET-KFVIALKVLFKKQVHAQG-IEHQVRREIEIQSHLR------
gi|345800233|ref|XP_849906.2|/1-344     PLGKGKFGNVYLAREKKS-HFIVALKVLFKSQIEKEG-VEHQLRREIEIQAHLQ------
gi|344296517|ref|XP_003419953.1|/1-405  PLGKGKFGNVYLAREKQS-KFILALKVLFKAQLEKAG-VEHQVRREVEIQSHLR------
gi|344290468|ref|XP_003416960.1|/1-345  PLGKGKFGNVYLAREKKS-HFIVALKVLFKSQIEKEG-VEHQLRREIEIQAHLQ------
gi|344269722|ref|XP_003406697.1|/1-306  PLGKGKFGNVYLARLQEN-HFLVALKVLFKSQIEKEG-LEHQLRREIEIQAHLQ------
gi|338709975|ref|XP_001502047.3|/1-304  PLGKGKFGNVYLARLKES-HFIVALKVLFKSQIEKEG-LEHQLRREIEIQAHLQ------
gi|334312551|ref|XP_001378079.2|/1-402  PLGKGKFGNVYLAREKQS-KFILALKVLFKAQLEKAG-VEHQLRREVEIQSHLR------
gi|332848444|ref|XP_511856.3|/1-344     PLGKGKFGNVYLAREKKS-HFIVALKVLFKSQIEKEG-VEHQLRREIEIQAHLH------
gi|330793523|ref|XP_003284833.1|/1-359  LLGMGRFGHVYLAREKKS-QFIVALKVLFKYQLQTHN-IEHQLRREIEIQSHLR------
gi|321461440|gb|EFX72472.1|/1-304       PLGKGKFGNVYLAREINT-KYIVALKVLFKSQLQKCQ-MEHQLRREIEIQSHLR------
gi|308497987|ref|XP_003111180.1|/1-306  PLGKGKFGSVYLARTKEG-HFHCAIKVLFKSQLISGG-VEHQLEREIEIQSHLH------
gi|302801442|ref|XP_002982477.1|/1-288  PLGRGKFGNVYLAREKRS-KYVVALKVLFKNQLQQSQ-VEHQLRREIEIQSHLR------
gi|302688103|ref|XP_003033731.1|/1-328  PLGKGKFGRVYMVRTKAEPKYILALKTLYKSEIVQSR-VEKQIRREIEIQQNLR------
gi|297802712|ref|XP_002869240.1|/1-290  PLGRGKFGHVYLAREKRS-NHIVALKVLFKSQLQQSQ-VEHQLRREVEIQSHLR------
gi|281427796|ref|NP_001164000.1|/1-322  PLGRGKFGNVYLAREKQS-KFVVALKVLFKSAIKDFN-NEHQVRREIEIQSHLR------
gi|268565413|ref|XP_002639437.1|/1-302  PLGKGKFGSVYLARTKTG-HFHCAIKVLFKSQLISGG-VEHQLEREIEIQSHLQ------
gi|260784959|ref|XP_002587531.1|/1-282  PLGKGKFGNVYLAREKNS-KFIVALKVLFKSQLMKAG-VEHQLRREIEIQSHLR------
gi|226499040|ref|NP_001148791.1|/1-296  PLGRGKFGHVYLAREKRS-SEIVALKVLFKSQLKQSQ-VEHQLRREVEIQSHLR------
gi|224088986|ref|XP_002308590.1|/1-293  PLGRGKFGHVYLAREKRS-NHIVALKVLFKSQLQQSQ-VEHQLRREVEIQSHLR------
gi|215983064|ref|NP_001135979.1|/1-405  PLGKGKFGNVYLAREKQS-KFILALKVLFKAQLEKAG-VEHQLRREIKIQSHLK------
gi|213404608|ref|XP_002173076.1|/1-346  PLGKGKFGRVYLAKEKKT-GYVVALKTLHKEELVHSK-VERQVRREIEIQSNLR------
gi|198430178|ref|XP_002119314.1|/1-305  PLGRGKFGSVYLAREKKS-KFIVALKVLFKSQLMKSN-VEHQLRREIEIQSHLR------
gi|196012778|ref|XP_002116251.1|/1-270  PLGKGKFGSVYLAREKQS-KYIVALKVLFKSQLQKAN-VEHQLRREIEIQSHLR------
gi|168044897|ref|XP_001774916.1|/1-297  PLGRGKFGNVYLAREKQS-KYVVALKVLFKNQLQQSQ-VEHQLRREIEIQSHLR------
gi|159468203|ref|XP_001692272.1|/1-292  PLGKGKFGNVYLAREKQS-KYIVALKVLFKSQLQQSN-VEHQLRREIEIQAHLR------
gi|158297402|ref|XP_317640.3|/1-307     ALGRGKFGRVYLARERET-GFMVAMKVMFKSQLTKWH-VEKQLLREIEIQSRLK------
gi|156368106|ref|XP_001627537.1|/1-314  PLGKGKFGNVYLAREKKS-KYIVALKVLFKSQLQKSN-VEHQLRREIEIQSHLR------
gi|149734120|ref|XP_001489320.1|/1-404  PLGKGKFGNVYLAREKQS-KFILALKVLFKAQLEKAG-VEHQLRREVEIQSHLR------
gi|149724949|ref|XP_001504864.1|/1-344  PLGKGKFGNVYLAREKKS-HFIVALKVLFKSQIEKEG-VEHQLRREIEIQAHLQ------
gi|148225464|ref|NP_001085657.1|/1-368  PLGKGKFGNVYLAREKQN-KFIMALKVLFKSQLEKEG-VEHQLRREIEIQSHLR------
gi|146172296|ref|XP_001018388.2|/1-360  PLGRGKFGQVHLAREKRS-NFIVALKCISKEQLRRSK-IEHQIRREIEIQSHLN------
gi|144226857|ref|NP_001016859.2|/1-360  PLGKGKFGNVYLARDKQN-KFIMALKVLFKSQLEKEG-VEHQLRREIEIQSHLR------
gi|125524746|gb|EAY72860.1|/1-292       PLGRGKFGHVYLAREKRS-NHIVALKVLFKSQLKQSQ-VEHQLRREVEIQSHLR------
gi|115749607|ref|NP_898907.2|/1-344     PLGKGKFGNVYLAREKKS-HFIVALKVLFKSQIEKEG-VEHQLRREIEIQAHLQ------
gi|115455545|ref|NP_001051373.1|/1-309  YIGEGKFGKVYLAREKQS-GYVVALKVTFKAKLDKYR-FHAHLRREIEIQHGLD------
gi|115435032|ref|NP_001042274.1|/1-292  PLGRGKFGHVYLAREKRS-NHIVALKVLFKSQLKQSQ-VEHQLRREVEIQSHLR------
gi|84000031|ref|NP_001033117.1|/1-402   PLGKGKFGNVYLAREKQS-KFILALKVLFKAQLEKAG-VEHQLRREVEIQSHLR------
gi|83776600|ref|NP_004208.2|/1-344      PLGKGKFGNVYLAREKKS-HFIVALKVLFKSQIEKEG-VEHQLRREIEIQAHLH------
gi|76253851|ref|NP_695208.2|/1-397      PLGKGKFGNVYLAREKQS-KFILALKVLFKVQLEKAG-VEHQLRREVEIQSHLR------
gi|68013217|ref|NP_001018849.1|/1-355   PLGKGKFGRVYLAKEKKT-GFIVALKTLHKSELVQSK-IEKQVRREIEIQSNLR------
gi|66815573|ref|XP_641803.1|/1-384      LLGMGRFGHVYLAREKKS-QFIVALKVLFKNQLQTHN-IEHQLRREIEIQSHLR------
gi|62865639|ref|NP_001015878.1|/1-309   PLGKGKFGNVYLARLKES-HFIVALKVLFKSQIEKEG-LEHQLRREIEIQAHLQ------
gi|62857967|ref|NP_001016570.1|/1-415   PLGKGKFGNVYLARERES-KFILALKVLFKTQLEKAG-VEHQLRREVEIQSHLR------
gi|57525577|ref|NP_001003640.1|/1-405   ALGKGKFGSVYLAREQQT-KFILALKVLFKKQLEKAG-VEHQLRREVEIQSHLR------
gi|50759065|ref|XP_425725.1|/1-409      PLGKGKFGNVYLAREKQS-KFILALKVLFKAQLEDAG-VEHQLRREVEIQSHLR------
gi|50303387|ref|XP_451635.1|/1-361      ILGKGKFGKVYCVKHRET-GLICALKAMEKKEIVQYT-IQKQFRREVEIQGSFK------
gi|46358064|ref|NP_035627.1|/1-417      PLGKGKFGNVYLARERQS-KFILALKVLFKTQLEKAN-VEHQLRREVEIQSHLR------
gi|45198420|ref|NP_985449.1|/1-367      VLGKGKFGRVYCVRHIES-GFVCALKAMEKKDIIQYN-IEKQFRREVEIQSSLR------
gi|38327570|ref|NP_940838.1|/1-403      PLGKGKFGNVYLAREKQS-KFILALKVLFKAQLEKAG-VEHQLRREVEIQSHLR------
gi|17505246|ref|NP_491714.1|/1-305      PLGKGKFGSVYLARTKTG-HFHVAIKVLFKSQLISGG-VEHQLEREIEIQSHLN------
gi|17137514|ref|NP_477336.1|/1-329      HLGRGKFGRVYLARERHS-HYLVAMKVMFKEELRKGC-VQRQVLREIEIQSRLK------
gi|17136516|ref|NP_476749.1|/1-411      LLGRGKFGNVYLAREKES-QFVVALKVLFKRQIGESN-VEHQVRREIEIQSHLR------
gi|16758580|ref|NP_446201.1|/1-343      PLGKGKFGNVYLAREKKS-RFIVALKILFKSQIEKEG-VEHQLRREIEIQAHLK------
gi|15233958|ref|NP_195009.1|/1-294      PLGRGKFGHVYLAREKRS-NHVVALKVLFKSQLQQSQ-VEHQLRREVEIQSHLR------
gi|15225495|ref|NP_182073.1|/1-288      PLGKGKFGRVYLAREAKS-KYIVALKVIFKEQIEKYK-IHHQLRREMEIQTSLR------
gi|6708213|gb|AAF25838.1|/1-282         PLGRGKFGRVYLARLKEN-HFIVALKVLFKSEIEKEG-LEHQLRREVEIQAHLQ------
gi|6325047|ref|NP_015115.1|/1-367       KLGKGKFGKVYCVRHRST-GYICALKVMEKEEIIKYN-LQKQFRREVEIQTSLN------
gi|2979623|gb|AAC12683.1|/1-345         PLGKGKFGNVYLAREKKS-RFIVALKILFKSQIEKEG-VEHQLRREIEIQAHLK------
```

```
                                   :* *:** *: .:         *:*    *  :       . :. **::**  :

XP_020952967.1/1-297                  -----HPNILRLYNYFHDARRVYLILEYAPRGELYKELQKS--HTLDEQRTAT-IMEELA
XP_012380002.1/1-290                  HPSSRHHNILRLYNYFHDTRRVYLILEYAPRGELYKELQKS--CTLDEQRTAT-IMEELA
XP_006102660.1/1-339                  -----HPNILRLYNYFYDRRRIYLILEYAPRGELYKELQKS--RTFDQQRTAT-IMEELA
XP_005673091.1/1-402                  -----HPNILRLYGYFHDATRVYLILEYAPLGAVYRELQKL--SKFDEQRTAT-YITELA
XP_004484680.1/1-405                  -----HPNILRLYGYFHDATRVYLILEYAPLGAVYRELQKL--SKFDEQRTAT-YITELA
XP_004462482.1/1-349                  -----HPNILRLYNYFYDRRRVYLILEYAPRGELYKELQKS--RTFDEQRTAT-IMEELA
Q6NW76.1/1-320                        -----HPNILRFYNYFHDDTRVFLILEYAPRGEMYKELQRY--GRFDDQRTAT-YMEEVS
NP_999084.2/1-344                     -----HPNILRLYNYFYDRRRIYLILEYAPRGELYKELQKC--RTFDEQRTAT-IMEELA
gi|1190438012|ref|XP_020822491.1|/1-365 -----HPNILRLYNYFHDRSRVYLILEYAPRGELYKELQKS--HTFDQQRTAT-IIEELA
gi|1190373986|ref|XP_020837071.1|/1-405 -----HPNILRLYGYFHDATRVYLILEYAPRGEVYRELQKL--SKFDEQRTAT-YITELA
gi|1154067554|ref|NP_001153906.2|/1-332 -----HPNILRFYNYFHDRKRVFLVLEYAPRGEMYKELQKY--QRFDDQRTAT-YMEEIS
gi|1131237831|ref|XP_019799799.1|/1-344 -----HPNILRLYNYFYDRRRIYLILEYAPRGELYKELQKS--HTFDEQRTAT-IMEELA
gi|1131178530|ref|XP_019784431.1|/1-296 -----HPNILRLYNYFHDARRMYLILEYAPRGELYKELQKS--HTLDEQRTAT-VMEELA
gi|1126479796|ref|XP_006940862.2|/1-308 -----HPNILRLYNYFHDARRVYLILEYAPRGELYKELQKS--NTLDEQHTAT-IMEELA
gi|1113784803|ref|XP_019353949.1|/1-376 -----HPNILRLYNYFHDRRRVYLILEYAPRGELYKELQKC--RRFDEQRSAT-IMEELA
gi|1022987698|ref|XP_016286291.1|/1-365 -----HPNILRLYNYFHDRRRVYLILEYAPRGELYKELQKS--HTFDQLRTAT-IMEELA
gi|966994491|ref|XP_014974040.1|/1-344  -----HPNILRLYNYFYDRRRIYLILEYAPRGELYKELQKS--RTFDEQRTAT-IMEELA
gi|966965902|ref|XP_015004225.1|/1-398  -----HPNILRLYGYFHDATRVYLILEYAPLGTVYRELQKL--SKFDEQRTAT-YITELA
gi|955535802|ref|XP_004439167.2|/1-335  -----HPNILRLYNYFHDARRVYLILEYAPRGELYKELQKS--HTLDEQRTAT-IMEELA
gi|951053762|ref|XP_014465099.1|/1-416  -----HPNILRLYGYFHDATRVYLILEYAPRGEVYKELQKL--TKFDEQRTAN-YITELA
gi|946796851|ref|XP_014384184.1|/1-404  -----HPNILRLYGYFHDATRVYLILEYAPLGTVYRELQKL--SRFDEQRTAT-YITELT
gi|946756071|ref|XP_014392719.1|/1-299  -----HPNILRLHNYFHDERXVYLILEYAPRGELYKELQKS--HTIDEQSTAT-IMEELA
gi|922383691|ref|XP_013462066.1|/1-300  -----HPHILRLYGYFYDQKRVYLILEYAPKGELYKELQKC--KYFSERRAAT-YVASLA
gi|909794683|ref|XP_013153467.1|/1-419  -----HPNILRLYGYFHDVTRVYLILEYAPRGEVYKELQKL--TKFDEQRTAT-YITELA
gi|891599507|ref|XP_013025412.1|/1-351  -----HKNILRLFGHFHDEKRIFLILEFAGRGELYQHLRRA--SRFPESTAAK-YIYQMA
gi|891565640|ref|XP_013019059.1|/1-351  -----HKNILRLFGHFHDEKRIFLILEFAGRGELYQHLRKA--SRFSESTAAK-YIYQMA
gi|884853217|ref|XP_013010870.1|/1-315  -----HPNILRLYNYFHDARRXYLIVEYAPRGELYKELLRS--RTLDEQRTAT-IVEELA
gi|830244266|ref|XP_012590158.1|/1-296  -----HPNILRLYNYFHDARRVYLILEYAPRGELYKELQNC--HTLDEQRTAT-IIEELA
gi|828211890|ref|XP_012558808.1|/1-339  -----HPHILRLYGYFYDAKRVFLILEYAPQGELYKHLTKS--ERFNEAKSAT-YISQLA
gi|820033943|ref|NP_001295465.1|/1-282  -----HPNILRLYNYFYDDSRVYLILEYAPRGELYKELQRN--QKLNQQRTAT-IIEELS
gi|795377289|ref|XP_011750175.1|/1-471  -----HPNILRLYNYFHDARRVYLILEFAPRGELYKELQKS--EKLDEQRTAT-IIEELA
gi|779989227|ref|XP_011666707.1|/1-321  -----HPNILRLFGYFYDESRVYLILEYAPRGELYKQLQRA--GRFDEQRTAS-YISQLA
gi|761905244|ref|XP_011402440.1|/1-385  -----HPNILRLYGYFYDDTRVYLILEYAPRGELYKILQKE--GTFSERQSAI-YIKQLA
gi|759175630|ref|XP_011377486.1|/1-303  -----HPNILRLYNYFHDARRVYLILEYAPRGELYKELQKS--QTLDEQRTAT-IMEELA
gi|759149200|ref|XP_011368486.1|/1-311  -----HPNILRLYGYFHDATRVYLILEYAPLGAVYRELQKL--SRFDEQRTAT-YITELA
gi|759116391|ref|XP_011357226.1|/1-339  -----HPNILRLYNYFYDRRRIYLILEYAPRGELYKELQKS--RTFDEQRTAT-IMEELA
gi|758991346|ref|XP_957232.2|/1-420     -----HPNILKLYGHFHDSKRIFLILEYAGKGELYKHLRKE--NRFPEWKAAQ-YVAQMA
gi|743922878|ref|XP_011005517.1|/1-293  -----HPNILRLYGYFYDQKRVYLILEYAAKGELYKELQKC--KYFSERRAAT-YVASLA
gi|698438703|ref|XP_009757361.1|/1-296  -----HPNILRLYGYFYDQKRVYLILEYAAKGELYKELQKC--KYFSERRAAT-YVASLA
gi|697188794|ref|XP_009603442.1|/1-294  -----HPNILRLYGYFYDQKRVYLILEYAAKGELYKELQKC--KYFSERRAAT-YVASLA
gi|658871623|ref|XP_008418750.1|/1-330  -----HPNILRFYNYFHDRKRVFLILEYAPRGEMYKELQKY--GRFDDQRTAT-YMEEIS
gi|657559162|ref|XP_008283516.1|/1-424  -----HPNILRLYGYFHDTSRVYLILEFAPKGELYSELQRS--GNFPESQSAT-YIMELA
gi|657529148|ref|XP_008293493.1|/1-319  -----HPNILRFYNYFHDRKRVFLVLEYAPRGEMYKELQRC--GRFDDRRTAT-YMEEIS
gi|655887082|ref|XP_008247187.1|/1-292  -----HPNILRLYNYFHDARRIYLILEYAPRGELYKELQKC--HTLDEQRTAT-IMEELA
gi|655881324|ref|XP_008272369.1|/1-402  -----HPNILRLYGYFHDATRVYLILEYAPLGTVYRELQKL--SKFDEQRTAT-YITELA
gi|655868725|ref|XP_008268971.1|/1-343  -----HPNILRLYNYFYDRRRIYLILEYAPRGELYKELQKS--RTFDEQRTAT-IMEELA
gi|641774253|ref|XP_008170712.1|/1-431  -----HPNILRLYGYFHDATRVYLILEYAPRGEVYKELQKL--TKFDEQRTAT-YMTELA
gi|620969612|ref|XP_007654691.1|/1-303  -----HPNILRLYGYFHDATRVYLILEYAPRGEVYRELQKF--SKFDEQRTAT-YITELA
gi|617451788|ref|XP_007568855.1|/1-331  -----HPNILRFYNYFHDRKRVFLILEYAPRGEMYKELQKY--GRFDDQRTAT-YMEEIS
gi|594674988|ref|XP_007185333.1|/1-402  -----HPNILRLYGYFHDATRVYLILEYAPLGAVYRELQKL--SKFDEQRTAT-YITELA
gi|594654199|ref|XP_007175509.1|/1-282  -----HPNILRLYNYFHDARRMYLILEYAPRGELYKELQKS--HTLDEQRTAT-VMEELA
gi|594623572|ref|XP_007166415.1|/1-344  -----HPNILRLYNYFYDRRRIYLILEYAPRGELYKELQKS--RTFDEQRTAT-IMEELA
gi|584038001|ref|XP_006753367.1|/1-368  -----HPNILRLYNYFHDERRVYLILEYAPRGELYKELQKS--HTIDEQRAAT-IMEELA
gi|573912300|ref|XP_006643864.1|/1-292  -----HSNILRLYGYFYDQTRVYLILEYAAKGELYKELQRC--KHFSERRSAT-YIASLA
gi|554569173|ref|XP_005878848.1|/1-339  -----HPNILRLYNYFYDRRRIYLILEYAPRGELYKELQKS--RTFDQQRTAT-IMEELA
gi|545541126|ref|XP_005635254.1|/1-405  -----HPNILRLYGYFHDATRVYLILEYAPLGAVYRELQKL--SKFDEQRTAT-YITELA
gi|545487136|ref|XP_005616134.1|/1-307  -----HPNILRLYNYFHDARRVYLILEYAPRGELYKELQKS--HTLDEQHTAT-IMEELA
gi|544514002|ref|XP_005590555.1|/1-471  -----HPNILRLYNYFHDARRVYLILEFAPRGELYKELQKS--EKLDEQRTAT-IIEELA
gi|530653727|ref|XP_005313062.1|/1-365  -----HPNILRLYNYFHDKKRVYLILEYAPRGELYKELQKC--RQFDEQKTAT-FMEELA
gi|528993703|ref|XP_005219854.1|/1-303  -----HPNILRLYNYFHDARRVYLILEYAPKGELYKALQRS--HTFDEQRTAT-IIEELA
gi|524890835|ref|XP_005101959.1|/1-332  -----HPNILKLFGYFHDESRVYLILEYAPKGELYKDLQRQ--KRFDDARSSK-YIAQLA
gi|514776534|ref|XP_004968502.1|/1-292  -----HPNILRLYGYFYDQTRVYLILEYAAKGELYKELTRC--KHFSERRSAT-YIASLA
gi|507960369|ref|XP_004686961.1|/1-405  -----HPNILRLYGYFHDATRVYLILEYAPLGAVYRELQKL--SKFDEQRTAT-YITELA
gi|507953708|ref|XP_004684672.1|/1-344  -----HPNILRLYNYFYDRRRIYLILEYAPRGELYKELQKS--RTFDEQRTAT-IMEELA
gi|478517838|ref|XP_004433150.1|/1-344  -----HPNILRLYNYFYDRRRIYLILEYAPRGELYKELQKS--RTFDEQRTAT-IMEELA
gi|478512086|ref|XP_004430306.1|/1-405  -----HPNILRLYGYFHDATRVYLILEYAPLGAVYRELQKL--SKFDEQRTAT-YITELA
gi|470628341|ref|XP_004321082.1|/1-402  -----HPNILRLYGYFHDATRVYLILEYAPLGAVYRELQKL--SKFDEQRTAT-YITELA
```

```
gi|470261727|ref|XP_004360485.1|/1-457  -----HPNILRLYGYFYDESRVYLIVEFAKGGECFKELQAV--QRFTEKVAAT-YTIQIA
gi|449486478|ref|XP_002195961.2|/1-319  -----HPNILRLYGYFHDVTRVYLILEHAPRGEVYRELQRL--SKFDEQRTAT-YITELA
gi|426243291|ref|XP_004015492.1|/1-303  -----HPNILRLYNYFHDARRVYLILEYAPKGELYKALQRS--HTFDEQRTAT-IIEELA
gi|426237569|ref|XP_004012730.1|/1-344  -----HPNILRLYNYFYDRRRIYLILEYAPRGELYKELQKS--RTFDEQRTAT-IMEELA
gi|410979749|ref|XP_003996244.1|/1-344  -----HPNILRLYNYFYDRRRIYLILEYAPRGELYKELQKS--RTFDEQRTAT-IMEELA
gi|410953494|ref|XP_003983405.1|/1-405  -----HPNILRLYGYFHDATRVYLILEYAPLGAVYRELQKL--SKFDEQRTAT-YITELA
gi|410914403|ref|XP_003970677.1|/1-332  -----HPNILRFYNYFHDRKRVFLVLEYAPRGEMYKELQRC--GRFDDQRTAT-YMEEIS
gi|389641901|ref|XP_003718583.1|/1-397  -----HPNILKMYGHFHDNKRIFLILEFAGKGELYKHLRKE--NRFPEWKAAQ-YVAQMA
gi|389635857|ref|XP_003715581.1|/1-400  -----HPNILKMYGHFHDSKRIFIILEFAGKGELYKHLRKE--TRFPEWRAAQ-CIAQMA
gi|357126095|ref|XP_003564724.1|/1-291  -----HPNILRLYGYFYDQTRVYLILEYAAKGELYKELQRC--KHFSERRSAT-YIASLA
gi|355703962|gb|EHH30453.1|/1-309       -----HPNILRLYNYFHDARRVYLILEFAPRGELYKELQKS--EKLDEQRTAT-IIEELA
gi|350538969|ref|NP_001233545.1|/1-403  -----HPNILRLYGYFHDATRVYLILEYAPLGTVYRELQKL--SKFDEQRTAT-YITELA
gi|347966592|ref|XP_321274.5|/1-404     -----HPNILRMYGYFHDESRIYLILEYAPGGTLFKEQQQQPGKRFPEKRCAI-YVYSLV
gi|345800233|ref|XP_849906.2|/1-344     -----HPNILRLYNYFYDRRRIYLILEYAPRGELYKELQKS--HTFDEQRTAT-IMEELA
gi|344296517|ref|XP_003419953.1|/1-405  -----HPNILRLYGYFHDATRVYLILEYAPLGTVYRELQKX--SKFDEQRTAPXYITELA
gi|344290468|ref|XP_003416960.1|/1-345  -----HPNILRLYNYFYDRRRVYLILEYAPRGELYKELQKS--RTFDEQRTAT-IMEELA
gi|344269722|ref|XP_003406697.1|/1-306  -----HPNILRLYNYFHDSRRVYLILEYAPRGELYKELQKS--HRLDEQRTAT-IMEELA
gi|338709975|ref|XP_001502047.3|/1-304  -----HPNILRLYNYFHDARRVYLILEYAPRGELYKELQKS--HTLDEQRTAT-IMEELA
gi|334312551|ref|XP_001378079.2|/1-402  -----HPNILRLYGYFHDATRVYLILEYAPRGEVYRELQKL--SKFDEQRTAT-YITELA
gi|332848444|ref|XP_511856.3|/1-344     -----HPNILRLYNYFYDRRRIYLILEYAPRGELYKELQKS--RTFDEQRTAT-IMEELA
gi|330793523|ref|XP_003284833.1|/1-359  -----HPNILRLFGYFYDEKRVFLIIEFAKGGECFKELQKV--GSFNEMTAAT-YTLQIA
gi|321461440|gb|EFX72472.1|/1-304       -----HPNVLRMYGYFYDETRIYLILEFAGNGEMYKYLKRQPNGRFSEPQTAN-YMAQLA
gi|308497987|ref|XP_003111180.1|/1-306  -----HPNIVRLYTYFWDAKKIYLVLEYAPGGEMYKSLTNQ--KRFTEVMAGK-YMYEIA
gi|302801442|ref|XP_002982477.1|/1-288  -----HPNILRLYGYFYDQNRVYLILEYAAKGELYKELQRC--KVFSEKRAAT-YIASLA
gi|302688103|ref|XP_003033731.1|/1-328  -----HPNVLRLYGYFHDEKRIFLMLEFAGKGELYKQLSKH--GCFSEKRSAR-YIDQMA
gi|297802712|ref|XP_002869240.1|/1-290  -----HPNILRLYGYFYDQKRVYLILEYAARGELYKELQKC--KYFSERRAAT-YVASLA
gi|281427796|ref|NP_001164000.1|/1-322  -----HNNILRMYGYFHDESRVYIILEYAPNGTCYKALQAAPNRRFPEETCAR-YIRQIA
gi|268565413|ref|XP_002639437.1|/1-302  -----HPNIIRLYNYFWDAKKIYLILEYAPGGEMYKQLTTQ--KRFTEAMAGK-YMYEIA
gi|260784959|ref|XP_002587531.1|/1-282  -----HPHILRLYGYFYDDTRVYLILEYAPRGELYKELQKQ--VRFDERRSAT-YIAQLA
gi|226499040|ref|NP_001148791.1|/1-296  -----HPNILRLYGYFYDQTRVYLILEYAAKGELYKELTRC--KHFSERRTAT-YVASLA
gi|224088986|ref|XP_002308590.1|/1-293  -----HPNILRLYGYFYDQKRVYLILEYAAKGELYKELQKC--KYFSERRAAT-YVASLA
gi|215983064|ref|NP_001135979.1|/1-405  -----HPNILRLYGYFHDATRVYLILEYAPLGAVYRELQKL--SKFDEQRTAT-YITELA
gi|213404608|ref|XP_002173076.1|/1-346  -----HKNILRLFGHFHDEKRIFLILEFAGRGELYKHLRRQ--QRFSEELSAK-YIFQMA
gi|198430178|ref|XP_002119314.1|/1-305  -----HPHILRLYGYFHDETRVYLILEYASRGEMYKELQKQ--GKFTEEMSAT-YIAELA
gi|196012778|ref|XP_002116251.1|/1-270  -----HPNILRLYGYFYDTSRVYLILEFAPGGELYKELQRQ--KVFDEPRAAK-FIAQLS
gi|168044897|ref|XP_001774916.1|/1-297  -----HPNILRLYGYFYDQARVYLILEYAAKGELYKELQRC--KVFSEKRAAT-YIASLA
gi|159468203|ref|XP_001692272.1|/1-292  -----HPNILRLYGYFYDKEKVYLILEYAARGELYKELVRC--NHFDERTSAT-YILSLA
gi|158297402|ref|XP_317640.3|/1-307     -----HPHILRLYTWFHDDRRIYLALELAAQGELYKHLKAAPKGRFDERRSAR-YISQVA
gi|156368106|ref|XP_001627537.1|/1-314  -----HNNILRLFGYFYDDTRVYLILEYAPRGELYKELTAC--EKFDEKRAAK-YIRQLA
gi|149734120|ref|XP_001489320.1|/1-404  -----HPNILRLYGYFHDATRVYLILEYAPLGAVYRELQKL--SKFDEQRTAT-YITELA
gi|149724949|ref|XP_001504864.1|/1-344  -----HPNILRLYNYFYDRRRIYLILEYAPRGELYKELQKS--RTFDEQRTAT-IMEELA
gi|148225464|ref|NP_001085657.1|/1-368  -----HPNILRMYNYFHDRKRIYLMLEFAPRGELYKELQKH--GRFDEQRSAT-FMEELA
gi|146172296|ref|XP_001018388.2|/1-360  -----HKNVLRMFGFFWDEQKIYLILEYAPQGELYQDLQKQINKRYPEQKAAN-YIKQMA
gi|144226857|ref|NP_001016859.2|/1-360  -----HPNILRMYNYFHDRKRIYLMLEFAPRGELYKELQKH--GRFDEQRSAT-FMEELA
gi|125524746|gb|EAY72860.1|/1-292       -----HPNILRLYGYFYDQTRVYLILEYALKGELYKELQRC--KHFSERRSAT-YIASLA
gi|115749607|ref|NP_898907.2|/1-344     -----HPNILRLYNYFYDRRRIYLILEYAPRGELYKELQKS--RTFDEQRTAT-IMEELA
gi|115455545|ref|NP_001051373.1|/1-309  -----HPNVLRLFAWFHDAERVVLVLEYAARGELYKLLRT--VRRFSERTAAT-YVASLA
gi|115435032|ref|NP_001042274.1|/1-292  -----HPNILRLYGYFYDQTRVYLILEYALKGELYKELQRC--KHFSERRSAT-YIASLA
gi|84000031|ref|NP_001033117.1|/1-402   -----HPNILRLYGYFHDATRVYLILEYAPLGAVYRELQKL--SKFDEQRTAT-YITELA
gi|83776600|ref|NP_004208.2|/1-344      -----HPNILRLYNYFYDRRRIYLILEYAPRGELYKELQKS--CTFDEQRTAT-IMEELA
gi|76253851|ref|NP_695208.2|/1-397      -----HPNILRLYGYFHDATRVYLILEYAPLGTVYRELQKL--SKFDEQRTAT-YITELA
gi|68013217|ref|NP_001018849.1|/1-355   -----HKNILRLYGHFHDEKRIYLILEFAGRGELYQHLRRA--KRFSEEVASK-YIFQMA
gi|66815573|ref|XP_641803.1|/1-384      -----HPNILRLFGYFYDDKRVFLIIEFAKGGECFKELQKV--GSFNEQTAAT-YTLQIA
gi|62865639|ref|NP_001015878.1|/1-309   -----HPNILRLYNYFHDARRVYLILEYAPRGELYKELQKS--EKLDEQRTAT-IIEELA
gi|62857967|ref|NP_001016570.1|/1-415   -----HPNILRLYGYFHDAARVYLILDYAPGGELFRELQKV--TRFDEQKSAL-YIKQLA
gi|57525577|ref|NP_001003640.1|/1-405   -----HPNILRLYGYFHDAARVYLILEFAPKGELYGELQRC--GTFDDQRSAT-YIMELA
gi|50759065|ref|XP_425725.1|/1-409      -----HPNILRLYGYFHDVTRVYLILEYAPRGEVFKELQKL--TKFDEQRTAT-YITELA
gi|50303387|ref|XP_451635.1|/1-361      -----HKNLTQLYGFFYDEKRVYLLMEYVYYGELYKFLKNN--GPLNETLASY-FVYQMA
gi|46358064|ref|NP_035627.1|/1-417      -----HPNILRLYGYFHDATRVYLILEYAPLGTVYRELQKL--SKFDEQRTAT-YITELA
gi|45198420|ref|NP_985449.1|/1-367      -----HPNLTQLYGYFHDEKRVYLLMEYLVNGELYKHLKGR--SHFNDVVASY-YVYQMA
gi|38327570|ref|NP_940838.1|/1-403      -----HPNILRLYGYFHDATRVYLILEYAPLGTVYRELQKL--SKFDEQRTAT-YITELA
gi|17505246|ref|NP_491714.1|/1-305      -----HPNIIKLYTYFWDAKKIYLVLEYAPGGEMYKQLTVS--KRFSEPTAAK-YMYEIA
gi|17137514|ref|NP_477336.1|/1-329      -----HPHILRLLTWFHDESRIYLALEIASEGELFKHLRGAPNHRFDEPRSAK-YTYQVA
gi|17136516|ref|NP_476749.1|/1-411      -----HPHILRLYAYFHDDVRIYLILEYAPQGTLFNALQAQPMKRFDERQSAT-YIQALC
gi|16758580|ref|NP_446201.1|/1-343      -----HPNILQLYNYFYDQQRIYLILEYAPRGELYKELQKS--GTFDEQRTAT-IMEELS
gi|15233958|ref|NP_195009.1|/1-294      -----HPNILRLYGYFYDQKRVYLILEYAARGELYKDLQKC--KYFSERRAAT-YVASLA
gi|15225495|ref|NP_182073.1|/1-288      -----HPNILRLFGWFHDNERIFLILEYAHGGELYGVLKQ--NGHLTEQQAAT-YIASLS
gi|6708213|gb|AAF25838.1|/1-282         -----HPNILRLYNYFYDDTRIYLILEYAPGGELYKELQRH--QKLDQQRTAT-IIQELS
gi|6325047|ref|NP_015115.1|/1-367       -----HPNLTKSYGYFHDEKRVYLLMEYLVNGEMYKLLRLH--GPFNDILASD-YIYQIA
gi|2979623|gb|AAC12683.1|/1-345         -----HPNILQLYNYFYDQQRIYLILEYAPRGELYKELQKS--RTFDEQRTAT-IMEELS
```

```
                                             * :: :    * *     : ::    *  :            :   .      :

XP_020952967.1/1-297                    DALTYCHEKKVIHRDIKPENLLLGFRGEVKIADFGWSVHTP-SLRRKTMCGTLDYLPPEM
XP_012380002.1/1-290                    DALTYCHQQKVIHRDIKPENLLLGLRGEVKIADFGWSVHTP-SLRRRTMCGTLDYLPPEM
XP_006102660.1/1-339                    DALMYCHKKKVIHRDIKPENLLLGLQGELKIADFGWSVHAP-SLRRKTMCGTLDYLPPEM
XP_005673091.1/1-402                    NALSYCHSKRVIHRDIKPENLLLGSAGELKIADFGWSVHAP-SSRRTTLCGTLDYLPPEM
XP_004484680.1/1-405                    DALSYCHSKKVIHRDIKPENLLLGSAGELKIADFGWSVHAP-SSRRTTLCGTLDYLPPEM
XP_004462482.1/1-349                    DALMYCHGRKVIHRDIKPENLLLGLRGELKIADFGWSVHAP-SLRRKTMCGTLDYLPPEM
Q6NW76.1/1-320                          DALQYCHEKKVIHRDIKPENLLLGYRGELKIADFGWSVHAP-SLRRRTMCGTLDYLPPEM
NP_999084.2/1-344                       DALIYCHGKKVIHRDIKPENLLLGLQGELKIADFGWSVHAP-SLRRKTMCGTLDYLPPEM
gi|1190438012|ref|XP_020822491.1|/1-365 DALIYCHGKKVIHRDIKPENLMGLRGELKIADFGWSVHAP-SLRRKTMCGTLDYLPPEM
gi|1190373986|ref|XP_020837071.1|/1-405 DALSYCHSKKVIHRDIKPENLLLGSDGELKIADFGWSVHAP-SSRRTTLCGTLDYLPPEM
gi|1154067554|ref|NP_001153906.2|/1-332 DALLYCHEKKVIHRDIKPENLLLGYRGELKIADFGWSVHAP-SLRRRTMCGTLDYLPPEM
gi|1131237831|ref|XP_019799799.1|/1-344 DALIYCHGKKVIHRDIKPENLLLGLQGELKIADFGWSVHAP-SLRRKTMCGTLDYLPPEM
gi|1131178530|ref|XP_019784431.1|/1-296 DALTYCHEKKVIHRDIKPENLLLGFRGEVKIADFGWSVHTP-SLRRRTMCGTLDYLPPEM
gi|1126479796|ref|XP_006940862.2|/1-308 DALTYCHEKKVIHRDIKPENLLLGFRGEVKIADFGWSVHTP-SLRRKTMCGTLDYLPPEM
gi|1113784803|ref|XP_019353949.1|/1-376 DALIYCHAKKVIHRDIKPENLLLGLKGELKIADFGWSVHAP-SLRRKTMCGTLDYLPPEM
gi|1022987698|ref|XP_016286291.1|/1-365 DALIYCHKKKVIHRDIKPENLMGLRGELKIADFGWSVHAP-SLRRKTMCGTLDYLPPEM
gi|966994491|ref|XP_014974040.1|/1-344  DALMYCHGKKVIHRDIKPENLLLGLKGELKIADFGWSVHAP-SLRRKTMCGTLDYLPPEM
gi|966965902|ref|XP_015004225.1|/1-398  NALSYCHSKRVIHRDIKPENLLLGSAGELKIADFGWSVHAP-SSRRTTLCGTLDYLPPEM
gi|955535802|ref|XP_004439167.2|/1-335  DALTYCHEKKVIHRDIKPENLLLGFRGEVKIADFGWSVHTP-SLRRKTMCGTLDYLPPEM
gi|951053762|ref|XP_014465099.1|/1-416  DALSYCHSKRVIHRDIKPENLLLGSNGELKMADFGWSVHAP-SSRRTTLCGTLDYLPPEM
gi|946796851|ref|XP_014384184.1|/1-404  NALSYCHSKRVIHRDIKPENLLLGSTGELKIADFGWSVHAP-SSRRTTLCGTLDYLPPEM
gi|946756071|ref|XP_014392719.1|/1-299  HALAYCHENKVIHRDIKPENLLLGLRGEVKIADFGWSVHTP-SLRRKTMCGTLDYLPPEM
gi|922383691|ref|XP_013462066.1|/1-300  RALIYCHGKHVIHRDIKPENLLIGAQGELKIADFGWSVHT--FNRRRTMCGTLDYLPPEM
gi|909794683|ref|XP_013153467.1|/1-419  DALLYCHSKSVIHRDIKPENLLLGSNGELKIADFGWSVHAP-SSRRTTLCGTLDYLPPEM
gi|891599507|ref|XP_013025412.1|/1-351  NALAYLHKKHVIHRDIKPENILLGIDGEIKLSDFGWSVHAP-SNRRTTLCGTLDYLPPEM
gi|891565640|ref|XP_013019059.1|/1-351  NALAYLHKKHVIHRDIKPENILLGIDGEIKLSDFGWSVHAP-SNRRTTLCGTLDYLPPEM
gi|884853217|ref|XP_013010870.1|/1-315  DALTYCHAKKVIHRDIKPENLLLGFQGEVKIADFGWSVHTL-SLRRKTMCGTMDYLPPEI
gi|830244266|ref|XP_012590158.1|/1-296  DALTYCHEKKVIHRDIKPENLLLGYRGEVKIADFGWSVHTP-SLRRKTMCGTLDYLPPEM
gi|828211890|ref|XP_012558808.1|/1-339  SALEYCHSKKVIHRDIKPENLLLGMKGELKISDFGWSVHAP-SSRRTTLCGTLDYLPPEM
gi|820033943|ref|NP_001295465.1|/1-282  DALIYCHRNKVIHRDLKPENLLLGLKGEVKIADFGWSVHTP-SLRRKTMCGTLDYLPPEM
gi|795377289|ref|XP_011750175.1|/1-471  DALTYCHEKKVIHRDIKPENLLLGFRGEVKIADFGWSVHTP-SLRRKTMCGTLDYLPPEM
gi|779989227|ref|XP_011666707.1|/1-321  DALKYCHSKKVIHRDIKPENLLLGLLGDLKIADFGWSVHAP-SSRRNTLCGTMDYLPPEM
gi|761905244|ref|XP_011402440.1|/1-385  DALRYCHSKKVIHRDIKPENLLVNFKGDLKIADFGWSVHAP-SSRRTTLCGTLDYLPPEM
gi|759175630|ref|XP_011377486.1|/1-303  DALTYCHENKVIHRDIKPENLLLGFRGEVKIADFGWSVHTP-SLRRNTMCGTLDYLPPEM
gi|759149200|ref|XP_011368486.1|/1-311  NALSYCHSKRVIHRDIKPENLLLGSAGELKIADFGWSVHAP-SSRRTTLCGTLDYLPPEM
gi|759116391|ref|XP_011357226.1|/1-339  DALIYCHGKKVIHRDIKPENLLLGLQGELKIADFGWSVHAP-SLRRKTMCGTLDYLPPEM
gi|758991346|ref|XP_957232.2|/1-420     SALKYLHRKHVIHRDIKPENILVGIHGEIKISDFGWSVHAP-NNRRQTLCGTLDYLPPEM
gi|743922878|ref|XP_011005517.1|/1-293  RALIYCHSKHVIHRDIKPENLLIGAQGELKIADFGWSVHT--FNRRRTMCGTLDYLPPEM
gi|698438703|ref|XP_009757361.1|/1-296  RALIYCHGKHVIHRDIKPENLLVGAQGELKIADFGWSVHT--FNRRRTMCGTLDYLPPEM
gi|697188794|ref|XP_009603442.1|/1-294  RALIYCHGKHVIHRDIKPENLLVGAQGELKIADFGWSVHT--FDRRRTMCGTLDYLPPEM
gi|658871623|ref|XP_008418750.1|/1-330  DALMYCHEKKVIHRDIKPENLLLGFRGELKIADFGWSVHAP-SLRRRTMCGTLDYLPPEM
gi|657559162|ref|XP_008283516.1|/1-424  DALNYCHSKKVIHRDIKPENLLLGANGELKIADFGWSVHTP-SSRRSTLCGTLDYLPPEM
gi|657529148|ref|XP_008293493.1|/1-319  DALMYCHEKKVIHRDIKPENLLLGYRGELKIADFGWSVHAP-SLRRRTMCGTLDYLPPEM
gi|655887082|ref|XP_008247187.1|/1-292  DALTYCHGKKVIHRDIKPENLLLGFRGEVKIADFGWSVHTL-SLRRKTMCGTLDYLPPEM
gi|655881324|ref|XP_008272369.1|/1-402  NALLYCHSKRVIHRDIKPENLLLGSAGELKIADFGWSVHAP-SSRRTTLCGTLDYLPPEM
gi|655868725|ref|XP_008268971.1|/1-343  DALRYCHEKKVIHRDIKPENLLLGLQGELKIADFGWSVHAP-SLRRKTMCGTLDYLPPEM
gi|641774253|ref|XP_008170712.1|/1-431  DALSYCHSKRVIHRDIKPENLLLGSNGELKIADFGWSVHAP-SSRRTTLCGTLDYLPPEM
gi|620969612|ref|XP_007654691.1|/1-303  DALSYCHSKKVIHRDIKPENLLLGSDGELKIADFGWSVHAP-SSRRTTLCGTLDYLPPEM
gi|617451788|ref|XP_007568855.1|/1-331  DALMYCHEKKVIHRDIKPENLLLGFRGELKIADFGWSVHAP-SLRRRTMCGTLDYLPPEM
gi|594674988|ref|XP_007185333.1|/1-402  NALSYCHSKRVIHRDIKPENLLLGSSGELKIADFGWSVHAP-SSRRTTLCGTLDYLPPEM
gi|594654199|ref|XP_007175509.1|/1-282  DALTYCHEKKVIHRDIKPENLLLGFRGEVKIADFGWSVHTP-SLRRRTMCGTLDYLPPEM
gi|594623572|ref|XP_007166415.1|/1-344  DALIYCHGKKVIHRDIKPENLLLGLQGELKIADFGWSVHAP-SLRRKTMCGTLDYLPPEM
gi|584038001|ref|XP_006753367.1|/1-368  RALAYCHENKVIHRDIKPENLLLGLRGEVKIADFGWSVHTP-SLRRKTMCGTLDYLPPEM
gi|573912300|ref|XP_006643864.1|/1-292  RALIYLHGKHVIHRDIKPENLLIGSQGELKIADFGWSVHT--FNRRRTMCGTLDYLPPEM
gi|554569173|ref|XP_005878848.1|/1-339  DALMYCHKKKVIHRDIKPENLLLGLQGELKIADFGWSVHAP-SLRRKTMCGTLDYLPPEM
gi|545541126|ref|XP_005635254.1|/1-405  DALSYCHSKRVIHRDIKPENLLLGSAGELKIADFGWSVHAP-SSRRTTLCGTLDYLPPEM
gi|545487136|ref|XP_005616134.1|/1-307  DALTYCHEKKVIHRDIKPENLLLGFKGEVKIADFGWSVHTP-SLRRKTMCGTLDYLPPEM
gi|544514002|ref|XP_005590555.1|/1-471  DALTYCHEKKVIHRDIKPENLLLGFRGEVKIADFGWSVHTP-SLRRKTMCGTLDYLPPEM
gi|530653727|ref|XP_005313062.1|/1-365  DALIYCHAKKVIHRDIKPENLMGLKGELKIADFGWSVHAP-SLRRRTMCGTLDYLPPEM
gi|528993703|ref|XP_005219854.1|/1-303  DALIYCHERKVIHRDIKPENLLLGLMGEVKIADFGWSVHTP-SLRRRTTCGTLDYLPPEM
gi|524890835|ref|XP_005101959.1|/1-332  DALIYCHAKKVIHRDIKPENILLGLKGDVKIADFGWSVHAP-SSRRQTMCGTLDYLPPEM
gi|514776534|ref|XP_004968502.1|/1-292  RALIYLHGKHVIHRDIKPENLLIGVQGELKIADFGWSVHT--FNRRRTMCGTLDYLPPEM
gi|507960369|ref|XP_004686961.1|/1-405  DALSYCHSKRVIHRDIKPENLLLGSAGELKIADFGWSVHAP-SSRRTTLCGTLDYLPPEM
gi|507953708|ref|XP_004684672.1|/1-344  DALIYCHGKKVIHRDIKPENLLLGLQGELKIADFGWSVHAP-SLRRKTMCGTLDYLPPEM
gi|478517838|ref|XP_004433150.1|/1-344  DALIYCHGKKVIHRDIKPENLLLGLQGELKIADFGWSVHAP-SLRRKTMCGTLDYLPPEM
gi|478512086|ref|XP_004430306.1|/1-405  NALAYCHSKRVIHRDIKPENLLLGSAGELKIADFGWSVHAP-SSRRTTLCGTLDYLPPEM
gi|470628341|ref|XP_004321082.1|/1-402  NALSYCHSKRVIHRDIKPENLLLGSSGELKIADFGWSVHAP-SSRRTTLCGTLDYLPPEM
```

gi|470261727|ref|XP_004360485.1|/1-457	DALRYCHSKHVIHRDIKPENLLIGINGEIKIADFGWSVHAP-NSKRQTYCGTVEYLPPEI
gi|449486478|ref|XP_002195961.2|/1-319	DALSYCHSKRVIHRDIKPENLLLGSNGELKIADFGWSVHAP-SSRRTTLCGTLDYLPPEM
gi|426243291|ref|XP_004015492.1|/1-303	DALIYCHEKKVIHRDIKPENLLLGLMGEVKIADFGWSVHTP-SLRRRTTCGTLDYLPPEM
gi|426237569|ref|XP_004012730.1|/1-344	DALTYCHAKKVIHRDIKPENLLLGLRGELKIADFGWSVHAP-SLRRKTMCGTLDYLPPEM
gi|410979749|ref|XP_003996244.1|/1-344	DALLYCHGKKVIHRDIKPENLLLGLQGELKIADFGWSVHAP-SLRRKTMCGTLDYLPPEM
gi|410953494|ref|XP_003983405.1|/1-405	DALSYCHSKRVIHRDIKPENLLLGSAGELKIADFGWSVHAP-SSRRTTLCGTLDYLPPEM
gi|410914403|ref|XP_003970677.1|/1-332	DALLYCHERKVIHRDIKPENLLLGYRGELKIADFGWSVHAP-SLRRRTMCGTLDYLPPEM
gi|389641901|ref|XP_003718583.1|/1-397	SALKYLHRKHVIHRDIKPENILVGLHGELKISDFGWSVHAP-NNRRKTLCGTLDYLPPEM
gi|389635857|ref|XP_003715581.1|/1-400	SALRYLHRKHVMHRDIKPENILVGFYGEIKISDFGWSVHAP-SDRRTTFCGTLDYLPPEM
gi|357126095|ref|XP_003564724.1|/1-291	RALIYLHGKHVIHRDIKPENLLIGVQGELKIADFGWSVHT--FNRRRTMCGTLDYLPPEM
gi|355703962|gb|EHH30453.1|/1-309	DALTYCHEKKVIHRDIKPENLLLGFRGEVKIADFGWSVHTP-SLRRKTMCGTLDYLPPEM
gi|350538969|ref|NP_001233545.1|/1-403	NALSYCHSKRVIHRDIKPENLLLGSAGELKIADFGWSVHAP-SSRRTTLCGTLDYLPPEM
gi|347966592|ref|XP_321274.5|/1-404	SALIYLHERNVIHRDIKPENLLLGHGGELKIADFGWSVHEP-TSSRTTLCGTLDYLSPEM
gi|345800233|ref|XP_849906.2|/1-344	DALMYCHGKKVIHRDIKPENLLLGLQGELKIADFGWSVHAP-SLRRKTMCGTLDYLPPEM
gi|344296517|ref|XP_003419953.1|/1-405	DALSYCHSKKVIHRDIKPENLLLGSTGELKIADFGWSVHAP-SSRRTTLCGTLDYLPPEM
gi|344290468|ref|XP_003416960.1|/1-345	DALMYCHGKKVIHRDIKPENLLLGLRGELKIADFGWSVHAP-SLRRKTMCGTLDYLPPEM
gi|344269722|ref|XP_003406697.1|/1-306	DALIYCHEKKVIHRDIKPENLLLGLRGEVKIADFGWSVHTL-SLRRKTMCGTLDYLPPEM
gi|338709975|ref|XP_001502047.3|/1-304	DALTYCHEKKVIHRDIKPENLLLGFRGEVKIADFGWSVHTP-SLRRKTMCGTLDYLPPEM
gi|334312551|ref|XP_001378079.2|/1-402	DALSYCHSKKVIHRDIKPENLLLGSDGELKIADFGWSVHAP-SSRRTTLCGTLDYLPPEM
gi|332848444|ref|XP_511856.3|/1-344	DALMYCHGKKVIHRDIKPENLLLGLKGELKIADFGWSVHAP-SLRRKTMCGTLDYLPPEM
gi|330793523|ref|XP_003284833.1|/1-359	DALRYCHSKHVIHRDIKPENLLIGINGEIKIADFGWSVHAP-NTKRNTFCGTLEYLPPEV
gi|321461440|gb|EFX72472.1|/1-304	DALMYCHARKVIHRDIKPENLLLGIRGEIKIADFGWSVHAP-SSRRTTMCGTLDYLAPEM
gi|308497987|ref|XP_003111180.1|/1-306	DALQYCHHKKVIHRDIKPENLLIGPQGELKIGDFGWSVHAP-SNKRQTMCGTMDYLPPEM
gi|302801442|ref|XP_002982477.1|/1-288	RALMYCHEKHVIHRDIKPENLLIGMKGELKIADFGWSVHT--FNRRRTMCGTLDYLPPEM
gi|302688103|ref|XP_003033731.1|/1-328	DALTYLHGKHVIHRDIKPENLLIGINGELKIGDFGWSVHAP-GNRRKTMCGTLDYLPPEM
gi|297802712|ref|XP_002869240.1|/1-290	RALIYCHGKHVIHRDIKPENLLIGAQGELKIADFGWSVHT--FNRRRTMCGTLDYLPPEM
gi|281427796|ref|NP_001164000.1|/1-322	DALSYCHTKKVIHRDIKPENLLLCAQGQIKIADFGWSVHAP-SSRRTTLCGTLDYLPPEM
gi|268565413|ref|XP_002639437.1|/1-302	DALSYCHRKNVIHRDIKPENLLIGAQGELKIGDFGWSVHAP-SNKRQTMCGTMDYLPPEM
gi|260784959|ref|XP_002587531.1|/1-282	DALKYCHSKKVIHRDIKPENLLLGLKGDLKIADFGWSVHAP-SSRRATLCGTLDYLPPEM
gi|226499040|ref|NP_001148791.1|/1-296	RALIYLHGKHVIHRDIKPENLLVGVQGEIKIADFGWSVHT--FNRRRTMCGTLDYLPPEM
gi|224088986|ref|XP_002308590.1|/1-293	RALIYCHGKHVIHRDIKPENLLIGAQGELKIADFGWSVHT--FNRRQTMCGTLDYLPPEM
gi|215983064|ref|NP_001135979.1|/1-405	NALSYCHSKRVIHRDIKPENLLLGSAGELKIADFGWSVHAP-SSRRTTLCGTLDYLPPEM
gi|213404608|ref|XP_002173076.1|/1-346	QALAYLHKKHVIHRDIKPENILLGIDGEIKLSDFGWSVHAP-SNRRTTLCGTLDYLPPEM
gi|198430178|ref|XP_002119314.1|/1-305	DALNYCHSKQVIHRDIKPENLLMGLRGELKIADFGWSVHAP-SSKRQTLCGTLDYLPPEM
gi|196012778|ref|XP_002116251.1|/1-270	DALRYCHTKKVIHRDIKPENLLLGIKGDLKIADFGWSVHAP-SSRRTTLCGTLDYLPPEM
gi|168044897|ref|XP_001774916.1|/1-297	RALMYCHEKHVIHRDIKPENLLIGLKGELKIADFGWSVHT--CNRRRTLCGTLDYLPPEM
gi|159468203|ref|XP_001692272.1|/1-292	RALHYCHTKHVIHRDVKPENLLVGLNGELKISDFGWSVHAP-SNRRKTLCGTLDYLPPEM
gi|158297402|ref|XP_317640.3|/1-307	DALNYCHANNVIHRDLKPENILLTDEDNIKLADFGWSAHTN-SNKRKTMCGTLDYLPPEM
gi|156368106|ref|XP_001627537.1|/1-314	DALAYCHSKKVIHRDIKPENLLLNYKGDIKIADFGWSVHAP-SSRRTTLCGTLDYLPPEM
gi|149734120|ref|XP_001489320.1|/1-404	NALAYCHSKRVIHRDIKPENLLLGSAGELKIADFGWSVHAP-SSRRTTLCGTLDYLPPEM
gi|149724949|ref|XP_001504864.1|/1-344	DALMYCHGKKVIHRDIKPENLLLGLQGELKIADFGWSVHAP-SLRRKTMCGTLDYLPPEM
gi|148225464|ref|NP_001085657.1|/1-368	DALQYCHERKVIHRDIKPENLLMGYKGELKIADFGWSVHAP-SLRRRTMCGTLDYLPPEM
gi|146172296|ref|XP_001018388.2|/1-360	EALIYLHSKDIIHRDIKPEN-LLNSFGTIKIADFGWSIHSP-SNKRQTICGTLDYLSPEM
gi|144226857|ref|NP_001016859.2|/1-360	DALQYCHERKVIHRDIKPENLLMGYKGELKIADFGWSVHAP-SLRRRTMCGTLDYLPPEM
gi|125524746|gb|EAY72860.1|/1-292	RALIYLHGKHVIHRDIKPENLLIGSQGELKIADFGWSVHT--FNRRRTMCGTLDYLPPEM
gi|115749607|ref|NP_898907.2|/1-344	DALTYCHAKKVIHRDIKPENLLLGLRGELKIADFGWSVHAP-SLRRKTMCGTLDYLPPEM
gi|115455545|ref|NP_001051373.1|/1-309	GALAYCHKKQVIHRDIKPENLLLDIEGRLKIADFGWAVRS--NAKRHTLCGTIDYLAPEM
gi|115435032|ref|NP_001042274.1|/1-292	HALIYLHGKHVIHRDIKPENLLIGSQGELKIADFGWSVHT--FNRRRTMCGTLDYLPPEM
gi|84000031|ref|NP_001033117.1|/1-402	NALSYCHSKRVIHRDIKPENLLLGSAGELKIADFGWSVHAP-SSRRTTLCGTLDYLPPEM
gi|83776600|ref|NP_004208.2|/1-344	DALMYCHGKKVIHRDIKPENLLLGLKGELKIADFGWSVHAP-SLRRKTMCGTLDYLPPEM
gi|76253851|ref|NP_695208.2|/1-397	NALSYCHSKRVIHRDIKPENLLLGSNGELKIADFGWSVHAP-SSRRTTLCGTLDYLPPEM
gi|68013217|ref|NP_001018849.1|/1-355	NALSYLHKKHVIHRDIKPENILLGIDGEIKLSDFGWSVHAP-SNRRTTLCGTLDYLPPEM
gi|66815573|ref|XP_641803.1|/1-384	DALRYCHSKHVIHRDIKPENLLIGVGGEIKIADFGWSVHAP-NTKRSTFCGTLEYLPPEV
gi|62865639|ref|NP_001015878.1|/1-309	DALTYCHDKKVIHRDIKPENLLLGFRGEVKIADFGWSVHTP-SLRRKTMCGTLDYLPPEM
gi|62857967|ref|NP_001016570.1|/1-415	EALLYCHSKNVIHRDIKPENLLLGANGELKIADFGWSVHAP-SSRRTTLCGTLDYLPPEM
gi|57525577|ref|NP_001003640.1|/1-405	DALRYCHSKAVIHRDIKPENLLLGANGELKIADFGWSVHTP-SSRRSTLCGTLDYLPPEM
gi|50759065|ref|XP_425725.1|/1-409	DALSYCHSKSVIHRDIKPENLLLGSNGELKIADFGWSVHAP-SSRRSTLCGTLDYLPPEM
gi|50303387|ref|XP_451635.1|/1-361	NALDYMHSKNILHRDIKPENILIGFNNTIKLTDFGWSVYNEDGQKRKTLCGTIDYLSPEL
gi|46358064|ref|NP_035627.1|/1-417	NALSYCHSKRVIHRDIKPENLLLGSNGELKIADFGWSVHAP-SSRRTTMCGTLDYLPPEM
gi|45198420|ref|NP_985449.1|/1-367	DALDYMHERNILHRDIKPENIIIGFNNTIKLTDFGWSVITPKGSKRKTLCGTVDYLSPEL
gi|38327570|ref|NP_940838.1|/1-403	NALSYCHSKRVIHRDIKPENLLLGSAGELKIADFGWSVHAP-SSRRTTLCGTLDYLPPEM
gi|17505246|ref|NP_491714.1|/1-305	DALSYCHRKNVIHRDIKPENLLIGSQGELKIGDFGWSVHAP-SNKRQTMCGTMDYLPPEM
gi|17137514|ref|NP_477336.1|/1-329	NALNYCHLNNVIHRDLKPENILLTSTDDLKLADFGWSAHTP-NNKRRTLCGTLDYLPPEM
gi|17136516|ref|NP_476749.1|/1-411	SALLYLHERDIIHRDIKPENLLLGHKGVLKIADFGWSVHEP-NSMRMTLCGTVDYLPPEM
gi|16758580|ref|NP_446201.1|/1-343	DALMYCHKKKVIHRDIKPENLLLGLQGELKIADFGWSVHAP-SLRRKTMCGTLDYLPPEM
gi|15233958|ref|NP_195009.1|/1-294	RALIYCHGKHVIHRDIKPENLLIGAQGELKIADFGWSVHT--FNRRRTMCGTLDYLPPEM
gi|15225495|ref|NP_182073.1|/1-288	QALAYCHGKCVIHRDIKPENLLLDHEGRLKIADFGWSVQS--SNKRKTMCGTLDYLAPEM
gi|6708213|gb|AAF25838.1|/1-282	DALTYCHEKKVIHRDIKPENLLLGLNGEVKISDFGWSVHTP-SLRRKTMCGTLDYLPPEM
gi|6325047|ref|NP_015115.1|/1-367	NALDYMHKKNIIHRDIKPENILIGFNNVIKLTDFGWSIINPPENRRKTVCGTIDYLSPEM
gi|2979623|gb|AAC12683.1|/1-345	DALTYCHKKKVIHRDIKPENLLLGLQGELKIADFGWSVHAP-SLRRKTMCGTLDYLPPEM

```
                                        ** * * . ::***:**** ::   . :*: ****:        * * ***::**.**:

XP_020952967.1/1-297                    IE----GRTYNEKVDLWCIGVLCYELLVGNPPFESAS-HNETYRRILKVDVRFPPS--LP
XP_012380002.1/1-290                    IE----GRTYDEKVDLWCIGVLCYELLMGSPPFESAS-HSETYRRILKVDVRFPSS--MP
XP_006102660.1/1-339                    IE----GRTHNEKVDLWCIGVLCYELLVGNPPFESAS-HNETYRRIVKVDLKFPPS--VP
XP_005673091.1/1-402                    IE----GRMHDEKVDLWSLGVLCYEFLVGKPPFEANT-YQETYKRISRVEFTFPDF--VP
XP_004484680.1/1-405                    IE----GRMHDEKVDLWSLGVLCYEFLVGKPPFEANT-YQETYNRISRVEFTFPDF--VT
XP_004462482.1/1-349                    IE----GRTHNEKVDLWCIGVLCYELLVGNPPFESAS-HNETYRRIVKVDLKFPPS--MP
Q6NW76.1/1-320                          IE----GHSHDEKVDLWSIGVLCYECLVGNPPFETAS-HAETYKRITKVDLQFPKL--VS
NP_999084.2/1-344                       IE----GRTHNEKVDLWCIGVLCYELLVGNPPFESAS-HNETYRRIVKVDLKFPPS--VP
gi|1190438012|ref|XP_020822491.1|/1-365 IE----GRTHDEKVDLWCIGVLCYELLVGNPPFESSS-HSETYRRITKVDLKFPSS--MP
gi|1190373986|ref|XP_020837071.1|/1-405 IE----GRMHDEKVDLWSLGVLCYEFLVGKPPFEAET-YQETYRSISKVEYKFPDF--VT
gi|1154067554|ref|NP_001153906.2|/1-332 IE----GHTHSEKVDLWCIGVLCFECLVGNPPFETAS-HTDTYKRIMKVDLNFPKV--VS
gi|1131237831|ref|XP_019799799.1|/1-344 IE----GRTHNEKVDLWCIGVLCYELLVGNPPFESAS-HNETYRRIVKVDLKIPPS--MP
gi|1131178530|ref|XP_019784431.1|/1-296 IE----GRTYNEKVDLWCIGVLCYELLVGNPPFESPS-HNETYRRILQVDVRFPPS--MP
gi|1126479796|ref|XP_006940862.2|/1-308 IE----RRTYNETVDLWCIGVLCYELLVGNPPFESHS-HNETYRRILKVDIRFPPS--IP
gi|1113784803|ref|XP_019353949.1|/1-376 IE----GQPHNEKVDLWCAGVLCYELLVGHPPFESAS-HTETYRRITKVDLRFPPT--MP
gi|1022987698|ref|XP_016286291.1|/1-365 IE----GRTHDEKVDLWCIGVLCYELLVGNPPFESSS-HSETYRRITKVDLKFPSS--MP
gi|966994491|ref|XP_014974040.1|/1-344  IE----GRTHNEKVDLWCIGVLCYELLVGNPPFESAS-HNETYRRIVKVDLKFPAS--VP
gi|966965902|ref|XP_015004225.1|/1-398  IE----GRMHDEKVDLWSLGVLCYEFLVGKPPFEANT-YQETYKRISRVEFTFPDF--VT
gi|955535802|ref|XP_004439167.2|/1-335  IE----GRTYNEKVDLWCIGVLCYELLVGNPPFESNS-HNETYRRILKVDVRFPPS--IP
gi|951053762|ref|XP_014465099.1|/1-416  IE----GRTHDEKVDIWSLGVLCYEFLVGKPPFEAET-YQETYRCISKVEFRYPPF--LS
gi|946796851|ref|XP_014384184.1|/1-404  IE----GRMHDEKVDLWSLGVLCYEFLVGKPPFEAST-YQETYKRISRVEFTFPDF--VR
gi|946756071|ref|XP_014392719.1|/1-299  VE----QRTYSEMVDLWCIGVLCYELLVGNPPFKSSS-YSETYRRILKVDVKFPPS--IP
gi|922383691|ref|XP_013462066.1|/1-300  VE----SVEHDASVDIWSLGVLCYEFLYGVPPFEAKE-HSDTYRRIIQVDLKFPPKPIVS
gi|909794683|ref|XP_013153467.1|/1-419  IE----GRTHDEKVDIWSLGVLCYEFLXGKPPFEAET-YQETYRAISRVEFKFPPF--VT
gi|891599507|ref|XP_013025412.1|/1-351  VE----GKEHTEKVDLWSLGVLTYEFIVGAPPFEDVSGHSATYKRIAKVDLKIPSF--VS
gi|891565640|ref|XP_013019059.1|/1-351  VE----GKEHTEKVDLWSLGVLTYEFIVGAPPFEDVSGHSATYRRIAKVDLKIPSF--VS
gi|884853217|ref|XP_013010870.1|/1-315  VM----GSTYDEKVDLWCVGVLCYELLVGSPPFESPS-HSETYRRILKVDMRFPLS--MP
gi|830244266|ref|XP_012590158.1|/1-296  IE----GRTYNEKVDLWCIGVLCYELLVGKPPFESST-HNETYRRILKVDVRFPPS--VP
gi|828211890|ref|XP_012558808.1|/1-339  IE----GKVHDEKVDLWSVGVLCYEFLVGKPPFETES-HDHTYQLISSVKYSFPDY--VS
gi|820033943|ref|NP_001295465.1|/1-282  IE----GKSYNETVDLWCIGVLCYELLVGKPPFESST-SSETCRRICQVDFRFPSS--MP
gi|795377289|ref|XP_011750175.1|/1-471  IE----GRTYDEKVDLWCIGVLCYELLVGYPPFESTS-HSETYRRILKVDVRFPLS--MP
gi|779989227|ref|XP_011666707.1|/1-321  IE----GRMHDDKVDLWSLGVLCYEFLVGKPPFEAEG-STETYRRITKVHYQFPSY--VS
gi|761905244|ref|XP_011402440.1|/1-385  VE----EKVHDEKVDLWSLGVLCYEFLVGKPPFETVN-TQDTYRKIVNVDFKFP-SY-VS
gi|759175630|ref|XP_011377486.1|/1-303  IE----GRTYNEKVDLWCIGVLCYELLVGSPPFESTS-HSETCRRILKVDVRFPPS--FP
gi|759149200|ref|XP_011368486.1|/1-311  IE----GRMHDEKVDLWSLGVLCYEFLVGKPPFEAST-YQETYKRISRVEFTFPDF--VP
gi|759116391|ref|XP_011357226.1|/1-339  IE----GRTHNEKVDLWCIGVLCYELLVGNPPFESAS-HNETYRRIVKVDLKFPTS--VP
gi|758991346|ref|XP_957232.2|/1-420     IRSGSKDNWYNEKVDLWSLGVLTYEFLVGEAPFEDTP--IMTQKRIARADMTIPEW--VS
gi|743922878|ref|XP_011005517.1|/1-293  VQ----SVEHDASVDIWSLGVLCYEFLYGIPPFEAKE-HSDTYRRIVQVDLKFPPKPIVS
gi|698438703|ref|XP_009757361.1|/1-296  VE----SVEHDASVDIWSLGILCFEFLYGMPPFEAKE-HSDTYRRIVQVDLKFPAKPIVS
gi|697188794|ref|XP_009603442.1|/1-294  VE----SVEHDANVDIWSLGILCYEFLYGVPPFEAKE-HSDTYRRIVQVDLKFPSRPVVS
gi|658871623|ref|XP_008418750.1|/1-330  IE----GHTHSEKVDLWCIGVLCYECLVGNPPFETAS-HSETYKRITKVDLNFPKV--IS
gi|657559162|ref|XP_008283516.1|/1-424  IE----GKTHDEKVDLWSLGVLCFEFLVGKPPFEAKT-HEETYRRISRVEYTYPAQTNIS
gi|657529148|ref|XP_008293493.1|/1-319  IE----GHTHSEKVDLWCIGVLCYECLVGNPPFETAS-HSETYKRIMKVDLKFPKV--IS
gi|655887082|ref|XP_008247187.1|/1-292  IE----GRTYDEKVDLWCIGVLCYELLVGNPPFESVS-HTETYRRILKVDVKFPVT--MS
gi|655881324|ref|XP_008272369.1|/1-402  IE----GRMHDEKVDLWSLGVLCYEFLVGKPPFEANT-YQETYRRISRVEFTFPDF--VT
gi|655868725|ref|XP_008268971.1|/1-343  IE----GRTHNEKVDLWCIGVLCYELLVGNPPFESAS-HNETYRRIVKVDLKFPAS--VP
gi|641774253|ref|XP_008170712.1|/1-431  IE----GRTHDEKVDLWSLGVLCYEFLVGKPPFEAET-YQETYRSISKVEFRFPPF--VS
gi|620969612|ref|XP_007654691.1|/1-303  IE----GRMHDEKVDLWSLGVLCYEFLVGKPPFEAET-YQETYRSISKVEYKFPGF--VT
gi|617451788|ref|XP_007568855.1|/1-331  IE----GHTHSEKVDLWCIGVLCYECLVGNPPFETAS-HSETYKRITKVDLNFPKV--IS
gi|594674988|ref|XP_007185333.1|/1-402  IE----GRMHDEKVDLWSLGVLCYEFLVGKPPFEANT-YQETYKRISRVEFTFPDF--VP
gi|594654199|ref|XP_007175509.1|/1-282  IE----GRTYNEKVDLWCIGVLCYELLVGNPPFESPS-HNETYRRILKVDVRFPPS--MP
gi|594623572|ref|XP_007166415.1|/1-344  IE----GRTHNEKVDLWCIGVLCYELLVGNPPFESAS-HNETYRRIVKVDLKFPPS--MP
gi|584038001|ref|XP_006753367.1|/1-368  IE----QRTYSEMVDLWCIGVLCYELLVGNPPFESTS-YSETYRRILKVDVKFPPS--IP
gi|573912300|ref|XP_006643864.1|/1-292  VE----KTEHDNHVDIWSLGILCYEFLYGAPPFEAKE-HSETYRRIVKVDLKFPPKPFVS
gi|554569173|ref|XP_005878848.1|/1-339  IE----GRTHNEKVDLWCIGVLCYELLVGNPPFESAS-HNETYRRIVKVDLKFPPS--VP
gi|545541126|ref|XP_005635254.1|/1-405  IE----GRMHDEKVDLWSLGVLCYEFLVGKPPFEAST-YQETYKRISRVEFTFPDF--VP
gi|545487136|ref|XP_005616134.1|/1-307  IE----RRTYNEKVDLWCIGVLCYELLVGYPPFESPS-HNETYRRILKVDLRFPPS--LP
gi|544514002|ref|XP_005590555.1|/1-471  IE----GRTYDEKVDLWCIGVLCYELLVGYPPFESTS-HSETYRRILKVDVRFPLS--MP
gi|530653727|ref|XP_005313062.1|/1-365  IE----GKTHDEKVDLWCIGVLCYELLVGHPPFESST-HTETYRRITKVDLQFPPF--MS
gi|528993703|ref|XP_005219854.1|/1-303  IE----GRTYDEKVDLWCIGVLCYELLVGNPPFESAS-TSETYRRILKVDLRFPPS--MS
gi|524890835|ref|XP_005101959.1|/1-332  VE----GKYHNECVDLWSLGVLCYEFLVGNPPFEAEG-HNETYRRIVKVDLVFPDH--VS
gi|514776534|ref|XP_004968502.1|/1-292  VE----KTEHDYHVDIWSLGILCYEFLYGVPPFEAKE-HSETYRRIVKVDLKFPLKPFVS
gi|507960369|ref|XP_004686961.1|/1-405  IE----GRMHDEKVDLWSLGVLCYEFLVGKPPFEAST-YQETYKRISRVEFTFPDF--VP
gi|507953708|ref|XP_004684672.1|/1-344  IE----GRTHNEKVDLWCIGVLCYELLVGSPPFESAS-HNETYRRIVKVDLKFPLS--VP
gi|478517838|ref|XP_004433150.1|/1-344  IE----GRTHNEKVDLWCIGVLCYELLVGNPPFESAS-HNETYRRIVKVDLKFPAS--VP
gi|478512086|ref|XP_004430306.1|/1-405  IE----GRMHDEKVDLWSLGVLCYEFLVGKPPFEAST-YQETYKRISRVEFTFPDF--VP
gi|470628341|ref|XP_004321082.1|/1-402  IE----GRMHDEKVDLWSLGVLCYEFLVGKPPFEANT-YQETYKRISRVEFTFPDF--VP
```

```
gi|470261727|ref|XP_004360485.1|/1-457  VN----KQHYDHKVDVWSLGILIYEFLCGRSAFCDET-EEAVMEKIQKGVVTFP--SYVS
gi|449486478|ref|XP_002195961.2|/1-319  IE----GRTHDEKVDIWSLGVLCYEFLVGKPPFETET-YQETYRAISRVEFKFPPF--VT
gi|426243291|ref|XP_004015492.1|/1-303  IE----GRTYDEKVDLWCIGVLCYELLVGNPPFESAS-TSETYRRILKVDLRFPPS--MS
gi|426237569|ref|XP_004012730.1|/1-344  IE----GRTHNEKVDLWCIGVLCYELLVGNPPFESAS-HNETYRRIVKVDLKFPPS--VP
gi|410979749|ref|XP_003996244.1|/1-344  IE----GRTHNEKVDLWCIGVLCYELLVGNPPFESAS-HNETYRRIVKVDLKFPPS--VP
gi|410953494|ref|XP_003983405.1|/1-405  IE----GRMHDEKVDLWSLGVLCYEFLVGKPPFEAST-YQETYKRISRVEFTFPDF--VP
gi|410914403|ref|XP_003970677.1|/1-332  IE----GHTHSEKVDLWCIGVLCYECLVGNPPFETTS-HSETYKRITKVDLKFPKV--VS
gi|389641901|ref|XP_003718583.1|/1-397  IKSGNKDNTYDEKVDLWSLGVLTYEFVVGEAPFEDTP--VMTQRRIARADMTIPSF--VS
gi|389635857|ref|XP_003715581.1|/1-400  IKPRTADKSYDHRVDLWALGVLTYELLVGEAPFEDTP--AMTHKKITRRDMKVPSF--VS
gi|357126095|ref|XP_003564724.1|/1-291  VE----KTEHDYHVDIWSLGILCYEFLYGLPPFEAKE-HSETYRRIVKVDLKFPAKPFVS
gi|355703962|gb|EHH30453.1|/1-309       IE----GRTYDEKVDLWCIGVLCYELLVGYPPFESTS-HSETYRRILKVDVRFPLS--MP
gi|350538969|ref|NP_001233545.1|/1-403  IE----GRMHDEKVDLWSLGVLCYEFLVGKPPFEANT-YQETYKRISRVEFTFPDF--VT
gi|347966592|ref|XP_321274.5|/1-404     VQ----GQPHTKTVDLWSLGVLAYELLCGKAPFLATT-YEETYRKIMKVQYTVPPD--VT
gi|345800233|ref|XP_849906.2|/1-344     IE----GRMHNEKVDLWCIGVLCYELLVGNPPFESAS-HNETYRRIVKVDLKFPPS--VP
gi|344296517|ref|XP_003419953.1|/1-405  IE----GRMHDEKVDLWSLGVLCYEFLVGKPPFEAST-YQETYNRISRVEFTFPDL--VT
gi|344290468|ref|XP_003416960.1|/1-345  IE----GRMHNEKVDLWCIGVLCYELLVGNPPFESAS-HNETYRRIVKVDLKFPPS--VP
gi|344269722|ref|XP_003406697.1|/1-306  IE----GRTYDERVDLWCIGVLCYELLVGNPPFESPS-HTETYRRILKVDVRFPSS--MP
gi|338709975|ref|XP_001502047.3|/1-304  IE----GRTYNEKVDLWCIGVLCYELLVGNPPFESNS-HNETYRRILKVDVRFPPS--IP
gi|334312551|ref|XP_001378079.2|/1-402  IE----GRMHDEKVDLWSLGVLCYEFLVGKPPFEAET-YQETYRSISKVEYKFPDF--VT
gi|332848444|ref|XP_511856.3|/1-344     IE----GRTHNEKVDLWCIGVLCYELLVGNPPFESAS-HNETYRRIVKVDLKFPAS--VP
gi|330793523|ref|XP_003284833.1|/1-359  VE----NKGYDQTADVWSLGILIYEFLVGHSPFASDE-EQRIFNNIKENEVHYP--SAIS
gi|321461440|gb|EFX72472.1|/1-304       VE----GRSHDERVDLWTLGILCYEFLVGSPPFEEEK-QDLTYRRICKVDLKFPAH--LS
gi|308497987|ref|XP_003111180.1|/1-306  IV----GNHHSDAVDLWAIGVLCYEFLVGKPPFEHDK-QADTYSAIKVARFSYPDY--VK
gi|302801442|ref|XP_002982477.1|/1-288  VE----NKEHDASVDVWSLGVLCYEFLFGTPPFEAQK-LSDTYRRIVRVDLKFPIKPVIS
gi|302688103|ref|XP_003033731.1|/1-328  VE----QKEHNERVDHWALGVLTYEFLIGNPPFEDRDSVNNTYRRIASVDLRFPNH--MS
gi|297802712|ref|XP_002869240.1|/1-290  VE----SVEHDASVDIWSLGILCYEFLYGVPPFEAME-HSDTYRRIVQVDLKFPPKPIVS
gi|281427796|ref|NP_001164000.1|/1-322  VV----GQTHNEKVDLWSLGVLCFEFLTGKPPFEAPT-FEETYRNISKAYYKFPPY--VS
gi|268565413|ref|XP_002639437.1|/1-302  VN----GNSHSDAVDLWAIGVLCYEFLVGKPPFEHEN-QADTYSAIKAGRFTYPDF--VK
gi|260784959|ref|XP_002587531.1|/1-282  IE----GKMHDEKVDLWSLGVLCYEFLVGKPPFEAEG-HSETYRRISKVDLRFPPH--VT
gi|226499040|ref|NP_001148791.1|/1-296  VE----KAEHDYHVDIWSLGVLCYEFLYGVPPFEAKE-HSETYRRIVKVDLKFPLKPFVS
gi|224088986|ref|XP_002308590.1|/1-293  VE----SVEHDASVDIWSLGVLCYEFLYGVPPFEAKE-HSDTYRRIVQVDLKFPPKPIVS
gi|215983064|ref|NP_001135979.1|/1-405  IE----GRMHDEKVDLWSLGVLCYEFLVGKPPFEADT-YQETYRRISRVEFTFPDC--VP
gi|213404608|ref|XP_002173076.1|/1-346  VE----GKEHGAKVDLWSLGVLTYEFLVGAPPFEDISGHSATYRRIAKVDLKFPSF--IS
gi|198430178|ref|XP_002119314.1|/1-305  IE----AKDHDANVDLWTLGILCYEFLVGKPPFETKS-TQETYLRITSLKYSFPPH--VS
gi|196012778|ref|XP_002116251.1|/1-270  II----GDEHDDKVDLWSLGILCYEFLVGCPPFETKT-HEDTYKRIIKVDLKFPENK-LS
gi|168044897|ref|XP_001774916.1|/1-297  VE----GKEHDAGVDVWSLGILCFEFLYGTPPFEAKK-HSDTYKRIVRVDLRFPSKPVIS
gi|159468203|ref|XP_001692272.1|/1-292  VE----GKEHTTSVDNWSLGVLTYEFLFGGPPFEAPG-HQETYRRIVRVDIKFPDKPAVS
gi|158297402|ref|XP_317640.3|/1-307     VD----GKMYDDSVDQWCLGILCYEFLVGNPPFESQT-TQTTYDKIRRLDIVYPRH--MT
gi|156368106|ref|XP_001627537.1|/1-314  IE----GKEHDEKVDLWSIGVLCYEFLVGKPPFEAEG-HNETYRRISRVDLRFPSH--VS
gi|149734120|ref|XP_001489320.1|/1-404  IE----GRMHDEKVDLWSLGVLCYEFLVGKPPFEAST-YQETYKRISRVEFTFPDF--VP
gi|149724949|ref|XP_001504864.1|/1-344  IE----GRTHNEKVDLWCIGVLCYELLVGNPPFESAS-HNETYRRIVKVDLKFPPS--VP
gi|148225464|ref|NP_001085657.1|/1-368  IE----GKTHDEKVDLWCAGVLCYEFLVGMPPFDSPS-HSETHRRIVNVDLKFPPF--LS
gi|146172296|ref|XP_001018388.2|/1-360  VE----GNTHNYTVDIWSLGVLCYEFCTGQPPFETKS-YDQTYDRIKKVQFSFP--DYLS
gi|144226857|ref|NP_001016859.2|/1-360  IE----GKTHDEKVDLWCAGVLCFEFLVGMPPFDSPS-HTETHRRIVNVDLKFPPF--LS
gi|125524746|gb|EAY72860.1|/1-292       VE----KTEHDYHVDIWSLGILCYEFLYGVPPFEAKE-HSETYRRIVKVDLKFPLKPFVS
gi|115749607|ref|NP_898907.2|/1-344     IE----GRTHNEKVDLWCIGVLCYELLVGNPPFESAS-HNETYRRIVKVDLKFPPS--VP
gi|115455545|ref|NP_001051373.1|/1-309  IE----KKAHDHAVDNWTLGILCYEFLYGSPPFEAAE-QDDTLRRIVKVDLSFPSTPYVS
gi|115435032|ref|NP_001042274.1|/1-292  VE----KTEHDYHVDIWSLGILCYEFLYGVPPFEAKE-HSETYRRIVKVDLKFPLKPFVS
gi|84000031|ref|NP_001033117.1|/1-402   IE----GRMHDEKVDLWSLGVLCYEFLVGKPPFEADT-YQETYRRISRVEFTFPDC--VP
gi|83776600|ref|NP_004208.2|/1-344      IE----GRMHNEKVDLWCIGVLCYELLVGNPPFESAS-HNETYRRIVKVDLKFPAS--VP
gi|76253851|ref|NP_695208.2|/1-397      IE----GRMHDEKVDLWSLGVLCYEFLVGMPPFEAHT-YQETYRRISRVEFTFPDF--VT
gi|68013217|ref|NP_001018849.1|/1-355   VE----GKEHTEKVDLWSLGVLTYEFLVGAPPFEDMSGHSATYKRIAKVDLKIPSF--VP
gi|66815573|ref|XP_641803.1|/1-384      IE----KKGYDQTADVWSLGILIFEFLVGRSPFTSDE-EKNIFHNIQENDVYYP--SSIS
gi|62865639|ref|NP_001015878.1|/1-309   IE----GRTYDEKVDLWCIGVLCYELLVGYPPFESAS-HSETYRRILKVDVRFPLS--MP
gi|62857967|ref|NP_001016570.1|/1-415   IE----GRMHDEKVDLWSLGVLCYEFLVGKPPFETDT-HQETYRRISKVEFQYPSY--VS
gi|57525577|ref|NP_001003640.1|/1-405   IE----GKTHDEKVDLWSLGVLCYEFLVGRPPFETKS-HEETYRKISRVEFTYPAH--VS
gi|50759065|ref|XP_425725.1|/1-409      IE----GRTHDEKVDIWSLGVLCYEFLVGKPPFEAQT-YQETYRAISRVEFKYPLF--VT
gi|50303387|ref|XP_451635.1|/1-361      IK----SREYNNKVDVWALGVLTYELLVGSPPFEEDT-KEMTYRRILRCDLKFPLN--VS
gi|46358064|ref|NP_035627.1|/1-417      IE----GRMHDEKVDLWSLGVLCYEFLVGMPPFEAHT-YQETYRRISRVEFTFPDF--VT
gi|45198420|ref|NP_985449.1|/1-367      IR----SREYNEKVDVWALGVLTYELLVGSPPFEEES-KELTYKRILKRNLIFPDH--VD
gi|38327570|ref|NP_940838.1|/1-403      IE----GRMHDEKVDLWSLGVLCYEFLVGKPPFEANT-YQETYKRISRVEFTFPDF--VT
gi|17505246|ref|NP_491714.1|/1-305      VN----GADHSDAVDLWAIGVLCYEFLVGKPPFEHED-QSKTYAAIKAARFTYPDS--VK
gi|17137514|ref|NP_477336.1|/1-329      VD----GNSYDDSVDQWCLGILCYEFVVGCPPFESNS-TESTYSKIRRMEISYPSH--LS
gi|17136516|ref|NP_476749.1|/1-411      VQ----GKPHTKNVDLWSLGVLCFELLVGHAPFYSKN-YDETYKKILKVDYKLPEH--IS
gi|16758580|ref|NP_446201.1|/1-343      IE----GRMHNEMVDLWCIGVLCYELMVGNPPFESPS-HSETYRRIVKVDLKFPSS--MP
gi|15233958|ref|NP_195009.1|/1-294      VE----SVEHDASVDIWSLGILCYEFLYGVPPFEAME-HSDTYRRIVQVDLKFPPKPIIS
gi|15225495|ref|NP_182073.1|/1-288      VE----NRDHDYAVDNWTLGILCYEFLYGNPPFEAES-QKDTFKRILKIDLSFPLTPNVS
gi|6708213|gb|AAF25838.1|/1-282         IA----QKPYNEMVDLWCIGVLCYELLVGKPPFESST-SSETYRRIRQVDFKFPSS--VP
gi|6325047|ref|NP_015115.1|/1-367       VE----SREYDHTIDAWALGVLAFELLTGAPPFEEEM-KDTTYKRIAALDIKMPSN--IS
gi|2979623|gb|AAC12683.1|/1-345         IE----GRMHNEMVDLWCIGVLCYELMVGNPPFESPS-HSETYRRIVKVDLKFPSS--VP
```

```
                                        :        :    * *  *:* :*   * ..*            *       *    .

XP_020952967.1/1-297                    LGAQDLIS------------------------------RLLRYQPSDRLPLDQILEHPWV
XP_012380002.1/1-290                    PGAQDLIS------------------------------KLLRYQPSERLPLAQILTHPWV
XP_006102660.1/1-339                    VGAQDLIS------------------------------KLLKHNPSDRLPLAQVSAHPWV
XP_005673091.1/1-402                    EGARDLIS------------------------------RLLKHNPSHRPTLKEVLEHPWI
XP_004484680.1/1-405                    EGARDLIS------------------------------RLLKHNPSQRPTLKEVLEHPWI
XP_004462482.1/1-349                    PGAQDLIS------------------------------KLLKHNPSERLPLAQVAAHPWV
Q6NW76.1/1-320                          EGARDLIS------------------------------KLLRHSPSMRLPLRSVMEHPWV
NP_999084.2/1-344                       AGAQDLIS------------------------------KLLKHNPSDRLPLAQVSAHPWV
gi|1190438012|ref|XP_020822491.1|/1-365 SGAQDLIS------------------------------KLLRHNPSERLPLTQVLAHPWV
gi|1190373986|ref|XP_020837071.1|/1-405 EGARDLIS------------------------------RLLKHNPYQRLTLKELLEHPWI
gi|1154067554|ref|NP_001153906.2|/1-332 DGARDLIS------------------------------KLLRHNPNDRLPLQKVMDHPWV
gi|1131237831|ref|XP_019799799.1|/1-344 AGAQDLIS------------------------------KLLKHNPSERLPLAQVAAHPWV
gi|1131178530|ref|XP_019784431.1|/1-296 LGAQDLIS------------------------------KLLRYQPSERLPLVQILEHPWV
gi|1126479796|ref|XP_006940862.2|/1-308 LGARDLIS------------------------------KLLRYQPLERLPLSQILEHPWV
gi|1113784803|ref|XP_019353949.1|/1-376 EGARDLIG------------------------------KLLRHVPAERLPLRAVLEHPWV
gi|1022987698|ref|XP_016286291.1|/1-365 SGAQDLIS------------------------------KLLRHNPSERLPLTQVLEHPWV
gi|966994491|ref|XP_014974040.1|/1-344  TGAQDLIS------------------------------KLLRHNPSERLPLAQVSAHPWV
gi|966965902|ref|XP_015004225.1|/1-398  EGARDLIS------------------------------RLLKHNPSQRPMLREVLEHPWI
gi|955535802|ref|XP_004439167.2|/1-335  LGAKDLIS------------------------------KLLRYQPLERLPLDQIMEHPWV
gi|951053762|ref|XP_014465099.1|/1-416  EGAKDLIS------------------------------KLLKHNPYHRLPLKDVLEHPWI
gi|946796851|ref|XP_014384184.1|/1-404  EGARDLIS------------------------------RLLMHNPNQRLTLKEVLEHPWI
gi|946756071|ref|XP_014392719.1|/1-299  LGAQDLIS------------------------------KLLRYQPSERLLLAQVLQHPWV
gi|922383691|ref|XP_013462066.1|/1-300  SAAKDLIS------------------------------QMLVKDSSERLPLHKLLEHPWI
gi|909794683|ref|XP_013153467.1|/1-419  EGARDLIA------------------------------KLLKHNPFHRLPLKDVLLHPWI
gi|891599507|ref|XP_013025412.1|/1-351  PEAKDLIT------------------------------RLLRHSPEQRISLEQVVKHPWM
gi|891565640|ref|XP_013019059.1|/1-351  PEGKDLIS------------------------------RLLRHSPEQRISLEQVVKHPWI
gi|884853217|ref|XP_013010870.1|/1-315  AGAQDLIS------------------------------RLLRFQPMERLPLSQVAVHPWV
gi|830244266|ref|XP_012590158.1|/1-296  LGAQDLIS------------------------------SLLRYQPSERLPLAQILQHPWV
gi|828211890|ref|XP_012558808.1|/1-339  SGARDLIS------------------------------QLLRKKPSDRLPLADVLKHPWI
gi|820033943|ref|NP_001295465.1|/1-282  AGAQDLIS------------------------------KLLRHHPSERLSLAQVLKHPWV
gi|795377289|ref|XP_011750175.1|/1-471  LGARDLIS------------------------------KLLRYQPLERLPLAQILKHPWV
gi|779989227|ref|XP_011666707.1|/1-321  AGARDVIK------------------------------RLLQHNPANRLPLEQVLAHPWI
gi|761905244|ref|XP_011402440.1|/1-385  PGAKDLII------------------------------KLLKKNPSERLALDEVLKHSWI
gi|759175630|ref|XP_011377486.1|/1-303  LGAQNLIS------------------------------KLLRYQPLERLPLAQILQHPWV
gi|759149200|ref|XP_011368486.1|/1-311  EGARDLIS------------------------------RLLKHNPSQRPTLKEVLEHPWI
gi|759116391|ref|XP_011357226.1|/1-339  TGAQDLIS------------------------------KLLKHNPSERLPLAQVSAHPWV
gi|758991346|ref|XP_957232.2|/1-420     KEAKDLIK------------------------------KLLVLDPEKRLPLEEVENHPWI
gi|743922878|ref|XP_011005517.1|/1-293  SAAKDLIS------------------------------QMLVKDSVERLPLHKLLEHPWI
gi|698438703|ref|XP_009757361.1|/1-296  SAAKDLIS------------------------------QMLVKDSSQRLPLKKVLEHPWI
gi|697188794|ref|XP_009603442.1|/1-294  SAARDLIS------------------------------QMLVKDSSQRLALKKLLEHPWI
gi|658871623|ref|XP_008418750.1|/1-330  DGARDLIS------------------------------KLLRHNPIDRLPLQNVIGHPWV
gi|657559162|ref|XP_008283516.1|/1-424  AGAKDLVA------------------------------RLLKHNPMHRLPIEGVLSHPWV
gi|657529148|ref|XP_008293493.1|/1-319  DGARDLIS------------------------------KLLRHNPIDRLSLQSVIDHPWV
gi|655887082|ref|XP_008247187.1|/1-292  LGARDLIS------------------------------RLLRYQPSERLPLAQILRHPWV
gi|655881324|ref|XP_008272369.1|/1-402  EGAKDFIS------------------------------RLLKHNPSHRPTLAEVLEHPWV
gi|655868725|ref|XP_008268971.1|/1-343  AGAQDLIS------------------------------KLLRHNPSDRLTLAQVSAHPWV
gi|641774253|ref|XP_008170712.1|/1-431  EGAKDLIV------------------------------KLLKHNPYQRLPLKDVLAHPWI
gi|620969612|ref|XP_007654691.1|/1-303  DGAKDLIS------------------------------KLLKHNPYQRLTLKEVLEHPWI
gi|617451788|ref|XP_007568855.1|/1-331  DGARDLIS------------------------------KLLRHNPIDRLPLQNVIDHPWV
gi|594674988|ref|XP_007185333.1|/1-402  EGARDLIS------------------------------RLLKHNPSQRPTLKEVLEHPWI
gi|594654199|ref|XP_007175509.1|/1-282  LGAQDLIS------------------------------KLLRYQPSERLPLVQILEHPWV
gi|594623572|ref|XP_007166415.1|/1-344  AGAQDLIS------------------------------KLLKHNPSERLPLAQVSAHPWV
gi|584038001|ref|XP_006753367.1|/1-368  LGAQDLIS------------------------------KLLRYQPSERLPLAQVLQHPWV
gi|573912300|ref|XP_006643864.1|/1-292  PSAKDLIS------------------------------QMLVKNSAHRLPLHKLLEHPWI
gi|554569173|ref|XP_005878848.1|/1-339  VGAQDLIS------------------------------KLLKHNPSDRLPLAQVSAHPWV
gi|545541126|ref|XP_005635254.1|/1-405  EGARDLIS------------------------------RLLKHNPSQRPTLKDVLEHPWI
gi|545487136|ref|XP_005616134.1|/1-307  LGAQDLVS------------------------------KLLRYQPLERLPLAQIMEHPWV
gi|544514002|ref|XP_005590555.1|/1-471  LGARDLIS------------------------------KLLRYQPLERLPLAQILKHPWV
gi|530653727|ref|XP_005313062.1|/1-365  EGSRDLIT------------------------------KLLCHNPAERLPLRAVLEHPWV
gi|528993703|ref|XP_005219854.1|/1-303  SGARDLIS------------------------------KLLRFQPLERLPLVRVLEHPWI
gi|524890835|ref|XP_005101959.1|/1-332  DGARDLIS------------------------------KLLRHDPKARLPLEQVLKHPWI
gi|514776534|ref|XP_004968502.1|/1-292  PAAKDLIS------------------------------QMLVKNSAHRLPLHKVLEHPWI
gi|507960369|ref|XP_004686961.1|/1-405  EGARDFIS------------------------------RLLKHNPSQRPTLKEVLEHPWI
gi|507953708|ref|XP_004684672.1|/1-344  SGAQDLIT------------------------------KLLKHNPSERLPLTQVSAHPWV
gi|478517838|ref|XP_004433150.1|/1-344  AGAQDLIS------------------------------KLLKHNPSERLPLAQVSAHPWV
gi|478512086|ref|XP_004430306.1|/1-405  DGARDLIS------------------------------RLLKHNPSQRPTLKEVLEHPWI
gi|470628341|ref|XP_004321082.1|/1-402  EGARDLIS------------------------------RLLKHNPSQRPTLKEVLEHPWI
```

```
gi|470261727|ref|XP_004360485.1|/1-457  MEAQHLIL------------------------------GLLTSDPSERLTMYDVINHPWI
gi|449486478|ref|XP_002195961.2|/1-319  EGARDLIS------------------------------RLLKHNPFHRLPLKDVLLHPWI
gi|426243291|ref|XP_004015492.1|/1-303  SGARDLIS------------------------------KLLRFQPLERLPLVRVLEHPWI
gi|426237569|ref|XP_004012730.1|/1-344  MGAQDLIS------------------------------KLLKHNPSERLPLAQVSAHPWV
gi|410979749|ref|XP_003996244.1|/1-344  TGAQDLIS------------------------------KLLKHNPSERLPLAQVSAHPWV
gi|410953494|ref|XP_003983405.1|/1-405  EGARDLIS------------------------------RLLKHNPSQRPTLKEVLEHPWV
gi|410914403|ref|XP_003970677.1|/1-332  DGARDLIS------------------------------KLLRHNPIDRLTLQNVIDHPWV
gi|389641901|ref|XP_003718583.1|/1-397  PECRDLIK------------------------------KLLVLDPVKRLSLDAVQKHPWI
gi|389635857|ref|XP_003715581.1|/1-400  REASDLIL------------------------------KLLVVDPNKRLPLDKVLDHPWI
gi|357126095|ref|XP_003564724.1|/1-291  PAAKDLIS------------------------------QMLVKNSAHRLPLNKVLEHPWI
gi|355703962|gb|EHH30453.1|/1-309       LGARDLIS------------------------------KLLRYQPLERLPLAQILKHPWV
gi|350538969|ref|NP_001233545.1|/1-403  EGARDLIS------------------------------RLLKHNPSQRPMLREVLEHPWI
gi|347966592|ref|XP_321274.5|/1-404     KAASHLIS------------------------------RLLVKDPASRMPLENVAIHPWI
gi|345800233|ref|XP_849906.2|/1-344     TGAQDLIS------------------------------KLLKHNPSERLPLSQVSAHPWV
gi|344296517|ref|XP_003419953.1|/1-405  VGARDLVS------------------------------RLLKHNPSQRLTLKEVLEHPWI
gi|344290468|ref|XP_003416960.1|/1-345  TGAQDLIS------------------------------KLLKHNPSERLPLAQVSAHPWV
gi|344269722|ref|XP_003406697.1|/1-306  SGAQDLIS------------------------------KLLRYQPFERLALAQVLEHPWV
gi|338709975|ref|XP_001502047.3|/1-304  SGAKDLIS------------------------------KLLRYQPLERLPLDQILEHPWV
gi|334312551|ref|XP_001378079.2|/1-402  EGARDLIS------------------------------RLLKHNPYQRLTLKELLEHPWI
gi|332848444|ref|XP_511856.3|/1-344     TGAQDLIS------------------------------KLLRHNPSERLPLAQVSAHPWV
gi|330793523|ref|XP_003284833.1|/1-359  TDAKDLIS------------------------------KLLISDPHQRISLKDVINHPWI
gi|321461440|gb|EFX72472.1|/1-304       TGAKDLIT------------------------------KLLKSRAEDRIPLRKLLEHSWI
gi|308497987|ref|XP_003111180.1|/1-306  KGARDLIG------------------------------KLLVVDPTKRCTLQKVKDHYWV
gi|302801442|ref|XP_002982477.1|/1-288  TAAKDLIC------------------------------QLLCKDSSQRLPLKELLAHPWI
gi|302688103|ref|XP_003033731.1|/1-328  SDARDLIS------------------------------KLLRHDPQDRISLAEVMKHPWI
gi|297802712|ref|XP_002869240.1|/1-290  ASAKDLIS------------------------------QMLVKESSQRLPLHKLLEHPWI
gi|281427796|ref|NP_001164000.1|/1-322  EGAQDLIR------------------------------KLLVVNPNERLELSQVMKHPWI
gi|268565413|ref|XP_002639437.1|/1-302  KGARDLIG------------------------------KLLVVDPRRRCSLQEVKDHYWV
gi|260784959|ref|XP_002587531.1|/1-282  SGARDLIS------------------------------KLLRHNPMLRLPLDSVLSHPWI
gi|226499040|ref|NP_001148791.1|/1-296  PAAKDLIS------------------------------QMLVKSSAQRLPLHKVLEHPWI
gi|224088986|ref|XP_002308590.1|/1-293  SAAKDLIS------------------------------QMLVKDSAERLPLHKLLDHPWI
gi|215983064|ref|NP_001135979.1|/1-405  EGARDLIS------------------------------RLLKHNPSQRPTLKEVLEHPWI
gi|213404608|ref|XP_002173076.1|/1-346  AEAKDLIS------------------------------RLLQHSPEKRLSLDQVMRHPWI
gi|198430178|ref|XP_002119314.1|/1-305  EGARDLIR------------------------------RLLKLEPRHRLPLESVMAHPWI
gi|196012778|ref|XP_002116251.1|/1-270  PEAMDLIR------------------------------KLLCRKPSERLSLDGVMSHPWI
gi|168044897|ref|XP_001774916.1|/1-297  SSARDLIC------------------------------QLLVKDSAQRLPLSKVLTHPWI
gi|159468203|ref|XP_001692272.1|/1-292  DSAKAFIS------------------------------KLLVKDAHRRLPLLEVQNDPWI
gi|158297402|ref|XP_317640.3|/1-307     AGAINLIS------------------------------KLLRIPSSSRITLRDVMNHPWV
gi|156368106|ref|XP_001627537.1|/1-314  PGARDLIS------------------------------KLLQHNPNNRLPLKAVLNHPWI
gi|149734120|ref|XP_001489320.1|/1-404  EGARDLIS------------------------------RLLKHNPSQRPTLKEVLEHPWI
gi|149724949|ref|XP_001504864.1|/1-344  AGAQDLIS------------------------------KLLRHNPSERLPLAQVSAHPWV
gi|148225464|ref|NP_001085657.1|/1-368  EGSKDLIS------------------------------KLLRYHPAQRLPLKGVMEHPWV
gi|146172296|ref|XP_001018388.2|/1-360  IEVRDLLS------------------------------KILVYEKSQRLDLNNILNHPWI
gi|144226857|ref|NP_001016859.2|/1-360  DGSKDLIS------------------------------KLLRYHPPQRLPLKGVMEHPWV
gi|125524746|gb|EAY72860.1|/1-292       PAAKDLIS------------------------------QMLVKNSAHRLPLHKLLEHPWI
gi|115749607|ref|NP_898907.2|/1-344     LGAQDLIY------------------------------KLLKHNPSERLPLAQVSAHPWV
gi|115455545|ref|NP_001051373.1|/1-309  ADAKDLICKVKFVVLIIPIVYVTLKWVELLSFELRVRMQLLVKDSNKRLSLDDIMKHPWI
gi|115435032|ref|NP_001042274.1|/1-292  PAAKDLIS------------------------------QMLVKNSAHRLPLHKLLEHPWI
gi|84000031|ref|NP_001033117.1|/1-402   EGARDLIS------------------------------RLLKHNPSQRPTLKEVLEHPWI
gi|83776600|ref|NP_004208.2|/1-344      MGAQDLIS------------------------------KLLRHNPSERLPLAQVSAHPWV
gi|76253851|ref|NP_695208.2|/1-397      EGARDLIS------------------------------RLLKHNSSQRLTLAEVLEHPWI
gi|68013217|ref|NP_001018849.1|/1-355   PDARDLIS------------------------------RLLQHNPEKRMSLEQVMRHPWI
gi|66815573|ref|XP_641803.1|/1-384      PEAKDLIS------------------------------RLLVSDPHQRITLKDVINHPWI
gi|62865639|ref|NP_001015878.1|/1-309   LGARDLIS------------------------------RLLRYQPLERLPLAQILKHPWV
gi|62857967|ref|NP_001016570.1|/1-415   EGARDLVS------------------------------KLLKHNPNHRLSLKGVLEHPWI
gi|57525577|ref|NP_001003640.1|/1-405   NGSRDLIN------------------------------RLLKHNPMHRLPIQGVMEHPWV
gi|50759065|ref|XP_425725.1|/1-409      EGARDLIS------------------------------KLLKHNPFHRLPLKDVLVHPWI
gi|50303387|ref|XP_451635.1|/1-361      PQARDLIV------------------------------RLLQFEPSKRIPLSEVKSHAWI
gi|46358064|ref|NP_035627.1|/1-417      EGARDLIS------------------------------RLLKHNASQRLTLAEVLEHPWI
gi|45198420|ref|NP_985449.1|/1-367      TEARHLIS------------------------------RLLEYDPGDRIPLKEVKKHPWI
gi|38327570|ref|NP_940838.1|/1-403      EGARDLIS------------------------------RLLKHNPSQRPMLREVLEHPWI
gi|17505246|ref|NP_491714.1|/1-305      KGARDLIG------------------------------RLLVVDPKARCTLEQVKEHYWI
gi|17137514|ref|NP_477336.1|/1-329      KGCKELIG------------------------------GLLRKESKGRITLVDVMTHYWV
gi|17136516|ref|NP_476749.1|/1-411      KAASHLIS------------------------------KLLVLNPQHRLPLDQVMVHPWI
gi|16758580|ref|NP_446201.1|/1-343      LGAKDLIS------------------------------KLLKHNPSQRLPLEQVSAHPWV
gi|15233958|ref|NP_195009.1|/1-294      ASAKDLIS------------------------------QMLVKESSQRLPLHKLLEHPWI
gi|15225495|ref|NP_182073.1|/1-288      EEAKNLIS------------------------------QLLVKDPSKRLSIEKIMQHPWI
gi|6708213|gb|AAF25838.1|/1-282         AGAQDLIS------------------------------KLLRYHPSERLSLAQVLKHPWV
gi|6325047|ref|NP_015115.1|/1-367       QDAQDLIL------------------------------KLLKYDPKDRMRLGDVKMHPWI
gi|2979623|gb|AAC12683.1|/1-345         SGAQDLIS------------------------------KLLKHNPWQRLPLAEVAAHPWV
```

```
                                            .:                       :*     *  :  :  . *:

XP_020952967.1/1-297                 RAHSRRVLPPSTQMVS------------
XP_012380002.1/1-290                 QAHSRRVLPPSAQMVS------------
XP_006102660.1/1-339                 RAHSRRVLPPSALQSVH-----------
XP_005673091.1/1-402                 TAN-SKPASSH-KKESTSKQP-------
XP_004484680.1/1-405                 TANSSKPPSSQKSKESTIQQS-------
XP_004462482.1/1-349                 RAHSRRVLPPSALQSVP-----------
Q6NW76.1/1-320                       KANSRRVLPPVCSSEPH-----------
NP_999084.2/1-344                    RAHSRRVLPPSAPQSVP-----------
gi|1190438012|ref|XP_020822491.1|/1-365 QTHSRRVLPPCSSRPAH-----------
gi|1190373986|ref|XP_020837071.1|/1-405 IANSSKSPSSRKSKESNSTKS-------
gi|1154067554|ref|NP_001153906.2|/1-332 RTNSHRLLPPTCPSKKC-----------
gi|1131237831|ref|XP_019799799.1|/1-344 RAHSRRVLPPSALQSVP-----------
gi|1131178530|ref|XP_019784431.1|/1-296 RAHSRRVLPPSAQMVS------------
gi|1126479796|ref|XP_006940862.2|/1-308 RAHSRRVVPPSVQMDS------------
gi|1113784803|ref|XP_019353949.1|/1-376 RANSRRVLPPAYAP--------------
gi|1022987698|ref|XP_016286291.1|/1-365 QTHSRRVLPPCSPQPAH-----------
gi|966994491|ref|XP_014974040.1|/1-344  RANSRRVLPPSALQSVA-----------
gi|966965902|ref|XP_015004225.1|/1-398  TANSSKPSNCQ-NRVN------------
gi|955535802|ref|XP_004439167.2|/1-335  RAHSRRVLPPSAQMAS------------
gi|951053762|ref|XP_014465099.1|/1-416  RENSTKSPASRVSEAAGSRKSEANNKT-
gi|946796851|ref|XP_014384184.1|/1-404  TANSSKPSSSQKSKESTSKQS-------
gi|946756071|ref|XP_014392719.1|/1-299  QAHSRRVGPPPAPSAS------------
gi|922383691|ref|XP_013462066.1|/1-300  VQNAEPSGIYRS----------------
gi|909794683|ref|XP_013153467.1|/1-419  TANSTKMPNSRKSDVAALPRTQS-----
gi|891599507|ref|XP_013025412.1|/1-351  LKHKASWSSDQ-----------------
gi|891565640|ref|XP_013019059.1|/1-351  LKFKASWNSDQ-----------------
gi|884853217|ref|XP_013010870.1|/1-315  KAHSRRVLPP------------------
gi|830244266|ref|XP_012590158.1|/1-296  RMHSRRVLPPSAQVAS------------
gi|828211890|ref|XP_012558808.1|/1-339  QQHSSMFNKQKVSVNQTPTTSVSSLSSS
gi|820033943|ref|NP_001295465.1|/1-282  RKHSQRMLPPCAQMAS------------
gi|795377289|ref|XP_011750175.1|/1-471  QVHSRRVLPPCAQMAS------------
gi|779989227|ref|XP_011666707.1|/1-321  VENSKKKPSSSSTSSSESQS--------
gi|761905244|ref|XP_011402440.1|/1-385  IENAK-----------------------
gi|759175630|ref|XP_011377486.1|/1-303  QAHSRRVLPPSAHMAS------------
gi|759149200|ref|XP_011368486.1|/1-311  TANSSKPSSSQQNKESSSKQS-------
gi|759116391|ref|XP_011357226.1|/1-339  RAHSRRVLPPSALQSVP-----------
gi|758991346|ref|XP_957232.2|/1-420     LKHCVKGERAANREKMASKMGN------
gi|743922878|ref|XP_011005517.1|/1-293  VQNVDPSGIFRG----------------
gi|698438703|ref|XP_009757361.1|/1-296  VQNADPSGVYKG----------------
gi|697188794|ref|XP_009603442.1|/1-294  VQNADPSGVYRG----------------
gi|658871623|ref|XP_008418750.1|/1-330  KANSRRFLPPTCLTKKP-----------
gi|657559162|ref|XP_008283516.1|/1-424  VQNS---TKKPITLNNEELSQ-------
gi|657529148|ref|XP_008293493.1|/1-319  RSNSHRLLPPTCPAKKS-----------
gi|655887082|ref|XP_008247187.1|/1-292  QAHSRRVLPPCAHPAS------------
gi|655881324|ref|XP_008272369.1|/1-402  KAKSSKPSSCP-DKEPASKQS-------
gi|655868725|ref|XP_008268971.1|/1-343  RAHSRRVLPPSAMQSVA-----------
gi|641774253|ref|XP_008170712.1|/1-431  TTNSTKQPSSRKSEAATSNRTPS-----
gi|620969612|ref|XP_007654691.1|/1-303  TANSSKAPNSLKSQESTSTKS-------
gi|617451788|ref|XP_007568855.1|/1-331  KANSRRLLPPTCPTKKP-----------
gi|594674988|ref|XP_007185333.1|/1-402  TAN-SKPSSSQ-KKESASKQS-------
gi|594654199|ref|XP_007175509.1|/1-282  RAHSRRVLPPSAQMVS------------
gi|594623572|ref|XP_007166415.1|/1-344  RAHSRRVLPPSALQSVP-----------
gi|584038001|ref|XP_006753367.1|/1-368  QAHSRRVEPPPAPSAS------------
gi|573912300|ref|XP_006643864.1|/1-292  VQNADPSGVYRG----------------
gi|554569173|ref|XP_005878848.1|/1-339  RAHSRRVLPPSALQSVH-----------
gi|545541126|ref|XP_005635254.1|/1-405  MANSSKPSSSQKNKDSTSKQS-------
gi|545487136|ref|XP_005616134.1|/1-307  RAHSRRVLPPSVQMAS------------
gi|544514002|ref|XP_005590555.1|/1-471  QVHSRRVLPPCAQMAS------------
gi|530653727|ref|XP_005313062.1|/1-365  KANSRRVLPPVFNPAPAN----------
gi|528993703|ref|XP_005219854.1|/1-303  RAHSQRVLPPSVPMAF------------
gi|524890835|ref|XP_005101959.1|/1-332  LKYNPDLASSSTSSAPSS----------
gi|514776534|ref|XP_004968502.1|/1-292  VQNADPSGVYRG----------------
gi|507960369|ref|XP_004686961.1|/1-405  TANSSKPPSSQKNKESTSKQS-------
gi|507953708|ref|XP_004684672.1|/1-344  RAHSRRVLPPSALQSVP-----------
gi|478517838|ref|XP_004433150.1|/1-344  RAHSRRVLPPSAPQSVP-----------
gi|478512086|ref|XP_004430306.1|/1-405  TANASKPSNSQKSKESTSKQS-------
gi|470628341|ref|XP_004321082.1|/1-402  TAN-SKPSSSQ-KKESASKQS-------
```

```
gi|470261727|ref|XP_004360485.1|/1-457  KKNAHPHSLIRT----------------
gi|449486478|ref|XP_002195961.2|/1-319  TANSTKIPTSRKSDAAAPSKT-------
gi|426243291|ref|XP_004015492.1|/1-303  RAHSQRVLPPSVQRAF------------
gi|426237569|ref|XP_004012730.1|/1-344  RAHSRRVLPPSAPQSVP-----------
gi|410979749|ref|XP_003996244.1|/1-344  RAHSRRVLPPSALQSVP-----------
gi|410953494|ref|XP_003983405.1|/1-405  TANSSKPSGSQKSKESTGKQS-------
gi|410914403|ref|XP_003970677.1|/1-332  RSNSRRVLPPICPAKKS-----------
gi|389641901|ref|XP_003718583.1|/1-397  IKHCVKGERATNREKGRGSD--------
gi|389635857|ref|XP_003715581.1|/1-400  VKHCRN-QKASDRLLPSTGNTPPED---
gi|357126095|ref|XP_003564724.1|/1-291  VQNADPSGVYRG----------------
gi|355703962|gb|EHH30453.1|/1-309       QVHSRRVLPPCAQMAS------------
gi|350538969|ref|NP_001233545.1|/1-403  TANSSKPSNCQ-NKESASKQS-------
gi|347966592|ref|XP_321274.5|/1-404     QLHVDKK---------------------
gi|345800233|ref|XP_849906.2|/1-344     RAHSRRMLPPSAVQAIP-----------
gi|344296517|ref|XP_003419953.1|/1-405  TANSKKPPSSQ-NKEPTSKQS-------
gi|344290468|ref|XP_003416960.1|/1-345  RAHSRRVLPPSALQSVP-----------
gi|344269722|ref|XP_003406697.1|/1-306  QAHSQRVLPPSAQMAS------------
gi|338709975|ref|XP_001502047.3|/1-304  RAHSRRVLPPSAQMAS------------
gi|334312551|ref|XP_001378079.2|/1-402  IANSSKSPSSRKSKESKP----------
gi|332848444|ref|XP_511856.3|/1-344     RANSRRVLPPSALQSVA-----------
gi|330793523|ref|XP_003284833.1|/1-359  QKNAHPHSLEPTKLGLPLPSQMTH----
gi|321461440|gb|EFX72472.1|/1-304       LQHLSDEVRSRLPTFQIMQPNKA-----
gi|308497987|ref|XP_003111180.1|/1-306  TQMVETARKVAEQAKAEKAASLRDH---
gi|302801442|ref|XP_002982477.1|/1-288  TANADPSGIYAGSGGASA----------
gi|302688103|ref|XP_003033731.1|/1-328  VKYRPKGQIRPSQLGM------------
gi|297802712|ref|XP_002869240.1|/1-290  VQNADPSGIYRG----------------
gi|281427796|ref|NP_001164000.1|/1-322  VSHTEEKSSQ------------------
gi|268565413|ref|XP_002639437.1|/1-302  TSMLDSCRRAAEKQKAERAASLRDH---
gi|260784959|ref|XP_002587531.1|/1-282  KDNAATQAESTPAHPTHASHTKS-----
gi|226499040|ref|NP_001148791.1|/1-296  VQNADPSGVYREGRSV------------
gi|224088986|ref|XP_002308590.1|/1-293  VQNADPSGIFRG----------------
gi|215983064|ref|NP_001135979.1|/1-405  TAN-SKPSSCQ-KKESTSKQS-------
gi|213404608|ref|XP_002173076.1|/1-346  LKHKNNWDRPGVDPQ-------------
gi|198430178|ref|XP_002119314.1|/1-305  KANA---KVHKFGPDGRPMD--------
gi|196012778|ref|XP_002116251.1|/1-270  VKHVQNDAKN------------------
gi|168044897|ref|XP_001774916.1|/1-297  VNNADPSGVYNGHN--------------
gi|159468203|ref|XP_001692272.1|/1-292  KANADPDMLARGN---------------
gi|158297402|ref|XP_317640.3|/1-307     --------VQMKK---------------
gi|156368106|ref|XP_001627537.1|/1-314  RMHATPVLEQKPASQR------------
gi|149734120|ref|XP_001489320.1|/1-404  TANSSKPSNSQKSKESTSKQP-------
gi|149724949|ref|XP_001504864.1|/1-344  RAHSRRVLPPSALQSVP-----------
gi|148225464|ref|NP_001085657.1|/1-368  KANSRRVLPPVYQSSHPK----------
gi|146172296|ref|XP_001018388.2|/1-360  QKHNPPQNQVTSQVPLQQLNQNIC----
gi|144226857|ref|NP_001016859.2|/1-360  KANSRRVLPPVFQSSSK-----------
gi|125524746|gb|EAY72860.1|/1-292       VQNADPSGVYRG----------------
gi|115749607|ref|NP_898907.2|/1-344     RTHSRRVLPPSAPQSVP-----------
gi|115455545|ref|NP_001051373.1|/1-309  VKNADPSGSCSDQKART-----------
gi|115435032|ref|NP_001042274.1|/1-292  VQNADPSGVYRG----------------
gi|84000031|ref|NP_001033117.1|/1-402   IAN-SKPSSCQ-KKESTSKQS-------
gi|83776600|ref|NP_004208.2|/1-344      RANSRRVLPPSALQSVA-----------
gi|76253851|ref|NP_695208.2|/1-397      KANSSKPPTGHNSKEATSKSS-------
gi|68013217|ref|NP_001018849.1|/1-355   VKYKDSWTRKSSESS-------------
gi|66815573|ref|XP_641803.1|/1-384      KKHAHPKSLEPTKLGLPLPSQMTY----
gi|62865639|ref|NP_001015878.1|/1-309   QAHSRRVLPPCAQMAS------------
gi|62857967|ref|NP_001016570.1|/1-415   IDNSAQKPKRKEGPVTGAQ---------
gi|57525577|ref|NP_001003640.1|/1-405   VENS---TKKPTTYTATANN--------
gi|50759065|ref|XP_425725.1|/1-409      TANSTKLPNNRK----------------
gi|50303387|ref|XP_451635.1|/1-361      ANNRGSWTDL------------------
gi|46358064|ref|NP_035627.1|/1-417      KANSSKPPTGHTSKEPTSKSS-------
gi|45198420|ref|NP_985449.1|/1-367      EKNKPFW---------------------
gi|38327570|ref|NP_940838.1|/1-403      TANSSKPSNCQ-NKESASKQS-------
gi|17505246|ref|NP_491714.1|/1-305      QGMMEAKIRAEKQQKIEKEASLRNH---
gi|17137514|ref|NP_477336.1|/1-329      KAGMAERELQLQKRERGKENTARN----
gi|17136516|ref|NP_476749.1|/1-411      LAHTQ-----------------------
gi|16758580|ref|NP_446201.1|/1-343      RANSRRVLPPSAL---------------
gi|15233958|ref|NP_195009.1|/1-294      VQNADPSGIYRV----------------
gi|15225495|ref|NP_182073.1|/1-288      VKNADPKGVCASIDI-------------
gi|6708213|gb|AAF25838.1|/1-282         REHSRRVLPPCVHGAS------------
gi|6325047|ref|NP_015115.1|/1-367       LRNKPFWENKRL----------------
gi|2979623|gb|AAC12683.1|/1-345         RANSRRVLPPSAL---------------
```
