# Supplementary material for: Differential Selective Pressures Experienced by the Aurora Kinase Gene Family
Source: Int J Mol Sci. 2017 Dec 28;19(1):72. doi: 10.3390/ijms19010072 (PMC5796022; doi:10.3390/ijms19010072)
Supplement: Supplementary file 1 [file ijms-19-00072-s001.zip › FigureS2AKABCalignwLegend.pdf]

Figure S2. Alignment of placental mammal AK sequences. The alignment of the placental mammal AKA, AKB, and AKC sequences used for the dN/dS analyses.

```
MATLAB multiple sequence alignment


lcl|NM_198436.2 MDRSKENCISGPVKATAPVG-GPKRVLVTQQFPCQNPLPVNSGQAQRVLCPSNSSQRIPL  59
lcl|XM_01514873 MDRSKENCISGPVKPAAPVG-GPKRVLVTQQFPCQNPLPANSGQAQRVLCPSNSSQRVPL  59
lcl|NM_00103802 MDRCKENCISGP-KTAVPLSDGPKRVPVAQQFPSQNPVSVNSGQAQRVLCPTNSSQRVPS  59
lcl|NM_00114250 MDRCKENCISGP-KTAVPLSDGPKRVPVAQQFPSQNPVSVNSGQAQRVLCPTNSSQRVPS  59
lcl|XM_00563519 MDKSKENCIAGPVKTAIALGDGPKRVLVTQQVPSQNPLSANSGQAQRVLCPSNSSQRVPP  60
lcl|XM_00398335 MDKSKENCVAGPVKTTIPLGDGPKRVPVTQQFPSQNLLSANSGQAQRVLCPSNSSQRIPS  60
lcl|XM_00567303 MDKCKENCISGL-KTTVPPGDGPKRVPVTQHFPAQHLPSANSGQAQRVLCPSNSSQRLPS  59
lcl|XM_00148927 MDRSKENCISGPVKTTVPLGDGPKRVPVTQQLP-QNLLSANSGQAQRVLCPSNSSHRVPS  59
lcl|XM_00443024 MDRSKENCVAGPVKTTIPLGDGPKRVLVTQQFPSQHPLSANSGQAQRVLCPSNSSHRAPT  60
lcl|XM_00827414 MERSKENCISGPIKAAALLGDGPKRVPVTQRFPSQNPVSACSGQAERVLCPSNSSQRVPS  60
lcl|XM_01452869 MDKSKENGISGLVKTTIPSGDGPKRVLVTQQFPSQYPLPANSGQAQRVLCPSNSSQQVPS  60
lcl|XM_00468690 MDKSKENCISGPVKTMVPLRDGAKRVPVHQHAPSQSALPASSGQAQRVLCPSN-SQRLPS  59
lcl|XM_00341990 MDKSKENCISGPIKTTLPLGDGPKRVPVTQPFPSQNPLSASGGQAQRVLCPSNSSQSIPS  60
lcl|XM_00432103 MDKCKENSISGP-KTTVPLGDGPKRVPVTQQFPSQNPVSANSGQAQRVLCPSNSSQRVPS  59
lcl|NM_004217.3 MAQKENSYPWPYGRQTAPSG--------------------------LSTLPQRVLRKEPV  34
lcl|XM_01511855 MAQKENAYPWPYGRQTAPSG--------------------------LSTLPQRVLRKEPV  34
lcl|NM_183084.2 MAQKENAYPWPYGRQTAQPG--------------------------LNTLPQRVLRKEPV  34
lcl|XM_00401268 MAQKENAYPWPYGRQTAQPG--------------------------LNTLPQRVLRKEAV  34
lcl|XM_844813.4 MTQKENAYPWPYGRQTTQPG--------------------------LNTLPQRVLRKDPA  34
lcl|XM_00399619 MAQKENAYPWPYGRQTTQPG--------------------------LNTLPQRVLRKEPA  34
lcl|NM_213919.2 MAQKENTYPWPYGRQTAQSG--------------------------LNILPQRVLRKEAV  34
lcl|XM_00150481 MTQKENAYPWPYGRQTAQSG--------------------------LNTLPQRVLRKEPA  34
lcl|XM_00443309 MAQKENAYPWPYGRQTAQSG--------------------------LNTLPQRVLRKEPV  34
lcl|XM_00827074 MAQKENAYPWPYSRPTPQSG--------------------------LNTLPQRILRKEAA  34
lcl|XM_00587878 MTQKENAYPWPYGRQTAQCG--------------------------LNTLPQRVLQKDPT  34
lcl|XM_00468461 MAQKENAYPWPYGRQTAQSG--------------------------LNTLPQRVLRKDPT  34
lcl|XM_00341691 MAQKENAYPWPYGRQTAQSG--------------------------LNTLPQRVFRKDLA  34
lcl|XM_01994424 MAQKENAYPWPYGRQTAQSG--------------------------LNTLPQRVLRKEPV  34
lcl|NM_00101587 MSSPRAVVQLGKAQ----------------------------------------------  14
lcl|XM_00109836 MSSPKAAVQLGRAQ----------------------------------------------  14
lcl|XM_00521979 MSHRGTARKAGGMQ----------------------------------------------  14
lcl|XM_00401544 MSHRGTARKAGGMQ----------------------------------------------  14
lcl|XM_00561607 MLSGSAQPASEGE-----------------------------------------------  13
lcl|XM_00694080 MQSGSVQPAAEEEE----------------------------------------------  14
lcl|XM_02109730 MKSGSVQPAVAAA-----------------------------------------------  13
lcl|XM_00150199 MSLPEAVMKSGSV-----------------------------------------------  13
lcl|XM_00443911 MSLPRAVMKLGDV-----------------------------------------------  13
lcl|XM_00824896 MQS---------------------------------------------------------   3
lcl|XM_01453723 M--------------------------------------------------------NPG   4
lcl|XM_01273470 MKS---------------------------------------------------------   3
lcl|XM_00340664 MFTNVALSFARREW----------------------------------------------  14
lcl|XM_01992887 MKSGSAQPAAVAG-----------------------------------------------  13
                *

lcl|NM_198436.2 QAQKLVSSHK--PVQNQKQKQLQATSVPHPVSRPLNNTQKSKQPLPSAPENNPEEELASK 117
lcl|XM_01514873 QAQKLVSSHK--LVQNQKQKQLQATSVPHPVSRPPSHTPKSKQPLPSAPENNPEEELASK 117
lcl|NM_00103802 QAQKLVSIQK--PVQTLKQKPPQAASAPRPVTRPPSNTQKSKQPQPPAPGNNPEKEVASK 117
lcl|NM_00114250 QAQKLVSIQK--PVQTLKQKPPQAASAPRPVARPPSNTQKSKQPQPPAPGNNPEKEVASK 117
lcl|XM_00563519 QTQKLVSSHK--PAQNLKQKQLQATGVPRPASRSLNNTQKSEQPSSSAPGNNSEKELATK 118
lcl|XM_00398335 QTQKLVSSHK--PVQNLKQKQLQATSVPRSVSRPLNNTQKSEQPLPSATGNNSEKELTTK 118
lcl|XM_00567303 HTQKLVSSHK--PVQNLKQKQSQATSGPRPVSRPLSNTQQSEQPQPAAPGNNPEKEAASK 117
lcl|XM_00148927 QAQKLVSSHK--PVQNLKQKQSQATSVPHPVPRPLNNTQRSEQLQPSAPGNNSEKELASK 117
lcl|XM_00443024 QAQKLVSSHR--PVQNLKQKQSQATSVPPPVSRPLNSTQKSEQPQPSAPGNNSEKERTSK 118
lcl|XM_00827414 QAQKLLPAEK----PVQKLKQAQAASVPRPVSKPPNNTPKSEQPPPSAPGNNPEKELTSN 116
lcl|XM_01452869 KAQKLVSSHK--PVLNLKQKQLQATSGPCPVSRPLNNTQKSEQPQPPAPGKNSEKEVASK 118
lcl|XM_00468690 QAQKLVSSHKAVPNPNLKQKQLQANNAPRAAPRPLAGTPKSEPP-PSAPGNNSEKEQASK 118
lcl|XM_00341990 QAQKLVSSRK--PVQSQKQKPLQITTVPRPVSRPPNNTPKGEQAQPPASGNNSGKELASK 118
lcl|XM_00432103 QVQKLVSSQK--SVQPLKQKPTQAASAPRSVSRPLSNTQKSEQPQLPAPGNNPEKEAASK 117
lcl|NM_004217.3 TPSALVLMSR------------------------------SNVQPTAAPGQ---KVMENS  61
lcl|XM_01511855 TPSALVLMSR------------------------------SNVQPTAAPGQ---KVVENS  61
lcl|NM_183084.2 TPSALVLMSR------------------------------SNAQPTAAPGQ---KVVENS  61
lcl|XM_00401268 IPSALVLMSR------------------------------SNAQPTAAPGQ---KVVENS  61
lcl|XM_844813.4 TPSALVLMSR------------------------------SNGQPTAAPGQ---KVVENS  61
lcl|XM_00399619 TPSALVLMSR------------------------------SNTQPTAAPGQ---KVVENS  61
lcl|NM_213919.2 TPSALVLMSR------------------------------SNTQPTAAPGQ---KVVENS  61
lcl|XM_00150481 TPSALVLMSR------------------------------SNAQPTVALGQ---KVMENS  61
lcl|XM_00443309 TPSALVLMSR------------------------------SNAQPTAALGQ---KVVENS  61
lcl|XM_00827074 TPSALVLLNR------------------------------SNAPPTAVLGQ---KGMENS  61
lcl|XM_00587878 TPSSLVLMSR------------------------------SNAQPTAAPGQ-------NS  57
lcl|XM_00468461 TPSALVLMNR------------------------------SNTQPTAAPGQ---KVVENS  61
lcl|XM_00341691 TPSALVLMSR------------------------------SNAQPTAAPGE--KKMTENS  62
lcl|XM_01994424 TPSALVLMSR------------------------------SNAQPTAAPLQ---KVVENN  61
lcl|NM_00101587 --------PA------------------------------GEELATANQTA---------  27
lcl|XM_00109836 --------PA------------------------------GEAVTAANQTA---------  27
lcl|XM_00521979 --------------------------------------------HAAAAGQ---------  21
lcl|XM_00401544 --------------------------------------------PAAAAGQ---------  21
lcl|XM_00561607 ---------K------------------------------TQEQSKDAAGH---------  25
lcl|XM_00694080 ---------Q------------------------------TRDQGTAAAGL---------  26
lcl|XM_02109730 -------------------------------------------------GQ---------  15
lcl|XM_00150199 ------------------------------------------RPAVPTAGQ---------  22
lcl|XM_00443911 ------------------------------------------QSAGETAGQ---------  22
lcl|XM_00824896 --------------------------------------------GSSQAAG---------  10
```

```
lcl|XM_01453723 FLLSIIAEKK------------------------------EPWPSSPSPGS---------  25
lcl|XM_01273470 ----------------------------------------GDAQAAGAAGG---------  14
lcl|XM_00340664 -----------------------------------------KELAPTERAL---------  24
lcl|XM_01992887 --------------------------------------------------Q---------  14


lcl|NM_198436.2 QKNEESKKRQWALEDFEIGRPLGKGKFGNVYLAREKQSKFILALKVLFKAQLEKAGVEHQ 177
lcl|XM_01514873 QKNEESKKRQWALEDFEIGRPLGKGKFGNVYLAREKQSKFILALKVLFKAQLEKAGVEHQ 177
lcl|NM_00103802 QKNEESKKRQWALEDFEIGRPLGKGKFGNVYLAREKQSKFILALKVLFKAQLEKAGVEHQ 177
lcl|NM_00114250 QKNEESKKRQWALEDFEIGRPLGKGKFGNVYLAREKQSKFILALKVLFKAQLEKAGVEHQ 177
lcl|XM_00563519 QKNEESKKRQWALEDFEIGRPLGKGKFGNVYLAREKQSKFILAIKVLFKAQLEKAGVEHQ 178
lcl|XM_00398335 QKNEESKKRQWALEDFEIGRPLGKGKFGNVYLAREKQSKFILALKVLFKAQLEKAGVEHQ 178
lcl|XM_00567303 QKNEESKKRQWALEDFEIGRPLGKGKFGNVYLAREKQSKFILALKVLFKTQLEKAGVEHQ 177
lcl|XM_00148927 EKNEESKKRQWALEDFEIGRPLGKGKFGNVYLAREKQSKFILALKVLFKAQLEKAGVEHQ 177
lcl|XM_00443024 EKNEDSKRRQWALEDFEIGRPLGKGKFGNVYLAREKQSKFILALKVLFKAQLEKAGVEHQ 178
lcl|XM_00827414 QKNEESKKRQWTLEDFDIGRPLGKGKFGNVYLAREKQSKFILALKVLFKAQLEKAGVEHQ 176
lcl|XM_01452869 QKNEESKK-QWTLEDFEIGRPLGKGKFGNVYLAREKQSKFILALKVLFKAQLEKAGVEHQ 177
lcl|XM_00468690 QKTEESKKRQWALEDFEIGRPLGKGKFGNVYLAREKQSKFILALKVLFKAQLEKAGVEHQ 178
lcl|XM_00341990 QKKEESKRRQWALEDFEIGRPLGKGKFGNVYLAREKQSKFILALKVLFKAQLEKAGVEHQ 178
lcl|XM_00432103 QKNEESKRRQWALEDFEIGRPLGKGKFGNVYLAREKQSKFILALKVLFKAQLEKAGVEHQ 177
lcl|NM_004217.3 SGTPDILTRHFTIDDFEIGRPLGKGKFGNVYLAREKKSHFIVALKVLFKSQIEKEGVEHQ 121
lcl|XM_01511855 SGTPNILMRHFTIDDFEIGRPLGKGKFGNVYLAREKKSHFIVALKVLFKSQIEKEGVEHQ 121
lcl|NM_183084.2 SGTPNIPKRSFTIDDFEIGRPLGKGKFGNVYLAREKKSHFIVALKVLFKSQIEKEGVEHQ 121
lcl|XM_00401268 SGTLNIPKRSFTIDDFEIGRPLGKGKFGNVYLAREKKSHFIVALKVLFKSQIEKEGVEHQ 121
lcl|XM_844813.4 SGIPNFSMRSFTIDDFEIGRPLGKGKFGNVYLAREKKSHFIVALKVLFKSQIEKEGVEHQ 121
lcl|XM_00399619 SGTPNFSMRSFTIDDFEIGRPLGKGKFGNVYLAREKKSHFIVALKVLFKSQIEKEGVEHQ 121
lcl|NM_213919.2 SGTPNFSTRSFTIDDFEIGRPLGKGKFGNVYLAREKKSHFIVALKVLFKSQIEKEGVEHQ 121
lcl|XM_00150481 SGTPNLLMRSFTIDDFEIGRPLGKGKFGNVYLAREKKSHFIVALKVLFKSQIEKEGVEHQ 121
lcl|XM_00443309 SGTPNLLMRSFTIDDFEIGRPLGKGKFGNVYLAREKKSHFIVALKVLFKSQIEKEGVEHQ 121
lcl|XM_00827074 SGAPNF-TRPFTIDDFEIGRPLGKGKFGNVYLAREKKSHFIVALKVLFKSQIEKEGVEHQ 120
lcl|XM_00587878 NGTPN-LMRSFTIDDFEIGRPLGKGKFGNVYLAREKKSRFIVALKVLFKSQIEKEGVEHQ 116
lcl|XM_00468461 SGTPNLSMRSFTIDDFEIGRPLGKGKFGNVYLAREKKSHFIVALKVLFKSQIEKEGVEHQ 121
lcl|XM_00341691 SGTPSFLMRPFTIDDFEIGRPLGKGKFGNVYLAREKKSHFIVALKVLFKSQIEKEGVEHQ 122
lcl|XM_01994424 SRTPNFSMRSFTIDDFEIGRPLGKGKFGNVYLAREKKSHFIVALKVLFKSQIEKEGVEHQ 121
lcl|NM_00101587 QQPSSPAMRRLTVDDFEIGRPLGKGKFGNVYLARLKESHFIVALKVLFKSQIEKEGLEHQ  87
lcl|XM_00109836 QQPSSPAMRRLKIDDFEIGRPLGKGKFGNVYLARLKESHFIVALKVLFKSQIEKEGLEHQ  87
lcl|XM_00521979 TVPGAPTARRFTVDDFEIGRPLGKGKFGNVYLARLKKNHFIVALKVLFKSQIEKEGLEHQ  81
lcl|XM_00401544 SVPGAPAARRFTIDDFEIGRPLGKGKFGNVYLARLKKNHFIVALKVLFKSQIEKEGLEHQ  81
lcl|XM_00561607 VELSVPPGRRLTIDDFEIGRPLGKGKFGNVYLARLKESHFIVALKVLFKSQIEKEGLEHQ  85
lcl|XM_00694080 VELGMPPVRRLTIEDFEIGRPLGKGKFGNVYLARLKESHFLVALKVIFKSQIEKEGLEHQ  86
lcl|XM_02109730 TVSNIPTGRRLTIDDFEIGRPLGKGKFGNVYLARLKESHFIVALKVLFKSQIEKEGLEHQ  75
lcl|XM_00150199 SQPGFPNVRHLTIDDFEIGRPLGKGKFGNVYLARLKESHFIVALKVLFKSQIEKEGLEHQ  82
lcl|XM_00443911 TQPSFPTVRHFTIDDFEIGRPLGKGKFGNVYLARLKESHFIVALKVLFKSQIEKEGLEHQ  82
lcl|XM_00824896 PAQPSVPLRRLTIEDFEIGRPLGKGKFGNVYLARLKDSHFIVALKVLFKSQIEKEGMEHQ  70
lcl|XM_01453723 --------RSLTIDDFDIRCPLGKGKFGNVYLARLRDSHFIVALKVLFKSQLEKEGMEHQ  77
lcl|XM_01273470 PALPVATVRRLTIDDFEIGRPLGKGKFGNVYLARLRESRFLVALKVLFKSQIEKEGLEHQ  74
lcl|XM_00340664 YGDVLLEMRRLRIDDFEIGRPLGKGKFGNVYLARLQENHFLVALKVLFKSQIEKEGLEHQ  84
lcl|XM_01992887 TMPSIPTVRRLTIDDFEIGRPLGKGKFGNVYLARLKESHFIVALKVLFKSQIEKEGLEHQ  74
                            ::**:*  ************** :..:*::*:**:**:*:** *:***

lcl|NM_198436.2 LRREVEIQSHLRHPNILRLYGYFHDATRVYLILEYAPLGTVYRELQKLSKFDEQRTAT-Y 236
lcl|XM_01514873 LRREVEIQSHLRHPNILRLYGYFHDATRVYLILEYAPLGTVYRELQKLSKFDEQRTAT-Y 236
lcl|NM_00103802 LRREVEIQSHLRHPNILRLYGYFHDATRVYLILEYAPLGAVYRELQKLSKFDEQRTAT-Y 236
lcl|NM_00114250 LRREIKIQSHLKHPNILRLYGYFHDATRVYLILEYAPLGAVYRELQKLSKFDEQRTAT-Y 236
lcl|XM_00563519 LRREVEIQSHLRHPNILRLYGYFHDATRVYLILEYAPLGAVYRELQKLSKFDEQRTAT-Y 237
lcl|XM_00398335 LRREVEIQSHLRHPNILRLYGYFHDATRVYLILEYAPLGAVYRELQKLSKFDEQRTAT-Y 237
lcl|XM_00567303 LRREVEIQSHLRHPNILRLYGYFHDATRVYLILEYAPLGAVYRELQKLSKFDEQRTAT-Y 236
lcl|XM_00148927 LRREVEIQSHLRHPNILRLYGYFHDATRVYLILEYAPLGAVYRELQKLSKFDEQRTAT-Y 236
lcl|XM_00443024 LRREVEIQSHLRHPNILRLYGYFHDATRVYLILEYAPLGAVYRELQKLSKFDEQRTAT-Y 237
lcl|XM_00827414 LRREVEIQSHLRHPNILRLYGYFHDATRVYLILEYAPLGTVYRELQKLSKFDEQRTAT-Y 235
lcl|XM_01452869 IRREVEIQSHLRHPNILRLYGYFHDATRVYLILEYAPLGTVYRELQKLSRFDEQRTAT-Y 236
lcl|XM_00468690 LRREVEIQSHLRHPNILRLYGYFHDATRVYLILEYAPLGAVYRELQKLSKFDEQRTAT-Y 237
lcl|XM_00341990 VRREVEIQSHLRHPNILRLYGYFHDATRVYLILEYAPLGTVYRELQKXSKFDEQRTAP*Y 238
lcl|XM_00432103 LRREVEIQSHLRHPNILRLYGYFHDATRVYLILEYAPLGAVYRELQKLSKFDEQRTAT-Y 236
lcl|NM_004217.3 LRREIEIQAHLHHPNILRLYNYFYDRRRIYLILEYAPRGELYKELQKSCTFDEQRTAT-I 180
lcl|XM_01511855 LRREIEIQAHLHHPNILRLYNYFYDRRRIYLILEYAPRGELYKELQKSRTFDEQRTAT-I 180
lcl|NM_183084.2 LRREIEIQAHLQHPNILRLYNYFYDRRRIYLILEYAPRGELYKELQKSRTFDEQRTAT-I 180
lcl|XM_00401268 LRREIEIQAHLQHPNILRLYNYFYDRRRIYLILEYAPRGELYKELQKSRTFDEQRTAT-I 180
lcl|XM_844813.4 LRREIEIQAHLQHPNILRLYNYFYDRRRIYLILEYAPRGELYKELQKSHTFDEQRTAT-I 180
lcl|XM_00399619 LRREIEIQAHLQHPNILRLYNYFYDRRRIYLILEYAPRGELYKELQKSRTFDEQRTAT-I 180
lcl|NM_213919.2 LRREIEIQAHLQHPNILRLYNYFYDRRRIYLILEYAPRGELYKELQKCRTFDEQRTAT-I 180
lcl|XM_00150481 LRREIEIQAHLQHPNILRLYNYFYDRRRIYLILEYAPRGELYKELQKSRTFDEQRTAT-I 180
lcl|XM_00443309 LRREIEIQAHLQHPNILRLYNYFYDRRRIYLILEYAPRGELYKELQKSRTFDEQRTAT-I 180
lcl|XM_00827074 LRREIEIQAHLQHPNILRLYNYFYDRRRIYLILEYAPRGELYKELQKSRTFDEQRTAT-I 179
lcl|XM_00587878 LRREIEIQAHLQHPNILRLYNYFYDRRRIYLILEYAPRGELYKELQKSRTFDQQRTAT-I 175
lcl|XM_00468461 LRREIEIQAHLQHPNILRLYNYFYDRRRIYLILEYAPRGELYKELQKSRTFDEQRTAT-I 180
lcl|XM_00341691 LRREIEIQAHLQHPNILRLYNYFYDRRRVYLILEYAPRGELYKELQKSRTFDEQRTAT-I 181
lcl|XM_01994424 LRREIEIQAHLQHPNILRLYNYFYDRRRIYLILEYAPRGELYKELQKSHTFDEQRTAT-I 180
lcl|NM_00101587 LRREIEIQAHLQHPNILRLYNYFHDARRVYLILEYAPRGELYKELQKSEKLDEQRTAT-I 146
lcl|XM_00109836 LRREIEIQAHLQHPNILRLYNYFHDARRVYLILEFAPRGELYKELQKSEKLDEQRAAT-I 146
lcl|XM_00521979 LRREVEIQAHLQHPNILRLYNYFHDARRVYLILEYAPKGELYKALQRSHTFDEQRTAT-I 140
lcl|XM_00401544 LRREVEIQAHLQHPNILRLYNYFHDARRVYLILEYAPKGELYKALQRSHTFDEQRTAT-I 140
lcl|XM_00561607 LRREIEIQAHLQHPNILRLYNYFHDARRVYLILEYAPRGELYKELQKSHTLDEQHTAT-I 144
lcl|XM_00694080 LRREIEIQAHLQHPNILRLYNYFHDARRVYLILEYAPRGELYKELQKSNTLDEQHTAT-I 145
lcl|XM_02109730 LRREIEIQAHLQHPNILRLYNYFHDARRVYLILEYAPRGELYKELQKSHTLDEQRTAT-I 134
```

```
lcl|XM_00150199 LRREIEIQAHLQHPNILRLYNYFHDARRVYLILEYAPRGELYKELQKSHTLDEQRTAT-I 141
lcl|XM_00443911 LRREIEIQAHLQHPNILRLYNYFHDARRVYLILEYAPRGELYKELQKSHTLDEQRTAT-I 141
lcl|XM_00824896 LRREIEIQAHLQHPNILRLYNYFHDARRIYLILEYAPRGELYKELQKCHTLDEQRTAT-I 129
lcl|XM_01453723 LRREIEIQSHLQHPNILRLHNYFHDERXVYLILEYAPRGELYKELQKSHTIDEQSTAT-I 136
lcl|XM_01273470 LRREVEIQAHLRHPNILRLYNYFHDARRVYLILEYAPRGELYKELQNCHTLDEQRTAT-I 133
lcl|XM_00340664 LRREIEIQAHLQHPNILRLYNYFHDSRRVYLILEYAPRGELYKELQKSHRLDEQRTAT-I 143
lcl|XM_01992887 LRREIEIQAHLQHPNILRLYNYFHDARRMYLILEYAPRGELYKELQKSHTLDEQRTAT-V 133
                :***::**:**:*******:.**:*   :*****:** * :*: **.   :*:* :*.

lcl|NM_198436.2 ITELANALSYCHSKRVIHRDIKPENLLLGSAGELKIADFGWSVHAPSSRRTTLCGTLDYL 296
lcl|XM_01514873 ITELANALSYCHSKRVIHRDIKPENLLLGSAGELKIADFGWSVHAPSSRRTTLCGTLDYL 296
lcl|NM_00103802 ITELANALSYCHSKRVIHRDIKPENLLLGSAGELKIADFGWSVHAPSSRRTTLCGTLDYL 296
lcl|NM_00114250 ITELANALSYCHSKRVIHRDIKPENLLLGSAGELKIADFGWSVHAPSSRRTTLCGTLDYL 296
lcl|XM_00563519 ITELADALSYCHSKRVIHRDIKPENLLLGSAGELKIADFGWSVHAPSSRRTTLCGTLDYL 297
lcl|XM_00398335 ITELADALSYCHSKRVIHRDIKPENLLLGSAGELKIADFGWSVHAPSSRRTTLCGTLDYL 297
lcl|XM_00567303 ITELANALSYCHSKRVIHRDIKPENLLLGSAGELKIADFGWSVHAPSSRRTTLCGTLDYL 296
lcl|XM_00148927 ITELANALAYCHSKRVIHRDIKPENLLLGSAGELKIADFGWSVHAPSSRRTTLCGTLDYL 296
lcl|XM_00443024 ITELANALAYCHSKRVIHRDIKPENLLLGSAGELKIADFGWSVHAPSSRRTTLCGTLDYL 297
lcl|XM_00827414 ITELANALLYCHSKRVIHRDIKPENLLLGSAGELKIADFGWSVHAPSSRRTTLCGTLDYL 295
lcl|XM_01452869 ITELTNALSYCHSKRVIHRDIKPENLLLGSTGELKIADFGWSVHAPSSRRTTLCGTLDYL 296
lcl|XM_00468690 ITELADALSYCHSKRVIHRDIKPENLLLGSAGELKIADFGWSVHAPSSRRTTLCGTLDYL 297
lcl|XM_00341990 ITELADALSYCHSKKVIHRDIKPENLLLGSTGELKIADFGWSVHAPSSRRTTLCGTLDYL 298
lcl|XM_00432103 ITELANALSYCHSKRVIHRDIKPENLLLGSSGELKIADFGWSVHAPSSRRTTLCGTLDYL 296
lcl|NM_004217.3 MEELADALMYCHGKKVIHRDIKPENLLLGLKGELKIADFGWSVHAPSLRRKTMCGTLDYL 240
lcl|XM_01511855 MEELADALMYCHGKKVIHRDIKPENLLLGLKGELKIADFGWSVHAPSLRRKTMCGTLDYL 240
lcl|NM_183084.2 MEELADALTYCHAKKVIHRDIKPENLLLGLRGELKIADFGWSVHAPSLRRKTMCGTLDYL 240
lcl|XM_00401268 MEELADALTYCHAKKVIHRDIKPENLLLGLRGELKIADFGWSVHAPSLRRKTMCGTLDYL 240
lcl|XM_844813.4 MEELADALMYCHGKKVIHRDIKPENLLLGLQGELKIADFGWSVHAPSLRRKTMCGTLDYL 240
lcl|XM_00399619 MEELADALLYCHGKKVIHRDIKPENLLLGLQGELKIADFGWSVHAPSLRRKTMCGTLDYL 240
lcl|NM_213919.2 MEELADALIYCHGKKVIHRDIKPENLLLGLQGELKIADFGWSVHAPSLRRKTMCGTLDYL 240
lcl|XM_00150481 MEELADALMYCHGKKVIHRDIKPENLLLGLQGELKIADFGWSVHAPSLRRKTMCGTLDYL 240
lcl|XM_00443309 MEELADALIYCHGKKVIHRDIKPENLLLGLQGELKIADFGWSVHAPSLRRKTMCGTLDYL 240
lcl|XM_00827074 MEELADALRYCHEKKVIHRDIKPENLLLGLQGELKIADFGWSVHAPSLRRKTMCGTLDYL 239
lcl|XM_00587878 MEELADALMYCHKKKVIHRDIKPENLLLGLQGELKIADFGWSVHAPSLRRKTMCGTLDYL 235
lcl|XM_00468461 MEELADALIYCHGKKVIHRDIKPENLLLGLQGELKIADFGWSVHAPSLRRKTMCGTLDYL 240
lcl|XM_00341691 MEELADALMYCHGKKVIHRDIKPENLLLGLRGELKIADFGWSVHAPSLRRKTMCGTLDYL 241
lcl|XM_01994424 MEELADALIYCHGKKVIHRDIKPENLLLGLQGELKIADFGWSVHAPSLRRKTMCGTLDYL 240
lcl|NM_00101587 IEELADALTYCHDKKVIHRDIKPENLLLGFRGEVKIADFGWSVHTPSLRRKTMCGTLDYL 206
lcl|XM_00109836 IEELADALTYCHEKKVIHRDIKPENLLLGFRGEVKIADFGWSVHTPSLRRKTMCGTLDYL 206
lcl|XM_00521979 IEELADALIYCHERKVIHRDIKPENLLLGLMGEVKIADFGWSVHTPSLRRRTTCGTLDYL 200
lcl|XM_00401544 IEELADALIYCHEKKVIHRDIKPENLLLGLMGEVKIADFGWSVHTPSLRRRTTCGTLDYL 200
lcl|XM_00561607 MEELADALTYCHEKKVIHRDIKPENLLLGFKGEVKIADFGWSVHTPSLRRKTMCGTLDYL 204
lcl|XM_00694080 MEELADALTYCHEKKVIHRDIKPENLLLGFRGEVKIADFGWSVHTPSLRRKTMCGTLDYL 205
lcl|XM_02109730 MEELADALTYCHEKKVIHRDIKPENLLLGFRGEVKIADFGWSVHTPSLRRKTMCGTLDYL 194
lcl|XM_00150199 MEELADALTYCHEKKVIHRDIKPENLLLGFRGEVKIADFGWSVHTPSLRRKTMCGTLDYL 201
lcl|XM_00443911 MEELADALTYCHEKKVIHRDIKPENLLLGFRGEVKIADFGWSVHTPSLRRKTMCGTLDYL 201
lcl|XM_00824896 MEELADALTYCHGKKVIHRDIKPENLLLGFRGEVKIADFGWSVHTLSLRRKTMCGTLDYL 189
lcl|XM_01453723 MEELAHALAYCHENKVIHRDIKPENLLLGLRGEVKIADFGWSVHTPSLRRKTMCGTLDYL 196
lcl|XM_01273470 IEELADALTYCHEKKVIHRDIKPENLLLGYRGEVKIADFGWSVHTPSLRRKTMCGTLDYL 193
lcl|XM_00340664 MEELADALIYCHEKKVIHRDIKPENLLLGLRGEVKIADFGWSVHTLSLRRKTMCGTLDYL 203
lcl|XM_01992887 MEELADALTYCHEKKVIHRDIKPENLLLGFRGEVKIADFGWSVHTPSLRRRTMCGTLDYL 193
                : **:.** *** .:**************  **:**********: * ** * *******

lcl|NM_198436.2 PPEMIEGRMHDEKVDLWSLGVLCYEFLVGKPPFEANTYQETYKRISRVEFTFPDFVTEGA 356
lcl|XM_01514873 PPEMIEGRMHDEKVDLWSLGVLCYEFLVGKPPFEANTYQETYKRISRVEFTFPDFVTEGA 356
lcl|NM_00103802 PPEMIEGRMHDEKVDLWSLGVLCYEFLVGKPPFEADTYQETYRRISRVEFTFPDCVPEGA 356
lcl|NM_00114250 PPEMIEGRMHDEKVDLWSLGVLCYEFLVGKPPFEADTYQETYRRISRVEFTFPDCVPEGA 356
lcl|XM_00563519 PPEMIEGRMHDEKVDLWSLGVLCYEFLVGKPPFEASTYQETYKRISRVEFTFPDFVPEGA 357
lcl|XM_00398335 PPEMIEGRMHDEKVDLWSLGVLCYEFLVGKPPFEASTYQETYKRISRVEFTFPDFVPEGA 357
lcl|XM_00567303 PPEMIEGRMHDEKVDLWSLGVLCYEFLVGKPPFEANTYQETYKRISRVEFTFPDFVPEGA 356
lcl|XM_00148927 PPEMIEGRMHDEKVDLWSLGVLCYEFLVGKPPFEASTYQETYKRISRVEFTFPDFVPEGA 356
lcl|XM_00443024 PPEMIEGRMHDEKVDLWSLGVLCYEFLVGKPPFEASTYQETYKRISRVEFTFPDFVPDGA 357
lcl|XM_00827414 PPEMIEGRMHDEKVDLWSLGVLCYEFLVGKPPFEANTYQETYRRISRVEFTFPDFVTEGA 355
lcl|XM_01452869 PPEMIEGRMHDEKVDLWSLGVLCYEFLVGKPPFEASTYQETYKRISRVEFTFPDFVREGA 356
lcl|XM_00468690 PPEMIEGRMHDEKVDLWSLGVLCYEFLVGKPPFEASTYQETYKRISRVEFTFPDFVPEGA 357
lcl|XM_00341990 PPEMIEGRMHDEKVDLWSLGVLCYEFLVGKPPFEASTYQETYNRISRVEFTFPDLVTVGA 358
lcl|XM_00432103 PPEMIEGRMHDEKVDLWSLGVLCYEFLVGKPPFEANTYQETYKRISRVEFTFPDFVPEGA 356
lcl|NM_004217.3 PPEMIEGRMHNEKVDLWCIGVLCYELLVGNPPFESASHNETYRRIVKVDLKFPASVPMGA 300
lcl|XM_01511855 PPEMIEGRTHNEKVDLWCIGVLCYELLVGNPPFESASHNETYRRIVKVDLKFPASVPTGA 300
lcl|NM_183084.2 PPEMIEGRTHNEKVDLWCIGVLCYELLVGNPPFESASHNETYRRIVKVDLKFPPSVPLGA 300
lcl|XM_00401268 PPEMIEGRTHNEKVDLWCIGVLCYELLVGNPPFESASHNETYRRIVKVDLKFPPSVPMGA 300
lcl|XM_844813.4 PPEMIEGRMHNEKVDLWCIGVLCYELLVGNPPFESASHNETYRRIVKVDLKFPPSVPTGA 300
lcl|XM_00399619 PPEMIEGRTHNEKVDLWCIGVLCYELLVGNPPFESASHNETYRRIVKVDLKFPPSVPTGA 300
lcl|NM_213919.2 PPEMIEGRTHNEKVDLWCIGVLCYELLVGNPPFESASHNETYRRIVKVDLKFPPSVPAGA 300
lcl|XM_00150481 PPEMIEGRTHNEKVDLWCIGVLCYELLVGNPPFESASHNETYRRIVKVDLKFPPSVPAGA 300
lcl|XM_00443309 PPEMIEGRTHNEKVDLWCIGVLCYELLVGNPPFESASHNETYRRIVKVDLKFPASVPAGA 300
lcl|XM_00827074 PPEMIEGRTHNEKVDLWCIGVLCYELLVGNPPFESASHNETYRRIVKVDLKFPASVPAGA 299
lcl|XM_00587878 PPEMIEGRTHNEKVDLWCIGVLCYELLVGNPPFESASHNETYRRIVKVDLKFPPSVPVGA 295
lcl|XM_00468461 PPEMIEGRTHNEKVDLWCIGVLCYELLVGSPPFESASHNETYRRIVKVDLKFPLSVPSGA 300
lcl|XM_00341691 PPEMIEGRMHNEKVDLWCIGVLCYELLVGNPPFESASHNETYRRIVKVDLKFPPSVPTGA 301
lcl|XM_01994424 PPEMIEGRTHNEKVDLWCIGVLCYELLVGNPPFESASHNETYRRIVKVDLKIPPSMPAGA 300
lcl|NM_00101587 PPEMIEGRTYDEKVDLWCIGVLCYELLVGYPPFESASHSETYRRILKVDVRFPLSMPLGA 266
lcl|XM_00109836 PPEMIEGRTYDEKVDLWCIGVLCYELLVGYPPFESTSHSETYRRILKVDVRFPLSMPLGA 266
lcl|XM_00521979 PPEMIEGRTYDEKVDLWCIGVLCYELLVGNPPFESASTSETYRRILKVDLRFPPSMSSGA 260
lcl|XM_00401544 PPEMIEGRTYDEKVDLWCIGVLCYELLVGNPPFESASTSETYRRILKVDLRFPPSMSSGA 260
```

```
lcl|XM_00561607 PPEMIERRTYNEKVDLWCIGVLCYELLVGYPPFESPSHNETYRRILKVDLRFPPSLPLGA 264
lcl|XM_00694080 PPEMIERRTYNETVDLWCIGVLCYELLVGNPPFESHSHNETYRRILKVDIRFPPSIPLGA 265
lcl|XM_02109730 PPEMIEGRTYNEKVDLWCIGVLCYELLVGNPPFESASHNETYRRILKVDVRFPPSLPLGA 254
lcl|XM_00150199 PPEMIEGRTYNEKVDLWCIGVLCYELLVGNPPFESNSHNETYRRILKVDVRFPPSIPSGA 261
lcl|XM_00443911 PPEMIEGRTYNEKVDLWCIGVLCYELLVGNPPFESNSHNETYRRILKVDVRFPPSIPLGA 261
lcl|XM_00824896 PPEMIEGRTYDEKVDLWCIGVLCYELLVGNPPFESVSHTETYRRILKVDVKFPVTMSLGA 249
lcl|XM_01453723 PPEMVEQRTYSEMVDLWCIGVLCYELLVGNPPFKSSSYSETYRRILKVDVKFPPSIPLGA 256
lcl|XM_01273470 PPEMIEGRTYNEKVDLWCIGVLCYELLVGKPPFESSTHNETYRRILKVDVRFPPSVPLGA 253
lcl|XM_00340664 PPEMIEGRTYDERVDLWCIGVLCYELLVGNPPFESPSHTETYRRILKVDVRFPSSMPSGA 263
lcl|XM_01992887 PPEMIEGRTYNEKVDLWCIGVLCYELLVGNPPFESPSHNETYRRILQVDVRFPPSMPLGA 253
                ****:* * :.* ****.:******:*** ***:: :  ***.** :*:. :*  :  **

lcl|NM_198436.2 RDLISRLLKHNPSQRPMLREVLEHPWITANSSKPSNCQNKESASKQS*- 404
lcl|XM_01514873 RDLISRLLKHNPSQRPMLREVLEHPWITANSSKPSNCQNRVN*------ 399
lcl|NM_00103802 RDLISRLLKHNPSQRPTLKEVLEHPWIIANSKPSSCQKKESTSKQS*-- 403
lcl|NM_00114250 RDLISRLLKHNPSQRPTLKEVLEHPWITANSKPSSCQKKESTSKQS*-- 403
lcl|XM_00563519 RDLISRLLKHNPSQRPTLKDVLEHPWIMANSSKPSSSQKNKDSTSKQS* 406
lcl|XM_00398335 RDLISRLLKHNPSQRPTLKEVLEHPWVTANSSKPSGSQKSKESTGKQS* 406
lcl|XM_00567303 RDLISRLLKHNPSHRPTLKEVLEHPWITANSKPASSHKKESTSKQP*-- 403
lcl|XM_00148927 RDLISRLLKHNPSQRPTLKEVLEHPWITANSSKPSNSQKSKESTSKQP* 405
lcl|XM_00443024 RDLISRLLKHNPSQRPTLKEVLEHPWITANASKPSNSQKSKESTSKQS* 406
lcl|XM_00827414 KDFISRLLKHNPSHRPTLAEVLEHPWVKAKSSKPSSCPDKEPASKQS*- 403
lcl|XM_01452869 RDLISRLLMHNPNQRLTLKEVLEHPWITANSSKPSSSQKSKESTSKQS* 405
lcl|XM_00468690 RDFISRLLKHNPSQRPTLKEVLEHPWITANSSKPPSSQKNKESTSKQS* 406
lcl|XM_00341990 RDLVSRLLKHNPSQRLTLKEVLEHPWITANSKKPPSSQNKEPTSKQS*- 406
lcl|XM_00432103 RDLISRLLKHNPSQRPTLKEVLEHPWITANSKPSSSQKKESASKQS*-- 403
lcl|NM_004217.3 QDLISKLLRHNPSERLPLAQVSAHPWVRANSRRVLPPSALQSVA*---- 345
lcl|XM_01511855 QDLISKLLRHNPSERLPLAQVSAHPWVRANSRRVLPPSALQSVA*---- 345
lcl|NM_183084.2 QDLIYKLLKHNPSERLPLAQVSAHPWVRTHSRRVLPPSAPQSVP*---- 345
lcl|XM_00401268 QDLISKLLKHNPSERLPLAQVSAHPWVRAHSRRVLPPSAPQSVP*---- 345
lcl|XM_844813.4 QDLISKLLKHNPSERLPLSQVSAHPWVRAHSRRMLPPSAVQAIP*---- 345
lcl|XM_00399619 QDLISKLLKHNPSERLPLAQVSAHPWVRAHSRRVLPPSALQSVP*---- 345
lcl|NM_213919.2 QDLISKLLKHNPSDRLPLAQVSAHPWVRAHSRRVLPPSAPQSVP*---- 345
lcl|XM_00150481 QDLISKLLRHNPSERLPLAQVSAHPWVRAHSRRVLPPSALQSVP*---- 345
lcl|XM_00443309 QDLISKLLKHNPSERLPLAQVSAHPWVRAHSRRVLPPSAPQSVP*---- 345
lcl|XM_00827074 QDLISKLLRHNPSDRLTLAQVSAHPWVRAHSRRVLPPSAMQSVA*---- 344
lcl|XM_00587878 QDLISKLLKHNPSDRLPLAQVSAHPWVRAHSRRVLPPSALQSVH*---- 340
lcl|XM_00468461 QDLITKLLKHNPSERLPLTQVSAHPWVRAHSRRVLPPSALQSVP*---- 345
lcl|XM_00341691 QDLISKLLKHNPSERLPLAQVSAHPWVRAHSRRVLPPSALQSVP*---- 346
lcl|XM_01994424 QDLISKLLKHNPSERLPLAQVAAHPWVRAHSRRVLPPSALQSVP*---- 345
lcl|NM_00101587 RDLISRLLRYQPLERLPLAQILKHPWVQAHSRRVLPPCAQMAS*----- 310
lcl|XM_00109836 RDLISKLLRYQPLERLPLAQILKHPWVQVHSRRVLPPCAQMAS*----- 310
lcl|XM_00521979 RDLISKLLRFQPLERLPLVRVLEHPWIRAHSQRVLPPSVPMAF*----- 304
lcl|XM_00401544 RDLISKLLRFQPLERLPLVRVLEHPWIRAHSQRVLPPSVQRAF*----- 304
lcl|XM_00561607 QDLVSKLLRYQPLERLPLAQIMEHPWVRAHSRRVLPPSVQMAS*----- 308
lcl|XM_00694080 RDLISKLLRYQPLERLPLSQILEHPWVRAHSRRVVPPSVQMDS*----- 309
lcl|XM_02109730 QDLISRLLRYQPSDRLPLDQILEHPWVRAHSRRVLPPSTQMVS*----- 298
lcl|XM_00150199 KDLISKLLRYQPLERLPLDQILEHPWVRAHSRRVLPPSAQMAS*----- 305
lcl|XM_00443911 KDLISKLLRYQPLERLPLDQIMEHPWVRAHSRRVLPPSAQMAS*----- 305
lcl|XM_00824896 RDLISRLLRYQPSERLPLAQILRHPWVQAHSRRVLPPCAHPAS*----- 293
lcl|XM_01453723 QDLISKLLRYQPSERLLLAQVLQHPWVQAHSRRVGPPPAPSAS*----- 300
lcl|XM_01273470 QDLISSLLRYQPSERLPLAQILQHPWVRMHSRRVLPPSAQVAS*----- 297
lcl|XM_00340664 QDLISKLLRYQPFERLALAQVLEHPWVQAHSQRVLPPSAQMAS*----- 307
lcl|XM_01992887 QDLISKLLRYQPSERLPLVQILEHPWVRAHSRRVLPPSAQMVS*----- 297
                :*::  ** .:* .*  *  :  ***:  ::
```
