# Supplementary material for: Differential Selective Pressures Experienced by the Aurora Kinase Gene Family
Source: Int J Mol Sci. 2017 Dec 28;19(1):72. doi: 10.3390/ijms19010072 (PMC5796022; doi:10.3390/ijms19010072)
Supplement: Supplementary file 1 [file ijms-19-00072-s001.zip › FigureS3AKAalignwLegend.pdf]

Figure S3. Alignment of placental mammal AKA sequences. The alignment of the placental mammal AKA sequences used for the dN/dS analyses.

```
MATLAB multiple sequence alignment


lcl|NM_198436.2 MDRSKENCISGPVKATAPVG-GPKRVLVTQQFPCQNPLPVNSGQAQRVLCPSNSSQRIPL  59
lcl|XM_01514873 MDRSKENCISGPVKPAAPVG-GPKRVLVTQQFPCQNPLPANSGQAQRVLCPSNSSQRVPL  59
lcl|NM_00103802 MDRCKENCISGP-KTAVPLSDGPKRVPVAQQFPSQNPVSVNSGQAQRVLCPTNSSQRVPS  59
lcl|NM_00114250 MDRCKENCISGP-KTAVPLSDGPKRVPVAQQFPSQNPVSVNSGQAQRVLCPTNSSQRVPS  59
lcl|XM_00563519 MDKSKENCIAGPVKTAIALGDGPKRVLVTQQVPSQNPLSANSGQAQRVLCPSNSSQRVPP  60
lcl|XM_00398335 MDKSKENCVAGPVKTTIPLGDGPKRVPVTQQFPSQNLLSANSGQAQRVLCPSNSSQRIPS  60
lcl|XM_00567303 MDKCKENCISGL-KTTVPPGDGPKRVPVTQHFPAQHLPSANSGQAQRVLCPSNSSQRLPS  59
lcl|XM_00148927 MDRSKENCISGPVKTTVPLGDGPKRVPVTQQLP-QNLLSANSGQAQRVLCPSNSSHRVPS  59
lcl|XM_00443024 MDRSKENCVAGPVKTTIPLGDGPKRVLVTQQFPSQHPLSANSGQAQRVLCPSNSSHRAPT  60
lcl|XM_00827414 MERSKENCISGPIKAAALLGDGPKRVPVTQRFPSQNPVSACSGQAERVLCPSNSSQRVPS  60
lcl|XM_01452869 MDKSKENGISGLVKTTIPSGDGPKRVLVTQQFPSQYPLPANSGQAQRVLCPSNSSQQVPS  60
lcl|XM_00468690 MDKSKENCISGPVKTMVPLRDGAKRVPVHQHAPSQSALPASSGQAQRVLCPSN-SQRLPS  59
lcl|XM_00341990 MDKSKENCISGPIKTTLPLGDGPKRVPVTQPFPSQNPLSASGGQAQRVLCPSNSSQSIPS  60
lcl|XM_00432103 MDKCKENSISGP-KTTVPLGDGPKRVPVTQQFPSQNPVSANSGQAQRVLCPSNSSQRVPS  59
                *::.*** ::*  *.      *.*** * *  * *   .. .***:*****:* *:  *

lcl|NM_198436.2 QAQKLVSSHK--PVQNQKQKQLQATSVPHPVSRPLNNTQKSKQPLPSAPENNPEEELASK 117
lcl|XM_01514873 QAQKLVSSHK--LVQNQKQKQLQATSVPHPVSRPPSHTPKSKQPLPSAPENNPEEELASK 117
lcl|NM_00103802 QAQKLVSIQK--PVQTLKQKPPQAASAPRPVTRPPSNTQKSKQPQPPAPGNNPEKEVASK 117
lcl|NM_00114250 QAQKLVSIQK--PVQTLKQKPPQAASAPRPVARPPSNTQKSKQPQPPAPGNNPEKEVASK 117
lcl|XM_00563519 QTQKLVSSHK--PAQNLKQKQLQATGVPRPASRSLNNTQKSEQPSSSAPGNNSEKELATK 118
lcl|XM_00398335 QTQKLVSSHK--PVQNLKQKQLQATSVPRSVSRPLNNTQKSEQPLPSATGNNSEKELTTK 118
lcl|XM_00567303 HTQKLVSSHK--PVQNLKQKQSQATSGPRPVSRPLSNTQQSEQPQPAAPGNNPEKEAASK 117
lcl|XM_00148927 QAQKLVSSHK--PVQNLKQKQSQATSVPHPVPRPLNNTQRSEQLQPSAPGNNSEKELASK 117
lcl|XM_00443024 QAQKLVSSHR--PVQNLKQKQSQATSVPPPVSRPLNSTQKSEQPQPSAPGNNSEKERTSK 118
lcl|XM_00827414 QAQKLLPAEK----PVQKLKQAQAASVPRPVSKPPNNTPKSEQPPPSAPGNNPEKELTSN 116
lcl|XM_01452869 KAQKLVSSHK--PVLNLKQKQLQATSGPCPVSRPLNNTQKSEQPQPPAPGKNSEKEVASK 118
lcl|XM_00468690 QAQKLVSSHKAVPNPNLKQKQLQANNAPRAAPRPLAGTPKSEPP-PSAPGNNSEKEQASK 118
lcl|XM_00341990 QAQKLVSSRK--PVQSQKQKPLQITTVPRPVSRPPNNTPKGEQAQPPASGNNSGKELASK 118
lcl|XM_00432103 QVQKLVSSQK--SVQPLKQKPTQAASAPRSVSRPLSNTQKSEQPQLPAPGNNPEKEAASK 117
                :.***:. .:       * *  *    * ...:.   * :.:    .*. :*. :* :::

lcl|NM_198436.2 QKNEESKKRQWALEDFEIGRPLGKGKFGNVYLAREKQSKFILALKVLFKAQLEKAGVEHQ 177
lcl|XM_01514873 QKNEESKKRQWALEDFEIGRPLGKGKFGNVYLAREKQSKFILALKVLFKAQLEKAGVEHQ 177
lcl|NM_00103802 QKNEESKKRQWALEDFEIGRPLGKGKFGNVYLAREKQSKFILALKVLFKAQLEKAGVEHQ 177
lcl|NM_00114250 QKNEESKKRQWALEDFEIGRPLGKGKFGNVYLAREKQSKFILALKVLFKAQLEKAGVEHQ 177
lcl|XM_00563519 QKNEESKKRQWALEDFEIGRPLGKGKFGNVYLAREKQSKFILAIKVLFKAQLEKAGVEHQ 178
lcl|XM_00398335 QKNEESKKRQWALEDFEIGRPLGKGKFGNVYLAREKQSKFILALKVLFKAQLEKAGVEHQ 178
lcl|XM_00567303 QKNEESKKRQWALEDFEIGRPLGKGKFGNVYLAREKQSKFILALKVLFKTQLEKAGVEHQ 177
lcl|XM_00148927 EKNEESKKRQWALEDFEIGRPLGKGKFGNVYLAREKQSKFILALKVLFKAQLEKAGVEHQ 177
lcl|XM_00443024 EKNEDSKRRQWALEDFEIGRPLGKGKFGNVYLAREKQSKFILALKVLFKAQLEKAGVEHQ 178
lcl|XM_00827414 QKNEESKKRQWTLEDFDIGRPLGKGKFGNVYLAREKQSKFILALKVLFKAQLEKAGVEHQ 176
lcl|XM_01452869 QKNEESKK-QWTLEDFEIGRPLGKGKFGNVYLAREKQSKFILALKVLFKAQLEKAGVEHQ 177
lcl|XM_00468690 QKTEESKKRQWALEDFEIGRPLGKGKFGNVYLAREKQSKFILALKVLFKAQLEKAGVEHQ 178
lcl|XM_00341990 QKKEESKRRQWALEDFEIGRPLGKGKFGNVYLAREKQSKFILALKVLFKAQLEKAGVEHQ 178
lcl|XM_00432103 QKNEESKRRQWALEDFEIGRPLGKGKFGNVYLAREKQSKFILALKVLFKAQLEKAGVEHQ 177
                :*.*:**: **:****:**************************:*****:**********

lcl|NM_198436.2 LRREVEIQSHLRHPNILRLYGYFHDATRVYLILEYAPLGTVYRELQKLSKFDEQRTAT-Y 236
lcl|XM_01514873 LRREVEIQSHLRHPNILRLYGYFHDATRVYLILEYAPLGTVYRELQKLSKFDEQRTAT-Y 236
lcl|NM_00103802 LRREVEIQSHLRHPNILRLYGYFHDATRVYLILEYAPLGAVYRELQKLSKFDEQRTAT-Y 236
lcl|NM_00114250 LRREIKIQSHLKHPNILRLYGYFHDATRVYLILEYAPLGAVYRELQKLSKFDEQRTAT-Y 236
lcl|XM_00563519 LRREVEIQSHLRHPNILRLYGYFHDATRVYLILEYAPLGAVYRELQKLSKFDEQRTAT-Y 237
lcl|XM_00398335 LRREVEIQSHLRHPNILRLYGYFHDATRVYLILEYAPLGAVYRELQKLSKFDEQRTAT-Y 237
lcl|XM_00567303 LRREVEIQSHLRHPNILRLYGYFHDATRVYLILEYAPLGAVYRELQKLSKFDEQRTAT-Y 236
lcl|XM_00148927 LRREVEIQSHLRHPNILRLYGYFHDATRVYLILEYAPLGAVYRELQKLSKFDEQRTAT-Y 236
lcl|XM_00443024 LRREVEIQSHLRHPNILRLYGYFHDATRVYLILEYAPLGAVYRELQKLSKFDEQRTAT-Y 237
lcl|XM_00827414 LRREVEIQSHLRHPNILRLYGYFHDATRVYLILEYAPLGTVYRELQKLSKFDEQRTAT-Y 235
lcl|XM_01452869 IRREVEIQSHLRHPNILRLYGYFHDATRVYLILEYAPLGTVYRELQKLSRFDEQRTAT-Y 236
lcl|XM_00468690 LRREVEIQSHLRHPNILRLYGYFHDATRVYLILEYAPLGAVYRELQKLSKFDEQRTAT-Y 237
lcl|XM_00341990 VRREVEIQSHLRHPNILRLYGYFHDATRVYLILEYAPLGTVYRELQKXSKFDEQRTAP*Y 238
lcl|XM_00432103 LRREVEIQSHLRHPNILRLYGYFHDATRVYLILEYAPLGAVYRELQKLSKFDEQRTAT-Y 236
                :***::*****:***************************:******* *:*******. *

lcl|NM_198436.2 ITELANALSYCHSKRVIHRDIKPENLLLGSAGELKIADFGWSVHAPSSRRTTLCGTLDYL 296
lcl|XM_01514873 ITELANALSYCHSKRVIHRDIKPENLLLGSAGELKIADFGWSVHAPSSRRTTLCGTLDYL 296
lcl|NM_00103802 ITELANALSYCHSKRVIHRDIKPENLLLGSAGELKIADFGWSVHAPSSRRTTLCGTLDYL 296
lcl|NM_00114250 ITELANALSYCHSKRVIHRDIKPENLLLGSAGELKIADFGWSVHAPSSRRTTLCGTLDYL 296
lcl|XM_00563519 ITELADALSYCHSKRVIHRDIKPENLLLGSAGELKIADFGWSVHAPSSRRTTLCGTLDYL 297
```

```
lcl|XM_00398335 ITELADALSYCHSKRVIHRDIKPENLLLGSAGELKIADFGWSVHAPSSRRTTLCGTLDYL 297
lcl|XM_00567303 ITELANALSYCHSKRVIHRDIKPENLLLGSAGELKIADFGWSVHAPSSRRTTLCGTLDYL 296
lcl|XM_00148927 ITELANALAYCHSKRVIHRDIKPENLLLGSAGELKIADFGWSVHAPSSRRTTLCGTLDYL 296
lcl|XM_00443024 ITELANALAYCHSKRVIHRDIKPENLLLGSAGELKIADFGWSVHAPSSRRTTLCGTLDYL 297
lcl|XM_00827414 ITELANALLYCHSKRVIHRDIKPENLLLGSAGELKIADFGWSVHAPSSRRTTLCGTLDYL 295
lcl|XM_01452869 ITELTNALSYCHSKRVIHRDIKPENLLLGSTGELKIADFGWSVHAPSSRRTTLCGTLDYL 296
lcl|XM_00468690 ITELADALSYCHSKRVIHRDIKPENLLLGSAGELKIADFGWSVHAPSSRRTTLCGTLDYL 297
lcl|XM_00341990 ITELADALSYCHSKKVIHRDIKPENLLLGSTGELKIADFGWSVHAPSSRRTTLCGTLDYL 298
lcl|XM_00432103 ITELANALSYCHSKRVIHRDIKPENLLLGSSGELKIADFGWSVHAPSSRRTTLCGTLDYL 296
                ****::** *****:***************:*****************************

lcl|NM_198436.2 PPEMIEGRMHDEKVDLWSLGVLCYEFLVGKPPFEANTYQETYKRISRVEFTFPDFVTEGA 356
lcl|XM_01514873 PPEMIEGRMHDEKVDLWSLGVLCYEFLVGKPPFEANTYQETYKRISRVEFTFPDFVTEGA 356
lcl|NM_00103802 PPEMIEGRMHDEKVDLWSLGVLCYEFLVGKPPFEADTYQETYRRISRVEFTFPDCVPEGA 356
lcl|NM_00114250 PPEMIEGRMHDEKVDLWSLGVLCYEFLVGKPPFEADTYQETYRRISRVEFTFPDCVPEGA 356
lcl|XM_00563519 PPEMIEGRMHDEKVDLWSLGVLCYEFLVGKPPFEASTYQETYKRISRVEFTFPDFVPEGA 357
lcl|XM_00398335 PPEMIEGRMHDEKVDLWSLGVLCYEFLVGKPPFEASTYQETYKRISRVEFTFPDFVPEGA 357
lcl|XM_00567303 PPEMIEGRMHDEKVDLWSLGVLCYEFLVGKPPFEANTYQETYKRISRVEFTFPDFVPEGA 356
lcl|XM_00148927 PPEMIEGRMHDEKVDLWSLGVLCYEFLVGKPPFEASTYQETYKRISRVEFTFPDFVPEGA 356
lcl|XM_00443024 PPEMIEGRMHDEKVDLWSLGVLCYEFLVGKPPFEASTYQETYKRISRVEFTFPDFVPDGA 357
lcl|XM_00827414 PPEMIEGRMHDEKVDLWSLGVLCYEFLVGKPPFEANTYQETYRRISRVEFTFPDFVTEGA 355
lcl|XM_01452869 PPEMIEGRMHDEKVDLWSLGVLCYEFLVGKPPFEASTYQETYKRISRVEFTFPDFVREGA 356
lcl|XM_00468690 PPEMIEGRMHDEKVDLWSLGVLCYEFLVGKPPFEASTYQETYKRISRVEFTFPDFVPEGA 357
lcl|XM_00341990 PPEMIEGRMHDEKVDLWSLGVLCYEFLVGKPPFEASTYQETYNRISRVEFTFPDLVTVGA 358
lcl|XM_00432103 PPEMIEGRMHDEKVDLWSLGVLCYEFLVGKPPFEANTYQETYKRISRVEFTFPDFVPEGA 356
                ***********************************.******.*********** *  **

lcl|NM_198436.2 RDLISRLLKHNPSQRPMLREVLEHPWITANSSKPSNCQNKESASKQS*- 404
lcl|XM_01514873 RDLISRLLKHNPSQRPMLREVLEHPWITANSSKPSNCQNRVN*------ 399
lcl|NM_00103802 RDLISRLLKHNPSQRPTLKEVLEHPWIIANSKPSSCQKKESTSKQS*-- 403
lcl|NM_00114250 RDLISRLLKHNPSQRPTLKEVLEHPWITANSKPSSCQKKESTSKQS*-- 403
lcl|XM_00563519 RDLISRLLKHNPSQRPTLKDVLEHPWIMANSSKPSSSQKNKDSTSKQS* 406
lcl|XM_00398335 RDLISRLLKHNPSQRPTLKEVLEHPWVTANSSKPSGSQKSKESTGKQS* 406
lcl|XM_00567303 RDLISRLLKHNPSHRPTLKEVLEHPWITANSKPASSHKKESTSKQP*-- 403
lcl|XM_00148927 RDLISRLLKHNPSQRPTLKEVLEHPWITANSSKPSNSQKSKESTSKQP* 405
lcl|XM_00443024 RDLISRLLKHNPSQRPTLKEVLEHPWITANASKPSNSQKSKESTSKQS* 406
lcl|XM_00827414 KDFISRLLKHNPSHRPTLAEVLEHPWVKAKSSKPSSCPDKEPASKQS*- 403
lcl|XM_01452869 RDLISRLLMHNPNQRLTLKEVLEHPWITANSSKPSSSQKSKESTSKQS* 405
lcl|XM_00468690 RDFISRLLKHNPSQRPTLKEVLEHPWITANSSKPPSSQKNKESTSKQS* 406
lcl|XM_00341990 RDLVSRLLKHNPSQRLTLKEVLEHPWITANSKKPPSSQNKEPTSKQS*- 406
lcl|XM_00432103 RDLISRLLKHNPSQRPTLKEVLEHPWITANSKPSSSQKKESASKQS*-- 403
                :*::**** ***.:*  * :******: *::. ..   .
```
