# Supplementary material for: Differential Selective Pressures Experienced by the Aurora Kinase Gene Family
Source: Int J Mol Sci. 2017 Dec 28;19(1):72. doi: 10.3390/ijms19010072 (PMC5796022; doi:10.3390/ijms19010072)
Supplement: Supplementary file 1 [file ijms-19-00072-s001.zip › FigureS4AKBalignmentwLegend.pdf]

Figure S4. Alignment of placental mammal AKB sequences. The alignment of the placental mammal AKB sequences used for the dN/dS analyses.

```
MATLAB multiple sequence alignment


lcl|NM_004217.3 MAQKENSYPWPYGRQTAPSGLSTLPQRVLRKEPVTPSALVLMSRSNVQPTAAPGQKVM-E  59
lcl|XM_01511855 MAQKENAYPWPYGRQTAPSGLSTLPQRVLRKEPVTPSALVLMSRSNVQPTAAPGQKVV-E  59
lcl|NM_183084.2 MAQKENAYPWPYGRQTAQPGLNTLPQRVLRKEPVTPSALVLMSRSNAQPTAAPGQKVV-E  59
lcl|XM_00401268 MAQKENAYPWPYGRQTAQPGLNTLPQRVLRKEAVIPSALVLMSRSNAQPTAAPGQKVV-E  59
lcl|XM_844813.4 MTQKENAYPWPYGRQTTQPGLNTLPQRVLRKDPATPSALVLMSRSNGQPTAAPGQKVV-E  59
lcl|XM_00399619 MAQKENAYPWPYGRQTTQPGLNTLPQRVLRKEPATPSALVLMSRSNTQPTAAPGQKVV-E  59
lcl|NM_213919.2 MAQKENTYPWPYGRQTAQSGLNILPQRVLRKEAVTPSALVLMSRSNTQPTAAPGQKVV-E  59
lcl|XM_00150481 MTQKENAYPWPYGRQTAQSGLNTLPQRVLRKEPATPSALVLMSRSNAQPTVALGQKVM-E  59
lcl|XM_00443309 MAQKENAYPWPYGRQTAQSGLNTLPQRVLRKEPVTPSALVLMSRSNAQPTAALGQKVV-E  59
lcl|XM_00827074 MAQKENAYPWPYSRPTPQSGLNTLPQRILRKEAATPSALVLLNRSNAPPTAVLGQKGM-E  59
lcl|XM_00587878 MTQKENAYPWPYGRQTAQCGLNTLPQRVLQKDPTTPSSLVLMSRSNAQPTAAPG-----Q  55
lcl|XM_00468461 MAQKENAYPWPYGRQTAQSGLNTLPQRVLRKDPTTPSALVLMNRSNTQPTAAPGQKVV-E  59
lcl|XM_00341691 MAQKENAYPWPYGRQTAQSGLNTLPQRVFRKDLATPSALVLMSRSNAQPTAAPGEKKMTE  60
lcl|XM_01994424 MAQKENAYPWPYGRQTAQSGLNTLPQRVLRKEPVTPSALVLMSRSNAQPTAAPLQKVV-E  59
                *:****:*****.* *.  **. ****:::*: . **:***:.***  **..       :

lcl|NM_004217.3 NSSGTPDILTRHFTIDDFEIGRPLGKGKFGNVYLAREKKSHFIVALKVLFKSQIEKEGVE 119
lcl|XM_01511855 NSSGTPNILMRHFTIDDFEIGRPLGKGKFGNVYLAREKKSHFIVALKVLFKSQIEKEGVE 119
lcl|NM_183084.2 NSSGTPNIPKRSFTIDDFEIGRPLGKGKFGNVYLAREKKSHFIVALKVLFKSQIEKEGVE 119
lcl|XM_00401268 NSSGTLNIPKRSFTIDDFEIGRPLGKGKFGNVYLAREKKSHFIVALKVLFKSQIEKEGVE 119
lcl|XM_844813.4 NSSGIPNFSMRSFTIDDFEIGRPLGKGKFGNVYLAREKKSHFIVALKVLFKSQIEKEGVE 119
lcl|XM_00399619 NSSGTPNFSMRSFTIDDFEIGRPLGKGKFGNVYLAREKKSHFIVALKVLFKSQIEKEGVE 119
lcl|NM_213919.2 NSSGTPNFSTRSFTIDDFEIGRPLGKGKFGNVYLAREKKSHFIVALKVLFKSQIEKEGVE 119
lcl|XM_00150481 NSSGTPNLLMRSFTIDDFEIGRPLGKGKFGNVYLAREKKSHFIVALKVLFKSQIEKEGVE 119
lcl|XM_00443309 NSSGTPNLLMRSFTIDDFEIGRPLGKGKFGNVYLAREKKSHFIVALKVLFKSQIEKEGVE 119
lcl|XM_00827074 NSSGAPNF-TRPFTIDDFEIGRPLGKGKFGNVYLAREKKSHFIVALKVLFKSQIEKEGVE 118
lcl|XM_00587878 NSNGTPN-LMRSFTIDDFEIGRPLGKGKFGNVYLAREKKSRFIVALKVLFKSQIEKEGVE 114
lcl|XM_00468461 NSSGTPNLSMRSFTIDDFEIGRPLGKGKFGNVYLAREKKSHFIVALKVLFKSQIEKEGVE 119
lcl|XM_00341691 NSSGTPSFLMRPFTIDDFEIGRPLGKGKFGNVYLAREKKSHFIVALKVLFKSQIEKEGVE 120
lcl|XM_01994424 NNSRTPNFSMRSFTIDDFEIGRPLGKGKFGNVYLAREKKSHFIVALKVLFKSQIEKEGVE 119
                *..   .   * ****************************:*******************

lcl|NM_004217.3 HQLRREIEIQAHLHHPNILRLYNYFYDRRRIYLILEYAPRGELYKELQKSCTFDEQRTAT 179
lcl|XM_01511855 HQLRREIEIQAHLHHPNILRLYNYFYDRRRIYLILEYAPRGELYKELQKSRTFDEQRTAT 179
lcl|NM_183084.2 HQLRREIEIQAHLQHPNILRLYNYFYDRRRIYLILEYAPRGELYKELQKSRTFDEQRTAT 179
lcl|XM_00401268 HQLRREIEIQAHLQHPNILRLYNYFYDRRRIYLILEYAPRGELYKELQKSRTFDEQRTAT 179
lcl|XM_844813.4 HQLRREIEIQAHLQHPNILRLYNYFYDRRRIYLILEYAPRGELYKELQKSHTFDEQRTAT 179
lcl|XM_00399619 HQLRREIEIQAHLQHPNILRLYNYFYDRRRIYLILEYAPRGELYKELQKSRTFDEQRTAT 179
lcl|NM_213919.2 HQLRREIEIQAHLQHPNILRLYNYFYDRRRIYLILEYAPRGELYKELQKCRTFDEQRTAT 179
lcl|XM_00150481 HQLRREIEIQAHLQHPNILRLYNYFYDRRRIYLILEYAPRGELYKELQKSRTFDEQRTAT 179
lcl|XM_00443309 HQLRREIEIQAHLQHPNILRLYNYFYDRRRIYLILEYAPRGELYKELQKSRTFDEQRTAT 179
lcl|XM_00827074 HQLRREIEIQAHLQHPNILRLYNYFYDRRRIYLILEYAPRGELYKELQKSRTFDEQRTAT 178
lcl|XM_00587878 HQLRREIEIQAHLQHPNILRLYNYFYDRRRIYLILEYAPRGELYKELQKSRTFDQQRTAT 174
lcl|XM_00468461 HQLRREIEIQAHLQHPNILRLYNYFYDRRRIYLILEYAPRGELYKELQKSRTFDEQRTAT 179
lcl|XM_00341691 HQLRREIEIQAHLQHPNILRLYNYFYDRRRVYLILEYAPRGELYKELQKSRTFDEQRTAT 180
lcl|XM_01994424 HQLRREIEIQAHLQHPNILRLYNYFYDRRRIYLILEYAPRGELYKELQKSHTFDEQRTAT 179
                *************:****************:******************. ***:*****

lcl|NM_004217.3 IMEELADALMYCHGKKVIHRDIKPENLLLGLKGELKIADFGWSVHAPSLRRKTMCGTLDY 239
lcl|XM_01511855 IMEELADALMYCHGKKVIHRDIKPENLLLGLKGELKIADFGWSVHAPSLRRKTMCGTLDY 239
lcl|NM_183084.2 IMEELADALTYCHAKKVIHRDIKPENLLLGLRGELKIADFGWSVHAPSLRRKTMCGTLDY 239
lcl|XM_00401268 IMEELADALTYCHAKKVIHRDIKPENLLLGLRGELKIADFGWSVHAPSLRRKTMCGTLDY 239
lcl|XM_844813.4 IMEELADALMYCHGKKVIHRDIKPENLLLGLQGELKIADFGWSVHAPSLRRKTMCGTLDY 239
lcl|XM_00399619 IMEELADALLYCHGKKVIHRDIKPENLLLGLQGELKIADFGWSVHAPSLRRKTMCGTLDY 239
lcl|NM_213919.2 IMEELADALIYCHGKKVIHRDIKPENLLLGLQGELKIADFGWSVHAPSLRRKTMCGTLDY 239
lcl|XM_00150481 IMEELADALMYCHGKKVIHRDIKPENLLLGLQGELKIADFGWSVHAPSLRRKTMCGTLDY 239
lcl|XM_00443309 IMEELADALIYCHGKKVIHRDIKPENLLLGLQGELKIADFGWSVHAPSLRRKTMCGTLDY 239
lcl|XM_00827074 IMEELADALRYCHEKKVIHRDIKPENLLLGLQGELKIADFGWSVHAPSLRRKTMCGTLDY 238
lcl|XM_00587878 IMEELADALMYCHKKKVIHRDIKPENLLLGLQGELKIADFGWSVHAPSLRRKTMCGTLDY 234
lcl|XM_00468461 IMEELADALIYCHGKKVIHRDIKPENLLLGLQGELKIADFGWSVHAPSLRRKTMCGTLDY 239
lcl|XM_00341691 IMEELADALMYCHGKKVIHRDIKPENLLLGLRGELKIADFGWSVHAPSLRRKTMCGTLDY 240
lcl|XM_01994424 IMEELADALIYCHGKKVIHRDIKPENLLLGLQGELKIADFGWSVHAPSLRRKTMCGTLDY 239
                ********* *** *****************:****************************

lcl|NM_004217.3 LPPEMIEGRMHNEKVDLWCIGVLCYELLVGNPPFESASHNETYRRIVKVDLKFPASVPMG 299
lcl|XM_01511855 LPPEMIEGRTHNEKVDLWCIGVLCYELLVGNPPFESASHNETYRRIVKVDLKFPASVPTG 299
lcl|NM_183084.2 LPPEMIEGRTHNEKVDLWCIGVLCYELLVGNPPFESASHNETYRRIVKVDLKFPPSVPLG 299
lcl|XM_00401268 LPPEMIEGRTHNEKVDLWCIGVLCYELLVGNPPFESASHNETYRRIVKVDLKFPPSVPMG 299
lcl|XM_844813.4 LPPEMIEGRMHNEKVDLWCIGVLCYELLVGNPPFESASHNETYRRIVKVDLKFPPSVPTG 299
lcl|XM_00399619 LPPEMIEGRTHNEKVDLWCIGVLCYELLVGNPPFESASHNETYRRIVKVDLKFPPSVPTG 299
lcl|NM_213919.2 LPPEMIEGRTHNEKVDLWCIGVLCYELLVGNPPFESASHNETYRRIVKVDLKFPPSVPAG 299
lcl|XM_00150481 LPPEMIEGRTHNEKVDLWCIGVLCYELLVGNPPFESASHNETYRRIVKVDLKFPPSVPAG 299
lcl|XM_00443309 LPPEMIEGRTHNEKVDLWCIGVLCYELLVGNPPFESASHNETYRRIVKVDLKFPASVPAG 299
lcl|XM_00827074 LPPEMIEGRTHNEKVDLWCIGVLCYELLVGNPPFESASHNETYRRIVKVDLKFPASVPAG 298
lcl|XM_00587878 LPPEMIEGRTHNEKVDLWCIGVLCYELLVGNPPFESASHNETYRRIVKVDLKFPPSVPVG 294
lcl|XM_00468461 LPPEMIEGRTHNEKVDLWCIGVLCYELLVGSPPFESASHNETYRRIVKVDLKFPLSVPSG 299
lcl|XM_00341691 LPPEMIEGRMHNEKVDLWCIGVLCYELLVGNPPFESASHNETYRRIVKVDLKFPPSVPTG 300
lcl|XM_01994424 LPPEMIEGRTHNEKVDLWCIGVLCYELLVGNPPFESASHNETYRRIVKVDLKIPPSMPAG 299
                ********* ********************.*********************:* *:* *

lcl|NM_004217.3 AQDLISKLLRHNPSERLPLAQVSAHPWVRANSRRVLPPSALQSVA* 345
lcl|XM_01511855 AQDLISKLLRHNPSERLPLAQVSAHPWVRANSRRVLPPSALQSVA* 345
lcl|NM_183084.2 AQDLIYKLLKHNPSERLPLAQVSAHPWVRTHSRRVLPPSAPQSVP* 345
lcl|XM_00401268 AQDLISKLLKHNPSERLPLAQVSAHPWVRAHSRRVLPPSAPQSVP* 345
lcl|XM_844813.4 AQDLISKLLKHNPSERLPLSQVSAHPWVRAHSRRMLPPSAVQAIP* 345
lcl|XM_00399619 AQDLISKLLKHNPSERLPLAQVSAHPWVRAHSRRVLPPSALQSVP* 345
lcl|NM_213919.2 AQDLISKLLKHNPSDRLPLAQVSAHPWVRAHSRRVLPPSAPQSVP* 345
```

```
lcl|XM_00150481 AQDLISKLLRHNPSERLPLAQVSAHPWVRAHSRRVLPPSALQSVP* 345
lcl|XM_00443309 AQDLISKLLKHNPSERLPLAQVSAHPWVRAHSRRVLPPSAPQSVP* 345
lcl|XM_00827074 AQDLISKLLRHNPSDRLTLAQVSAHPWVRAHSRRVLPPSAMQSVA* 344
lcl|XM_00587878 AQDLISKLLKHNPSDRLPLAQVSAHPWVRAHSRRVLPPSALQSVH* 340
lcl|XM_00468461 AQDLITKLLKHNPSERLPLTQVSAHPWVRAHSRRVLPPSALQSVP* 345
lcl|XM_00341691 AQDLISKLLKHNPSERLPLAQVSAHPWVRAHSRRVLPPSALQSVP* 346
lcl|XM_01994424 AQDLISKLLKHNPSERLPLAQVAAHPWVRAHSRRVLPPSALQSVP* 345
                ***** ***:****:**.*:**:******::***:***** *::
```
