# Supplementary material for: Differential Selective Pressures Experienced by the Aurora Kinase Gene Family
Source: Int J Mol Sci. 2017 Dec 28;19(1):72. doi: 10.3390/ijms19010072 (PMC5796022; doi:10.3390/ijms19010072)
Supplement: Supplementary file 1 [file ijms-19-00072-s001.zip › FigureS5AKCalignwLegend.pdf]

Figure S5. Alignment of placental mammal AKC sequences. The alignment of the placental mammal AKC sequences used for the dN/dS analyses.

```
MATLAB multiple sequence alignment


lcl|NM_00101587 MSSPRAVVQLGKAQPAGE------ELATANQTAQQPSSPAMRRLTVDDFEIGRPLGKGKF  54
lcl|XM_00109836 MSSPKAAVQLGRAQPAGE------AVTAANQTAQQPSSPAMRRLKIDDFEIGRPLGKGKF  54
lcl|XM_00521979 MSHRGTARKAGGMQHAAA------------AGQTVPGAPTARRFTVDDFEIGRPLGKGKF  48
lcl|XM_00401544 MSHRGTARKAGGMQPAAA------------AGQSVPGAPAARRFTIDDFEIGRPLGKGKF  48
lcl|XM_00561607 -------MLSGSAQPASE-GEKTQEQSKDAAGHVELSVPPGRRLTIDDFEIGRPLGKGKF  52
lcl|XM_00694080 -------MQSGSVQPAAEEEEQTRDQGTAAAGLVELGMPPVRRLTIEDFEIGRPLGKGKF  53
lcl|XM_02109730 -------MKSGSVQPAVA-----------AAGQTVSNIPTGRRLTIDDFEIGRPLGKGKF  42
lcl|XM_00150199 MSLPEAVMKSGSVRPAVP-----------TAGQSQPGFPNVRHLTIDDFEIGRPLGKGKF  49
lcl|XM_00443911 MSLPRAVMKLGDVQSAGE-----------TAGQTQPSFPTVRHFTIDDFEIGRPLGKGKF  49
lcl|XM_00824896 MQS-----------------------GSSQAAGPAQPSVPLRRLTIEDFEIGRPLGKGKF  37
lcl|XM_01453723 MNPGFLLSIIAEKKEPWP----------------SSPSPGSRSLTIDDFDIRCPLGKGKF  44
lcl|XM_01273470 -------MKSGDAQAAGA------------AGGPALPVATVRRLTIDDFEIGRPLGKGKF  41
lcl|XM_00340664 MFTNVALSFARREWKELA---------PTERALYGDVLLEMRRLRIDDFEIGRPLGKGKF  51
lcl|XM_01992887 -------MKSGSAQPAA------------VAGQTMPSIPTVRRLTIDDFEIGRPLGKGKF  41
                                                         * : ::**:*  *******

lcl|NM_00101587 GNVYLARLKESHFIVALKVLFKSQIEKEGLEHQLRREIEIQAHLQHPNILRLYNYFHDAR 114
lcl|XM_00109836 GNVYLARLKESHFIVALKVLFKSQIEKEGLEHQLRREIEIQAHLQHPNILRLYNYFHDAR 114
lcl|XM_00521979 GNVYLARLKKNHFIVALKVLFKSQIEKEGLEHQLRREVEIQAHLQHPNILRLYNYFHDAR 108
lcl|XM_00401544 GNVYLARLKKNHFIVALKVLFKSQIEKEGLEHQLRREVEIQAHLQHPNILRLYNYFHDAR 108
lcl|XM_00561607 GNVYLARLKESHFIVALKVLFKSQIEKEGLEHQLRREIEIQAHLQHPNILRLYNYFHDAR 112
lcl|XM_00694080 GNVYLARLKESHFLVALKVIFKSQIEKEGLEHQLRREIEIQAHLQHPNILRLYNYFHDAR 113
lcl|XM_02109730 GNVYLARLKESHFIVALKVLFKSQIEKEGLEHQLRREIEIQAHLQHPNILRLYNYFHDAR 102
lcl|XM_00150199 GNVYLARLKESHFIVALKVLFKSQIEKEGLEHQLRREIEIQAHLQHPNILRLYNYFHDAR 109
lcl|XM_00443911 GNVYLARLKESHFIVALKVLFKSQIEKEGLEHQLRREIEIQAHLQHPNILRLYNYFHDAR 109
lcl|XM_00824896 GNVYLARLKDSHFIVALKVLFKSQIEKEGMEHQLRREIEIQAHLQHPNILRLYNYFHDAR  97
lcl|XM_01453723 GNVYLARLRDSHFIVALKVLFKSQLEKEGMEHQLRREIEIQSHLQHPNILRLHNYFHDER 104
lcl|XM_01273470 GNVYLARLRESRFLVALKVLFKSQIEKEGLEHQLRREVEIQAHLRHPNILRLYNYFHDAR 101
lcl|XM_00340664 GNVYLARLQENHFLVALKVLFKSQIEKEGLEHQLRREIEIQAHLQHPNILRLYNYFHDSR 111
lcl|XM_01992887 GNVYLARLKESHFIVALKVLFKSQIEKEGLEHQLRREIEIQAHLQHPNILRLYNYFHDAR 101
                ********:..:*:*****:****:****:*******:***:**:*******:***** *

lcl|NM_00101587 RVYLILEYAPRGELYKELQKSEKLDEQRTATIIEELADALTYCHDKKVIHRDIKPENLLL 174
lcl|XM_00109836 RVYLILEFAPRGELYKELQKSEKLDEQRAATIIEELADALTYCHEKKVIHRDIKPENLLL 174
lcl|XM_00521979 RVYLILEYAPKGELYKALQRSHTFDEQRTATIIEELADALIYCHERKVIHRDIKPENLLL 168
lcl|XM_00401544 RVYLILEYAPKGELYKALQRSHTFDEQRTATIIEELADALIYCHEKKVIHRDIKPENLLL 168
lcl|XM_00561607 RVYLILEYAPRGELYKELQKSHTLDEQHTATIMEELADALTYCHEKKVIHRDIKPENLLL 172
lcl|XM_00694080 RVYLILEYAPRGELYKELQKSNTLDEQHTATIMEELADALTYCHEKKVIHRDIKPENLLL 173
lcl|XM_02109730 RVYLILEYAPRGELYKELQKSHTLDEQRTATIMEELADALTYCHEKKVIHRDIKPENLLL 162
lcl|XM_00150199 RVYLILEYAPRGELYKELQKSHTLDEQRTATIMEELADALTYCHEKKVIHRDIKPENLLL 169
lcl|XM_00443911 RVYLILEYAPRGELYKELQKSHTLDEQRTATIMEELADALTYCHEKKVIHRDIKPENLLL 169
lcl|XM_00824896 RIYLILEYAPRGELYKELQKCHTLDEQRTATIMEELADALTYCHGKKVIHRDIKPENLLL 157
lcl|XM_01453723 XVYLILEYAPRGELYKELQKSHTIDEQSTATIMEELAHALAYCHENKVIHRDIKPENLLL 164
lcl|XM_01273470 RVYLILEYAPRGELYKELQNCHTLDEQRTATIIEELADALTYCHEKKVIHRDIKPENLLL 161
lcl|XM_00340664 RVYLILEYAPRGELYKELQKSHRLDEQRTATIMEELADALIYCHEKKVIHRDIKPENLLL 171
lcl|XM_01992887 RMYLILEYAPRGELYKELQKSHTLDEQRTATVMEELADALTYCHEKKVIHRDIKPENLLL 161
                 :*****:**:***** **... :*** :**::****.** *** .**************

lcl|NM_00101587 GFRGEVKIADFGWSVHTPSLRRKTMCGTLDYLPPEMIEGRTYDEKVDLWCIGVLCYELLV 234
lcl|XM_00109836 GFRGEVKIADFGWSVHTPSLRRKTMCGTLDYLPPEMIEGRTYDEKVDLWCIGVLCYELLV 234
lcl|XM_00521979 GLMGEVKIADFGWSVHTPSLRRRTTCGTLDYLPPEMIEGRTYDEKVDLWCIGVLCYELLV 228
lcl|XM_00401544 GLMGEVKIADFGWSVHTPSLRRRTTCGTLDYLPPEMIEGRTYDEKVDLWCIGVLCYELLV 228
lcl|XM_00561607 GFKGEVKIADFGWSVHTPSLRRKTMCGTLDYLPPEMIERRTYNEKVDLWCIGVLCYELLV 232
lcl|XM_00694080 GFRGEVKIADFGWSVHTPSLRRKTMCGTLDYLPPEMIERRTYNETVDLWCIGVLCYELLV 233
lcl|XM_02109730 GFRGEVKIADFGWSVHTPSLRRKTMCGTLDYLPPEMIEGRTYNEKVDLWCIGVLCYELLV 222
lcl|XM_00150199 GFRGEVKIADFGWSVHTPSLRRKTMCGTLDYLPPEMIEGRTYNEKVDLWCIGVLCYELLV 229
lcl|XM_00443911 GFRGEVKIADFGWSVHTPSLRRKTMCGTLDYLPPEMIEGRTYNEKVDLWCIGVLCYELLV 229
lcl|XM_00824896 GFRGEVKIADFGWSVHTLSLRRKTMCGTLDYLPPEMIEGRTYDEKVDLWCIGVLCYELLV 217
lcl|XM_01453723 GLRGEVKIADFGWSVHTPSLRRKTMCGTLDYLPPEMVEQRTYSEMVDLWCIGVLCYELLV 224
lcl|XM_01273470 GYRGEVKIADFGWSVHTPSLRRKTMCGTLDYLPPEMIEGRTYNEKVDLWCIGVLCYELLV 221
lcl|XM_00340664 GLRGEVKIADFGWSVHTLSLRRKTMCGTLDYLPPEMIEGRTYDERVDLWCIGVLCYELLV 231
lcl|XM_01992887 GFRGEVKIADFGWSVHTPSLRRRTMCGTLDYLPPEMIEGRTYNEKVDLWCIGVLCYELLV 221
                *  ************** ****:* ***********:* ***.* ***************

lcl|NM_00101587 GYPPFESASHSETYRRILKVDVRFPLSMPLGARDLISRLLRYQPLERLPLAQILKHPWVQ 294
```

```
lcl|XM_00109836 GYPPFESTSHSETYRRILKVDVRFPLSMPLGARDLISKLLRYQPLERLPLAQILKHPWVQ 294
lcl|XM_00521979 GNPPFESASTSETYRRILKVDLRFPPSMSSGARDLISKLLRFQPLERLPLVRVLEHPWIR 288
lcl|XM_00401544 GNPPFESASTSETYRRILKVDLRFPPSMSSGARDLISKLLRFQPLERLPLVRVLEHPWIR 288
lcl|XM_00561607 GYPPFESPSHNETYRRILKVDLRFPPSLPLGAQDLVSKLLRYQPLERLPLAQIMEHPWVR 292
lcl|XM_00694080 GNPPFESHSHNETYRRILKVDIRFPPSIPLGARDLISKLLRYQPLERLPLSQILEHPWVR 293
lcl|XM_02109730 GNPPFESASHNETYRRILKVDVRFPPSLPLGAQDLISRLLRYQPSDRLPLDQILEHPWVR 282
lcl|XM_00150199 GNPPFESNSHNETYRRILKVDVRFPPSIPSGAKDLISKLLRYQPLERLPLDQILEHPWVR 289
lcl|XM_00443911 GNPPFESNSHNETYRRILKVDVRFPPSIPLGAKDLISKLLRYQPLERLPLDQIMEHPWVR 289
lcl|XM_00824896 GNPPFESVSHTETYRRILKVDVKFPVTMSLGARDLISRLLRYQPSERLPLAQILRHPWVQ 277
lcl|XM_01453723 GNPPFKSSSYSETYRRILKVDVKFPPSIPLGAQDLISKLLRYQPSERLLLAQVLQHPWVQ 284
lcl|XM_01273470 GKPPFESSTHNETYRRILKVDVRFPPSVPLGAQDLISSLLRYQPSERLPLAQILQHPWVR 281
lcl|XM_00340664 GNPPFESPSHTETYRRILKVDVRFPSSMPSGAQDLISKLLRYQPFERLALAQVLEHPWVQ 291
lcl|XM_01992887 GNPPFESPSHNETYRRILQVDVRFPPSMPLGAQDLISKLLRYQPSERLPLVQILEHPWVR 281
                * ***:* : .*******:**::** ::. **:**:* ***:** :** * :::.***::

lcl|NM_00101587 AHSRRVLPPCAQMAS* 310
lcl|XM_00109836 VHSRRVLPPCAQMAS* 310
lcl|XM_00521979 AHSQRVLPPSVPMAF* 304
lcl|XM_00401544 AHSQRVLPPSVQRAF* 304
lcl|XM_00561607 AHSRRVLPPSVQMAS* 308
lcl|XM_00694080 AHSRRVVPPSVQMDS* 309
lcl|XM_02109730 AHSRRVLPPSTQMVS* 298
lcl|XM_00150199 AHSRRVLPPSAQMAS* 305
lcl|XM_00443911 AHSRRVLPPSAQMAS* 305
lcl|XM_00824896 AHSRRVLPPCAHPAS* 293
lcl|XM_01453723 AHSRRVGPPPAPSAS* 300
lcl|XM_01273470 MHSRRVLPPSAQVAS* 297
lcl|XM_00340664 AHSQRVLPPSAQMAS* 307
lcl|XM_01992887 AHSRRVLPPSAQMVS* 297
                 **:** **  .
```
