# Supplementary figures and images for: Differential Selective Pressures Experienced by the Aurora Kinase Gene Family
Source: Int J Mol Sci. 2017 Dec 28;19(1):72. doi: 10.3390/ijms19010072 (PMC5796022; doi:10.3390/ijms19010072)

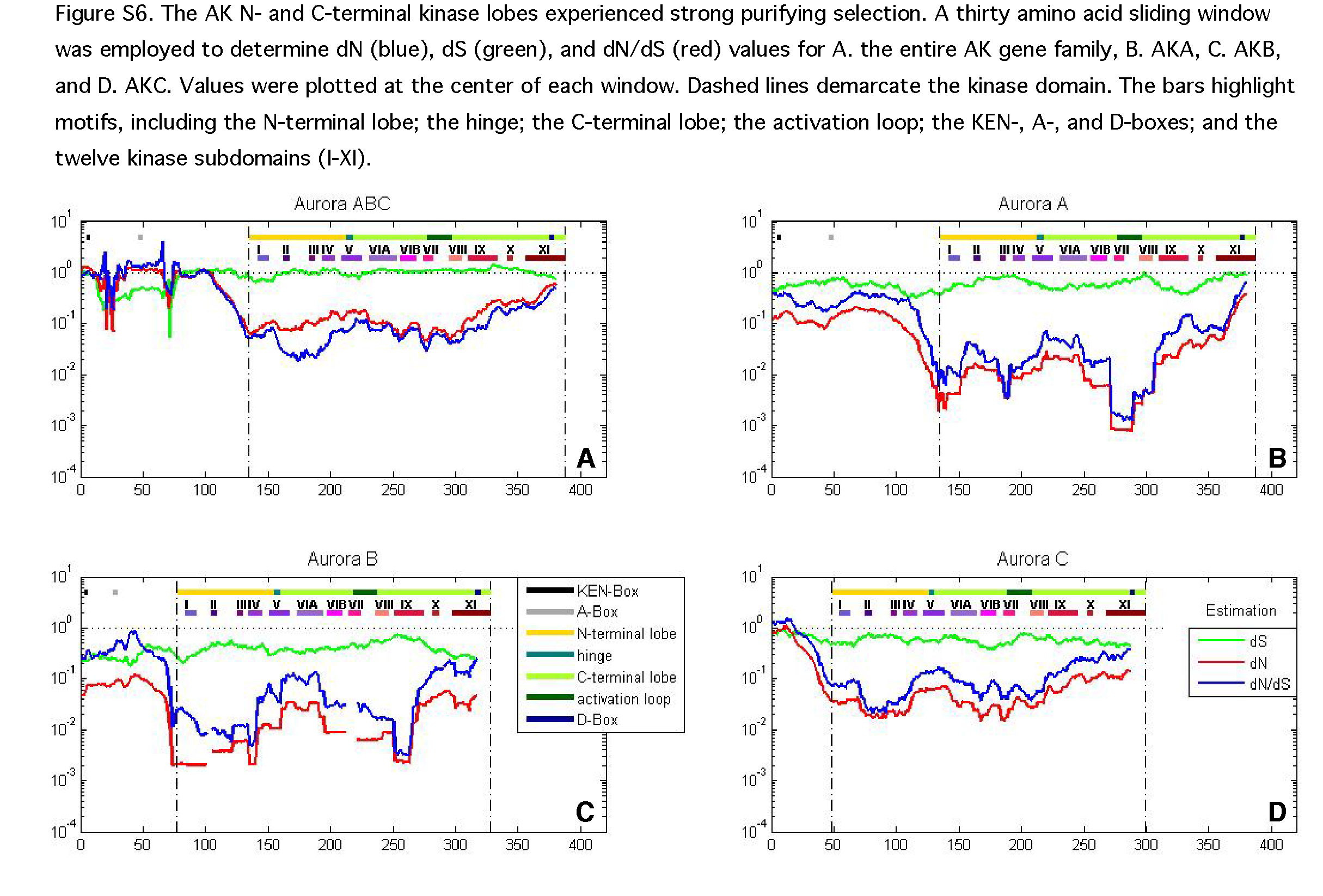

Supplement: Supplementary file 1 [file ijms-19-00072-s001.zip › FigureS6wLegendRev.tif]

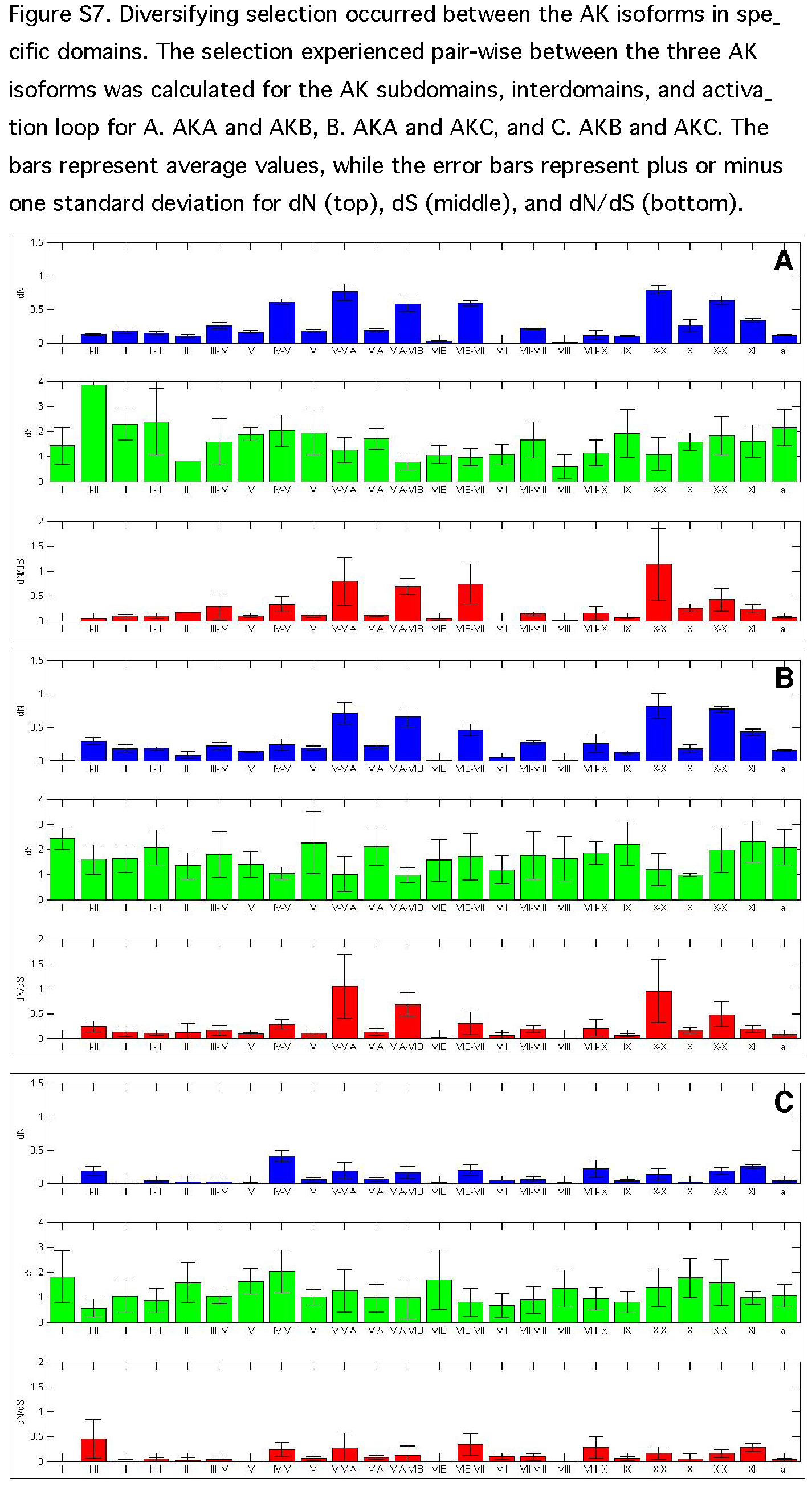

Supplement: Supplementary file 1 [file ijms-19-00072-s001.zip › FigureS7wLegendRev.tif]
